# Targeting arginine metabolism reverses bone immunosuppressive microenvironment and metastasis in *ARID1A*-deficient triple negative breast cancer

**DOI:** 10.1038/s41467-026-73574-3

**Published:** 2026-05-26

**Authors:** Shuangyue Pan, Jinyan Wang, Boya Wang, Fangqian Wang, Qingjian Chen, Tiantian Liu, Aima Zhang, Shenyuqi Wu, Bin Li, Hai Hu, Mengdi Yang, Zhonghua Tao, Xichun Hu

**Affiliations:** 1 Department of Medical Oncology, Fudan University Shanghai Cancer Center, Shanghai, China; 2 Department of Oncology, Shanghai Medical College of Fudan University, Shanghai, China; 3 Department of Orthopedic Surgery, The Second Affliated Hospital of Zhejiang University School of Medicine, Zhejiang University, Hangzhou, China; 4 Department of Oncology, State Key Laboratory of Systems Medicine for Cancer, Shanghai General Hospital, Shanghai Jiao Tong University School of Medicine, Shanghai, China; 5 Breast Cancer Center, Zhejiang Cancer Hospital, Hangzhou Institute of Medicine, Chinese Academy of Sciences, Hangzhou, China

**Keywords:** Bone metastases, Breast cancer, Cancer metabolism

## Abstract

Bone metastasis is a lethal consequence of breast cancer. AT-rich interaction domain 1 A gene (*ARID1A*), a subunit of the switch/sucrose non-fermentable (SWI/SNF) complex, regulates immunosuppressive tumor microenvironment. However, its specific role in bone metastasis remains unclear. Here, we show that female patients with *ARID1A*‑mutated triple negative breast cancer (TNBC) exhibit a higher bone metastasis incidence. Most *ARID1A* mutations result in loss of protein expression. *ARID1A* deficiency upregulates the arginine metabolic pathway, increasing ornithine and spermine levels that promote the expansion of polymorphonuclear myeloid-derived suppressor cells (PMN-MDSCs) within the bone marrow, thereby facilitating bone metastasis. Targeting key enzymes of arginase 2 (ARG2) and ornithine decarboxylase 1 (ODC1) in arginine metabolic pathway effectively reduces bone metastasis in *ARID1A*-deficient models. Collectively, these findings reveal that *ARID1A* deficiency promotes bone metastasis by activating arginine metabolism to expand PMN-MDSCs and potentially offers therapeutic strategies for preventing bone metastasis in female patients with *ARID1A*-deficient TNBC.

## Abstract (web summary)

The mechanisms driving bone metastasis in triple-negative breast cancer remain incompletely understood. Here, the authors show that loss of the SWI/SNF subunit ARID1A rewires arginine metabolism, fostering an immunosuppressive bone microenvironment and targeting this metabolic pathway significantly reduces bone metastatic burden.

## Introduction

Triple negative breast cancer (TNBC) is the most aggressive subtype of breast cancer, characterized by high metastasis incidence and poor prognosis^1,2^. Bone metastasis not only causes significant morbidity but also serves as a critical hub for tumor cell dissemination, facilitating secondary metastases to vital organs such as the lung, liver, and brain. The bone microenvironment provides a supportive niche for circulating tumor cells, enabling their survival, dormancy, and eventual reactivation, thereby accelerating disease progression and limiting therapeutic efficacy^3,4^. Thus, early detection and targeted interventions against bone metastasis are essential to mitigate morbidity and improve survival in TNBC patients.

As a core member of the mammalian chromatin remodeling complex switch/sucrose non-fermentable (SWI/SNF), the AT-rich interaction domain 1 A gene (*ARID1A*) mutations are common in multiple cancer types, including breast cancer^5,6^. *ARID1A* mutations, present in ~10% of tumors (about 7% in breast cancer)^7^, predominantly consist of inactivating mutations that drive loss of ARID1A protein, with its deficiency conferring poor clinical outcomes across multiple cancer types^8,9^. Emerging evidence highlights a role for *ARID1A* in regulating anti-tumor immunity^10–12^. Further, previous study disclosed that the role of *ARID1A* in cancer metastasis, demonstrating that *ARID1A* deficiency disrupts the regulation of epithelial-mesenchymal transition (EMT)^13,14^. However, the association between *ARID1A* deficiency and bone metastasis in TNBC has not yet been studied, and the underlying mechanisms remain unclear.

Amino acid metabolism has emerged as an indispensable and central determinant within the highly intricate and dynamic metabolic landscape of the tumor microenvironment (TME)^15^. Arginine metabolism has emerged as a highly versatile and pivotal process, exerting profound influences on both cancer cells and immune cells^16^. Emerging research suggests that *ARID1A* deficiency plays a crucial role in glycolysis and lipid metabolism alterations, promoting tumor growth and progression^17,18^. Despite these substantial insights into the metabolic reprogramming influenced by *ARID1A* deficiency, the potential crosstalk between *ARID1A* and arginine metabolism remains obscure. Additionally, it remains unclear which primary cell type in bone marrow engages with *ARID1A*-deficient TNBC cells through arginine metabolism.

The interaction among immune, stromal, and cancer cells shapes the TME, creating a tumor-permissive “soil” that facilitates breast cancer progression and relapse^19,20^. Myeloid-derived suppressor cells (MDSCs) are a heterogeneous population of immunosuppressive myeloid cells that play a crucial role in the TME^21^. In mouse tumor models, two main MDSC subtypes have been characterized: monocytic MDSCs (M-MDSCs) labelled as CD11b^+^Ly6G^low^Ly6C^+^ and polymorphonuclear MDSCs (PMN-MDSCs) labelled as CD11b^+^Ly6G^+^Ly6C^low22^. In humans, M-MDSCs are identified as CD11b⁺CD14^+^HLA-DR^-/low^CD66b^-^ and PMN-MDSCs are labelled as CD11b^+^CD14^-^ HLA-DR^-/low^CD66b^+^^22^. PMN-MDSCs represent a functional immunosuppressive state acquired by neutrophils under specific tumor microenvironmental pressures. PMN-MDSCs resemble neutrophils in morphology and are typically the dominant subset within the TME^23^. They facilitate tumor metastasis by promoting an immunosuppressive milieu, enhancing tumor cell extravasation, and contributing to the formation of pre-metastatic niches, thereby supporting tumor cell colonization at distant sites^24–26^. Deciphering how tumor cells regulate PMN-MDSCs mediated immunosuppression might provide more effective treatment strategies.

In this study, we integrated clinical cohort analysis, single-cell RNA sequencing (scRNA-seq), cell co-culture, and animal models to investigate the role and mechanisms of arginine metabolism in *ARID1A*-deficient TNBC bone metastasis. Through circulating tumor DNA (ctDNA) analysis of 663 breast cancer patients, we identified that *ARID1A* mutations significantly promote bone metastasis, particularly in TNBC. Further findings demonstrated that *ARID1A* deficiency in TNBC cells leads to increased biosynthesis of ornithine and spermine—mediated by activation of arginine metabolic pathways—thereby driving expansion of the PMN-MDSCs, formation of an immunosuppressive microenvironment in bone marrow, and promotion of bone metastasis in *ARID1A*-deficient TNBC. Targeting the arginine metabolic pathway effectively reduces bone metastasis in *ARID1A*-deficient models, highlighting its therapeutic potential.

## Results

### ARID1A mutations promote bone metastasis in breast cancer patients

**Fig. 1:**
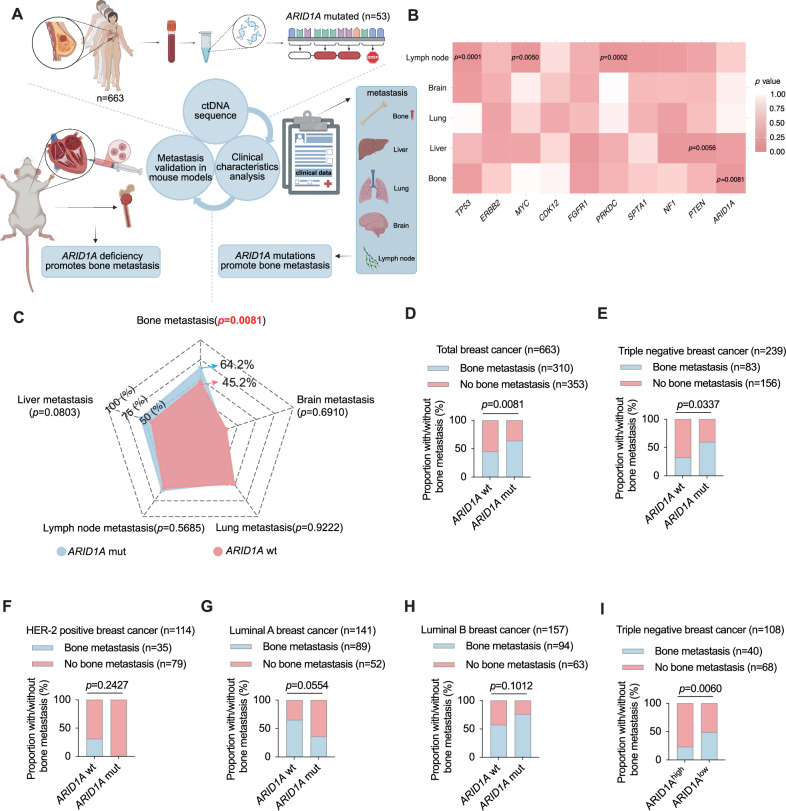
ARID1A mutations promote bone metastasis in breast cancer patients. **A** Schematic illustration of experiment design and patient sample processing procedure (*n* = 663 patients). Created in BioRender. Pan, S. (https://BioRender.com/1pl9j6l). **B** The differential statistical association between mutated genes and their metastatic sites. A two-sided Pearson’s chi-square test was used to evaluate the association between metastatic sites and gene mutations. **C** Radar chart depicting metastasis to multiple organs in patients with and without *ARID1A* mutations. Two-sided Pearson’s chi-square test was used. *ARID1A* wt, *ARID1A* wild-type. *ARID1A* mut, *ARID1A* mutations. **D** Statistical analysis of the incidence of bone metastasis in breast cancer patients with and without *ARID1A* mutations. Two-sided Pearson’s chi-square test was used. **E–H** Statistical analysis of the association between *ARID1A* mutations and bone metastasis in triple negative, HER-2 positive, luminal A and luminal B breast cancer, excluding 12 patients with unavailable subtype information. Two-sided Pearson’s chi-square test was used. **I** The association between ARID1A protein expression levels and bone metastasis in TNBC patients. A two-sided Pearson’s chi-square test was used. All *p* values are indicated in the figures. Source data are provided as a Source Data file.

We collected peripheral blood samples from 663 metastatic breast cancer patients at the Fudan University Shanghai Cancer Center (FUSCC) and performed ctDNA analysis (Fig. 1A). The study comprehensively identified mutations across multiple genes, with multi-gene mutations found to be associated with organ-specific metastasis. Significantly, among the top genes with the highest mutation rates we detected, mutations in *ARID1A* alone manifested a highly significant association with bone metastasis (Fig. 1B). *ARID1A* mutations were identified in 53 patients, and clinical data analysis showed that the incidence of bone metastasis was 64.2% in patients with *ARID1A* mutations, compared with 45.2% in those without *ARID1A* mutations, indicating that *ARID1A*-mutated cases had a significantly higher incidence of bone metastasis (Fig. 1C, D; *p* = 0.0081). Further, no statistically significant associations were observed in the liver (Supplementary Fig. 1A; *p* = 0.0803), lung (Supplementary Fig. 1B, *p* = 0.9222), brain (Supplementary Fig. 1C, *p* = 0.6910), or lymph node metastases (Supplementary Fig. 1D, *p* = 0.5685) between *ARID1A* wild-type and *ARID1A*-mutated groups (Supplementary Table 1). We also detected mutations in additional SWI/SNF subunits in our ctDNA cohort, with the following frequencies: *SMARCA4* in 20 patients (3.02%), *SMARCB1* in 3 (0.45%), *ARID1B* in 14 (2.11%), *ARID2* in 14 (2.11%), and *BRD7* in 2 patients (0.30%). Notably, none of these mutations showed a statistically significant correlation with bone metastasis (Supplementary Table 2). For further analysis, we divided 663 breast cancer patients into triple negative, luminal A, luminal B and human epidermal growth factor receptor 2 (HER2)-positive breast cancer, then evaluated the association between *ARID1A* mutations and bone metastasis. The results showed that *ARID1A* mutations were significantly more associated with bone metastasis in TNBC (Fig. 1E–H, and Supplementary Table 3). In our cohort, 68% (36/53) of *ARID1A* mutations were truncating or deletion events (Stop Gained, Frameshift variant, Splice Donor variant, and Copy Number Deletion), which are predicted to cause loss of protein function^27,28^. Thus, we hypothesized that *ARID1A* deficiency promotes bone metastasis in TNBC. To test this hypothesis at the protein level, we analyzed ARID1A expression in a set of tissue microarray (TMA) comprising 108 TNBC patients. Supporting our genetic findings, patients with low ARID1A expression exhibited a significantly higher incidence of bone metastasis (28/57, 49.1%) than those with high expression (12/51, 23.5%, *p* = 0.0060; Fig. 1I). The baseline characteristics are presented in supplementary Table 4. These findings collectively suggest that ARID1A deficiency plays a crucial role in promoting TNBC bone metastasis.

### ARID1A deficiency promotes bone metastasis of TNBC

**Fig. 2:**
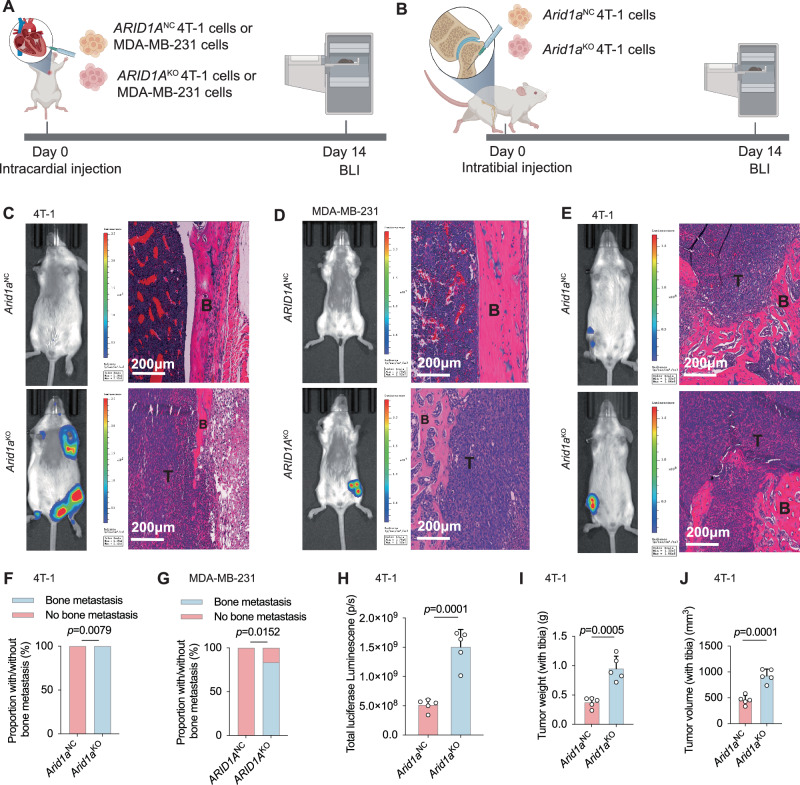
ARID1A deficiency promotes bone metastasis of breast cancer. **A**,** B** Experimental design of intracardiac and intratibial injection mouse models for bone metastasis. BLI, bioluminescence imaging. Created in BioRender. Pan, S. (https://BioRender.com/kxvt4p7) and (https://BioRender.com/9x1stls). **C** Representative BLI, H&E staining images of two groups of mice following intracardiac injection with equal numbers of *Arid1a*^NC^ and *Arid1a*^KO^ 4T-1 cells. B bone; T tumor; Scale bar, 200 μm. *n* = 5 mice per group. **D** Representative BLI, H&E staining images of two groups of mice following intracardiac injection with equal numbers of *ARID1A*^NC^ and *ARID1A*^KO^ MDA-MB-231 cells. Scale bar, 200 μm. *n *= 6 mice per group. **E** Representative BLI, H&E staining images of two groups of mice following intratibial injection with equal numbers of *Arid1a*^NC^ and *Arid1a*^KO^ 4T-1 cells. Scale bar, 200 μm. *n* = 5 mice per group. **F** Statistical charts of bone metastasis incidence following intracardiac injection of *Arid1a*^NC^ and *Arid1a*^KO^ 4T-1 cells. *n* = 5 mice per group, two-sided Fisher’s exact test was used. **G** Following intracardiac injection of MDA-MB-231 cells, bone metastasis was detected in 5 mice (5/6) in the *ARID1A*^KO^ group, whereas no metastasis (0/6) was observed in the *ARID1A*^NC^ group. *n* = 6 mice per group, two-sided Fisher’s exact test was used. **H** Quantification of the BLI intensity in the right leg region of the two groups of mice after intratibial injection. Data are presented as mean ± SD (*n* = 5 mice per group), unpaired two-tailed Student’s t test was used. **I, J** Intratibial tumor progression was increased in mice injected with *Arid1a*^KO^ cells compared to those injected with *Arid1a*^NC^ cells, as demonstrated by tumor weight (**I**), and tumor volume (**J**). Data are presented as mean ± SD (*n* = 5 mice per group), unpaired two-tailed Student’s t test was used. All *p* values are indicated in the figures. Source data are provided as a Source Data file.

We established two murine models of bone metastasis: an intracardiac injection model to investigate systemic metastatic dissemination, coupled with an intratibial injection model specifically designed to evaluate orthotopic bone metastasis progression^29–33^. We injected the same number of luciferase-labeled *ARID1A*-negative control (*ARID1A*^NC^) and *ARID1A*-knockout (*ARID1A*^KO^) TNBC cells into the left ventricle or tibial of each mouse (Supplementary Fig. 2A). To monitor tumor progression in vivo, bioluminescence imaging (BLI) was performed (Fig. 2A, B). Mice were euthanized when BLI revealed a significant increase in total flux, indicating established bone metastasis. Histopathological confirmation of tumor invasion was subsequently performed through hematoxylin and eosin (H&E) staining of bone sections (Fig. 2C–E). In the model established by intracardiac injection of 4T‑1 cells into Balb/c mice, the incidence of bone metastasis was significantly higher in the *Arid1a*^KO^ group compared with the *Arid1a*^NC^ group, as confirmed by in vivo BLI and H&E staining (Fig. 2F). Similarly, in NOD-scid IL2Rγ^null^ (NSG) mice humanized with human CD34+ hematopoietic stem cells and injected with MDA‑MB‑231 cells, the incidence of bone metastasis was significantly greater in the KO group compared with the NC controls (Fig. 2G). In the intratibial injection model, an equal number of luciferase-labeled *Arid1a*^KO^ or *Arid1a*^NC^ 4T-1 cells were inoculated into the tibial marrow of each mouse. On the 14th day, quantification of the BLI intensity showed that mice in the *Arid1a*^KO^ group exhibited significantly higher intensity in the right leg region compared to the *Arid1a*^NC^ group (Fig. 2H). Mice were sacrificed and right hind legs were harvested. Tumor image, tumor weight, and tumor volume all demonstrated that intratibial tumor progression was more pronounced in mice injected with *Arid1a*^KO^ 4T-1 cells compared to the *Arid1a*^NC^ group (tumor volume: NC, 455.56 ± 91.95 mm^3^; KO, 926.62 ± 126.77 mm^3^; tumor weight: NC, 0.37 ± 0.09 g; KO, 0.95 ± 0.19 g; Supplementary Fig. 2B and Fig. 2I, J).

To further validate that *ARID1A* correlates with the bone metastasis in TNBC, we established an *Arid1a*-overexpression (*Arid1a*^OE^) 4T-1 cells (Supplementary Fig. 2C). The results revealed that *Arid1a*^KO^ significantly increased the intratibial tumor burden, whereas *Arid1a*^OE^ attenuated this effect compared to the other two groups (Supplementary Fig. 3A, B). On the 14th day, the mice were sacrificed and right hind legs were harvested. The weight and volume of intratibial tumors demonstrated that *Arid1a*^KO^ promoted tumor growth in bone, while *Arid1a*^OE^ reversed this effect (tumor volume: NC, 645.72 ± 32.21 mm^3^; KO, 1185.78 ± 48.27 mm^3^; OE, 470.42 ± 95.63 mm^3^; tumor weight: NC, 0.81 ± 0.06 g; KO, 1.09 ± 0.06 g; OE, 0.35 ± 0.10 g; Supplementary Fig. 3C–E). Taken together, these findings suggest that *Arid1a* deficiency could contribute to TNBC bone metastasis.

### *ARID1A* deficiency upregulates arginine metabolic pathway in TNBC

**Fig. 3:**
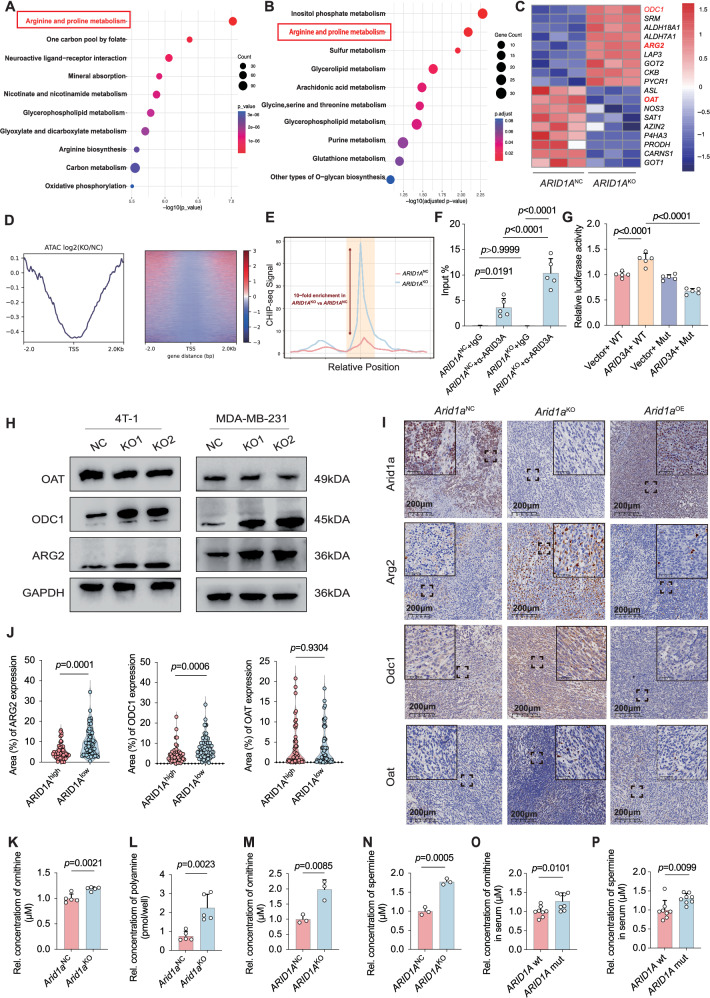
ARID1A deficiency promotes arginine pathway activation and the production of ornithine and spermine. **A**, **B** KEGG enrichment analysis of metabolic pathways in metabolomics (**A**) and transcriptomics (**B**) data from *ARID1A*^NC^ and *ARID1A*^KO^ cells (*n* = 3 biological replicates; one-sided hypergeometric test). **C** Heatmap of the most differentially expressed genes in the arginine metabolic pathway between *ARID1A*^NC^ and *ARID1A*^KO^ MDA-MB-231 cells. **D** ATAC signal at global chromatin accessibility in *ARID1A*^KO^ cells compared to *ARID1A*^NC^ cells. TSS, Transcription start site. **E** H3K27ac ChIP-seq signal enrichment at *ARG2* regulatory region in *ARID1A*^KO^ and *ARID1A*^NC^ MDA-MB-231 cells. **F** ChIP-qPCR assays of ARID3A binding in the *ARG2* enhancer. Data are presented as mean ± SD (*n* = 5 biological replicates), two-way ANOVA for multiple comparisons. Exact *p*‑values: *p* = 0.99997831 (*ARID1A*^NC^ + IgG vs. *ARID1A*^KO^ + IgG); *p* = 1.7 × 10^−7^ (*ARID1A*^KO^ + IgG vs. *ARID1A*^KO^ + α-ARID3A); *p* = 3.9 × 10^−5^ (*ARID1A*^NC^ + α-ARID3A vs. *ARID1A*^KO^ + α-ARID3A). **G** Luciferase activity of the *ARG2* enhancer upon *ARID3A* overexpression in MDA-MB-231 cells co-transfected with WT or Mut reporter constructs. Data are presented as mean ± SD (*n* = 5 biological replicates), two-way ANOVA for multiple comparisons. *p* = 5.9 × 10^−5^ (Vector+WT vs. *ARID3A* + WT); *p* = 3.2 × 10^−9^ (*ARID3A* + WT vs *ARID3A*+Mut); WT, wild-type; Mut, mutant. **H** Western blot analysis of the expression levels of ARG2, ODC1, OAT between *ARID1A*^NC^ and *ARID1A*^KO^ cells. The samples derive from the same experiment but different gels for ARG2, another for ODC1, and another for OAT were processed in parallel. The experiment was repeated independently three times. **I** Representative immunohistochemistry images of the tumors in each group. Scale bar, 200μm. **J** Quantification of ARG2, ODC1, OAT expression in TNBC tissue microarrays with low and high ARID1A expression. Total *n* = 108 patients, unpaired two-tailed Student’s t test. **K**,** L** ELISA results of ornithine and polyamine levels in tumors derived from *Arid1a*^KO^ versus *Arid1a*^NC^ 4T-1 cells. Data are presented as mean ± SD (*n* = 5 mice), unpaired two-tailed Student’s t test. **M, N** The LC-MS/MS results indicate an elevation in ornithine and spermine levels in the *ARID1A*^KO^ MDA-MB-231 cells. Data are presented as mean ± SD (*n* = 3 biological replicates), unpaired two-tailed Student’s t test. **O**,** P** The levels of ornithine and spermine in the peripheral blood serum of TNBC patients. Data are presented as mean ± SD (*n *= 8 patients), unpaired two-tailed Student’s t test. Source data are provided as a Source Data file.

To further explore the mechanisms underlying *ARID1A* deficiency-induced bone metastasis, we performed integrated metabolomic and transcriptome analyses on *ARID1A*^NC^ and *ARID1A*^KO^ cells. Transcriptomic and metabolomic profiling of *ARID1A*^KO^ and negative control cells revealed distinct global profiles for each group (Supplementary Fig. 4A, B). Volcano plots identified 1445 upregulated and 2835 downregulated genes, along with 693 upregulated and 710 downregulated metabolites (Supplementary Fig. 4C, D). Kyoto Encyclopedia of Genes and Genomes (KEGG) pathway enrichment analysis of metabolomics data revealed that arginine and proline metabolism was a significantly enriched pathway in the *ARID1A*^KO^ group (Fig. 3A). Consistently, transcriptomic analysis further supported this finding, showing pronounced enrichment of the arginine and proline metabolism pathway (Fig. 3B). The consistent dysregulation of arginine and proline metabolism across both omics layers implicates its centrality in *ARID1A* deficiency-associated bone metastasis, suggesting its potential role as a key metabolic node in this process.

As shown in Supplementary Fig. 4E, arginine is converted to ornithine by the key enzyme arginase 2 (ARG2), which serves as a precursor for polyamine synthesis via ornithine decarboxylase 1 (ODC1) and proline synthesis via ornithine aminotransferase (OAT)^34,35^. Through transcriptomic analysis of the arginine metabolic pathway, we identified the most differentially expressed genes between the two groups. The heatmap revealed that the key enzyme *ODC1* was the most significantly upregulated in *ARID1A*^KO^ MDA-MB-231 cells compared to the negative control cells. Concurrently, *ARG2* expression was also elevated, whereas OAT showed no significant increase (Fig. 3C). To explore the potential regulatory mechanism underlying low *ARID1A* expression-induced *ARG2* transcription activation, we performed Assay for transposase accessible chromatin (ATAC)-seq and Chromatin Immunoprecipitation-Sequencing (ChIP-seq) analysis in *ARID1A*^KO^ and negative control MDA-MB-231 cells. Our analyses revealed that *ARID1A* deficiency led to a global reduction in chromatin accessibility, indicating impaired SWI/SNF-dependent chromatin remodeling—consistent with *ARID1A*’s established role as a core subunit of the SWI/SNF complex (Fig. 3D). Notably, the H3K27ac enrichment at the specific regulatory region of *ARG2* was increased by 10-fold in *ARID1A*^KO^ MDA-MB-231 cells compared with control cells, indicating enhanced transcriptional potential of *ARG2* in the absence of *ARID1A* (Fig. 3E). Subsequent motif enrichment analysis of this differentially accessible region identified *ARID3A* as a highly enriched candidate transcription factor. To determine its functional role, we conducted siRNA-mediated knockdown of *ARID3A*, which significantly reduced the expression of *ARG2* (Supplementary Fig. 4F, G). This suggests that *ARID3A* may serve as a key transcriptional regulator mediating the upregulation of *ARG2* following *ARID1A* deficiency. We next performed ChIP-quantitative polymerase chain reaction (CHIP-qPCR) to investigate the binding of ARID3A to the regulatory region of *ARG2* and further localized this binding to a specific enhancer site (Fig. 3F). To determine whether this binding site is functionally required for enhancer activity, we cloned the enhancer fragment containing the wild-type (WT) binding site, alongside a corresponding mutant version (MUT) into the pGL3-promoter luciferase reporter plasmid. Dual-luciferase reporter assays revealed that the luciferase activity driven by the enhancer with the WT site was significantly higher than that with the MUT site, demonstrating that this binding site is essential for ARID3A to activate the enhancer of *ARG2* (Fig. 3G and Supplementary Fig. 4H). Consistently, we observed that the H3K27ac signal at this specific binding site was significantly elevated in *ARID1A*^KO^ cells compared to *ARID1A*^NC^ cells (Supplementary Fig. 4I).

To further verify the up-regulation of the arginine metabolic pathway in *ARID1A*^KO^ TNBC cells, reverse transcription  quantitative real-time polymerase chain reaction (RT­-qPCR) analysis was employed to detect the mRNA expression levels of *ARG2*, *ODC1*, and OAT in the *ARID1A*^NC^ and *ARID1A*^KO^ groups of 4T-1, BT-549, and MDA-MB-231 cells (Supplementary Fig. 4J-L). Additionally, we used western blotting analysis to detect the differences in the protein expression of ARG2, ODC1, and OAT between *ARID1A*^NC^ and *ARID1A*^KO^ cells (Fig. 3H). The results consistently indicated that the expression of ARG2 and ODC1 in *ARID1A*^KO^ TNBC cells was up-regulated compared to the negative control group, whereas there was no significant difference in the expression of OAT between the two groups. Furthermore, the RT-qPCR and western blotting analysis indicated that the expression of ARG2 and ODC1 in *ARID1A*^OE^ cells was lower than that in the negative control group, while there was no difference in the expression of OAT (Supplementary Fig. 4M, N).

We collected bone metastatic tumors from *Arid1a*^NC^, *Arid1a*^KO^, and *Arid1a*^OE^ groups of mice and performed immunohistochemistry (IHC) staining. The results revealed that the expression levels of Arg2 and Odc1 were upregulated in the *Arid1a*^KO^ group compared to the negative control group, while *Arid1a*^OE^ exhibited reduced expression of Arg2 and Odc1 compared to the other two groups. No significant differences in Oat expression were observed among the three groups (Fig. 3I and Supplementary Fig. 4O).

To determine the clinical relevance of arginine metabolic pathway, IHC staining in TNBC specimens (*n* = 108) was performed to detect the expression of ARG2, ODC1, and OAT in two groups based on high or low ARID1A expression levels (Fig. 3J). The analysis revealed a positive association between the expression of ARG2, ODC1 with low level of ARID1A, while there was no significant difference in OAT. These experiments suggest that ARID1A deficiency upregulates the arginine metabolic pathway.

### *ARID1A* deficiency promotes ornithine and spermine production in TNBC

Metabolomics analysis suggested elevated ornithine levels in *ARID1A*^KO^ cells (Supplementary Fig. 5A). To further confirm this finding, Enzyme-Linked Immunosorbent Assay (ELISA) was employed to quantify ornithine concentrations. The analysis revealed that *Arid1a*^KO^ cells exhibited higher ornithine concentrations both in tumor tissues and in vitro compared with the negative control group (Fig. 3K and Supplementary Fig. 5B). Given the observed upregulation of ODC1, a key enzyme in polyamine biosynthesis, we measured polyamine concentrations in *ARID1A*^KO^ and *ARID1A*^NC^ cells. We found that the polyamine concentration was significantly elevated in the *ARID1A*^KO^ group compared to the control group (Fig. 3L; Supplementary Fig. 5C–E). Polyamines, comprising putrescine, spermidine, and spermine, play a crucial role in cellular processes. To identify which specific polyamine showed increased levels, Liquid Chromatography-Tandem Mass Spectrometry (LC-MS/MS) was performed to profile the metabolites in arginine metabolic pathway. The analysis showed that in the *ARID1A*^KO^ group, the levels of ornithine and spermine were significantly increased (Fig. 3M, N), while the content of arginine decreased (Supplementary Fig. 5F).

To investigate the contribution of *ARID1A*-deficient TNBC cells to systemic ornithine and spermine concentrations, we measured the levels of ornithine and spermine in the peripheral serum of human and mice. We found that TNBC patients harboring *ARID1A* loss-of-function mutations exhibited significantly elevated serum levels of ornithine and spermine compared to those with wild-type *ARID1A* (Fig. 3O, P). Concurrently, in the mouse model, serum levels of ornithine and spermine were elevated in mice bearing bone metastases following intracardiac injection with *Arid1a*^KO^ 4T-1 cells versus those injected with *Arid1a*^NC^ 4T-1 cells (Supplementary Fig. 5G, H). Collectively, our results demonstrate that *ARID1A* deficiency in tumor cells not only enhances ornithine and spermine biosynthesis, but also leads to significantly elevated circulating levels of these metabolites in peripheral blood.

To dissect the roles of ornithine and spermine in bone metastasis, we measured the peripheral blood serum levels of ornithine and spermine in 40 TNBC patients. Patients were stratified into high and low-level groups based on the median concentration of these metabolites. We found that the incidence of bone metastasis was 15.0% (3/20) in the group with low ornithine levels, compared to 60.0% (12/20) in the group with high levels (*p* = 0.0033); similarly, it was 10.0% (2/20) in the low spermine group, versus 65.0% (13/20) in the high spermine group (*p* = 0.0003; Supplementary Fig. 5I, J). The clinical baseline characteristics of 40 patients were shown in Supplementary Table 5 and 6. Furthermore, to assess direct effects on cancer cells, we performed colony formation and wound healing assays in *ARID1A*^NC^ and *ARID1A*^KO^ MDA-MB-231 cells. Our findings reveal that ornithine and spermine do not directly affect cancer cell proliferation or migration capacity, suggesting their roles in bone metastasis rely on the microenvironment (Supplementary Fig. 5K-N).

### *ARID1A* deficiency promotes TNBC bone metastasis via expanding PMN-MDSCs in bone marrow

**Fig. 4:**
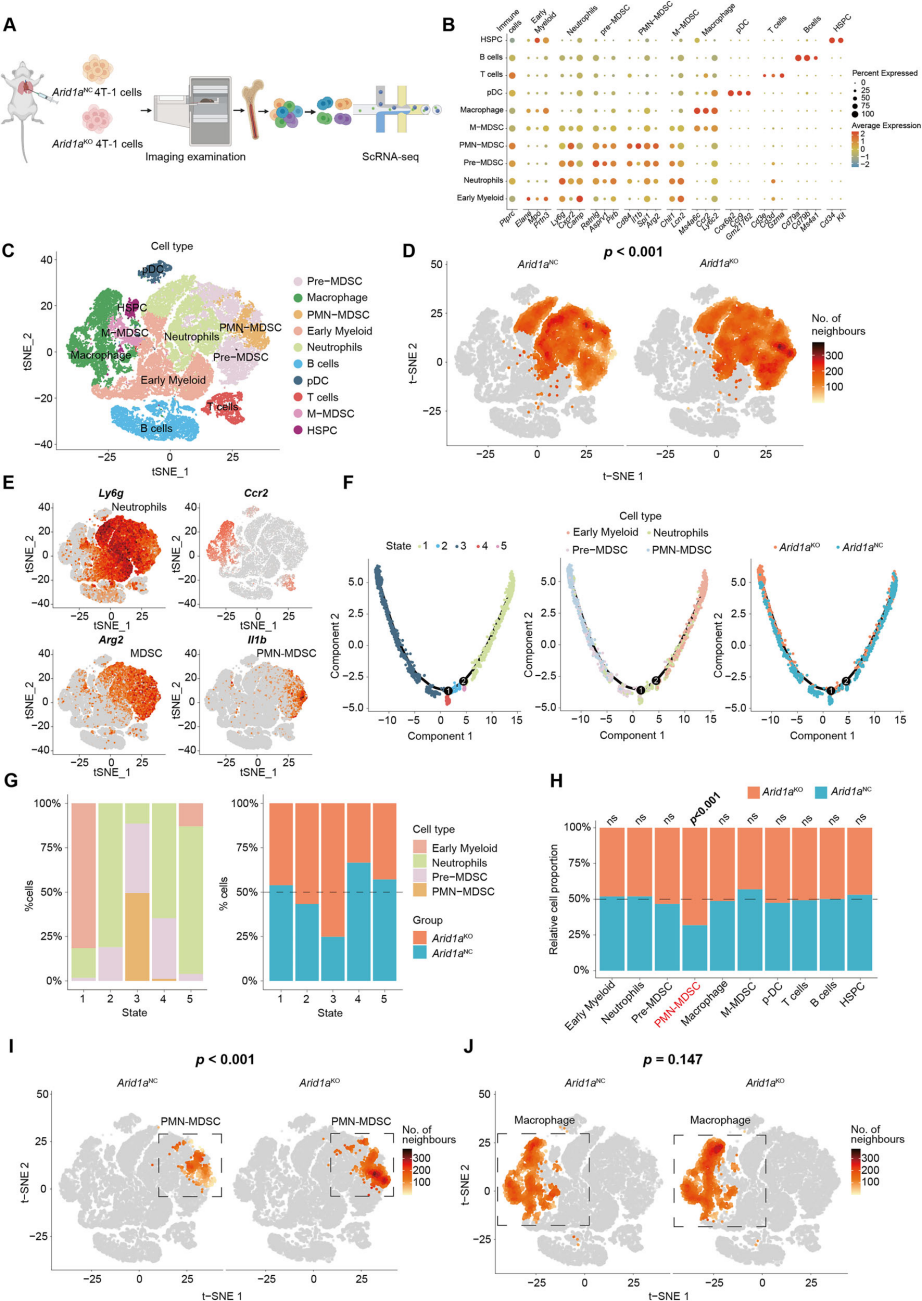
ARID1A deficiency facilitates TNBC bone metastasis via modulating PMN-MDSCs enrichment in bone marrow. **A** Schematic illustration of the intracardiac injection experimental design for scRNA-seq. *n* = 3 biological replicates. Created in BioRender. Pan, S. (https://BioRender.com/walt4i7). **B** A dot plot shows the gene expression of canonical marker genes. **C** tSNE plots of cells in mouse bone marrow following intracardiac injection of *Arid1a*^NC^ and *Arid1a*^KO^ 4T-1 cells. **D** Proportions of granulocytic myeloid cells between the *Arid1a*^NC^ and *Arid1a*^KO^ groups. Two‑sided Fisher’s exact test was used. **E** Feature plots of characteristic markers, with expression levels ranging from low (gray) to high (orange). **F** Monocle pseudotime analysis of neutrophil dynamics and early myeloid development within granulocytic myeloid clusters. **G** State3, predominantly composed of PMN-MDSCs, exhibited a higher proportion of cells in the *Arid1a*^KO^ group. **H** A comparison of the proportional counts of all immune cells in the bone marrow between the *Arid1a*^NC^ and *Arid1a*^KO^ groups. **I**,** J** Proportions of PMN-MDSCs and macrophages in the *Arid1a*^NC^ and *Arid1a*^KO^ groups. n.s., not significant. Two-sided Fisher’s exact test was used. Other *p* values are indicated in the figures. Source data are provided as a Source Data file.

Thus, scRNA-seq analysis was performed to explore the cellular components in bone metastasis. We selected bone marrow samples from intracardiac-injected mice in the *Arid1a*^NC^ and *Arid1a*^KO^ groups (Fig. 4A). First, based on the expression of canonical marker genes in different clusters shown in the dot plot, we identified and visualized 10 major clusters using t-distributed stochastic neighbor embedding (t-SNE) (Fig. 4B, C). We found that the count of granulocytic myeloid cells in the *Arid1a*^KO^ group was significantly elevated compared to that in the *Arid1a*^NC^ group (37.11% vs 40.03%, *p* < 0.001; Fisher’s exact test. Fig. 4D), while the other types of cells showed no difference. Feature plots were generated to depict characteristic markers^36^, with expression levels color-coded from low (gray) to high (orange) (Fig. 4E). To further investigate which specific cluster within the granulocytic myeloid cells showed increased abundance in the *Arid1a*^KO^ group, we constructed a pseudotime trajectory. The trajectory was rooted in early myeloid cells, defined as neutrophil progenitors expressing high levels of progenitor markers such as *Elane*, *Mpo*, and *Prtn3* (Supplementary Fig. 6A). The Pre-MDSC represents a transitional cell type within the granulocytic transcriptional continuum, marked by high expression of *Asprv1, Pirb* and *Retnlg* (Supplementary Fig. 6B). PMN-MDSC were characterized as a functional immunosuppressive state acquired by neutrophils under specific tumor microenvironmental pressures, defined by high expression of *Cd84*, *Arg2*, *Il1b*, and *Spi1* (Supplementary Fig. 6C). We found that PMN-MDSCs are mainly derived from the conversion of neutrophils and the development of early myeloid cells within granulocytic myeloid clusters (Fig. 4F). Among these, state 3 exhibited the highest proportion of PMN-MDSCs, with the majority of cells in this state originating from the *Arid1a*^KO^ group. This finding suggested that the bone marrow immune microenvironment in the *Arid1a*^KO^ group harbored a higher abundance of PMN-MDSCs compared to the negative control group (Fig. 4G). To further validate this finding, we performed an independent analysis using Single-Cell Velocity (scVelo), a method that infers directionality in cellular state transitions. ScVelo consistently reconstructed the same developmental trajectory (Supplementary Fig. 6D). Further comparative analysis of immune cell proportions revealed that PMN-MDSCs were significantly more abundant in the *Arid1a*^KO^ group than in the negative control group. Notably, among all immune cell types analyzed, only PMN-MDSC proportion showed a significant increase in the *Arid1a*^KO^ group (Fig. 4H, I). Remarkably, there was no significant difference in the proportion of macrophages between the two groups (Fig. 4J). Numerous prior studies have demonstrated that PMN-MDSCs exhibit substantial immunosuppressive capabilities^25,37,38^. Thus, we focused on PMN-MDSCs to investigate their functional roles in bone metastasis promoted by *ARID1A* deficiency.

### *ARID1A* deficiency drives the expansion of PMN-MDSCs

**Fig. 5:**
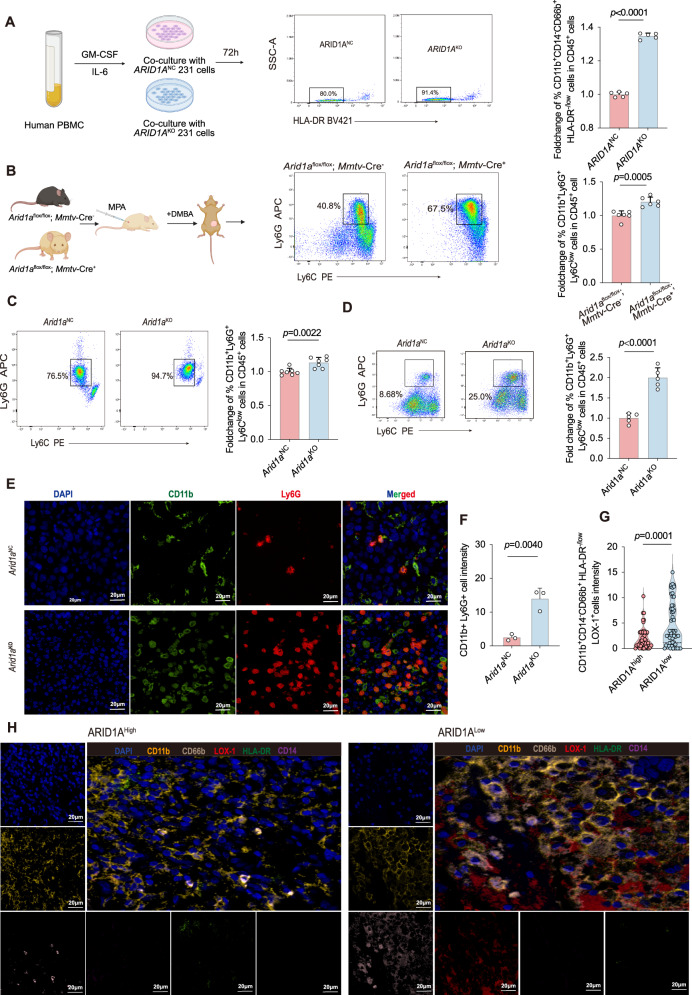
ARID1A deficiency drives the expansion of PMN-MDSCs. **A** Proportions of human PMN-MDSCs after in vitro co-culture with *ARID1A*^NC^ and *ARID1A*^KO^ MDA-MB-231 cells. Created in BioRender. Pan, S. (https://BioRender.com/f67lsrb). Data are presented as mean ± SD (*n* = 5 donors per group), unpaired two-tailed Student’s t test was used (*p* = 7.0 × 10^−10^). **B** Proportions of PMN-MDSCs in the bone marrow of *Arid1a*^flox/flox^;*Mmtv*-Cre^+^ mice compared to *Arid1a*^flox/flox^;*Mmtv*-Cre^-^ control mice. Data are presented as mean ± SD (*n* = 6 mice per group), unpaired two-tailed Student’s t test was used. Created in BioRender. Pan, S. (2https://BioRender.com/ksgts2j). **C** Bone marrow was harvested from Balb/c mice following intracardiac injection of *Arid1a*^NC^ and *Arid1a*^KO^ 4T-1 cells, and analyzed by flow cytometry to quantify PMN-MDSC proportions. Data are presented as mean ± SD (*n* = 7 mice per group), unpaired two-tailed Student’s t test was used. **D** PMN-MDSC proportions in bone metastatic tumors from Balb/c mice following intratibial injection of *Arid1a*^NC^ and *Arid1a*^KO^ 4T-1 cells. Data are presented as mean ± SD (*n* = 5 mice per group), unpaired two-tailed Student’s t test was used (*p* = 4.3 × 10^−5^). **E**,** F** The percentage of CD11b + Ly6G+ cells in bone metastatic tumors from Balb/c mice following intratibial injection of *Arid1a*^NC^ and *Arid1a*^KO^ 4T-1 cells was quantified by immunofluorescence (IF) staining, with quantitative analysis between experimental groups. Data are presented as mean ± SD (*n* = 3 mice per group), unpaired two-tailed Student’s t test was used. Scale bar, 20μm. **G, H** The proportion of PMN-MDSCs in tumor tissues from TNBC patients with high versus low ARID1A expression was assessed by TSA staining on tissue microarrays, followed by quantitative comparison. Total *n* = 108 patients, unpaired two-tailed Student’s t test was used. Scale bar, 20μm. All *p* values are indicated in the figures. Source data are provided as a Source Data file.

To verify *ARID1A* deficiency promotes the expansion of PMN-MDSCs, we isolated peripheral blood mononuclear cells (PBMCs) from human peripheral blood and co-cultured with *ARID1A*^NC^ and *ARID1A*^KO^ MDA-MB-231 cells for 72 hours^36^. We found that the proportion of PMN-MDSCs was significantly higher in the *ARID1A*^KO^ group compared to the *ARID1A*^NC^ group (Fig. 5A). Furthermore, primary cells isolated from mouse bone marrow co-cultured with *Arid1a*^NC^ and *Arid1a*^KO^ 4T-1 cells also showed a significantly higher proportion of PMN-MDSCs in the *Arid1a*^KO^ group (Supplementary Fig. 7A). The gating strategy for flow cytometry was shown in Supplementary Fig. 8.

We generated *Arid1a*^flox/flox^;*Mmtv*-Cre^+^ mice, a well-established model in which the *Arid1a* gene was specifically ablated in the mammary gland. To trigger the spontaneous development of mammary tumors, both *Arid1a*^flox/flox^;*Mmtv*-Cre^-^ control mice and *Arid1a*^flox/flox^;*Mmtv*-Cre^+^ mice were subcutaneously injected with medroxyprogesterone acetate (MPA) and orally administered 7,12-dimethylbenz[a]anthracene (DMBA) via gavage^39^. Analysis of flow cytometry revealed a higher proportion of PMN-MDSCs in the bone marrow of mammary-specific knockout of *Arid1a* mice compared to *Arid1a*^flox/flox^;*Mmtv*-Cre^-^ control mice (Fig. 5B). IHC of mammary tumors from *Arid1a*^flox/flox^;*Mmtv*-Cre^+^ mice revealed loss of Arid1a protein expression (Supplementary Fig. 9A, B).

We replicated the intracardiac injection model and cytometry analysis reaffirmed a markedly elevated proportion of PMN-MDSCs in the bone marrow of *Arid1a*^KO^ group when compared to the *Arid1a*^NC^ group (Fig. 5C). Additionally, we collected bone metastatic tumors from intratibial injection model and subjected to both flow cytometry and immunofluorescence (IF) analyses. Both approaches consistently demonstrated a significantly higher proportion of PMN-MDSCs in the bone metastatic tumors of *Arid1a*^KO^ group compared to the *Arid1a*^NC^ group (Fig. 5D–F). Furthermore, to assess the clinical relevance of *ARID1A* and PMN-MDSCs in breast cancer, we analyzed a set of tissue microarray comprising samples from 108 TNBC patients. Multiplex immunofluorescence staining for human PMN-MDSC markers revealed that tumors with low ARID1A expression exhibited a significantly higher density of PMN-MDSCs compared to those with high ARID1A expression (Fig. 5G, H). These results further confirm that ARID1A deficiency drives PMN-MDSC expansion.

### Ornithine and spermine secreted by cancer cells increased the expansion of PMN-MDSCs

Given the increased number of PMN-MDSCs in the *Arid1a*^KO^ group revealed by scRNA-seq, we sought to investigate the mechanism through which *ARID1A* deficiency promotes PMN-MDSC expansion. We found that PMN-MDSCs exhibited a significant enrichment in amine and polyamine metabolism pathways compared to other cell types, indicating a metabolic reprogramming that renders them particularly dependent on this pathway to fuel their rapid expansion and functional activity (Supplementary Fig. 10A). Accordingly, to delineate the drivers of this reprogramming, we further analyzed differentially expressed genes in PMN-MDSCs from *Arid1a*^NC^ and *Arid1a*^KO^ groups. We found that *Arg2*, *Malat1*, and spermidine/spermine N1-acetyltransferase 1 (*Sat1)* showed the most pronounced differential expression in PMN-MDSCs between the *Arid1a*^NC^ and *Arid1a*^KO^ group (Supplementary Fig. 10B). We isolated primary cells from mouse bone marrow and PBMCs from human peripheral blood, followed by sorting of PMN–MDSCs^40^. RT-qPCR was employed to validate the expression. The results revealed that *Arg2* and *Sat1* were markedly upregulated in PMN-MDSCs co-cultured with *Arid1a*^KO^ cells (Supplementary Fig. 10C–E). We examined the expression of *Arg2* and *Sat1* over pseudotime in different states and discovered that cells in state 3 exhibited significantly higher *Arg2* and *Sat1* expression than those in state 4. Critically, state 3 contained the highest proportion of PMN-MDSCs, with the majority of cells in this state derived from the *Arid1a*^KO^ group—findings consistent with our prior validation (Supplementary Fig. 10F). Given that *Arg2* and *Sat1* are key regulators of arginine metabolism, we hypothesized that PMN-MDSC expansion in bone marrow was driven by cancer cell-secreted ornithine and spermine.

**Fig. 6:**
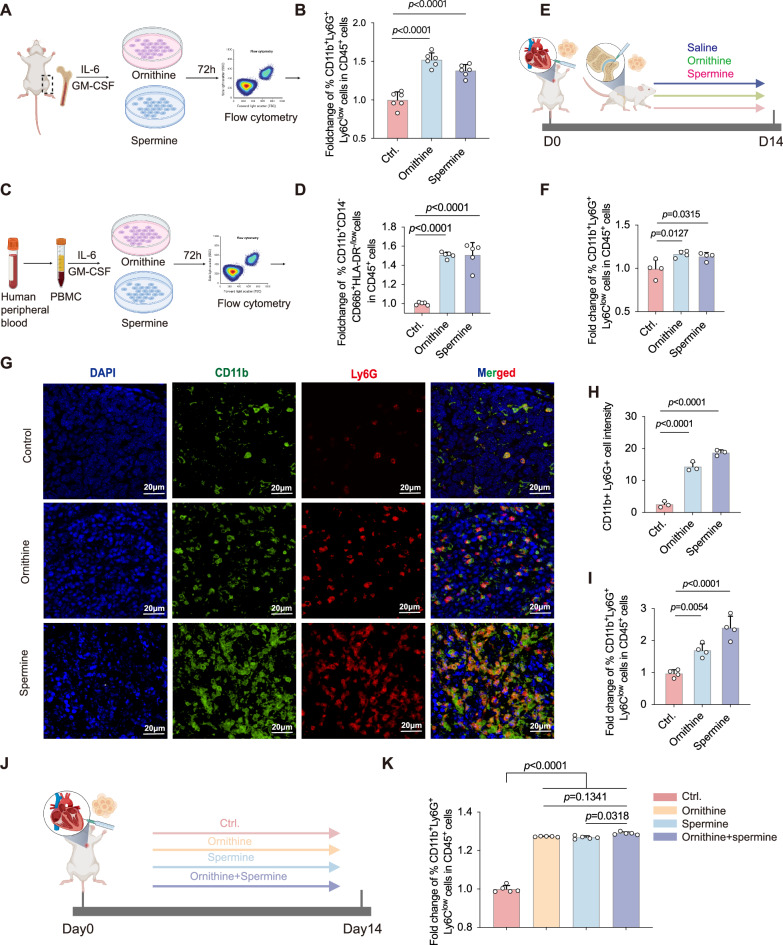
Ornithine and spermine secreted by cancer cells expand PMN-MDSCs. **A** Schematic illustration of the experimental design for primary mouse bone marrow cells co-cultured with metabolites to induce PMN-MDSCs. Created in BioRender. Pan, S. (https://BioRender.com/v0galhk). **B** Proportions of mouse PMN-MDSCs induced following in vitro treatment with ornithine (100 μM) and spermine (20 μM). Data are presented as mean ± SD (*n* = 6 mice), one-way ANOVA for multiple comparisons. Exact *p*‑values are as follows: *p* = 1.3 × 10^−7^ (Ctrl. vs. Ornithine); *p* = 6.7 × 10^−6^ (Ctrl. vs. Spermine). **C** Schematic illustration of the experimental design for human PBMC cells co-cultured with metabolites to induce PMN-MDSCs. Created in BioRender. Pan, S. (https://BioRender.com/0zfhnft). **D** Proportions of human PMN-MDSCs induced following in vitro treatment with ornithine (100 μM) and spermine (20 μM). Data are presented as mean ± SD (*n* = 5 donors), one-way ANOVA for multiple comparisons. Exact *p*‑values are as follows: *p* = 4.8 × 10^−7^ (Ctrl. vs. Ornithine); *p* = 4.7 × 10^−7^ (Ctrl. vs. Spermine). **E** Mouse models with exogenous ornithine and spermine supplementation. Created in BioRender. Pan, S. (https://BioRender.com/9tbd1la). **F** PMN-MDSC proportions in bone marrow of the intracardiac injection model. Data are presented as mean ± SD (*n* = 4 mice), one-way ANOVA for multiple comparisons. **G**,** H** The percentages of CD11b + Ly6G+ cells in bone metastatic tumors were quantified by immunofluorescence (IF) staining in the intratibial injection model. Data are presented as mean ± SD (*n* = 3 mice), one-way ANOVA for multiple comparisons. Exact *p*‑values are as follows: *p* = 2.5 × 10^−5^ (Ctrl. vs. Ornithine); *p* = 3.9 × 10^−6^ (Ctrl. vs. Spermine). Scale bar, 20μm. (**I**) PMN-MDSC proportions in bone metastatic tumors from mice following intratibial injection. Data are presented as mean ± SD (*n* = 4 mice), one-way ANOVA for multiple comparisons. Exact *p*‑values are as follows: *p* = 4.3×10^−5^ (Ctrl. vs. Spermine). **J** Mouse models were established by intracardiac injection of wild-type 4T-1 cells, and then subjected to exogenous supplementation with ornithine, spermine, or their combination. Created in BioRender. Pan, S. (https://BioRender.com/y6t9qqp). **K** PMN-MDSC proportions in bone marrow of each group. Data are presented as mean ± SD (*n* = 5 mice), two-way ANOVA for multiple comparisons. The interaction between the two factors was significant (*p* < 0.0001), other *p* values are indicated in the figures. Source data are provided as a Source Data file.

We next focused on exploring the effect of ornithine and spermine in promoting the expansion of PMN-MDSCs. Figure 6A showed that primary cells isolated from mouse bone marrow were co-cultured with ornithine and spermine. The results demonstrated that both ornithine and spermine promoted PMN-MDSC expansion in vitro (Fig. 6B). To ascertain whether myeloid cells in humans exhibit analogous regulatory programs, we co-cultured human PBMCs with ornithine and spermine for 72 hours, confirming that ornithine and spermine enhanced human PMN-MDSC expansion (Fig. 6C, D). To further determine whether ornithine and spermine independently promote PMN-MDSC expansion, we established bone metastasis models via intracardial and intratibial injection of wild-type 4T-1 cells, followed by daily intraperitoneal injection of saline, ornithine, or spermine (Fig. 6E). In the intracardial injection model, mice receiving ornithine or spermine showed a significant increase in PMN-MDSCs in bone marrow compared with the control group (Fig. 6F). Additionally, we collected bone metastases from intratibial injection model and subjected to both flow cytometry and IF analyses, which further confirmed the increased accumulation of PMN-MDSCs in the ornithine and spermine-treated groups (Fig. 6G-I). To further explore the combined effect of ornithine and spermine on promoting PMN-MDSCs, we utilized an intracardiac injection model (Fig. 6J). Although a significant interaction was observed between ornithine and spermine (*p* < 0.0001). However, we found that their combined administration induced only a modest increase in bone marrow PMN‑MDSCs compared to spermine alone, while remaining comparable to ornithine alone. (Fig. 6K). Sorted human and mouse PMN-MDSCs were co-cultured with ornithine or spermine, respectively. Significant upregulation of *ARG2* and *SAT1* expression was observed in both treatment group (Supplementary Fig. 10G–N). These results indicate that *ARID1A*^KO^ TNBC cells promote the expansion of PMN-MDSCs by producing increased levels of ornithine and spermine, with PMN-MDSCs of the *ARID1A*^KO^ group exhibiting higher expression of *ARG2* and *SAT1*.

### PMN-MDSCs play a critical role in *ARID1A* deficiency-mediated bone metastasis

To further validate the critical role of PMN-MDSCs in *ARID1A* deficiency-mediated bone metastasis, we examined whether the proliferative capacity differed between *ARID1A*^NC^ and *ARID1A*^KO^ cells. We found that *ARID1A*^NC^ and *ARID1A*^KO^ cells exhibited comparable proliferative capacities in vitro (Supplementary Fig. 11A, B). Given that the intrinsic growth of tumor cells was unaffected, we administered anti-Ly6G antibody to delete PMN-MDSCs in mice^41^. In the intracardiac injection model established with *Arid1a*^NC^ or *Arid1a*^KO^ cells, anti-Ly6G antibody treatment significantly reduced bone metastatic incidence in *Arid1a*^KO^ group (Supplementary Fig. 11C–E). These findings demonstrate PMN-MDSC as a key mediator of bone metastasis in *ARID1A*-deficient TNBC.

### ARID1A deficiency relies on residual SWI/SNF complex to promote bone metastasis

To evaluate whether *ARID1A* deficiency promotes bone metastasis through residual SWI/SNF activity, we employed a bone metastasis model using the BRM014, a catalytic inhibitor of SWI/SNF ATPase activity. We found that BRM014 treatment did not significantly alter bone metastasis in *Arid1a*^NC^ control mice. In contrast, BRM014 suppressed bone metastasis in *Arid1a*^KO^ mice (Supplementary Fig. 12A, B). Similarly, BRM014 failed to alter the concentration of PMN-MDSCs in the bone marrow of *Arid1a*^NC^ mice but significantly reduced their elevated levels in *Arid1a*^KO^ mice (Supplementary Fig. 12C). Collectively, these data indicate that bone metastasis driven by *ARID1A* deficiency is dependent on the residual SWI/SNF complex; notably, standalone SWI/SNF inhibition cannot mimic the phenotypic alterations caused by *ARID1A* deficiency, which implicates additional *ARID1A*-specific functional roles in mediating tumor progression. However, targeting the SWI/SNF complex poses a significant challenge due to concerns of on-target toxicity, stemming from its ubiquitous expression and indispensable functions in normal cells, we pivoted to a more promising therapeutic strategy centered on targeting the arginine signaling pathway.

### Targeting arginine signaling pathway prevents *ARID1A*-deficient TNBC bone metastasis

**Fig. 7:**
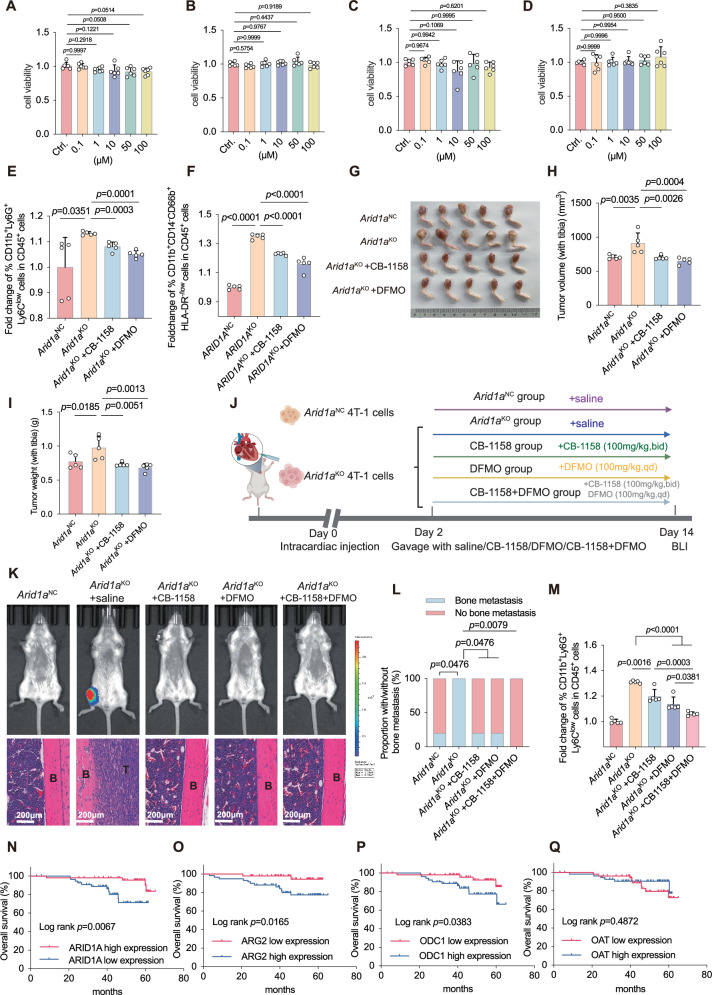
ARG2 and ODC1 inhibitors suppress ARID1A-deficient TNBC bone metastasis. **A**,** B** CCK-8 assays of different concentrations of CB-1158 (**A**) and DFMO (**B**) on 4T-1 cells for 48 h. **C**,** D** CCK-8 assays of different concentrations of CB-1158 (**C**) and DFMO (**D**) on MDA-MB-231 cells for 48 h. Data are presented as mean ± SD (*n *= 6 biological replicates), one-way ANOVA for multiple comparisons (A-D). **E** Mouse PMN-MDSCs proportions after co-culture with *Arid1a*^NC^, *Arid1a*^KO^, *Arid1a*^KO^ plus CB-1158, or *Arid1a*^KO^ plus DFMO 4T-1 cells. Data are presented as mean ± SD (*n* = 5 mice), one-way ANOVA for multiple comparisons. **F** Human PMN-MDSCs proportions after co-culture with *ARID1A*^NC^, *ARID1A*^KO^, *ARID1A*^KO^ plus CB-1158, or *ARID1A*^KO^ plus DFMO MDA-MB-231 cells. Data are presented as mean ± SD (*n* = 5 donors), one-way ANOVA for multiple comparisons. *ARID1A*^NC^ vs. *ARID1A*^KO^, *p* = 1.1 × 10^−12^; *ARID1A*^KO^ vs. *ARID1A*^KO^ plus CB-1158, *p* = 5.9 × 10^−6^; *ARID1A*^KO^ vs. *ARID1A*^KO^ plus DFMO, *p* = 1.1×10^−8^. **G–I** Image of intratibial tumors in mice injected with *Arid1a*^NC^ 4T-1 cells, *Arid1a*^KO^ 4T-1 cells, as well as those treated with CB-1158 and DFMO (**G**), tumor volume (**H**), and tumor weight (**I**). Data are presented as mean ± SD (*n* = 5 mice), one-way ANOVA for multiple comparisons. **J** Schematic diagram illustrating the experimental design, including drug administration schedules. bid: twice daily; qd: once daily. Created in BioRender. Pan, S. (https://BioRender.com/qpmw6rz). **K** Bioluminescence imaging (BLI) and H&E staining images of mice in each group with intracardiac injection. Scale bar, 200μm. B bone, T tumor. **L** Statistical charts presenting the bone metastasis incidence in each group with intracardiac injection. *n *= 5 mice per group, two-sided Fisher’s exact test was used. **M** Bone marrow of mice was harvested from each group with intracardiac injection, and analyzed by flow cytometry to quantify PMN-MDSC proportions. Data are presented as mean ± SD (*n* = 5 mice), statistical analysis by two‑way ANOVA showed no significant interaction between treatments (*p* = 0.3035), other *p* values are indicated in the figures. **N–Q** Kaplan-Meier survival curves for 108 TNBC patients with low and high level of ARID1A, ARG2, ODC1 and OAT. The log-rank test was employed to calculate the difference between the two groups. Source data are provided as a Source Data file.

Given the pivotal role of the arginine signaling pathway in driving PMN-MDSC expansion and consequent bone metastasis in *ARID1A*-deficient breast cancer, we investigated its therapeutic potential in controlling metastatic progression. ARG2 and ODC1 are key rate-limiting enzymes in the arginine metabolic pathway, which play critical roles in regulating ornithine and spermine production^42,43^. CB-1158, a selective ARG2 inhibitor blocking arginine from being converted to ornithine, and DFMO (2-difluoromethylornithine), an ODC1 inhibitor, are frequently utilized to impede the biosynthesis of spermine^44,45^. Previous studies have demonstrated that blocking key enzymes of the arginine metabolic pathway leads to a reduction in ornithine and its downstream metabolites^46^. We employed CB-1158 and DFMO to investigate their inhibitory effects on bone metastasis induced by *ARID1A* deficiency. First, we assessed whether CB-1158 and DFMO exert cytotoxic effects on tumor cells and found that neither CB-1158 nor DFMO exhibited significant direct cytotoxicity against TNBC cells (Fig. 7A–D). Then we focused on their potential immunomodulatory roles in the bone metastatic niche and found that CB-1158 and DFMO exhibited a remarkable capacity to curtail the proportions of PMN-MDSCs in both human and murine in vitro systems (Fig. 7E, F). Further intracardiac inoculation of *Arid1a*^KO^ 4T-1 cells significantly increased PMN-MDSC accumulation in murine bone marrow compared to controls, whereas combined treatment with CB-1158 or DFMO effectively reduced PMN-MDSC proportions (Supplementary Fig. 13A). Furthermore, the increased incidence of bone metastasis triggered by *Arid1a* knockout was significantly mitigated by administering inhibitors targeting ARG2 and ODC1 (Supplementary Fig. 13B, C). In the intratibial bone metastasis model, CB-1158 and DFMO treatment significantly attenuated intratibial tumor progression [tumor volume: NC, 706.10 ± 29.72 mm^3^; KO, 901.68 ± 152.39 mm^3^; CB-1158, 699.93 ± 26.34 mm^3^; DFMO, 651.29 ± 47.78 mm^3^; tumor weight: NC, 0.77 ± 0.09 g; KO, 0.97 ± 0.17 g; CB-1158, 0.73 ± 0.03 g; DFMO, 0.69 ± 0.05 g; Fig. 7 G-I]. We next assessed the potential interaction between CB-1158 and DFMO (Fig. 7J). Their combination demonstrated greater efficacy in reducing bone metastasis incidence (Fig. 7K, L). For the reduction of PMN-MDSCs, although no significant interaction was detected (*p* = 0.3035), the combination was more effective than either agent alone, suggesting an additive rather than a synergistic effect (Fig. 7M). Collectively, these data indicate that the inhibition of ARG2 or ODC1, especially that of their combination, resulted in a significant reduction in the proportion of PMN-MDSCs. This, in turn, substantially impaired the progression of bone metastasis, highlighting the pivotal role of these molecular interventions in disrupting the metastatic cascade within the bone microenvironment.

Given the critical role of arginine metabolism in TNBC bone metastasis, we further investigated its clinical relevance. Analysis revealed that low ARID1A expression and upregulation of ARG2 and ODC1 correlated with poor prognosis in TNBC patients, underscoring the therapeutic potential of targeting this pathway (Fig. 7N–Q).

### Systemic assessment of CB-1158 and DFMO treatment in mice

To evaluate the clinical relevance of the inhibitors, we conducted a comprehensive safety assessment in mice treated with CB-1158, DFMO and their combination. The results demonstrated that neither CB-1158, DFMO nor their combination induced any significant adverse effects on liver or kidney function, as indicated by biochemical analyses of parameters such as alanine transaminase (ALT), aspartate transaminase (AST), alkaline phosphatase (ALP), urea (UREA), uric acid (UA), and creatinine (CREA) (Supplementary Fig. 14A, B). Additionally, complete blood count (CBC) parameters, including white blood cell count (WBC), red blood cell count (RBC), lymphocyte count, platelet count (PLT), hematocrit (HCT), hemoglobin (HGB), mean corpuscular hemoglobin (MCH), and red cell distribution width (RDW), remained within normal ranges, suggesting no hematological toxicity associated with the inhibitors (Supplementary Fig. 14C). Furthermore, histopathological examination of the heart, liver, spleen, lung, and kidney revealed no evidence of tissue damage (Supplementary Fig. 14D). These results indicate that CB-1158, DFMO and their combination have high safety profiles, thereby presenting promising opportunities for the development of targeted clinical therapies.

## Discussion

**Fig. 8:**
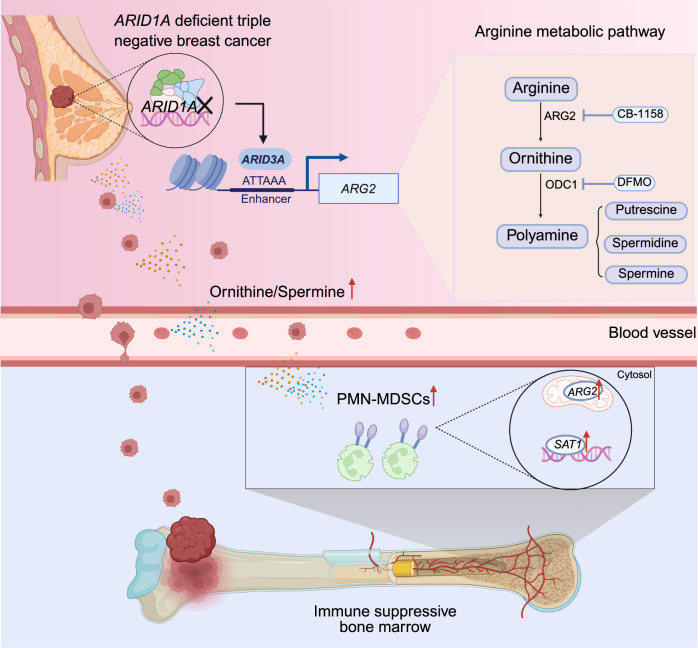
Schematic representation of the model by which ARID1A deficiency promotes TNBC bone metastasis. In our study, *ARID1A*-deficient TNBC through *ARID3A*-mediated transcriptional activation of *ARG2* upregulates the arginine metabolic pathway, increasing ornithine and spermine levels, which augment the abundance of PMN-MDSCs in the bone marrow, fostering an immunosuppressive bone marrow microenvironment. Ultimately, this series of events promote bone metastasis in *ARID1A*-deficient TNBC. The inhibition of ARG2 and ODC1 effectively curtails the bone metastasis triggered by *ARID1A* deficiency. Created in BioRender. Pan, S. (https://BioRender.com/tq7lrfa).

Metabolic crosstalk between cancer cells and immune cells within the bone marrow plays a critical role in bone metastasis. In this study, we demonstrate that *ARID1A* deficiency upregulates the arginine metabolic pathway, leading to increased levels of ornithine and spermine. Furthermore, we reveal that metabolic communication between cancer cells and PMN-MDSCs in bone marrow serves as a key driver of bone metastasis in *ARID1A*-deficient TNBC (Fig. 8). These findings highlight the necessity to integrate intercellular metabolic crosstalk within the TME during cancer metastasis.

TNBC, characterized by its high metastatic propensity and limited treatment options, represents the most aggressive breast cancer subtype^47^. Bone metastasis in breast cancer serves as a pivotal dissemination hub, where the supportive microenvironment enables circulating tumor cell survival, reactivation, and secondary organ spread, driving disease progression^3,29^. Consequently, bone metastasis in TNBC patients warrants urgent clinical attention and intervention. Our findings delineate a coherent molecular axis linking *ARID1A* deficiency to arginine metabolism-driven immunosuppression and bone metastasis. However, it is important to note that our clinical data indicate among patients without *ARID1A* mutations, 45.2% of them still developed bone metastasis, suggesting that the mechanism we describe represents a significant yet specific subset. The broader process is driven by diverse and complex synergistic mechanisms, including enhanced angiogenesis, adaptive immune resistance, among others.

We established models of bone metastasis in mice by performing intracardiac and intratibial injections with luciferase-expressing TNBC cells, and monitored tumor burden in vivo using BLI. It is important to acknowledge that these experimental models bypass the critical early stages of the metastatic cascade, including primary tumor development and spontaneous dissemination, thus limiting their ability to fully recapitulate the natural progression of breast cancer to bone. Future studies employing more physiologically relevant, spontaneous metastasis models will be necessary to better mimic the clinical course of the disease. Additionally, while the luciferase reporter is essential for in vivo imaging, its expression may introduce immunogenic effects that could influence tumor behavior and the host microenvironment^48^. Despite this potential constraint, luciferase-based imaging remains an effective method for longitudinally tracking metastatic progression in preclinical bone metastasis research^29,31,49^.

Arginine metabolism plays a vital role in cellular processes affecting both cancer and immune cells. In our study, loss of *ARID1A* facilitates the recruitment of the transcription factor ARID3A to the enhancer region of *ARG2*, promoting its transcription. The resulting accumulation of ornithine fuels its enzyme ODC1, thereby driving the arginine metabolic pathway. Evidence indicates that ornithine derived from tumor-associated macrophages (TAMs) mediates metabolic competition in the TME, depleting arginine to impair anti-tumor CD8 + T cell responses and facilitate immune evasion^50^. It has also been reported that there is a molecular pathway through which the polyamine metabolism derived from MDSCs promotes T helper 17 (Th17) differentiation and exacerbates systemic lupus erythematosus (SLE)^51^. However, the specific roles of tumor cell-secreted ornithine and individual polyamines in bone metastasis remain unclear. Our study demonstrates that ornithine and spermine produced by TNBC cells serve as the most potent drivers for stimulating the expansion of PMN-MDSCs, which are primarily derived from the bone marrow, thereby facilitating immunosuppression within the bone marrow^52,53^. The combined administration of ornithine and spermine significantly elevated PMN-MDSCs in the bone marrow, with only a modest enhancement compared to spermine alone. This suggests a partial functional overlap between the two metabolites, possibly approaching a saturation point in PMN-MDSC induction.

PMN-MDSCs, also known as pathologically activated neutrophils, are a critical component of the TME and play crucial roles in tumor progression and therapy resistance^54–56^. As defined by the phenotypic markers CD11b^+^Ly6G^+^Ly6C^low^, PMN-MDSCs are better understood as a functional immunosuppressive state acquired by neutrophils, under specific tumor microenvironmental pressures^53^. We revealed a critical immunosuppressive functional state of neutrophils within the *ARID1A* deficient bone metastatic niche which underscores the dynamic nature of myeloid cells in cancer. PMN-MDSCs inhibit the anti-tumor immune response by highly expressing immunosuppressive factors such as Interleukin-10 (IL-10), inducible Nitric Oxide Synthase (iNOS), and Arginase 1 (ARG1), thereby promoting the survival and metastasis of tumor cells^37^. In our study, ornithine and spermine mediated PMN-MDSCs to highly express *ARG2* and *SAT1*, which reflects a metabolic reprogramming that enables these cells to adopt a more specialized immune-suppressive phenotype and is worthy of further investigation. Previous studies have demonstrated that cancer cell-secreted arginine drives polyamine biosynthesis in TAMs, sustaining their pro-tumor polarization and inhibiting anti-tumor CD8 + T cell activity^50,57^. However, in our experimental paradigm, despite decreased arginine levels and increased downstream metabolites, macrophages exhibited no statistically significant alterations. This may be attributable to the complexity of metabolic networks, warranting further investigation in future studies.

There is currently no effective treatment for breast cancer patients with bone metastasis. Despite increasing recognition of cancer cell metabolism as a therapeutic target, few metabolic drugs have reached clinical use. Researchers are exploring metabolic pathways, such as energy production, amino acid metabolism, and lipid biosynthesis to disrupt metabolic adaptations critical for cancer survival and metastasis^58,59^. However, therapeutic options remain limited. This underscores the urgent need for approaches that can specifically target the unique metabolic alterations within the bone microenvironment^60,61^. While the residual SWI/SNF complex remains partially functional in *ARID1A*-deficient cells and targeting SWI/SNF directly poses significant challenges due to its broad expression and essential roles in normal cellular processes. Consequently, we therefore focused on downstream metabolic pathway. Our data demonstrated that inhibition of ARG2 and ODC1 significantly suppresses bone metastasis of *ARID1A*-deficient TNBC by reducing the generation of PMN-MDSCs in bone marrow. The additive effect in suppressing PMN-MDSCs by dual inhibition confirms the vulnerability of *ARID1A*-deficient bone metastases to arginine pathway blockade, highlighting a tangible clinical opportunity with several relevant agents already in development. Promising candidates include the arginase inhibitor CB-1158 and the ODC1 inhibitor DFMO. CB-1158 has shown acceptable safety and early signs of immune modulation in Phase I/II trials for solid tumors (NCT02903914; NCT03314935), while DFMO is being actively repurposed in oncology (NCT06059118; NCT06892678; NCT03536728). Consequently, pre-clinical and clinical studies could potentially serve to validate their therapeutic utility to reduce bone metastasis in TNBC patients with *ARID1A* mutations.

Taken together, our results elucidate a pivotal role of *ARID1A* deficiency activates arginine metabolism, resulting in ornithine and spermine accumulation that expands PMN-MDSCs and establishes an immunosuppressive bone microenvironment to drive TNBC bone metastasis. Given these insights, therapeutic targeting of the coordinated arginine metabolic signaling cascade to reduce PMN-MDSC accumulation in bone marrow represents a promising strategy to suppress bone metastasis in *ARID1A*-deficient TNBC.

## Methods

### Patients

A cohort of 663 female patients with breast cancer were recruited at the FUSCC. Informed consent was obtained for each patient involved. This study was approved and monitored by the Ethics Committee of FUSCC (1705172-9). Results of ctDNA test for all patients came from Burning Rock Biotech Co., Ltd (Guangzhou, China). We recorded all the metastatic sites and the baseline clinicopathologic characteristics of these patients, which were used to analyze the correlation between the mutated genes and the metastatic sites.

### Circulating DNA extraction

DNA isolation and subsequent sequencing procedures were performed in the laboratory of Burning Rock Biotech (Guangzhou, China) accredited and certified by the CAP and Clinical Laboratory Improvement Amendments (CLIA). Circulating cell-free DNA (cfDNA) were extracted using QIAamp Circulating Nucleic Acid Kits (Qiagen, Hilden, Germany) from 0.5–2.0 mL of the plasma samples. GenomicDNA (gDNA) used as normal control were extracted from white blood cells (WBCs) by QIAamp DNA Blood Mini Kit (Qiagen, Hilden, Germany). Qubit fluorometer with the dsDNA high-sensitivity assay kit (Life Technologies, Carlsbad, CA, USA) was used to measure DNA quality following the manufacturer’s instructions.

### Next-Generation Sequencing (NGS) library preparation, capture-based targeted DNA sequencing and sequence data analysis

NGS library was constructed for the DNA isolated from plasma and white blood cells according to optimized protocol, requiring a minimum of 50 ng of DNA. Target capture was performed using commercial panels consisting of 108 breast cancer-related genes (PurePlasma) and 520 cancer-related genes (OncoScreen Plus), spanning 0.249 megabases (Mb) and 1.64 Mb of the human genome, respectively (Burning Rock Biotech, Guangzhou,China). Indexed samples were sequenced on Nextseq500 sequencer (Illumina, Inc., US) with paired-end reads achieving target coverage of 10,000X for plasma samples. Sequencing data were analyzed using proprietary computational algorithms optimized for somatic variant calling. Variants with population frequency over 0.1% in the ExAC, 1000 Genomes, dbSNP or ESP6500SI-V2 databases were grouped as single nucleotide polymorphisms and excluded from further analysis. Variants detected from the patient’s own WBCs were filtered out to retain only the somatic variants. Only the variants with pathogenic/likely pathogenic classification based from ClinVar and other similar databases identified from the WBCs were flagged for reporting of incidental findings. Copy number variations (CNV) were analyzed based on the depth of coverage data of capture intervals. Coverage data were corrected for sequencing bias resulting from GC content and probe design. The average coverage of captured regions was used to normalize sample coverage, and CNV was calculated by comparing the patient’s coverage depth to that of at least 50 CNV-free reference samples per capture interval. CNVs were called when coverage data were quantitatively and statistically significant.

### Human tissue specimens

A set of TMA including 108 TNBC tissue samples was obtained from the recruited breast cancer patients, at their first radical surgery with no prior systemic neoadjuvant treatment at the FUSCC. The resected specimens were examined macroscopically to determine the location and size of the tumor. Specimens for histology were fixed in 10% formalin and processed for paraffin embedding. Overall survival (OS) was calculated from the date of surgery to the date of any-cause death or the last follow-up, which ever came first. The clinical baseline characteristics of 108 patients were shown in Supplementary Table 4.

### Cell lines and reagents

MDA-MB-231(HTB-26), BT-549(HTB-122) and 4T-1(CRL-2539) cells were obtained from the American Type Culture Collection (ATCC). MDA-MB-231 cells were grown in Dulbecco’s Modified Eagle Medium (DMEM, Gibco, USA) with 10% FBS and 1% penicillin-streptomycin. BT-549 and 4T-1 cells were cultured in Roswell Park Memorial Institute (RPMI)-1640 medium (Gibco, USA) supplemented with 10% FBS (Gibco, USA) and 1% penicillin-streptomycin. All cell lines were maintained at 37 °C in a humidified atmosphere of 5% CO_2_ incubator. All cell lines were free of mycoplasma and underwent DNA STR (short tandem repeat) genotyping for cell identification.

### Stable transfection using lentiviral infection

To generate *ARID1A*^KO^ cells, a lentiviral CRISPR/Cas9 system was employed. The Lenti-CAS9-Puro vector and the single-guide RNA (sgRNA) expression vector GV371-EGFP vector (Shanghai Genechem, Shanghai, China), carrying Cas9 and an *ARID1A*-targeting guide RNA respectively, were co-transfected with packaging plasmids into HEK293T cells using Lipofectamine 3000 (L3000-150, Invitrogen) to generate lentiviral particles. For the construction of *ARID1A*^OE^ cells, a CRISPR/Cas9 activation (CRISPRa) approach was employed using the synergistic activation mediator (SAM) system (Genechem). This system is based on a deactivated Cas9 (dCas9) platform. The SAM vector was programmed with a specific sgRNA targeting the promoter region of the *ARID1A* and the empty vector was used as negative control. The vector was similarly co-transfected with packaging plasmids into HEK293T cells for lentivirus production. For both KO and OE constructions, the virus-containing supernatant was collected 72 h after incubation, filtered and used to transduce cells in the presence of polybrene (5 μg/mL). To establish stable polyclonal populations, successfully transduced cells were selected with puromycin, with the antibiotic concentration optimized for the specific cell line. The primers used for plasmid construction were listed in Supplementary Table 7. The *ARID1A* knockout and overexpression efficiency was confirmed via western blotting.

### CCK-8 assay

The cell viability was examined using the CCK-8 cell proliferation assay. A total of 3000 cells per well were seeded and cultured in 100 µL of the media in 96-well microplates. For the drug intervention experiment, cells were seeded and treated with drugs (CB-1158, MCE, HY-101979; DFMO, Selleck Cat# S4582) at various concentrations for 48 h. Absorbance at 450 nm was measured using a microplate reader (BioRad, USA) 2 h after adding 10 µL CCK-8 reagent to each well.

### Wound healing assay

*ARID1A*^NC^ and *ARID1A*^KO^ MDA-MB-231 cells were seeded in six-well plates and grown to confluence. A sterile pipette tip was used to create a linear wound across the cell monolayer. The wound area was gently washed to remove detached cells and debris, followed by the addition of fresh medium containing the respective treatments (sterile water, ornithine, and spermine). Wound closure was monitored 48 hours later using phase-contrast microscopy.

### Colony formation assay

For colony formation assay, cells were seeded in six-well plates (1000 cells per well) and cultured with medium containing sterile water, ornithine or spermine for 10-14 days. Colonies were fixed and stained with 0.2% crystal violet.

### Mice

For isograft mouse model, female Balb/c mice (6-8-week-old) were utilized to establish bone metastasis model. Intracardiac injection and intratibial injection were performed as previously described^62,63^. In the intracardiac and intra-tibial injection models, 2 × 10⁵ luciferase-labeled 4T-1 cells were injected into the left ventricle and the bone marrow cavity of the tibia, respectively. For the intracardiac model, in vivo bioluminescence imaging was performed to confirm the development of bone metastases. At the experimental endpoint (day 14), mice were euthanized. Bone metastases were further verified by H&E staining of the tibiae. For intratibial injection model, tumor growth was monitored by quantifying bioluminescence intensity. On day 14, mice were euthanized, and tumor burden was assessed by in vivo bioluminescence intensity, tumor weight, and volume. For xenograft mouse model, we used 6-8-week-old female NSG mice that has been humanized with human CD34^+^ hematopoietic stem cells. Luciferase-labeled MDA-MB-231 cells (2 × 10⁵) were injected into the left ventricle, and bone metastasis was monitored via BLI. On day 14, mice were euthanized and bone metastases were further verified by H&E staining of the tibiae. All experiments were designed and performed in compliance with the NIH Guide for the Care and Use of Laboratory Animals and the Guide of the Animal Care Committee of Fudan University.

*Arid1a*^flox/flox^;*Mmtv*-Cre mice were purchased from Shanghai Model Organisms Center, Inc (Shanghai, China). Female mice at 6–8 weeks of age were used to initiate the experiments. The mice were intraperitoneally injected with MPA (Selleck Chemicals, Texas, United States) combined with an oral carcinogen, DMBA (TCI, D0677). Mice were administered a dose of 1.0 mg of DMBA in 0.2 ml of corn oil until tumor developed. Mice were then euthanized when the primary tumor reached a predefined humane endpoint size of 2 cm in diameter to ensure comparable tumor burden at analysis and bone marrow was collected for flow cytometric analysis.

Female Balb/c mice (6–8 weeks old) were used for PMN-MDSC depletion, after tumor cell inoculation, mice were received intraperitoneal injections of anti-Ly6G antibody (10 mg/kg, BioXCell, BE0075-1) or the mouse control IgG (cIgG) (10 mg/kg, Sigma, I8765) every 3 d. Female Balb/c mice (6–8 weeks old) were used for the BRM014 treatment group. After tumor cell inoculation, mice were intraperitoneal injections of BRM014 (7.5 mg/kg, once daily; MCE, HY-119374). Metastasis was confirmed by in vivo bioluminescence imaging. On day 14, mice were euthanized and bone metastases were verified by H&E staining of the tibiae.

Female Balb/c mice (6–8 weeks old) were used for the CB-1158, DFMO and their combination treatment groups. After tumor cell inoculation, mice were gavaged with CB-1158 (100 mg/kg, twice daily; MCE, HY-101979), DFMO (100 mg/kg, once daily; Selleck Cat# S4582) and their combination, respectively.

All animals were maintained in a specific pathogen-free (SPF) facility with a temperature between 21 ± 2 °C, humidity of 45 to 65%, and a regulated 12-hour light/dark cycle. The animals were provided with normal diet food and water ad libitum (Jiangsu Xietong Pharmaceutical Bio-engineering Co., Ltd., catalog number 1010088). All animals were female, as this study focused on breast cancer, which predominantly affects females. The animal experiments were approved by the Fudan University Shanghai Cancer Center Institutional Review Board and strictly carried out in accordance with the People’s Republic of China Legislation Regarding the Use and Care of Laboratory Animals (FUSCC-IACUC-2023673). The experimental endpoint was determined either when the scheduled experimental day was reached, or when the tumor reached the maximal allowed size (2 cm in diameter), or upon the appearance of predefined humane endpoints, which included severe weight loss ( > 20% of initial body weight), impaired mobility, signs of pain or distress, or inability to access food or water. No animals exceeded the maximal tumor size or humane endpoints prior to the scheduled experimental endpoint. All mice were euthanized by carbon dioxide inhalation.

### Bioluminescence imaging

In vivo bioluminescence imaging was performed with IVIS Spectrum (PerkinElmer) to monitor the bone metastatic colonization of breast tumor cells. Mice were anesthetized and imaged immediately after intraperitoneal injection of 150 μL of d-luciferin (MCE, HY-12591A). To quantify the metastatic burden, the total flux was calculated over the same region of interest using Living Image software (PerkinElmer).

### Tissue collection and cell isolation

#### Bone marrow

After mouse dissection, bone marrow was flushed from mouse tibia and femurs using a needle. Bone marrow cells were filtered through a 70 μm filter and centrifuged at 600 g at 4 °C for 7 min. Cells were incubated for 5 min at room temperature (RT) in 2 ml of red blood cell lysis buffer (Solarbio, R1010). The lysis reaction was terminated and centrifuged again, after which the living cells were counted using the automated cell counter.

### Tumor

Tumors were surgically excised from the tibia and washed in PBS. Necrotic portions of the tumor were carefully excised. The viable tumor tissues were then minced into small fragments and dissociated in DMEM with collagenase type IV (2 mg/ml, Sigma­ Aldrich), deoxyribonuclease (0.1 mg/ml, Sigma-Aldrich), hyaluronidase (0.1 mg/ml, Sigma­Aldrich), and bovine serum albumin (BSA) (1 mg/ml, Sigma-Aldrich) at 37 °C for 2 hours to facilitate enzymatic digestion. The resulting cell suspension was passed through a 70 µm cell strainer to remove undigested tissue fragments. The filtrate was then centrifuged at 500 g for 7 minutes to pellet the cells. The supernatant was discarded, and the cell pellet was resuspended in fresh culture medium for further analysis or experimental use.

### Single-cell RNA sequencing

Cell count and viability was estimated using fluorescence Cell Analyzer (Countstar^®^ Rigel S2) with AO/PI reagent after removal erythrocytes (Solarbio R1010) and then debris and dead cells removal was decided to be performed or not (Miltenyi 130-109-398/130-090-101). ScRNA-seq libraries were prepared using Chromium Next GEM Single Cell 3ʹ Reagent Kits v3.1 (10x Genomics Catalog No.1000268) then sequenced on illumina NovaSeq 6000 with PE150 read length or DNBSEQ-T7 platform with PE150 read length (SeekGene, China).

### Mouse MDSCs induction

Bone-marrow cells were obtained from 6-week-old female Balb/c mice and treated with GM-CSF (50 ng/ml) (Gibco, 315-03-50UG) and IL-6 (50 ng/ml) (Gibco, 216-16-50UG) after removing erythrocytes. After co-culturing with breast cancer cells (4T-1 cells) and metabolites (ornithine or spermine) for 72 h, cells were harvested and labeled for flow cytometry with antibodies.

### Human MDSCs induction

We followed established protocols for in vitro generation of human MDSCs^64^. Peripheral blood from healthy adult females was incubated with 3% dextran for 18 min, and supernatants were collected and followed by differential density gradient separation. Samples were centrifuged at 500 relative centrifugal force for 30 min at 20 °C. PBMCs including granulocytes were collected and incubated with GM-CSF (20 ng/ml) (Gibco, 300-03-20UG) and IL-6 (20 ng/ml) (Gibco, 200-06-20UG) for 72 h.

### Flow cytometry and fluorescence-activated cell sorting (FACS)

Single-cell suspensions from mouse bone marrow and tumor were counted and washed in DPBS (Gibco) and blocked with FcR (anti-mouse CD16/32 antibody, BD Pharmingen, 553141). Cells from human PBMC were washed in DPBS and blocked with FcR (BD Pharmingen, 564220). Then, cells were incubated with Fixable Viability Stain 510 (BD Pharmingen, 564406) for dead cell exclusion. Cells were washed in DPBS and then stained with cell surface markers. The single cell suspensions of mouse were stained with the following antibodies: Allophycocyanin-Cyanine 7 (APC-CY7) anti-mouse CD45 (1:200, BD Pharmingen, 557659), Fluorescein Isothiocyanate (FITC) anti-mouse CD11b (1:200, BD Pharmingen, 557396), Phycoerythrin (PE) anti-mouse Ly6C (1:200, BD Pharmingen, 560592), APC anti-mouse Ly6G (1:200, BD Pharmingen, 560599). The single cell suspensions of human were stained with the following antibodies: FITC anti-human CD45 (1:100, Absin, abs1840506), PE anti-human CD14 (1:100, Absin, abs1840145), Peridinin-Chlorophyll-Protein-Complex- Cyanine 5.5 (PERCP-CY5.5) anti-human CD11b (1:200, BD Pharmingen, 550993), PE-CY7 anti-human CD66b (1:200, BD Pharmingen, 305116), Brilliant Violet 421 (BV421) anti-human HLA-DR (1:200, BD Pharmingen, 562804). Flow cytometry analyses were performed using Beckman Cytomics FC 500 BD FACSCanto II, and data were analyzed using FlowJo v10.8.1.

### Sorting PMN-MDSC

After obtaining single-cell suspensions, erythrocytes were lysed and treate with GM-CSF and IL-6. After 72 h, cells were harvested and labeled for flow cytometry with antibodies. Fluorescence-activated cell sorting was performed using MoFlo XDP. The sorted PMN-MDSCs were used for subsequent experiments.

### Metabolomics

#### Sample preparation

For metabolomics analysis, six samples were analyzed, comprising three biological replicates per group. Quality control (QC) samples were prepared by pooling equal aliquots of all samples and were injected regularly throughout the analytical run to monitor instrument stability and data quality. Cell pellets (~1 × 10⁷ cells) were lyophilized and extracted with 1000 μL of extraction solvent (methanol/acetonitrile/H_2_O, 2:2:1, v/v/v) containing deuterated internal standards. The mixture was vortexed for 30 s, subjected to three freeze‑thaw cycles (1 min in liquid nitrogen followed by thawing at room temperature, each with 30 s vortexing), sonicated in an ice‑water bath for 10 min, and then incubated at −40 °C for 1 h. After centrifugation at 15,300 × g for 15 min at 4 °C, the supernatant was transferred to an autosampler vial for LC‑MS analysis.

#### LC‑MS/MS analysis

Chromatographic separation was performed on a Vanquish UHPLC system (Thermo Fisher Scientific) equipped with a Waters ACQUITY UPLC BEH Amide column (2.1 × 50 mm, 1.7 μm). Mobile phase A consisted of 25 mM ammonium acetate and 25 mM ammonium hydroxide in water (pH 9.75), and mobile phase B was acetonitrile. The flow rate was set at 0.3 mL/min, with an injection volume of 2 μL and autosampler temperature maintained at 4 °C.

Mass spectrometry was performed using an Orbitrap Exploris 120 mass spectrometer (Thermo Fisher Scientific) equipped with a heated electrospray ionization source operating in both positive and negative ion modes. The ESI source parameters were: sheath gas flow rate 50 Arb, auxiliary gas flow rate 15 Arb, capillary temperature 320 °C, spray voltage 3.8 kV (positive) or −3.4 kV (negative). Data were acquired in information‑dependent acquisition (IDA) mode using Xcalibur software (version 4.4, Thermo Fisher Scientific). Full MS scans were acquired at a resolution of 60,000 (full width at half maximum), followed by MS/MS scans at a resolution of 15,000 with stepped collision energy (SNCE 20/30/40).

#### Data processing

Raw data files were converted to mzXML format using ProteoWizard. Peak detection, retention time alignment, and feature extraction were performed using the XCMS package in R. The R package and the BiotreeDB (V3.0) were applied in metabolite identification. Extracted Ion Chromatogram (EIC) chromatograms and MS/MS spectra of the metabolites are provided in Supplementary Fig. 15–19. The metabolite identification summary table is provided in Supplementary Table 8. After normalization, the variable importance in the projection (VIP) value of each metabolite in the orthogonal partial least-squares discriminant analysis (OPLS-DA) model was calculated to show its contribution to the classification. The values of *p* ≤ 0.05, VIP > 1, and |log2FC | ≥ 1 were considered to be statistically significant and identified as differentially expressed metabolites (DEMs).

### RNA sequencing

Total RNAs were extracted by Trizol reagent (Invitrogen, 15596018). RNA integrity was assessed using the Agilent 2100 Bioanalyzer (Agilent Technologies, Santa Clara, CA, USA). Then the libraries were constructed using VAHTS Universal V6 RNA-seq Library Prep Kit according to the manufacturer’s instructions. The transcriptome sequencing and analysis were conducted by OE Biotech Co., Ltd. (Shanghai, China). Genes with fold changes > 1.5 and *p* value < 0.05 were selected as significantly differentially expressed genes.

### ATAC-seq and ChIP-seq library construction and analysis

The ATAC-seq and ChIP-seq data for MDA-MB-231 cells were obtained from the Gene Expression Omnibus (GSE234179), deposited by our research team (Chen et al., Cancer Commun, 2023). ChIP-seq libraries were sequenced on an Illumina NovaSeq 6000 platform at Cloud-seq (Shanghai, China). ATAC-seq libraries were sequenced on an Illumina X-Ten system. Both generated 150-bp paired-end reads. ATAC-seq and H3K27ac ChIP-seq signals were normalized to RPKM, and comparative log_2_(KO/NC) tracks were generated. ATAC-seq peaks were called from aligned reads using MACS3, and a union peak set was intersected with transcriptional start sites (TSSs, UCSC refGene, hg38) to define promoter-proximal regions. Visualization matrices were generated using deepTools computeMatrix in reference-point mode with a window of ±2 kb and a bin size of 100 bp.

### RNA extraction and real-time quantitative PCR assay

RNA extraction was performed using kits from Beyotime manufacturer (No. R0027). Reverse transcription was achieved using HiScript ^®^ Ⅲ RT SuperMix for qPCR ( + gDNA wiper) kit from Vazyme (No. R323). The RT-qPCR was performed with AceQ Universal SYBR qPCR Master Mix (Vazyme Biotech) using 7300Plus Real-Time PCR System (Eppdorf realplex) according to the manufacturer’s instructions. The mRNA level of each gene was expressed relative to reference gene. Relative expression value was calculated by using the comparative C_t_ method (2^−ΔCt^). The primers used for RT-qPCR were purchased from Genewiz (Suzhou, China) and are listed in Supplementary Table 8.

### CHIP-qPCR assay

Chromatin immunoprecipitation was performed using the Pierce™ Magnetic ChIP Kit (Thermo Fisher Scientific, 26157) according to the manufacturer’s protocol. Cross-linked chromatin was digested with micrococcal nuclease and immunoprecipitated overnight with an anti-ARID3A antibody or control IgG. The purified DNA was analyzed by quantitative PCR using primer pairs specific to the target genomic region (sequence listed in Supplementary Table 9). The enrichment of each region was calculated using the percentage of input method and is presented as fold change relative to the IgG control.

### Dual-luciferase reporter assay

To investigated the functional role of a specific ARID3A binding site (chr14: 67623164-67623169) within the *ARG2* enhancer, both the WT and MUT versions of the human *ARG2* enhancer region were cloned into the pGL3-Promoter luciferase reporter plasmid (GeneAdv Co. Ltd, Suzhou, China). Their sequences are provided in Supplementary Table 10. Cells were seeded in a 24-well plate at a density of 1 × 10⁵ cells per well. Cells were then co-transfected with 0.5 μg of the respective firefly luciferase reporter plasmid (WT or MUT) and 10 ng of the pRL-TK Renilla luciferase control plasmid (GeneAdv Co. Ltd) for normalization. After 48 hours had elapsed post-transfection, the luciferase activity was measured using the Dual-Luciferase Reporter Assay System Kit (RG027, Beyotime, China), strictly adhering to the protocol provided by the manufacturer.

### Ornithine, spermine and total polyamine measurement

Levels of ornithine and total polyamine in cultured cells and tumor tissues were measured using ornithine ELISA kit (JM-12619M1) and total polyamine assay kit (Abcam, ab239728), respectively. For cultured cells and tumor tissues, 100 μL of ice-cold ornithine or polyamine assay buffer was added to 1 × 10^6^ pelleted cells or 10 mg tissue samples. Samples were homogenized with a Dounce homogenizer, centrifuged at 10000 g for 10 min at 4 °C, and supernatants were collected. Supernatants from cultured cells, tumor tissues were transferred into a microcon-10kDa centrifugal filter (Millipore) and filtrates were collected by centrifugation at 10000 g for 20 min at 4 °C. For the measurement of ornithine and polyamines, the reaction mixtures were prepared according to the instructions of the respective assay kits. The prepared mixture was then added to a black 96-well plate and incubated for 30 minutes at room temperature in the dark. Subsequently, the fluorescence was measured using a Biotek Synergy 4 multimode microplate reader.

Furthermore, UHPLC-MS/MS analysis was performed to quantify polyamine-related metabolites, with three biological replicates per group. For sample preparation, each sample was mixed with 200 μL of acetonitrile, vortexed for 30 s, sonicated in an ice‑water bath for 15 min, and incubated at −40 °C for 1 h. After centrifugation at 15,300 × *g* for 15 min at 4 °C, 100 μL of the supernatant was taken for derivatization. Derivatization was performed by adding 50 μL of 100 mM sodium bicarbonate and 50 μL of 20 mg/mL dansyl chloride in acetonitrile, followed by incubation at 40 °C for 1 h in the dark. The reaction was quenched with 50 μL of 1% formic acid in water, vortexed for 30 s, and centrifuged at 15,300 × *g* for 15 min at 4 °C. The final supernatant (100 μL) was transferred to an autosampler vial for UHPLC‑MS/MS analysis. Chromatographic separation was performed on an Agilent 1290 Infinity II series UHPLC system (Agilent Technologies) equipped with a Waters ACQUITY UPLC HSS T3 column (100 × 2.1 mm, 1.8 μm). The mobile phase A was 10 mM ammonium formate with 0.1% formic acid in water, and mobile phase B was acetonitrile. The injection volume was 2 μL. Mass spectrometry was carried out on an Agilent 6460 triple quadrupole mass spectrometer (Agilent Technologies) equipped with an AJS‑electrospray ionization source operated in positive and negative ion modes. Multiple reaction monitoring (MRM) was used for data acquisition. The ion source parameters were set as follows: capillary voltage +4000 V (positive) or −3500 V (negative), nozzle voltage +500 V or −500 V, gas (N₂) temperature 300 °C, gas flow 5 L/min, sheath gas temperature 250 °C, sheath gas flow 11 L/min, and nebulizer pressure 45 psi. Calibration curves were constructed using a series of standard solutions. A linear regression with 1/x weighting was applied, and all analytes showed correlation coefficients (R²) > 0.9974. Data acquisition and processing were performed using Agilent MassHunter Workstation Software (B.08.00, Agilent Technologies). The concentration of each analyte in the sample was obtained from the calibration curve. Levels of ornithine and spermine in peripheral serum were measured using ornithine ELISA kit (JM-12619M1; JX-76717B2) and spermine ELISA kit (JM-12608M2; JX-76716B2).

### Western blotting

Cells were lysed with cell lysis buffer (Beyotime, P0013) with protease inhibitor (Thermo Fisher Scientific, catalog no. 78438). Protein concentrations were calculated using a BCA Protein Assay Kit (Beyotime, P0011). Protein extracts were diluted with 5× SDS sample loading buffer (Yeasen, 20315ES05) and boiled for 10 min. Samples were subjected to SDS-PAGE for separation and then transferred to PVDF membranes (Merck Millipore). The membranes were blocked with 5% milk in TBST buffer at room temperature for 1 hour. Subsequently, they were incubated with primary antibodies at 4 °C overnight. After washing with TBST buffer, the membranes were incubated with HRP-conjugated secondary antibodies at room temperature for 1 hour, and then washed again with TBST buffer. Blots were exposed using LAS 4000 or Typhoon (GE Health). Exposed blots were quantified by densitometry analysis using ImageJ software. The antibodies used were listed in Supplementary Table 11.

### Immunohistochemistry and Immunofluorescence staining

For IHC and IF staining, tumor tissues were initially fixed in formalin, dehydrated through graded alcohols, and embedded in paraffin. Sections were deparaffinized with xylene, rehydrated, and treated with 3% H₂O₂ in methanol to quench endogenous peroxidase activity. Antigen retrieval was achieved using citric acid under high-temperature and high-pressure conditions. Sections were blocked with animal non-immune serum and incubated with primary antibodies overnight at 4 °C. Following washing, secondary antibodies were applied for 30 minutes at room temperature, and staining was developed using a DAB detection kit (E-IR-R101). Nuclei were counterstained with hematoxylin for IHC, or with DAPI for IF. IHC images were captured using a digital slide scanner (KFBIO KF-PRO-120). IF images were captured using a digital slide scanner (PANNORAMIC SCAN II). Antibody details are provided in Supplementary Table 11.

### Tyramide signal amplification (TSA)

Multiplex immunofluorescence was performed using a TSA system. Two TMAs were processed following standard deparaffinization, rehydration, and antigen retrieval in Tris-EDTA buffer (pH 9.0). Endogenous peroxidase activity was quenched with 3% H₂O₂. Sequential labeling was carried out through repeated cycles, each consisting of incubation with a primary antibody overnight at 4 °C, followed by an HRP-conjugated secondary antibody and signal development with fluorophore-conjugated tyramide. Between cycles, antibody complexes were thoroughly removed by repeated antigen retrieval to prevent cross-reactivity. After the final cycle, nuclei were stained with DAPI. Whole-slide scanning was performed using a digital slide scanner (PANNORAMIC SCAN II). Primary antibody details are provided in Supplementary Table 11.

### Ornithine and spermine supplementation

In mouse models supplemented with ornithine and spermine, we constructed intracardial and intratibial injection models. After wildtype 4T-1 cells inoculation, mice were intraperitoneally injected with spermine (10 mg/kg, Merck, 55513-100MG), ornithine (10 mg/kg, MCE, HY-B1352) or saline once a day^44,65^. To evaluate the combined effects, an intracardial injection model was established. Following wildtype 4T-1 cell inoculation, mice received daily intraperitoneal injections of spermine, ornithine, or their combination, using the same dosage and frequency as described above. On day 14 post-inoculation, mice were euthanized. For mice with intracardiac injection, bone marrow was collected for flow cytometric analysis. For mice with intratibial injection, bone tumors were harvested for subsequent IF and flow cytometry.

### Hematoxylin-eosin (H&E) staining

The heart, liver, spleen, lungs and kidneys were excised from the mice, fixed, embedded and then sectioned. The bones and tumor-bearing bones were fixed in 10% neutral-buffered formalin, decalcified in 10% EDTA for 2 weeks and embedded in paraffin. Sections were stained with hematoxylin (Sigma, H3136) for 5 minutes and differentiated with acid alcohol. After rinsing, they were counterstained with eosin (Sigma, E4009) for 1–2 minutes. Finally, sections were dehydrated, cleared in xylene, and mounted. Images were captured by a digital slide scanner (KFBIO KF-PRO-120).

### Blood routine and biochemical testing

Blood routine tests were performed using an automated hematology analyzer. Parameters measured included white blood cell count, red blood cell count, hemoglobin concentration, hematocrit, platelet count and so on. For biochemical testing, serum was separated from blood samples by centrifugating at 3000 g for 15 minutes at 4 °C. Biochemical assays include liver and renal function tests. Liver function was assessed by measuring serum level of ALT, AST and ALP. Renal function markers were evaluated using standard enzymatic methods.

### Statistics and reproducibility

Samples were compared statistically using the GraphPad Prism software (Version 6.0) and R for Windows (R 4.1.1). Survival curves were calculated according to the Kaplan-Meier method. Flow cytometric plots were generated using Flowjo ver10. The statistical significance of comparisons between two groups was analyzed with a two-tailed Student’s t-test. One­way analysis of variance (ANOVA) was used to compare differences among multiple groups. Two-way ANOVA was used to assess the interaction between two independent factors. Categorical variables were analyzed using the two-tailed Chi-square test when all expected cell frequencies were ≥ 5; otherwise, two-tailed Fisher’s exact test was employed for small sample sizes or sparse data. All statistical tests were with the significant level being set at *p* < 0.05.

### Reporting summary

Further information on research design is available in the Nature Portfolio Reporting Summary linked to this article.

## Supplementary information

Supplementary Information

Reporting Summary

Transparent Peer Review file

## Source data

Source data

## Data Availability

The raw single-cell sequencing data generated in this study have been deposited in the Genome Sequence Archive at National Genomics Data Center, China. BioProject: PRJCA043637 (https://ngdc.cncb.ac.cn/gsa/browse/CRA028289). The raw RNA-Sequencing data generated in this study have been deposited in National Center for Biotechnology Information. BioProject: PRJNA1295162. The mass spectrometry-based untargeted metabolomics data have been deposited to Metabolights under the accession number MTBLS12768. The CHIP-Seq and ATAC-Seq data were downloaded from the GEO under accession code GSE234179. The remaining data are available within the Article, Supplementary Information or Source Data file. Source data are provided with this paper.

## Code availability

The code used to analyse the dataset is openly available at GitHub (https://github.com/shuangyuePan/Bone-marrow-ScRNA-Seq) and the 10.5281/zenodo.19248693.
