# Supplementary material for: Targeting arginine metabolism reverses bone immunosuppressive microenvironment and metastasis in ARID1A-deficient triple negative breast cancer
Source: Nat Commun. 2026 May 26;17:6863. doi: 10.1038/s41467-026-73574-3 (PMC13389217; doi:10.1038/s41467-026-73574-3)
Supplement: Supplementary file 1 — Supplementary Information [file 41467_2026_73574_MOESM1_ESM.pdf]

Targeting arginine metabolism reverses bone immunosuppressive microenvironment and metastasis in *ARID1A*-deficient triple negative breast cancer

Authors

Shuangyue Pan[1,2#], Jinyan Wang[1,2#], Boya Wang[1,2#], Fangqian Wang[3], Qingjian Chen[4], Tiantian Liu[1,2], Aima Zhang[1,2], Shenyuqi Wu[1,2], Bin Li[1,2], Hai Hu[5], Mengdi Yang[1,2*], Zhonghua Tao[1,2*], Xichun Hu[1,2*]

Correspondence to:

Xichun Hu

E-mail: xchu2009@hotmail.com

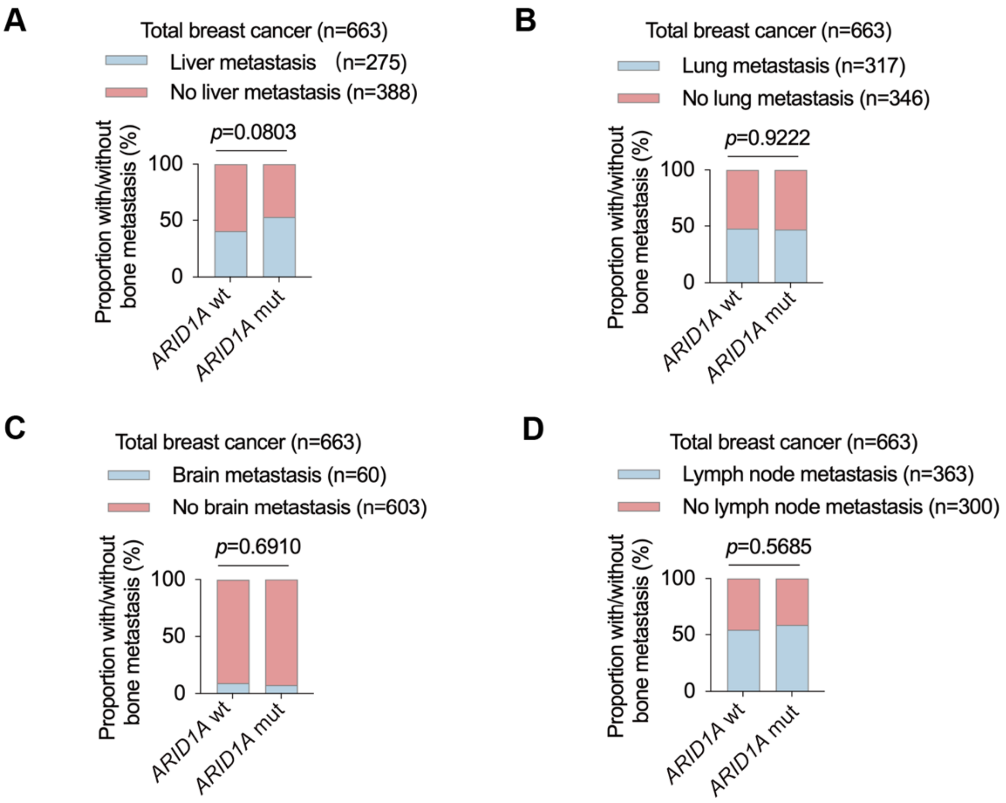

Supplementary Fig. 1 Impact of *ARID1A* mutations on metastatic sites in breast cancer patients. (**A-D**) Statistical analysis of the incidence of liver, lung, brain, and lymph node metastases in breast cancer patients with and without *ARID1A* mutations. Two-sided Pearson's chi-square test was used. All *p* values are indicated in the figures. *ARID1A* wt, *ARID1A* wild-type. *ARID1A* mut, *ARID1A* mutations. Source data are provided as a Source Data file.

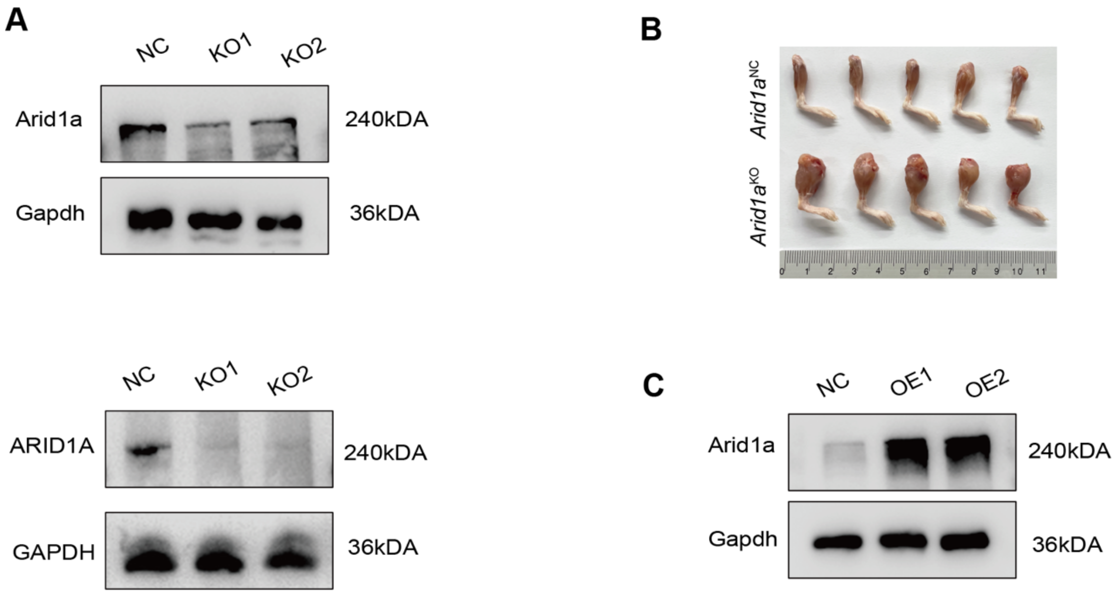

Supplementary Fig. 2 Validation of *ARID1A* stable transfection cell lines and in vivo tumor progression following *Arid1a* knockout. (**A**) Western blot analysis of ARID1A expression in *Arid1a*^{NC} and *Arid1a*^{KO} 4T-1 cells; and in *ARID1A*^{NC} and *ARID1A*^{KO} MDA-MB-231 cells. (**B**) Intratibial tumor progression was increased in mice injected with *Arid1a*^{KO} cells compared to those injected with *Arid1a*^{NC} cells, as demonstrated by tumor image. (**C**) Western blot analysis of Arid1a expression in *Arid1a*^{NC} and *Arid1a*^{OE} 4T-1 cells. The experiment was repeated independently three times. Source data are provided as a Source Data file.

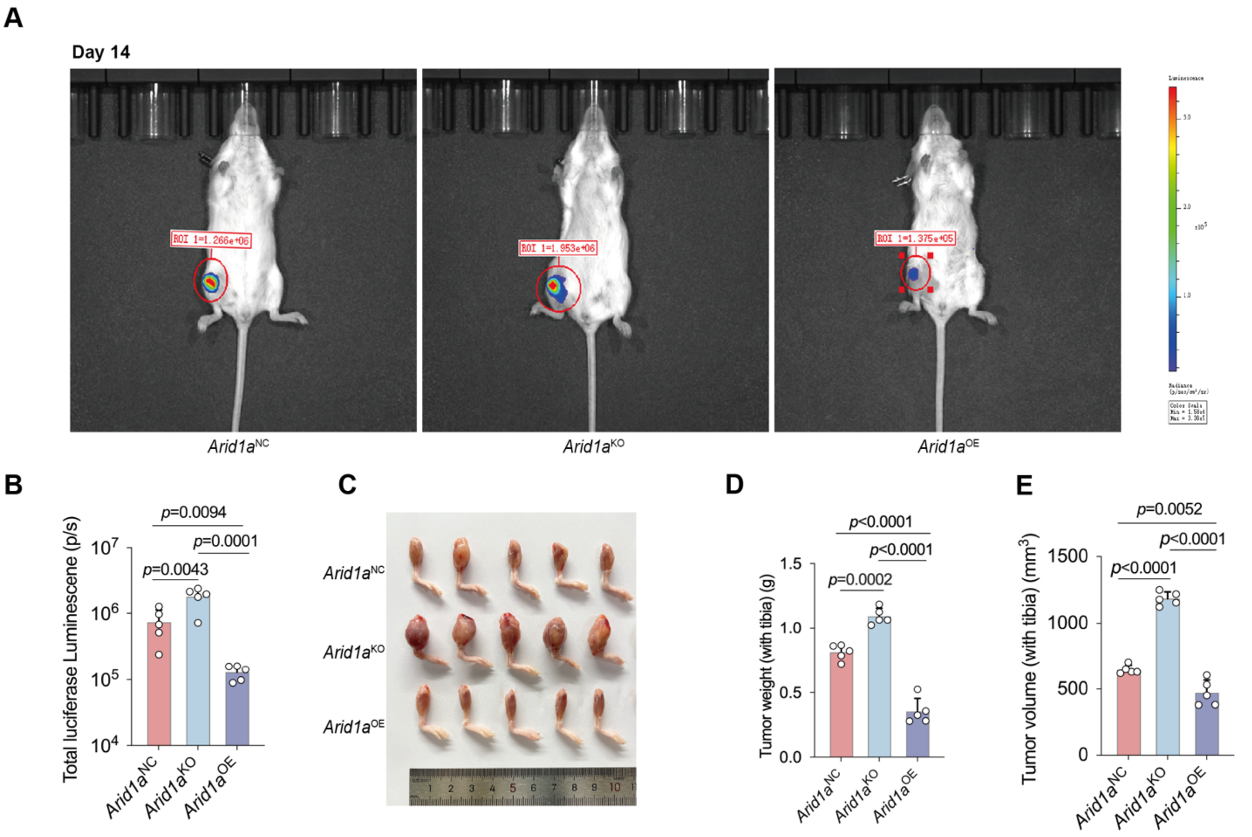

Supplementary Fig. 3 *ARID1A* correlates with bone metastasis in TNBC. (**A**) Representative bioluminescence imaging (BLI) images of three groups of mice after intratibial injection with equal numbers of *Arid1a*^{NC}, *Arid1a*^{KO} and *Arid1a*^{OE} 4T-1 cells. n=5 mice per group. (**B**) Quantification of the BLI intensity in the right leg region of the three groups of mice after intratibial injection. Data are presented as mean ± SD (n=5 mice per group), one-way ANOVA for multiple comparisons. (**C-E**) Tumor image (C), tumor weight (D) and tumor volume (E) in three groups. Data are presented as mean ± SD (n=5 mice per group), one-way ANOVA for multiple comparisons. All *p* values are indicated in the figures. Source data are provided as a Source Data file.

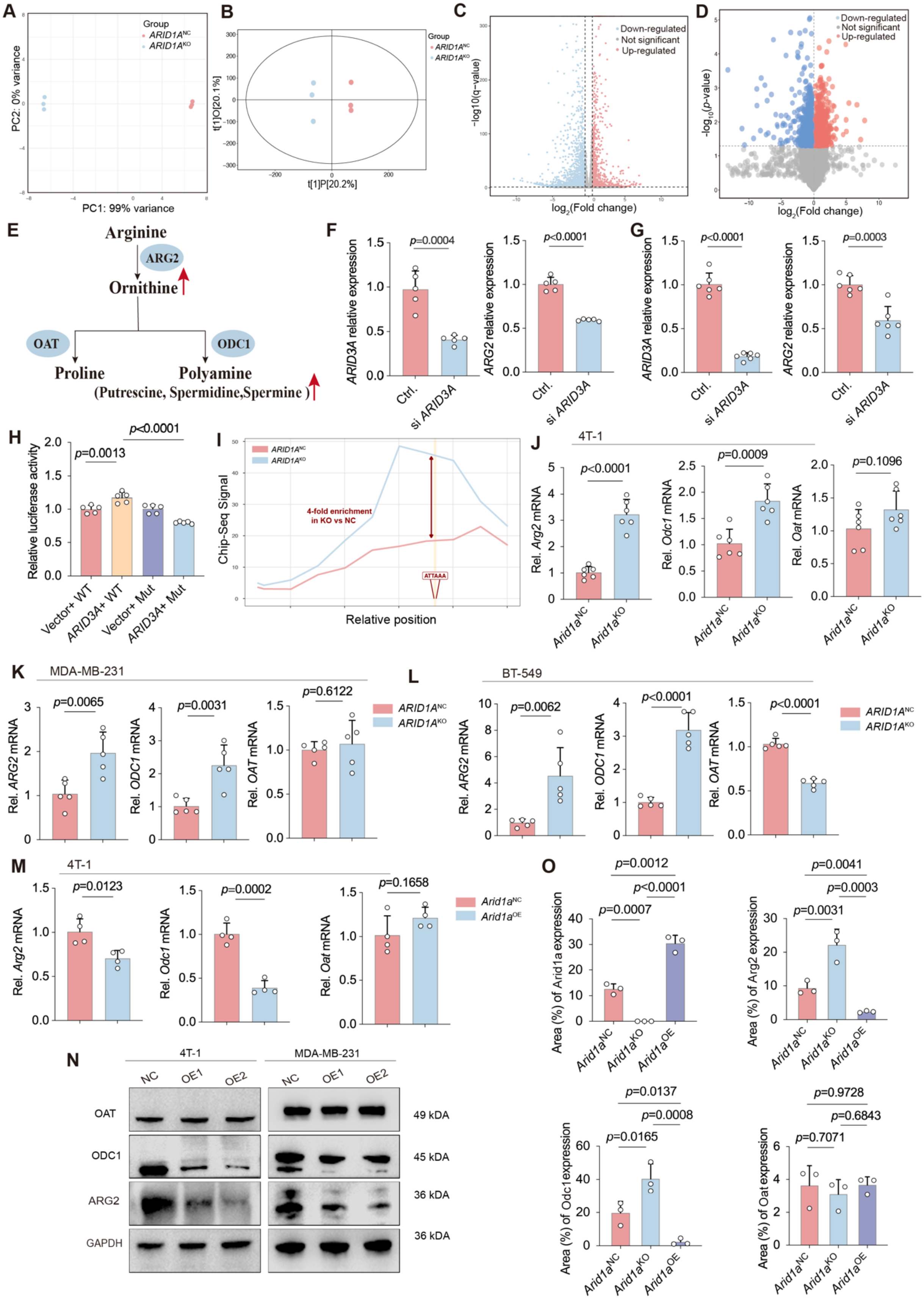

A
Group
ARID1A^NC
ARID1A^KO
PC2: 0% variance
PC1: 99% variance
B
Group
ARID1A^NC
ARID1A^KO
t[1]O[20.1%]
t[1]P[20.2%]
C
Down-regulated
Not significant
Up-regulated
−log10(q−value)
log2(Fold change)
D
Down-regulated
Not significant
Up-regulated
-log10(p-value)
log2(Fold change)
E
Arginine
ARG2
Ornithine
OAT
ODC1
Proline
Polyamine
(Putrescine, Spermidine,Spermine)
F
p=0.0004
ARID3A relative expression
Ctrl.
si ARID3A
p<0.0001
ARG2 relative expression
G
p<0.0001
ARID3A relative expression
p=0.0003
ARG2 relative expression
H
p=0.0013
p<0.0001
Relative luciferase activity
Vector+ WT
ARID3A+ WT
Vector+ Mut
ARID3A+ Mut
I
ARID1A^NC
ARID1A^KO
4-fold enrichment in KO vs NC
ATTAAA
Chip-Seq Signal
Relative position
J
4T-1
p<0.0001
Rel. Arg2 mRNA
p=0.0009
Rel. Odc1 mRNA
p=0.1096
Rel. Oat mRNA
Arid1a^NC
Arid1a^KO
K
MDA-MB-231
p=0.0065
Rel. ARG2 mRNA
p=0.0031
Rel. ODC1 mRNA
p=0.6122
Rel. OAT mRNA
ARID1A^NC
ARID1A^KO
L
BT-549
p=0.0062
Rel. ARG2 mRNA
p<0.0001
Rel. ODC1 mRNA
p<0.0001
Rel. OAT mRNA
ARID1A^NC
ARID1A^KO
M
4T-1
p=0.0123
Rel. Arg2 mRNA
p=0.0002
Rel. Odc1 mRNA
p=0.1658
Rel. Oat mRNA
Arid1a^NC
Arid1a^OE
N
4T-1
MDA-MB-231
NC
OE1
OE2
OAT
ODC1
ARG2
GAPDH
49 kDA
45 kDA
36 kDA
36 kDA
O
p=0.0012
p<0.0001
p=0.0007
Area (%) of Arid1a expression
p=0.0041
p=0.0003
p=0.0031
Area (%) of Arg2 expression
p=0.0137
p=0.0008
p=0.0165
Area (%) of Odc1expression
p=0.9728
p=0.6843
p=0.7071
Area (%) of Oat expression
Arid1a^NC
Arid1a^KO
Arid1a^OE

Supplementary Fig. 4 *ARID1A*-deficient TNBC upregulates the arginine metabolic pathway. (**A**) Principal Component Analysis (PCA) plot of RNA-Seq from $ARID1A^{NC}$ and $ARID1A^{KO}$ cells. (**B**) Orthogonal Partial Least Squares Discriminant Analysis (OPLS-DA) plot of metabolomics from $ARID1A^{NC}$ and $ARID1A^{KO}$ cells. (**C**) Volcano plot of differentially expressed genes from RNA-Seq in $ARID1A^{NC}$ and $ARID1A^{KO}$ cells. (**D**) Volcano plot of differential metabolites from metabolomics in $ARID1A^{NC}$ and $ARID1A^{KO}$ cells. (**E**) Scheme diagram of the arginine metabolic flux and associated key enzymes. (**F**) *ARG2* mRNA expression in MDA-MB-231 cells upon si*ARID3A* knockdown. Data are presented as mean ± SD (n=5 biological replicates per group). Unpaired two-tailed Student's t-test was used. (**G**) *ARG2* mRNA expression in BT-549 cells upon si*ARID3A* knockdown. Data are presented as mean ± SD (n=6 biological replicates per group). Unpaired two-tailed Student's t-test was used. (**H**) Luciferase activity of the *ARG2* enhancer upon *ARID3A* overexpression in BT-549 cells co-transfected with WT or MUT reporter constructs. Data are presented as mean ± SD (n=5 biological replicates per group), two-way ANOVA for multiple comparisons. WT, wild-type; MUT, mutant. (**I**) H3K27ac ChIP−seq signal enrichment at the ARID3A-binding site within the *ARG2* enhancer in $ARID1A^{KO}$ and $ARID1A^{NC}$ MDA-MB-231 cells. (**J-L**) Relative mRNA expression levels of *ARG2*, *ODC1*, and *OAT* in the $ARID1A^{NC}$ and $ARID1A^{KO}$ groups in 4T-1 (J), MDA-MB-231 (K) and BT-549 cells (L). Data are presented as mean ± SD, unpaired two-tailed Student's t test was used. (**M**) Relative mRNA expression levels of *Arg2*, *Odc1* and *Oat* in the $Arid1a^{NC}$ and $Arid1a^{OE}$ groups in 4T-1 cells. Data are presented as mean ± SD (n=4 biological replicates per group), unpaired two-tailed Student's t test was used. (**N**) Western blot analysis of the expression levels of ARG2, ODC1 and OAT in the $ARID1A^{NC}$ and $ARID1A^{OE}$ groups in 4T-1 and MDA-MB-231 cells. The samples derive from the same experiment but different gels for ARG2, another for ODC1, and another for OAT were processed in parallel. The experiment was repeated independently three times. (**O**) Quantification of the protein expression levels of Arid1a, Arg2, Odc1 and Oat in Immunohistochemistry (IHC) images of the tumors in each group. Data are presented as mean ± SD (n=3 mice per group), one-way ANOVA for multiple comparisons. Source data are provided as a Source Data file.

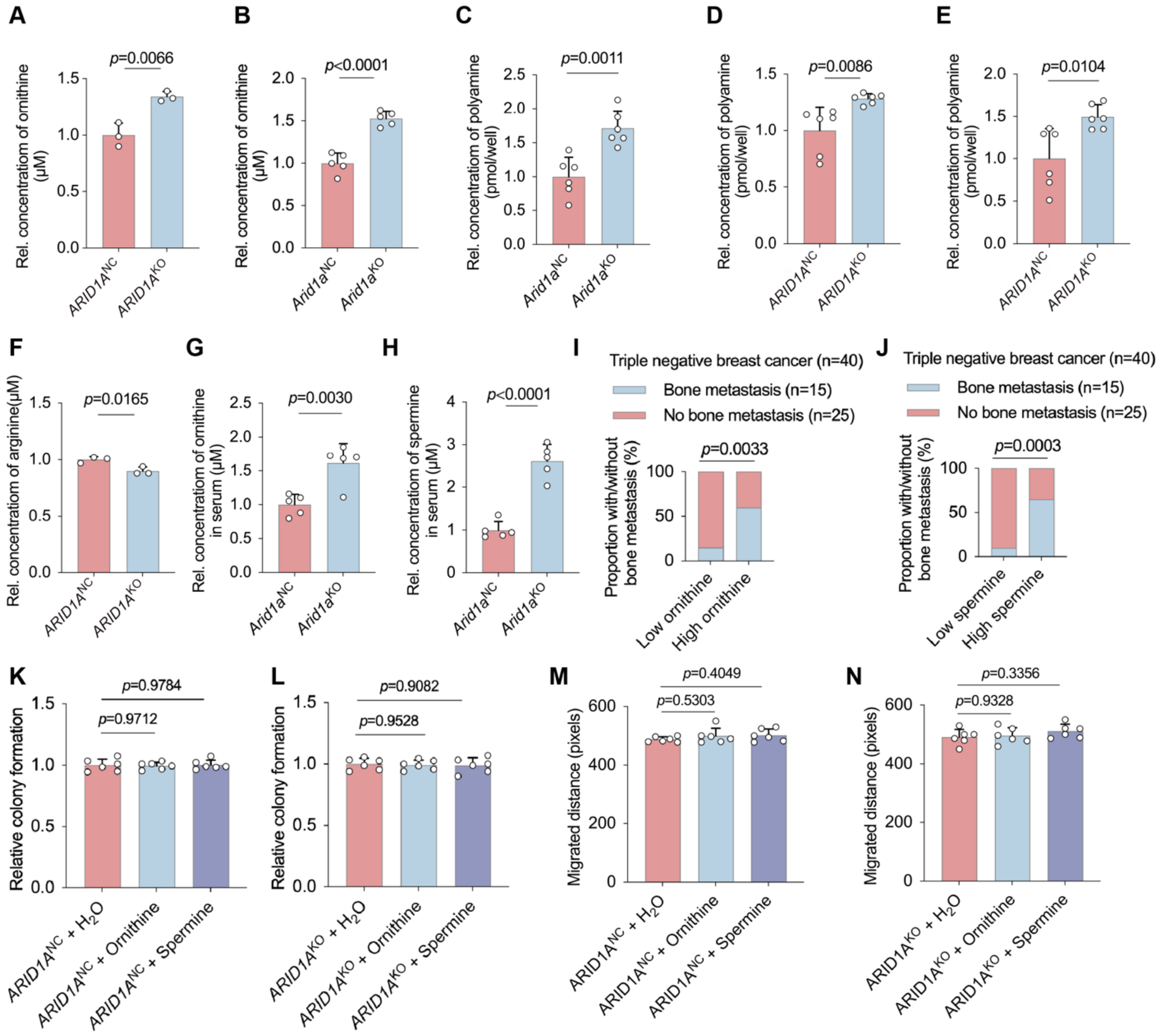

Supplementary Fig. 5 *ARID1A* deficiency promotes ornithine and spermine production which are associated with bone metastasis in TNBC. (**A**) The concentration of ornithine in the *ARID1A*^{NC} and *ARID1A*^{KO} groups in metabolome. Data are presented as mean ± SD (n=3 biological replicates), unpaired two-tailed Student's t test was used. (**B**) ELISA results of ornithine levels in the *Arid1a*^{NC} and *Arid1a*^{KO} groups in 4T-1 cells in vitro. Data are presented as mean ± SD (n=5 biological replicates), unpaired two-tailed Student's t test was used. (**C-E**) ELISA results of polyamine levels in the *ARID1A*^{NC} and *ARID1A*^{KO} groups in 4T-1 (C), MDA-MB-231 (D) and BT-549 cells (E) in vitro. Data are presented as mean ± SD (n=6 biological replicates), unpaired two-tailed Student's t test was used. (**F**) The LC-MS/MS results showed a decrease in arginine levels in the *ARID1A*^{KO} group. Data are presented as mean ± SD (n=3 biological replicates),

unpaired two-tailed Student's t test was used. (**G-H**) The levels of ornithine and spermine in the peripheral blood serum of mice. Data are presented as mean ± SD (n=5 mice), unpaired two-tailed Student's t test was used. (**I**) Association between peripheral blood serum ornithine levels and bone metastasis in TNBC patients. n=40 patients, two-sided Pearson's chi-square test was used. (**J**) Association between serum spermine levels and bone metastasis in TNBC patients. n=40 patients, two-sided Pearson's chi-square test was used. (**K-L**) Clone formation assays of each group. Data are presented as mean ± SD (n=6 biological replicates), one-way ANOVA for multiple comparisons. (**M-N**) Wound healing migration assays of each group. Data are presented as mean ± SD (n=6 biological replicates), one-way ANOVA for multiple comparisons. All *p* values are indicated in the figures. Source data are provided as a Source Data file.

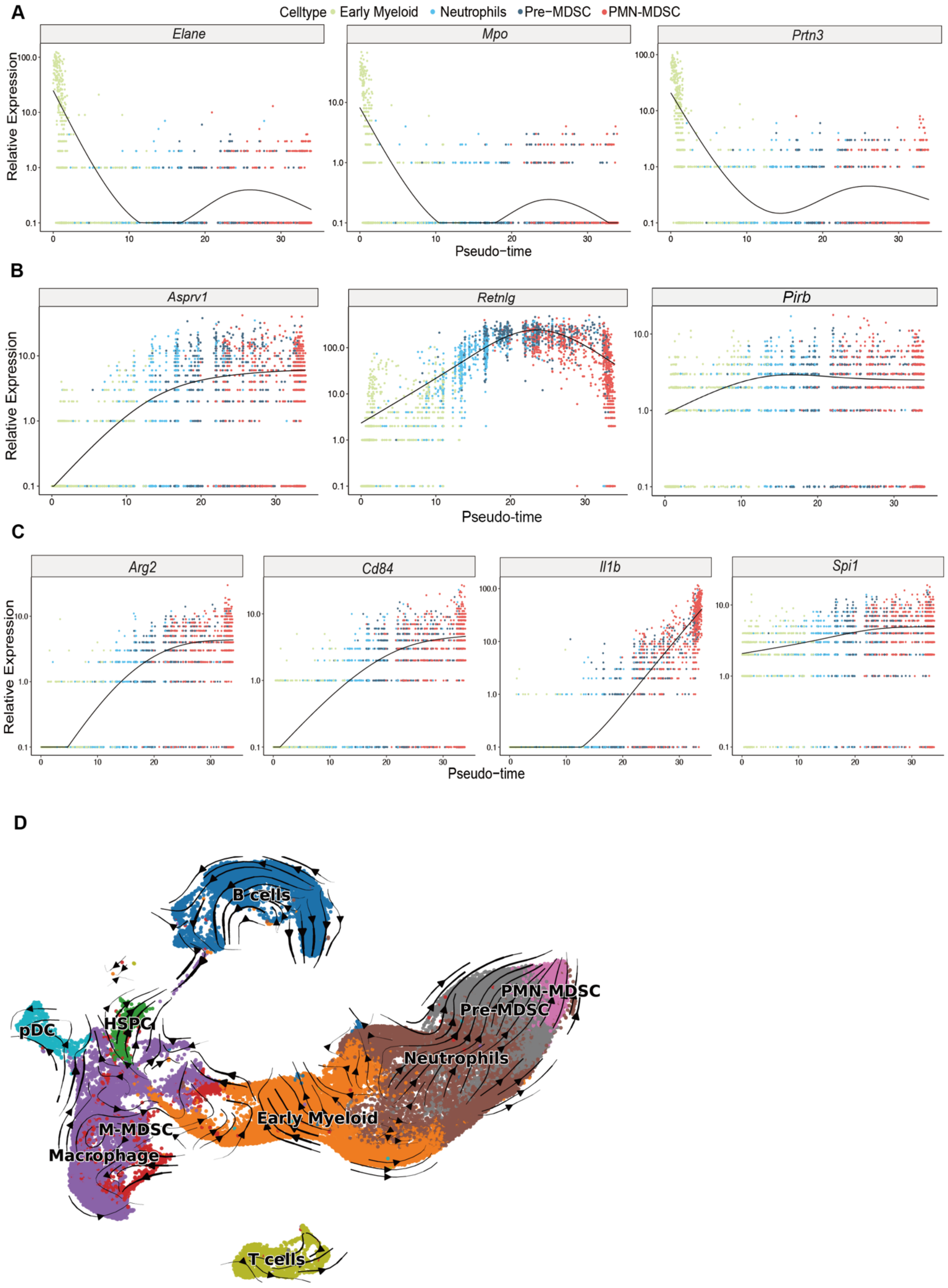
A
Celltype
Early Myeloid
Neutrophils
Pre−MDSC
PMN-MDSC
Elane
Mpo
Prtn3
Relative Expression
Pseudo−time
B
Asprv1
Retnlg
Pirb
Relative Expression
Pseudo−time
C
Arg2
Cd84
Il1b
Spi1
Relative Expression
Pseudo−time
D
B cells
PMN-MDSC
Pre-MDSC
HSPC
pDC
Neutrophils
Early Myeloid
M-MDSC
Macrophage
T cells

Supplementary Fig. 6 The developmental trajectory of PMN-MDSCs in TNBC. (**A**) Early myeloid cells were marked by high expression of *Elane*, *Mpo*, and *Prtn3*. (**B**) Pre-MDSCs were marked by high expression of *Asprv1*, *Pirb* and *Retnlg*. (**C**) PMN-MDSCs were marked by high expression of *Cd84*, *Arg2*, *Il1b*, and *Spi1*. (**D**) ScVelo analysis of the developmental trajectory within granulocytic myeloid clusters.

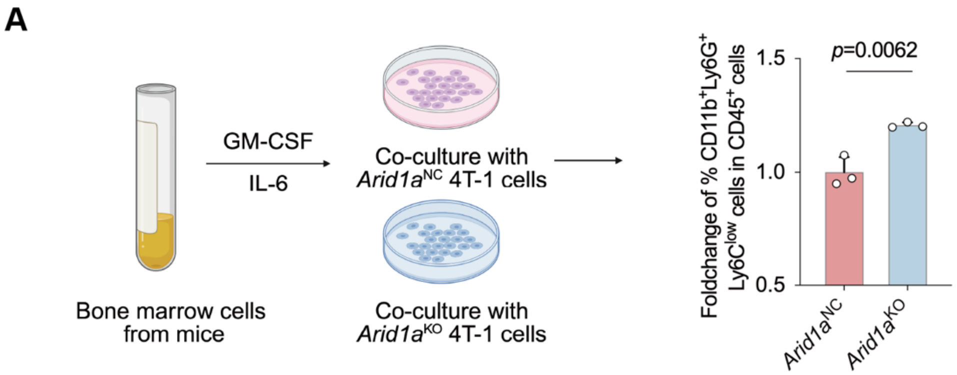

Supplementary Fig. 7 *ARID1A* deficiency drives the expansion of PMN-MDSCs. (A) Proportions of mouse PMN-MDSCs after in vitro co-culture with $Arid1a^{NC}$ and $Arid1a^{KO}$ 4T-1 cells. Data are presented as mean ± SD (n=3 mice per group), unpaired two-tailed Student's t test was used. *p* value is indicated in the figure. Created in BioRender. Pan, S. (2026) https://BioRender.com/fvtznq0. Source data are provided as a Source Data file.

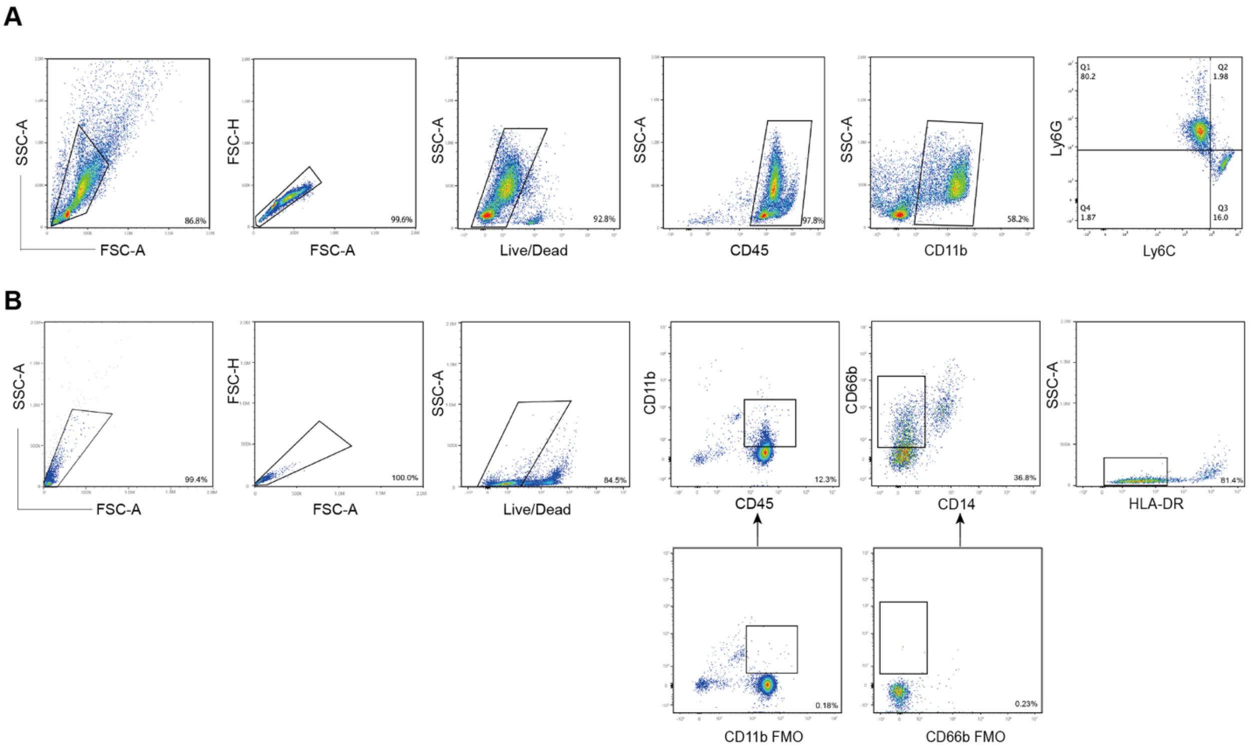

Supplementary Fig. 8 Gating strategies for isolating PMN-MDSCs. (**A**) Gating logic for isolating PMN-MDSCs of mice. This gating strategy corresponds to the FACS data presented in Fig. 5B, 5C, 5D, 6B, 6F, 6I, 6K, 7E, and 7M. (**B**) Gating logic for isolating PMN-MDSCs of humans. This gating strategy corresponds to the FACS data presented in Fig. 5A, 6D, and 7F.

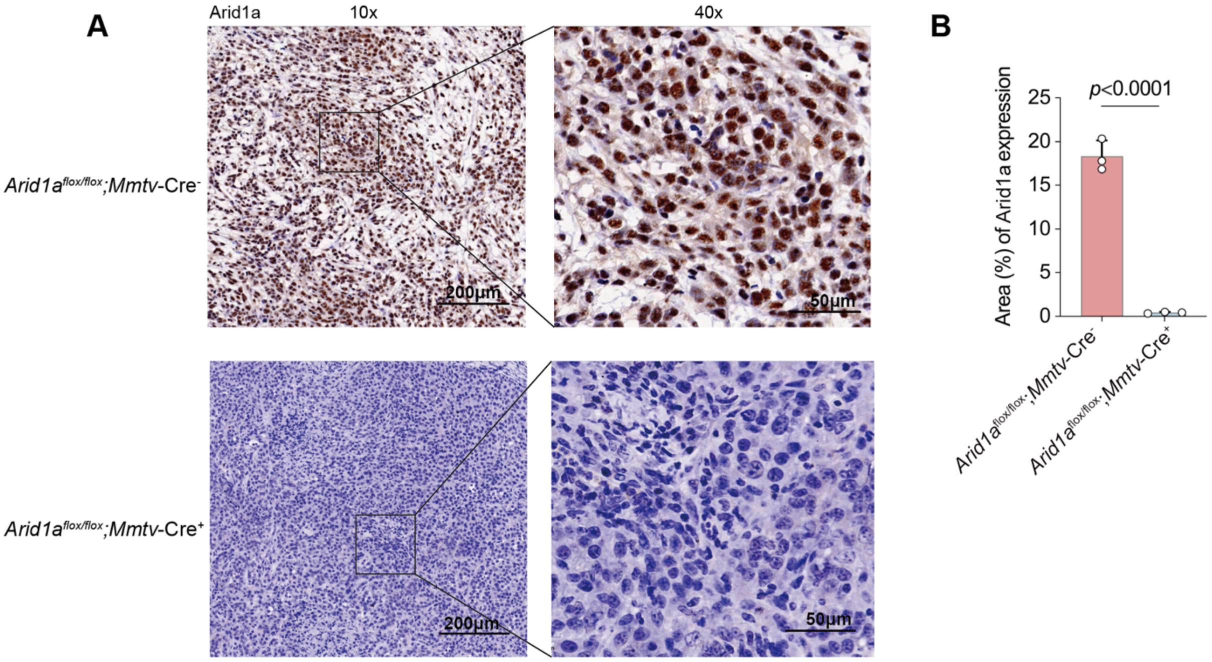

Supplementary Fig. 9 The expression level of Arid1a in *Arid1a*$^{\mathbf{flox/flox}}$; *Mmtv*-Cre mice. (**A**) Immunohistochemistry (IHC) images of Arid1a expression in tumors from *Arid1a*$^{flox/flox}$; *Mmtv*-Cre$^+$ mice compared to *Arid1a*$^{flox/flox}$; *Mmtv*-Cre$^-$ control mice. Scale bar, 10x, 200μm. (**B**) Quantification of the protein expression levels of Arid1a in two groups. Data are presented as mean ± SD (n=3 mice per group), unpaired two-tailed Student's t test was used. *p* value is indicated in the figure. Source data are provided as a Source Data file.

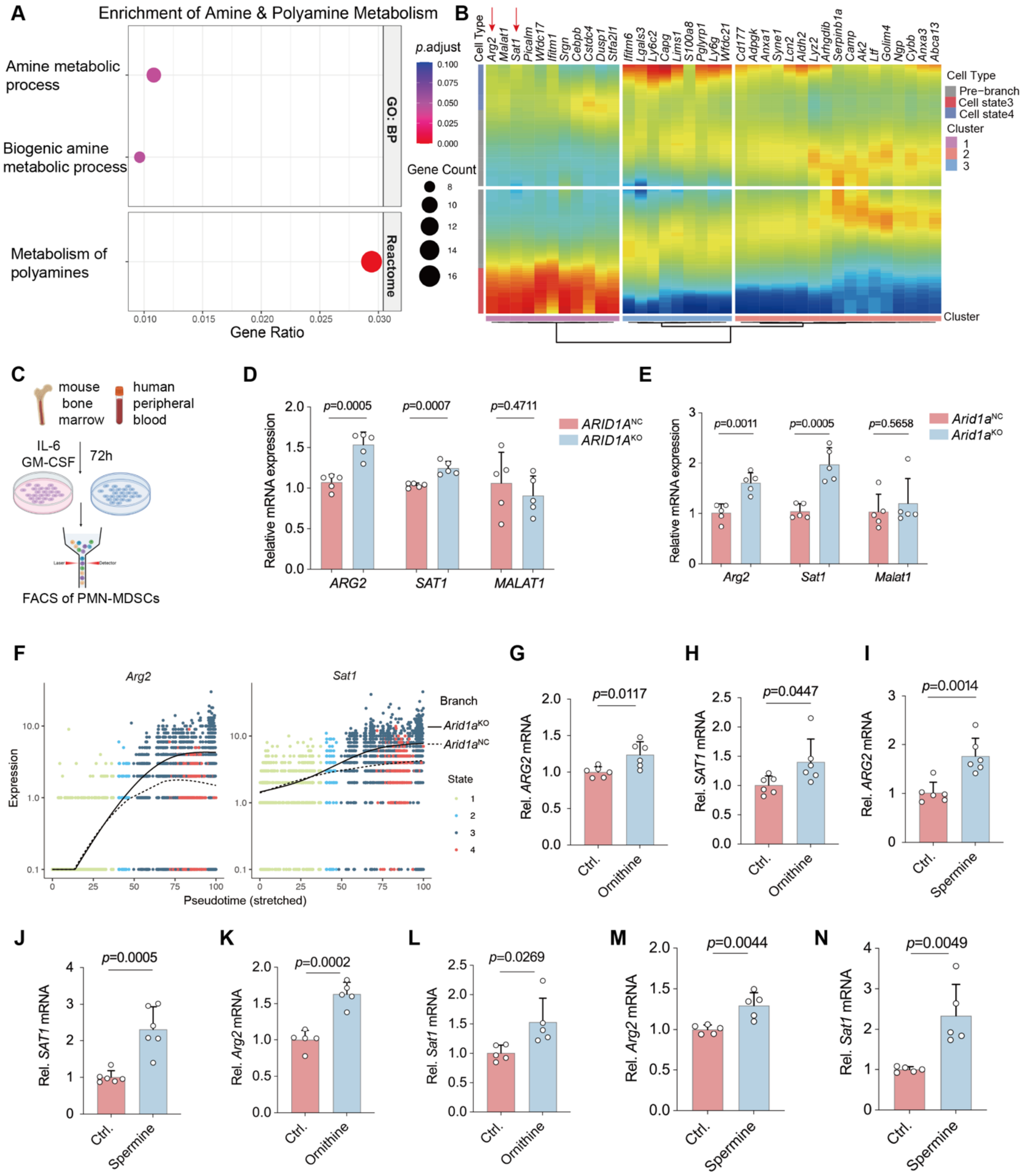

Supplementary Fig. 10 ***ARG2*** **and** ***SAT1*** **are highly expressed in PMN-MDSCs from** ***ARID1A***$^{\mathbf{KO}}$ **group.** (**A**) Enrichment of amine and polyamine metabolism in PMN-MDSCs. Hypergeometric test (one-side) was used for *p*-value calculation. (**B**) Heatmap of the most significantly differentially expressed genes of PMN-MDSCs in the *Arid1a*^{NC} and *Arid1a*^{KO} groups.

(**C**) Schematic diagram of the procedure in which MDSCs were induced from primary mouse bone marrow cells and human PBMCs in vitro respectively, and then FACS was used to sort out PMN-MDSCs for subsequent experiments. Created in BioRender. Pan, S. (2026) https://BioRender.com/ls2cq4k. (**D-E**) Relative mRNA expression of *ARG2*, *MALAT1* and *SAT1* in human and mouse PMN-MDSCs co-cultured with *ARID1A*^{NC} and *ARID1A*^{KO} cells. Data are presented as mean ± SD (n=5 biological replicates per group), unpaired Student's t-test was used. (**F**) Expression dynamics of *Arg2* and *Sat1* over stretched pseudotime. (**G-J**) Relative mRNA expression of *ARG2* and *SAT1* in human PMN-MDSCs co-cultured with ornithine and spermine. Data are presented as mean ± SD (n=6 donors per group), unpaired two-tailed Student's t test was used. (**K-N**) Relative mRNA expression of *Arg2* and *Sat1* in mouse PMN-MDSCs co-cultured with ornithine and spermine. Data are presented as mean ± SD (n=5 mice per group), unpaired two-tailed Student's t test was used. All *p* values are indicated in the figures. Source data are provided as a Source Data file.

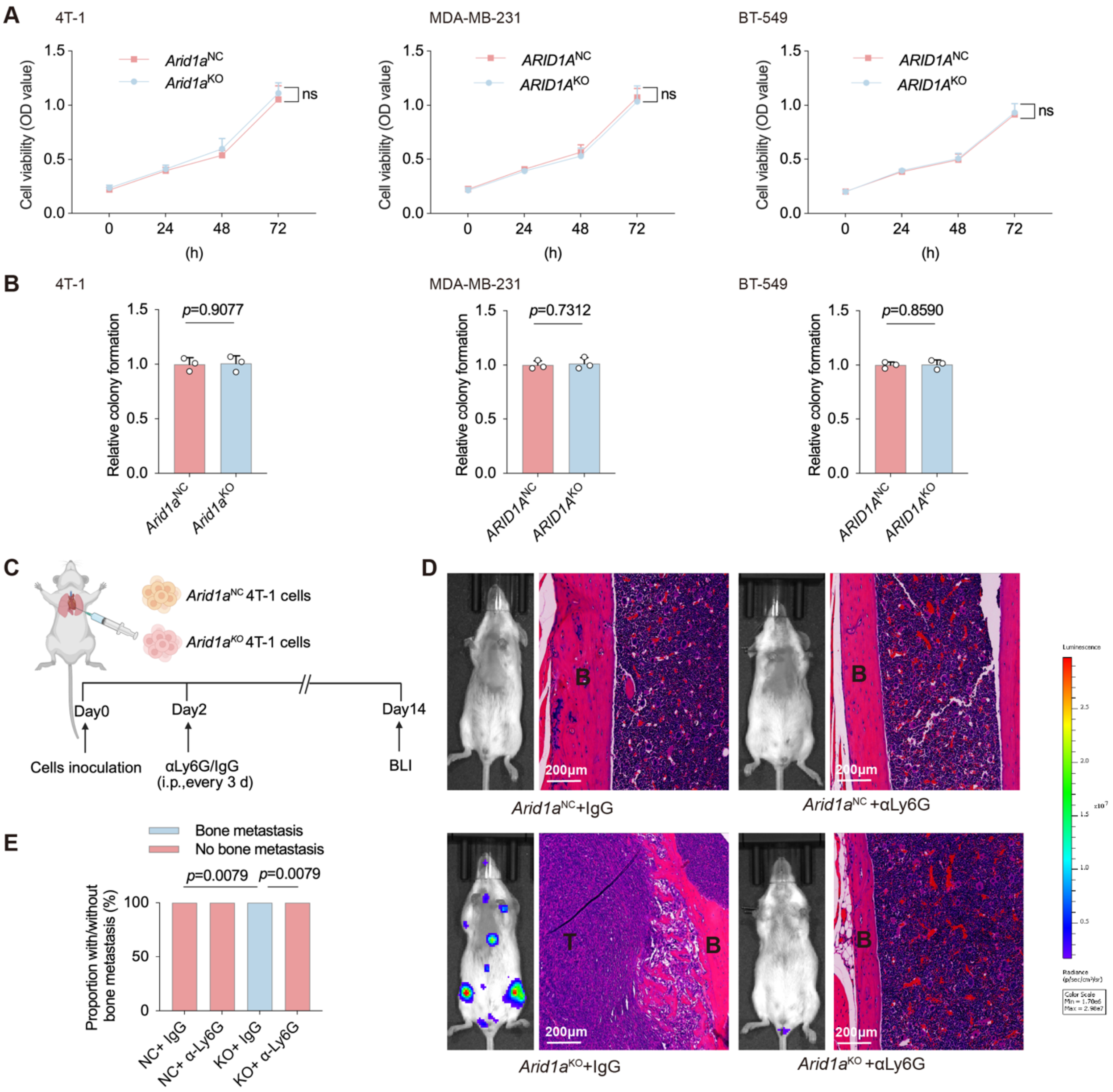

Supplementary Fig. 11 PMN-MDSCs play a critical role in *ARID1A* deficiency-mediated bone metastasis. (**A**) CCK-8 assays of TNBC cells (4T-1, MDA-MB-231, BT-549) between *ARID1A*^{NC} and *ARID1A*^{KO} group. n=7 biological replicates, two-way ANOVA was used. (**B**) Clone formation assays of TNBC cells (4T-1, MDA-MB-231, BT-549) between *ARID1A*^{NC} and *ARID1A*^{KO} group. Data are presented as mean ± SD (n=3 biological replicates per group), unpaired two-tailed Student's t test was used. (**C**) Treatment schedule schematic. Following intracardiac injection of *Arid1a*^{NC} and *Arid1a*^{KO} 4T-1 cells, mice were allocated to receive either anti-Ly6G antibody or isotype control IgG. BLI, bioluminescence imaging. Created in BioRender. Pan, S.

(2026) https://BioRender.com/puypue9. (**D**) BLI and H&E staining images of mice in *Arid1a*^{NC} plus IgG, *Arid1a*^{NC} plus αLy6G, *Arid1a*^{KO} plus IgG， *Arid1a*^{NC} plus αLy6G group. Scale bar, 200μm. B bone, T tumor. n=5 mice per group. (**E**) Statistical charts presenting the bone metastasis incidence in each group. n=5 mice, data was analyzed by two-sided Fisher's exact test. *p* values are indicated in the figures. Source data are provided as a Source Data file.

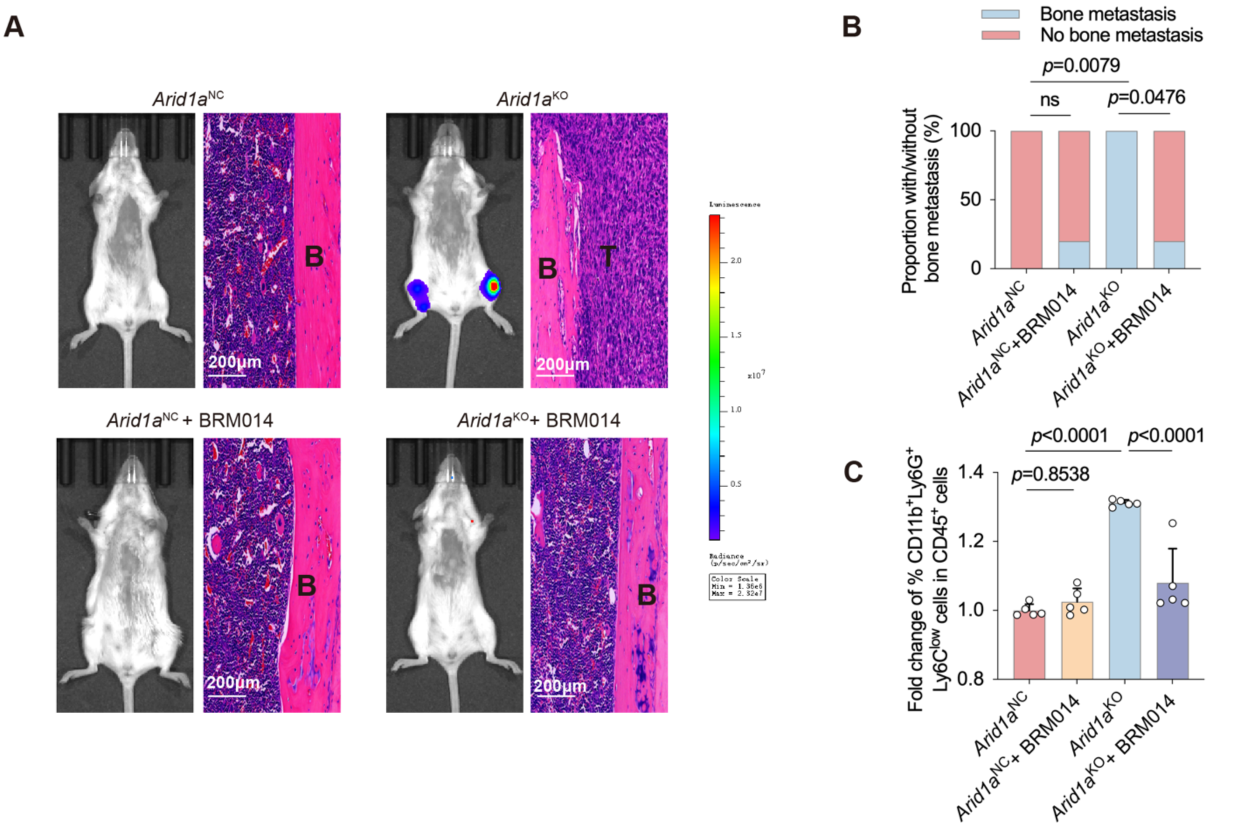

Supplementary Fig. 12 *ARID1A* deficiency relies on residual SWI/SNF complex to promote bone metastasis. (**A**) Bioluminescence imaging (BLI) and H&E staining images of mice in *Arid1a*^{NC}, *Arid1a*^{NC} plus BRM014, *Arid1a*^{KO}, *Arid1a*^{KO} plus BRM014 group with intracardiac injection. Scale bar, 200μm. B bone, T tumor. (**B**) Statistical charts presenting the bone metastasis incidence in each group with intracardiac injection. n=5 mice per group, two-sided Fisher's exact test was used. (**C**) Bone marrow of mice was harvested from each group and analyzed by flow cytometry to quantify PMN-MDSC proportions. Data are presented as mean ± SD (n=5 mice per group), one-way ANOVA for multiple comparisons. *p* values are indicated in the figures. Source data are provided as a Source Data file.

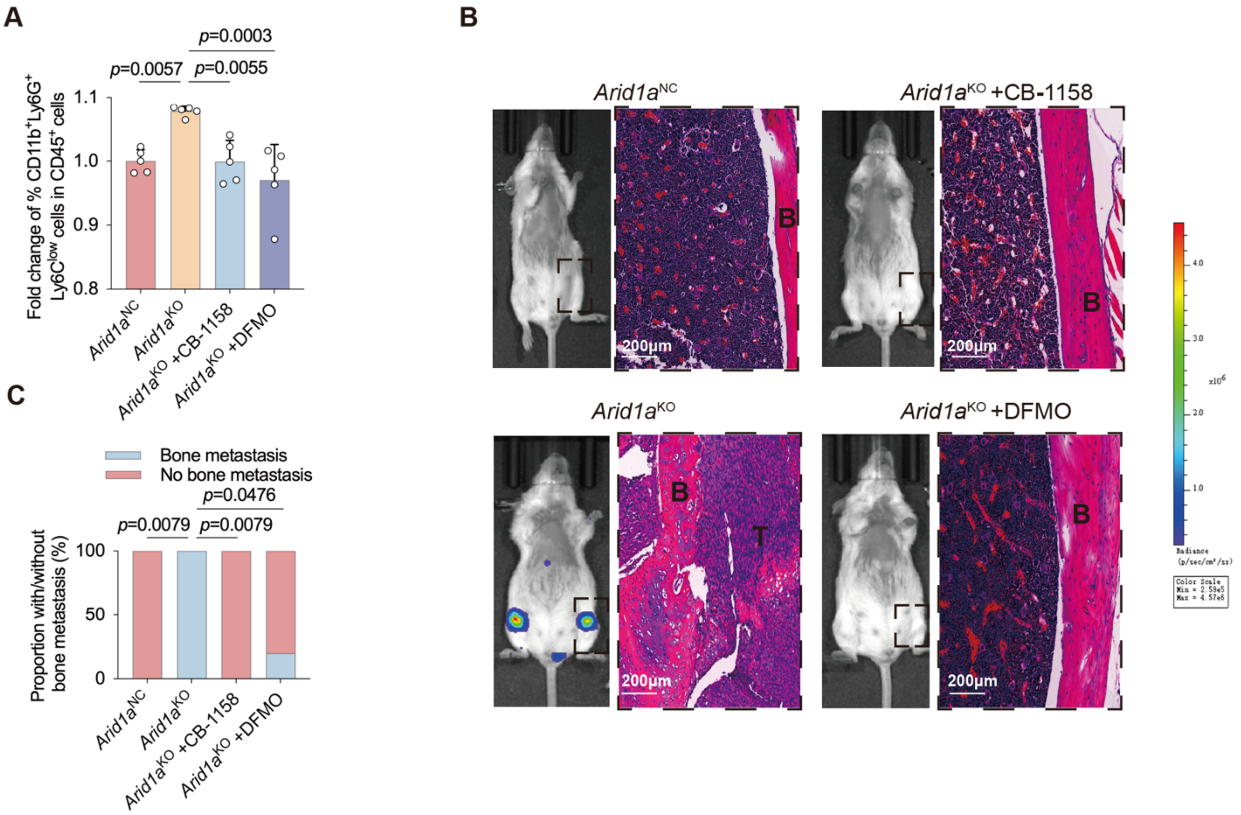

Supplementary Fig. 13 Systemic assessment of CB-1158 and DFMO treatment in mice. (**A**) Bone marrow of mice was harvested from *Arid1a*^{NC}, *Arid1a*^{KO}, *Arid1a*^{KO} plus CB-1158 and *Arid1a*^{KO} plus DFMO groups with intracardiac injection, and analyzed by flow cytometry to quantify PMN-MDSC proportions. Data are presented as mean ± SD (n=5 mice per group), one-way ANOVA for multiple comparisons. (**B**) Bioluminescence imaging (BLI) and H&E staining images of mice in *Arid1a*^{NC}, *Arid1a*^{KO}, *Arid1a*^{KO} plus CB-1158 and *Arid1a*^{KO} plus DFMO groups with intracardiac injection. n=5 mice per group. (**C**) Statistical charts presenting the bone metastasis incidence in *Arid1a*^{NC}, *Arid1a*^{KO}, *Arid1a*^{KO} plus CB-1158 and *Arid1a*^{KO} plus DFMO groups with intracardiac injection. n=5 mice per group, two-sided Fisher's exact test was used. Source data are provided as a Source Data file.

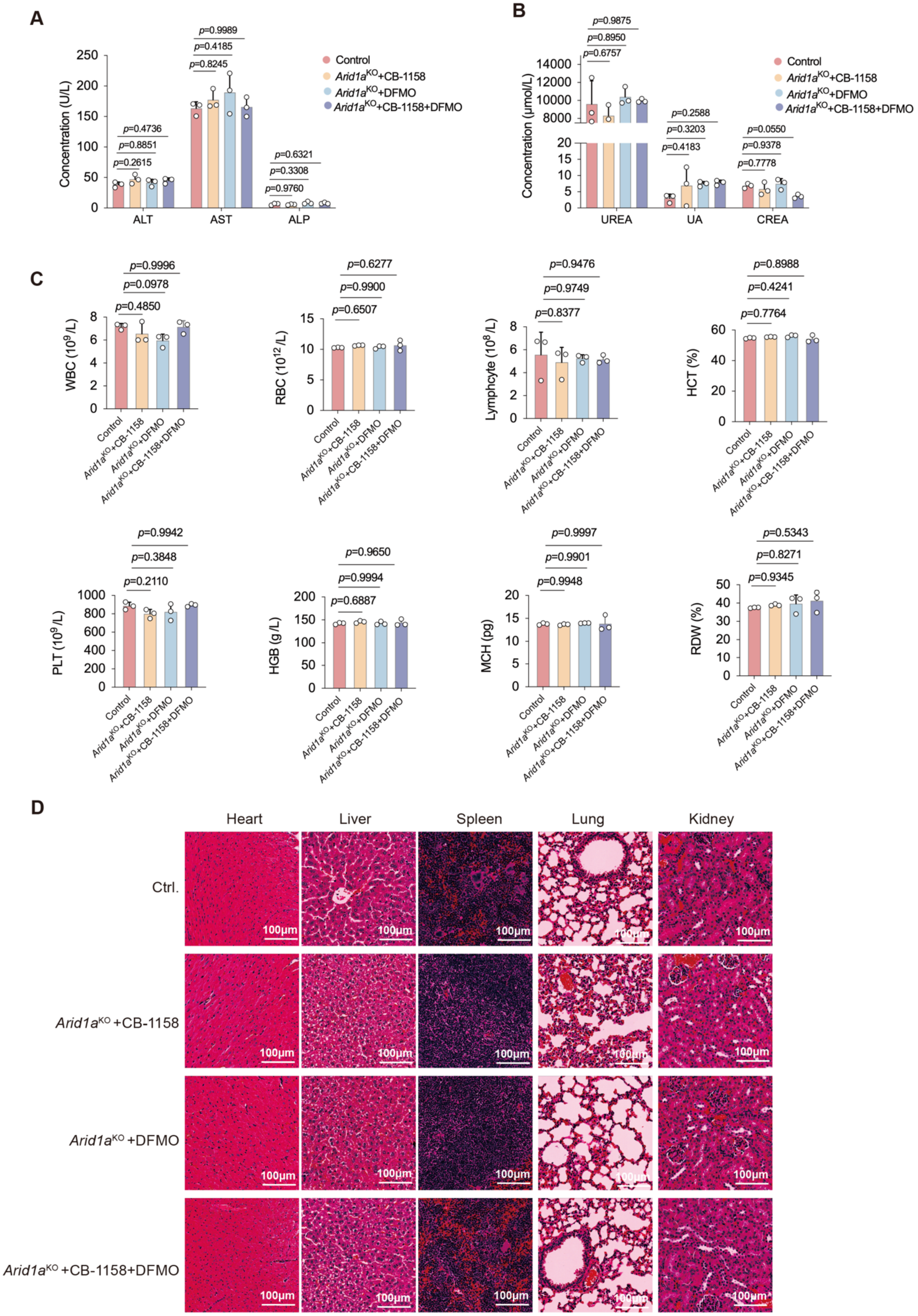
A
Concentration (U/L)
ALT
AST
ALP
p=0.4736
p=0.8851
p=0.2615
p=0.9989
p=0.4185
p=0.8245
p=0.6321
p=0.3308
p=0.9760
Control
Arid1a^KO+CB-1158
Arid1a^KO+DFMO
Arid1a^KO+CB-1158+DFMO
B
Concentration (μmol/L)
UREA
UA
CREA
p=0.9875
p=0.8950
p=0.6757
p=0.2588
p=0.3203
p=0.4183
p=0.0550
p=0.9378
p=0.7778
C
WBC (10^9/L)
p=0.9996
p=0.0978
p=0.4850
RBC (10^12/L)
p=0.6277
p=0.9900
p=0.6507
Lymphcyte (10^8/L)
p=0.9476
p=0.9749
p=0.8377
HCT (%)
p=0.8988
p=0.4241
p=0.7764
PLT (10^9/L)
p=0.9942
p=0.3848
p=0.2110
HGB (g/L)
p=0.9650
p=0.9994
p=0.6887
MCH (pg)
p=0.9997
p=0.9901
p=0.9948
RDW (%)
p=0.5343
p=0.8271
p=0.9345
D
Heart
Liver
Spleen
Lung
Kidney
Ctrl.
Arid1a^KO +CB-1158
Arid1a^KO +DFMO
Arid1a^KO +CB-1158+DFMO
100μm

Supplementary Fig. 14 In vivo safety assessment of CB-1158, DFMO, and their combination treatment. (**A-B**) Analysis of alanine transaminase (ALT), aspartate transaminase (AST), alkaline phosphatase (ALP) for liver function, and urea (UREA), uric acid (UA), and creatinine (CREA) for kidney function in peripheral blood from mice in the control, CB-1158, DFMO and combined groups. Data are presented as mean ± SD (n=3 mice per group), one-way ANOVA for multiple comparisons. (**C**) Routine blood tests of peripheral blood from mice in the control, CB-1158, DFMO and combined group. Data are presented as mean ± SD (n=3 mice per group), one-way ANOVA for multiple comparisons. WBC, white blood cell; RBC, red blood cell; HCT, hematocrit; PLT, platelet; HGB, hemoglobin; MCH, mean corpuscular hemoglobin; RDW, red cell distribution width. (**D**) Representative H&E staining images of heart, liver, spleen, lung, and kidney tissues from mice in the control, CB-1158, DFMO and combined groups. Scale bar, 100μm. The experiment was repeated independently three times. All *p* values are indicated in the figures. Source data are provided as a Source Data file.

Arginine

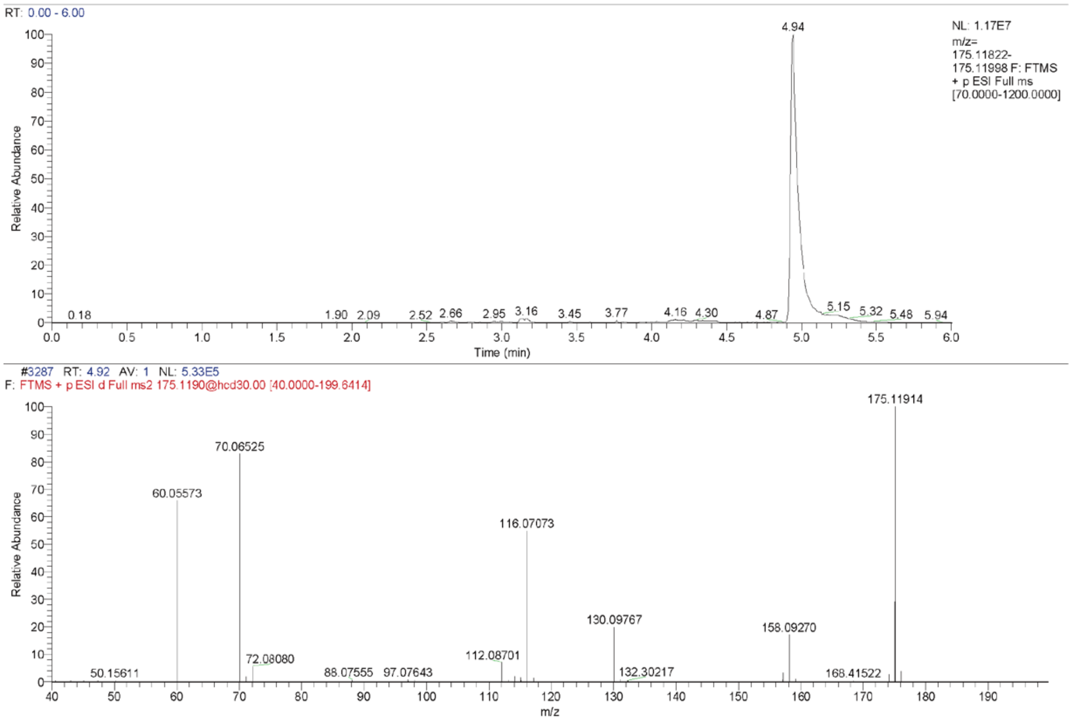

Glutamate

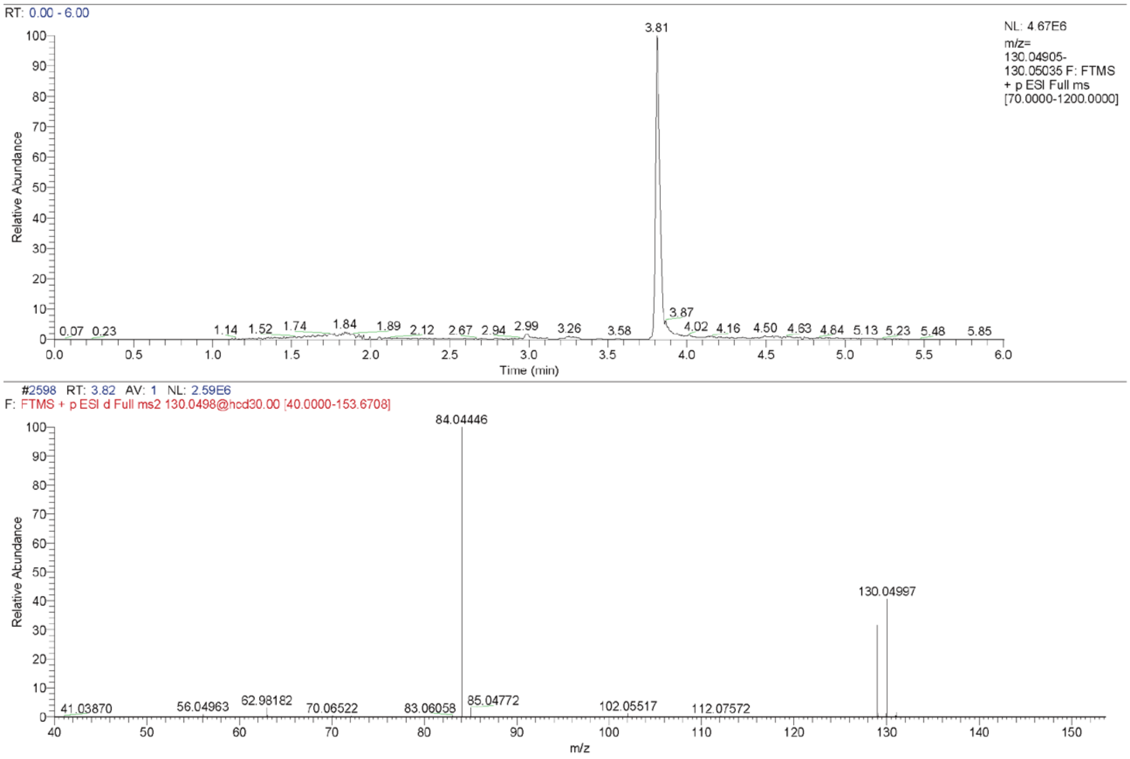

Supplementary Fig. 15 Extracted Ion Chromatograms and MS/MS spectra of the identified metabolites. Extracted Ion Chromatograms and MS/MS spectra of arginine and glutamate.

4-Guanidinobutyric acid

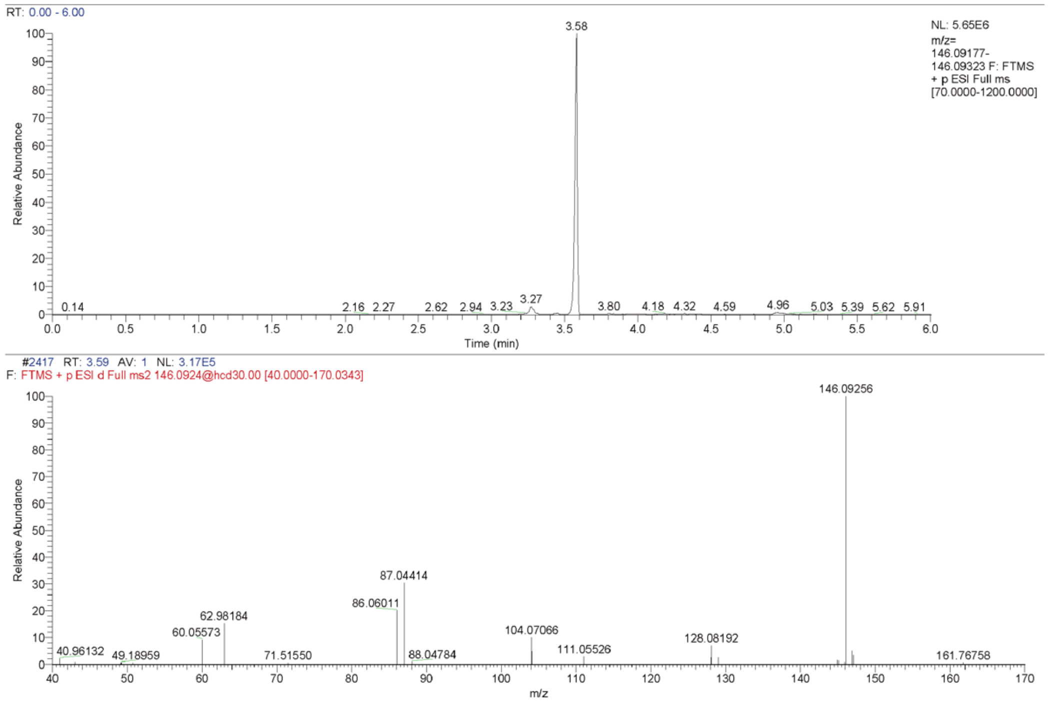

5-Aminopentanoic acid

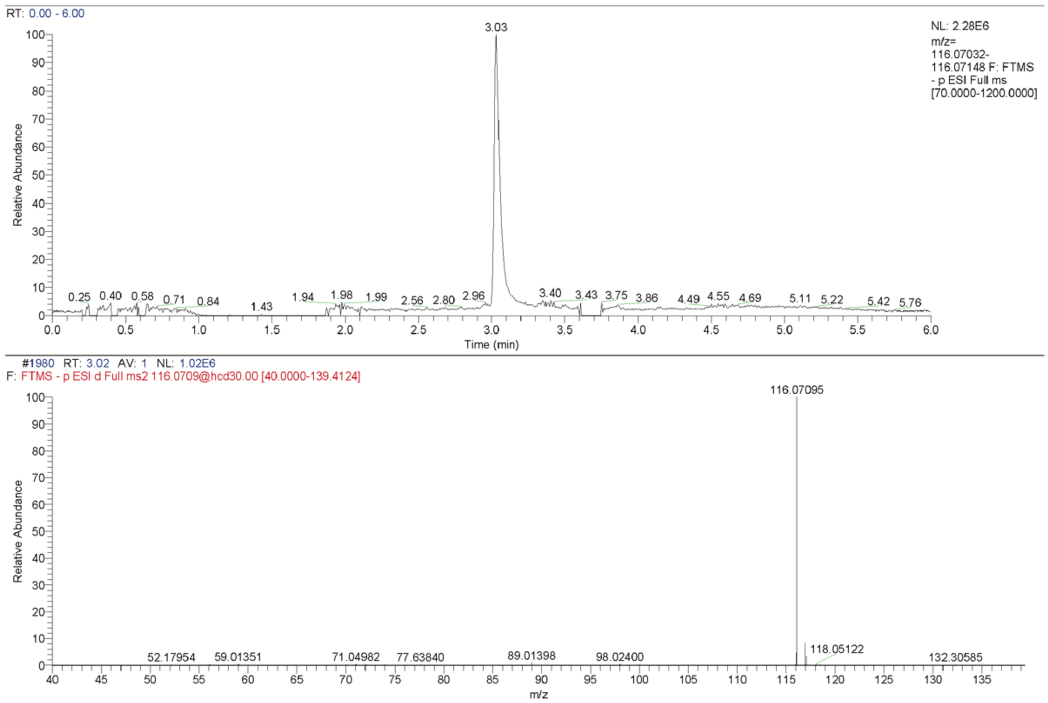

Supplementary Fig. 16 Extracted Ion Chromatograms and MS/MS spectra of the identified metabolites. Extracted Ion Chromatograms and MS/MS spectra of 4-Guanidinobutyric acid and 5-Aminopentanoic acid.

Creatinine

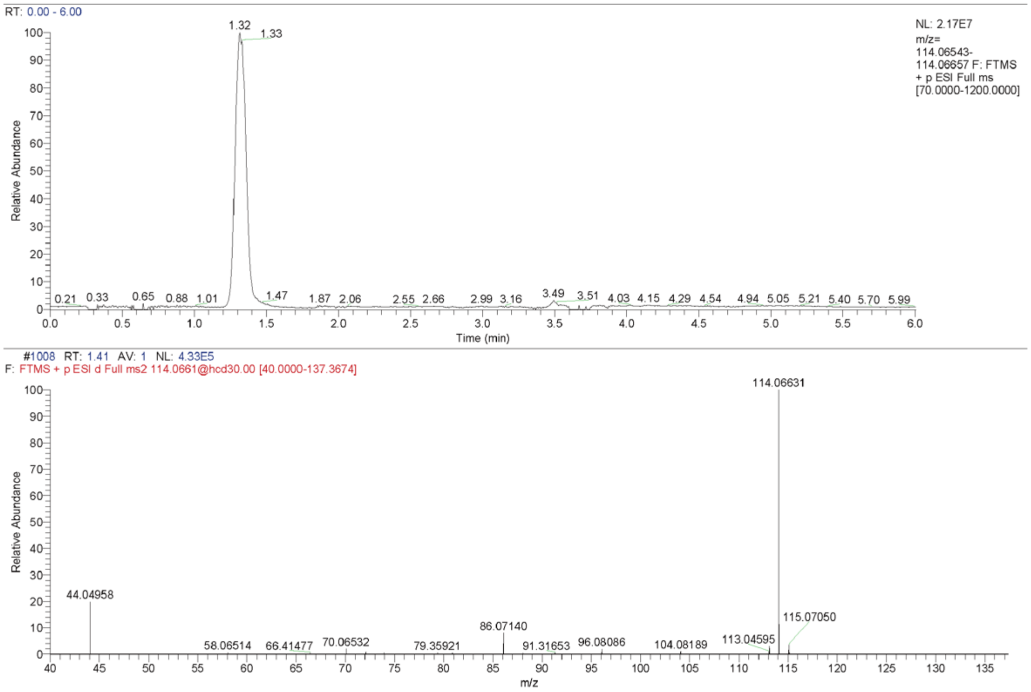

Pyruvate

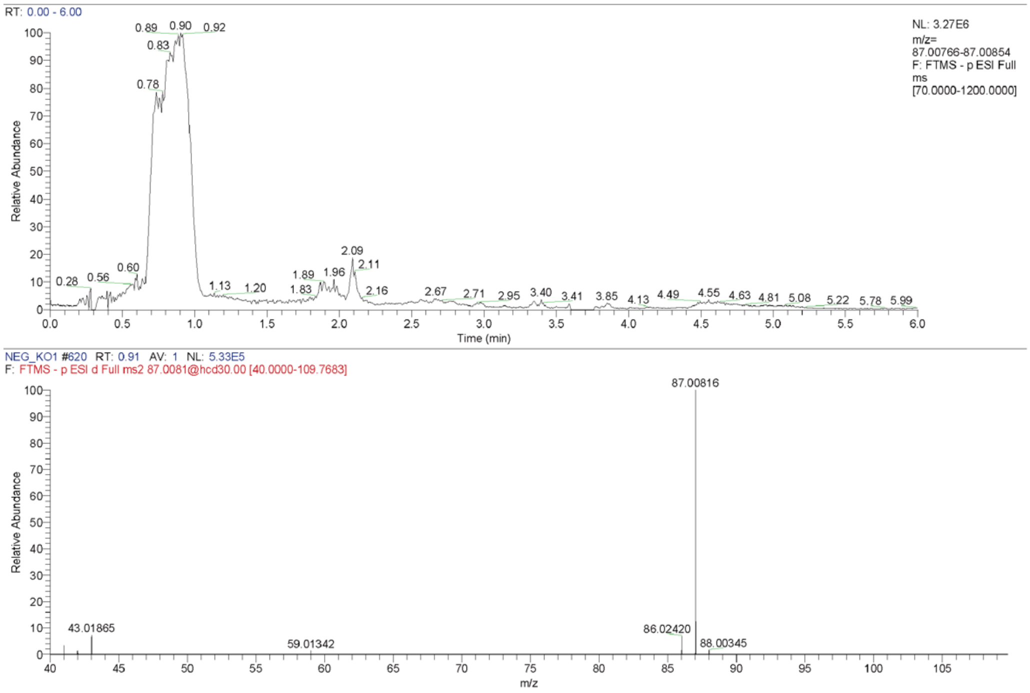

Supplementary Fig. 17 Extracted Ion Chromatograms and MS/MS spectra of the identified metabolites. Extracted Ion Chromatograms and MS/MS spectra of creatinine and pyruvate.

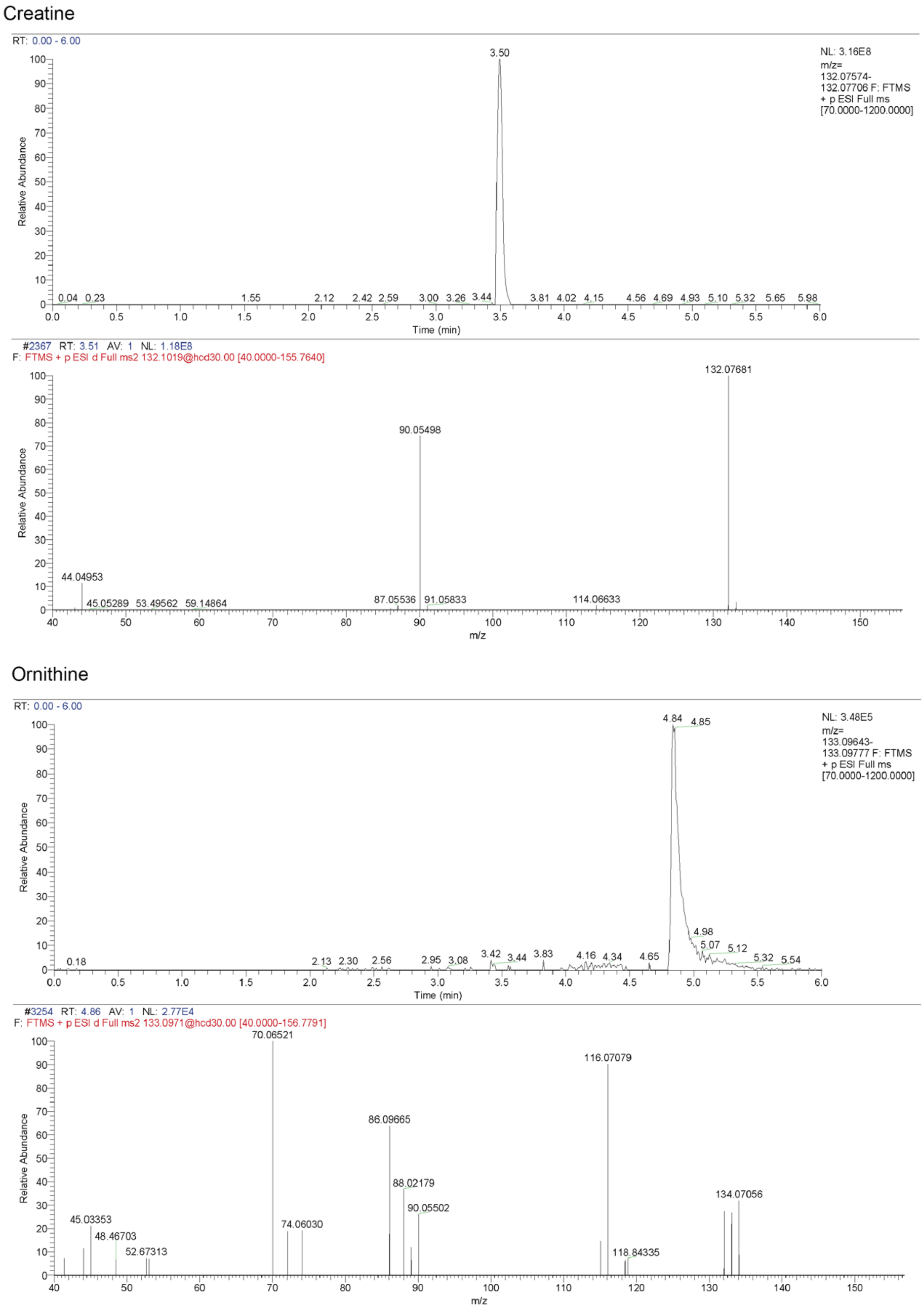

Supplementary Fig. 18 Extracted Ion Chromatograms and MS/MS spectra of the identified metabolites. Extracted Ion Chromatograms and MS/MS spectra of creatine and ornithine.

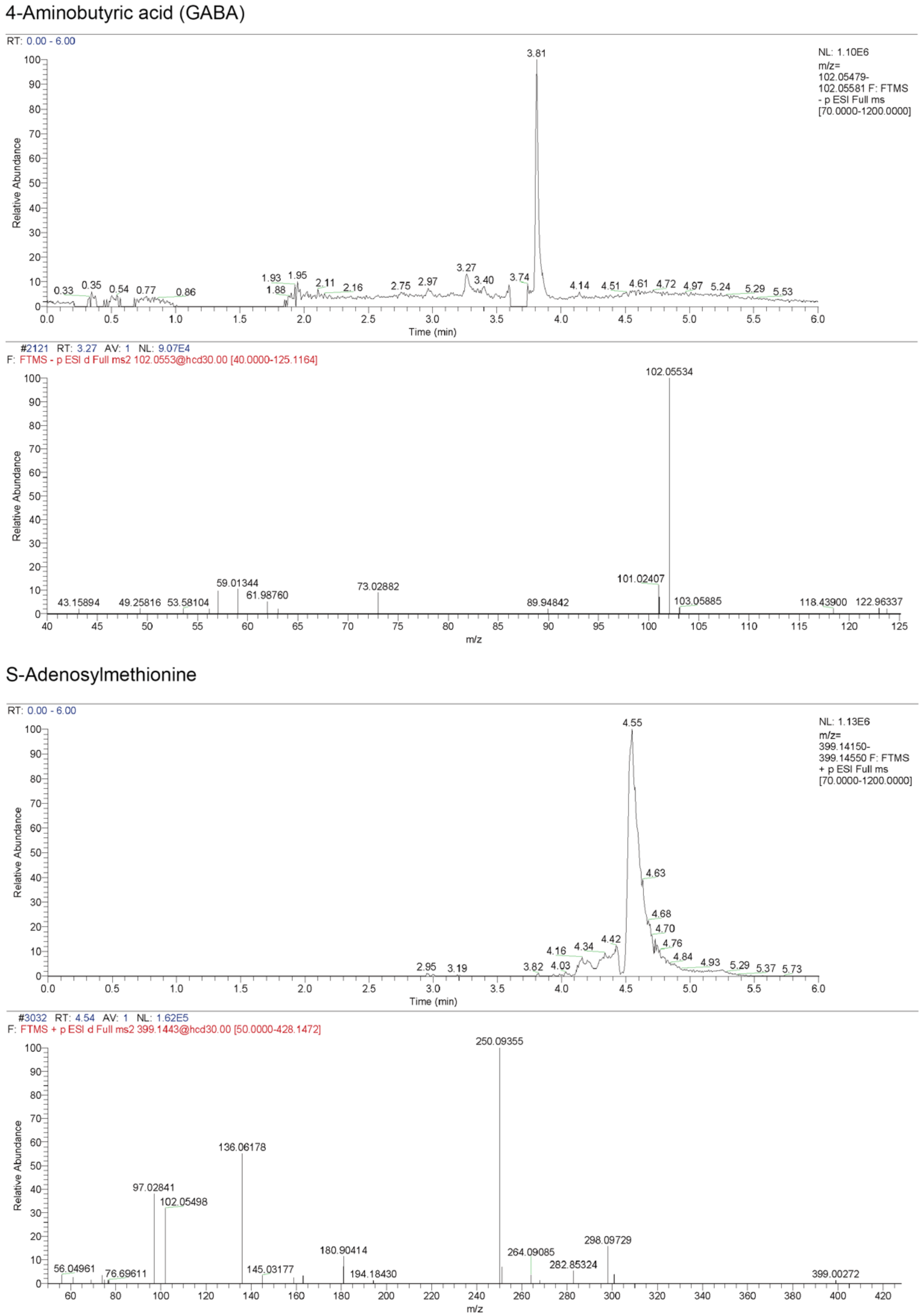

Supplementary Fig. 19 Extracted Ion Chromatograms and MS/MS spectra of the identified metabolites. Extracted Ion Chromatograms and MS/MS spectra of 4-Aminobutyric acid (GABA) and S-Adenosylmethionine.

Supplementary Table 1. Metastatic sites distribution by *ARID1A* mutation status in breast cancer

Characteristic	Total [cases (%)]	*ARID1A*-mut[a] [cases (%)]	*ARID1A*-wt[b] [cases (%)]	p^c
Total	663 (100)	53 (8.0)	610 (92.0)	
Bone metastasis				0.0081
Negative	353 (53.2)	19 (35.8)	334 (54.8)	
Positive	310 (46.8)	34 (64.2)	276 (45.2)	
Age at diagnosis				0.8294
18-34 years	107 (16.1)	8 (15.1)	99 (16.2)	
≥35years	556 (83.9)	45 (84.9)	511 (83.8)	
Menstruation				0.8214
Pre-menopause	359 (54.1)	28 (52.8)	331 (54.3)	
Menopause	302 (45.6)	25 (47.2)	277 (45.4)	
NA[d]	2 (0.3)	0 (0.0)	2 (0.3)	
Family history				0.3740
No	532 (80.2)	45 (84.9)	487 (79.8)	
Yes	131 (19.8)	8 (15.1)	123 (20.2)	
Liver metastasis				0.0803
No	388 (58.5)	25 (47.2)	363 (59.5)	
Yes	275 (41.5)	28 (52.8)	247 (40.5)	
Lymph node metastasis				0.5685
No	300 (45.2)	22 (41.5)	278 (45.6)	
Yes	363 (54.8)	31 (58.5)	332 (54.4)	
Lung metastasis				0.9222
No	346 (52.2)	28 (52.8)	318 (52.1)	
Yes	317 (47.8)	25 (47.2)	292 (47.9)	
Brain metastasis				0.6910
No	603 (91.0)	49 (92.5)	554 (90.8)	
Yes	60 (9.0)	4 (7.5)	56 (9.2)	

[a]*ARID1A*-mut, *ARID1A* mutations.

[b]*ARID1A*-wt, *ARID1A* wild-type.

[c]Differences between *ARID1A*-mut and *ARID1A*-wt were compared using the two-sided Pearson's chi-square test.

[d]NA indicates that the data is not available and was not included in the *p* value calculation.

Supplementary Table 2. Mutated SWI/SNF subunits in the ctDNA cohort.

Mutated SWI/SNF subunits (Total=663)	Bone metastasis	No bone metastasis	*p* value[a]
ARID1A (n=53)	34	19	0.0081
ARID1B (n=14)	6	8	0.7675
ARID2 (n=14)	7	7	0.8058
SMARCA4 (n=20)	11	9	0.4531
SMARCB1 (n=3)	3	0	0.0640
BRD7 (n=2)	2	0	0.1307

[a] *p* values were determined using the two-sided Pearson's chi-square test.

Supplementary Table 3. Bone metastasis incidence by *ARID1A* status across breast cancer subtype

Characteristic	Total [cases (%)]	*ARID1A* mut [cases (%)]	*ARID1A* wt [cases (%)]	p^a
Total	663 (100)	53 (8.0)	610 (92.0)	
Bone metastasis				
Triple negative	239 (36.0)	15 (6.3)	224 (93.7)	0.0337
Negative	156 (65.3)	6 (40.0)	150 (67.0)	
Positive	83 (34.7)	9 (60.0)	74 (33.0)	
HER-2 positive	114 (17.2)	3 (2.6)	111 (97.4)	0.2427
Negative	79 (69.3)	3 (100.0)	76 (68.5)	
Positive	35 (30.7)	0 (0.00)	35 (31.5)	
Luminal A	141 (21.3)	11 (7.8)	130 (92.2)	0.0554
Negative	52 (36.9)	7 (63.6)	45 (34.6)	
Positive	89 (63.1)	4 (36.4)	85 (65.4)	
Luminal B	157 (23.7)	21 (9.8)	136 (10.5)	0.1012
Negative	63 (40.1)	5 (23.8)	58 (42.6)	
Positive	94 (59.9)	16 (76.2)	78 (57.4)	

[a]Differences between *ARID1A*-mut and *ARID1A*-wt were compared using the two-sided Pearson's chi-square test.

[b]Breast cancer subtype was not available in 12 patients.

Supplementary Table 4. Characteristics of 108 TNBC patients in the FUSCC cohort

Characteristic	Total [cases (%)]	ARID1A-high [cases (%)]	ARID1A-low [cases (%)]	p^a
Total	108(100)	51 (47.2)	57(52.8)	
Age at diagnosis				
18-34 years	5(4.6)	2(3.9)	3(5.3)	0.7405
≥35 years	103(95.4)	49(96.1)	54(94.7)	
Ki67 expression				
Low (≤20%)	8(7.4)	3(5.9)	5(8.8)	0.5670
High (>20%)	100(92.6)	48(94.1)	52(91.2)	
Histological grade				
I	0(0)	0(0)	0(0)	0.3030
II	19(17.6)	7(13.7)	12(21.1)	
III	78(72.2)	39(76.5)	39(68.4)	
NA[b]	11(10.2)	5(9.8)	6(10.5)	
Vascular invasion				
Negative	65(60.2)	31(60.8)	34(59.6)	0.8921
Positive	41(38.0)	19(37.3)	22(38.6)	
NA	2 (1.8)	1(1.9)	1 (1.8)	

[a]Differences between ARID1A-high and ARID1A-low were compared using the two-sided Pearson's chi-square test.

[b]NA indicates that the data is not available and was not included in the *p* value calculation.

Supplementary Table 5. Characteristics of 40 TNBC patients in the FUSCC

Characteristic	Total [cases (%)]	High ornithine [cases (%)]	Low ornithine [cases (%)]	p^a
Total	40(100)	20 (50.0)	20(50.0)	
Age at diagnosis				
18-34 years	4(10.0)	0(0)	0(0)	/
≥35 years	36(90.0)	20(100)	20(100)	
Ki67 expression				
Low (≤20%)	0(0)	0(0)	0(0)	/
High (>20%)	40(100)	20(100)	20(100)	
Histological grade				
I	8(20.0)	2(10.0)	6(30.0)	0.1697
II	13(32.5)	6(30.0)	7(35.0)	
III	17(42.5)	11(55.0)	6(30.0)	
NA[b]	2(5.0)	1(5.0)	1(5.0)	
Vascular invasion				
Negative	19(47.5)	9(45.0)	10(50.0)	0.8728
Positive	18(45.0)	9(45.0)	9(45.0)	
NA[b]	3 (7.5)	2(10.0)	1 (5.0)	

[a]Differences between high ornithine and low ornithine were compared using the two-sided Pearson's chi-square test.

[b]NA indicates that the data is not available and was not included in the *p* value calculation.

Supplementary Table 6. Characteristics of 40 TNBC patients in the FUSCC

Characteristic	Total [cases (%)]	High spermine [cases (%)]	Low spermine [cases (%)]	p^{a}
Total	40(100)	20 (50.0)	20(50.0)	
Age at diagnosis				
18-34 years	4(10.0)	0(0)	0(0)	/
≥35 years	36(90.0)	20(100)	20(100)	
Ki67 expression				
Low (≤20%)	0(0)	0(0)	0(0)	/
High (>20%)	40(100)	20(100)	20(100)	
Histological grade				
I	8(20.0)	2(10.0)	6(30.0)	0.1697
II	13(32.5)	6(30.0)	7(35.0)	
III	17(42.5)	11(55.0)	6(30.0)	
NA[b]	2(5.0)	1(5.0)	1(5.0)	
Vascular invasion				
Negative	19(47.5)	8(40.0)	11(55.0)	0.4133
Positive	18(45.0)	10(50.0)	8(40.0)	
NA[b]	3 (7.5)	2(10.0)	1 (5.0)	

[a]Differences between high spermine and low spermine were compared using the two-sided Pearson's chi-square test.

[b]NA indicates that the data is not available and was not included in the *p* value calculation.

Supplementary Table 7. Primer sequences for plasmid construction

Target	Sequence (5'→3')
M-Sg*Arid1a*-KO1	GGTCCCTGTTGTTGCGAGTA
M-Sg*Arid1a*-KO2	ACCCCATGACCATGCAGGGC
Hu-Sg*ARID1A*-KO1	ATGGTCATCGGGTACCGCTG
Hu-Sg*ARID1A*-KO2	CCCCTCAATGACCTCCAGTA
M-Sg*Arid1a*-OE1	TCCAGACGGAAATCCACGCC
M-Sg*Arid1a*-OE2	AACAAAAAGAGCGTGAAAGA
Hu- Sg*ARID1A*-OE1	GGCGCTCTAGCCGCTCAGTC
Hu- Sg*ARID1A*-OE1	CTTGGGTCGAGGCTGCTGCG
Hu-Si*ARID3A*-1	GCAUGUCGGUGGAGAUCAATT
Hu-Si*ARID3A*-2	GAUGACUUGUUCAGCUUCATT
Hu-Si*ARID3A*-3	CCUCCGACGAGGACAUGAATT
M-Sg*Arg2*-KO1	GTGCCGGGCGTGACCGATAA
M-Sg*Arg2*-KO2	CCAGCTGCCATTCGAGAAGC
M-Sg*Odc1*-KO1	GCGTTCTATGTTGCGGACCT
M-Sg*Odc1*-KO2	GCGTAAAAGGGAGTGACGCG

Supplementary Table 8. Metabolite identification summary table

MS2 name[a]	MS2 score	Level	mz[b]	rt [c](s)	Type	rt(min)
Arginine	3.85	Level 1	175.1191	296.9	POS	4.9483
Glutamate	3.86	Level 1	130.0497	229.1000	POS	3.8183
4-Guanidinobutyric acid	3.97	Level 1	146.0925	214.7000	POS	3.5783
5-Aminopentanoic acid	3.63	Level 1	116.0709	182.7000	NEG	3.0450
Creatinine	3.76	Level 1	114.0660	80.3000	POS	1.3383
Pyruvate	3.73	Level 1	87.0081	54.7000	NEG	0.9117
Creatine	3.91	Level 1	132.0764	209.9000	POS	3.4983
Ornithine	3.92	Level 1	133.0971	291.1000	POS	4.8517
4-Aminobutyric acid (GABA)	3.72	Level 1	102.0553	196.4	NEG	3.2733
S-Adenosylmethionine	3.73	Level 1	399.1435	273.1000	POS	4.5517

[a]MS2 name, MS/MS name

[b]mz, mass-to-charge ratio

[c]rt, retention time

Supplementary Table 9. Primer sequence information for RT-qPCR

Species	Orientation	Gene	Sequence (5' to 3')
Mouse	Forward	*Arid1a*	GCCACAAACTCCTCAGTCAACC
Mouse	Reverse	*Arid1a*	GCATCCTGGATTCCGACTGAGT
Mouse	Forward	*Arg2*	CACCTCTCACCACTGTATCTGG
Mouse	Reverse	*Arg2*	CCAGGAAAATCCTGGCAGTTGTG
Mouse	Forward	*Odc1*	TGCCACACTCAAAACCAGCAGG
Mouse	Reverse	*Odc1*	ACACTGCCTGAACGAAGGTCTC
Mouse	Forward	*Oat*	CCGACCAGTTATGATGGCTTTGG
Mouse	Reverse	*Oat*	CTCCACCATGAAGGCAGCAACA
Mouse	Forward	*Malat1*	AGCAGGCATTGTGGAGAGGA
Mouse	Reverse	*Malat1*	ATGTTGCCGACCTCAAGGAA
Mouse	Forward	*Arid3a*	TCCATCACCAGTGCTGCCTTCA
Mouse	Reverse	*Arid3a*	TCCCTGCGATTGCTGTCTATGG
Human	Forward	*ARID1A*	AAGCCACCAACTCCAGCATCCA
Human	Reverse	*ARID1A*	CGCTTCTGGAATGTGGAGTCAC
Human	Forward	*ARG2*	CTGGCTTGATGAAAAGGCTCTCC
Human	Reverse	*ARG2*	TGAGCGTGGATTCACTATCAGGT
Human	Forward	*ODC1*	CCAAAGCAGTCTGTCGTCTCAG
Human	Reverse	*ODC1*	CAGAGATTGCCTGCACGAAGGT
Human	Forward	*OAT*	CGTTGTCTGCTATCTCCAGTTCC
Human	Reverse	*OAT*	CCATGAACGCAGCCACATTTGG
Human	Forward	*MALAT1*	AATGTTAAGAGAAGCCCAGGG
Human	Reverse	*MALAT1*	AAGGTCAAGAGAAGTGTCAGC
Human	Forward	*ARID3A*	TCCATCACCAGTGCAGCCTTCA
Human	Reverse	*ARID3A*	TCTATGGCTGCCTGGAGCTCAT

Supplementary Table 10. Primer sequence information for CHIP-qPCR

Species	Orientation	Sequence (5' to 3')
Human	Forward	GATGGTGAGGCTTCAGTTTAT
Human	Reverse	TATGAGATTCTATTTCAGACTGCT

Supplementary Table 11. Primer sequence information for CHIP-qPCR

ARG2 enhancer wildtype
TTTAATTGTACAATTATATCCTTACTGTGTAGCTGACTGGTAGCTCAGTATCACCA<br>GCAGTCTGAAATAGAATCTCATAGCCTATTAAATAAAACTGTGGCTCTCAGCAGA<br>TTTCTAGCATGTGGTTTTGGCTAATCTCCTGGAATATATTGGAGAAAAGTACCAAG<br>AGACATTTCAGTTGCTCTGTTCCTAAACAGTACCAAAATAACTTGAGTGTTTTGAA<br>CACTTTCAAATAAACAGCATGGATCAAATTTCAAAGGAGAGACTCATGGAAAGGT<br>AACTTTCACAATCCCTTTGTTCCTTGCCTTTGCTCTGATGTCACAGGCTGCCGGTGT<br>TACCATAGCATGAGTTAAATTTCCTGTTTTCTCTCGAATGAATGTATTTGACTTAGT<br>GTCTGGGCTTGTTTTATTTCCTTGGGCTTAGTAAGGACACTCAACAGTACTCTCAG<br>GTAACCTTTTTTTTTTTTTTTTTTTTTTTTTTGAGACGGAGTCTCGCTCTGTCACCAG<br>GCTGGAGTACAGTGATGCGATCTTGGCTCACTGCAACCTCCGCCTCCCGGGTTCAA<br>GCAATTCTCCTGCCTCAACCTCCAAAGTAGCTGGGATTACAGGTGCCTGCCACTGC<br>GCCTGACTAATTTTTGTATTTTTAGTAGAGATGGGGTTTCACCATGTTGGCCAGGC<br>TGATCTCGAACTCCTGACCTCAGGTGATCTGCCCATCTAGGCCTCCCAAAGTGCTG<br>GGATTACAAGAGTGAGCCACTGCTCCCAGCCACCTTTTATTTTTCTTTGAGAGAGG<br>GTCTCACTCTGTCACTCAGGCTGGAGTGCAGTGGCATAGTCACAGCCTACTGCAAT<br>CTCAACCTCCTGGACTCAAGCGATCCTCCCACTTTAGCCTCTTGAGTAGCTGGGAC<br>TACAGACACATGCCAGTATGCCTGGATAATTTTTTTTTCTTTTGTAGAGCCGGGGT<br>CTCACTATGTTGCCCAGGCTGGTCCCGAACTCCTGGGCTTAAGAGGGCCTAAGAG<br>AAATGAAAAGGCATTGCTCTGGCAAGGCTGAGGAGCAGTATATCTGCACTCGATT<br>TTGGTTAGCTCTGAGAACCATGGTAATTTGTGAATTCTAGCCACCTGTTACCATGT<br>GAGAAGGAGTGGGCCTTGTACTCTTTTCTCCAGTTTTTCAAGAAAAGCCAGAAATT<br>TGCACTTTAAT
ARG2 enhancer mutant
TTTAATTGTACAATTATATCCTTACTGTGTAGCTGACTGGTAGCTCAGTATCACCA<br>GCAGTCTGAAATAGAATCTCATAGCCTGCCGGGTAAAACTGTGGCTCTCAGCAGA<br>TTTCTAGCATGTGGTTTTGGCTAATCTCCTGGAATATATTGGAGAAAAGTACCAAG<br>AGACATTTCAGTTGCTCTGTTCCTAAACAGTACCAAAATAACTTGAGTGTTTTGAA<br>CACTTTCAAATAAACAGCATGGATCAAATTTCAAAGGAGAGACTCATGGAAAGGT

AACTTTCACAATCCCTTTGTTCCTTGCCTTTGCTCTGATGTCACAGGCTGCCGGTGT
TACCATAGCATGAGTTAAATTTCCTGTTTTCTCTCGAATGAATGTATTTGACTTAGT
GTCTGGGCTTGTTTTATTTCCTTGGGCTTAGTAAGGACACTCAACAGTACTCTCAG
GTAACCTTTTTTTTTTTTTTTTTTTTTTTTTTGAGACGGAGTCTCGCTCTGTCACCAG
GCTGGAGTACAGTGATGCGATCTTGGCTCACTGCAACCTCCGCCTCCCGGGTTCAA
GCAATTCTCCTGCCTCAACCTCCAAAGTAGCTGGGATTACAGGTGCCTGCCACTGC
GCCTGACTAATTTTTGTATTTTTAGTAGAGATGGGGTTTCACCATGTTGGCCAGGC
TGATCTCGAACTCCTGACCTCAGGTGATCTGCCCATCTAGGCCTCCCAAAGTGCTG
GGATTACAAGAGTGAGCCACTGCTCCCAGCCACCTTTTATTTTTCTTTGAGAGAGG
GTCTCACTCTGTCACTCAGGCTGGAGTGCAGTGGCATAGTCACAGCCTACTGCAAT
CTCAACCTCCTGGACTCAAGCGATCCTCCCACTTTAGCCTCTTGAGTAGCTGGGAC
TACAGACACATGCCAGTATGCCTGGATAATTTTTTTTCTTTTGTAGAGCCGGGGT
CTCACTATGTTGCCCAGGCTGGTCCCGAACTCCTGGGCTTAAGAGGGCCTAAGAG
AAATGAAAAGGCATTGCTCTGGCAAGGCTGAGGAGCAGTATATCTGCACTCGATT
TTGGTTAGCTCTGAGAACCATGGTAATTTGTGAATTCTAGCCACCTGTTACCATGT
GAGAAGGAGTGGGCCTTGTACTCTTTTCTCCAGTTTTTCAAGAAAAGCCAGAAATT
TGCACTTTAAT

Supplementary Table 12. The antibody information used in this study

Antibodies	Dilution	Catalog	Manufacturer
**For flow cytometry staining**			
Anti-human CD45	1:100	abs1840506	Absin
Anti-human CD11b	1:200	550993	BD Pharmingen
Anti-human CD14	1:100	abs1840145	Absin
Anti-human CD66b	1:200	305116	BD Pharmingen
Anti-human HLA-DR	1:200	562804	BD Pharmingen
Anti-mouse CD45	1:200	557659	BD Pharmingen
Anti-mouse CD11b	1:200	557396	BD Pharmingen
Anti-mouse Ly6C	1:200	560592	BD Pharmingen
Anti-mouse Ly6G	1:200	560599	BD Pharmingen
**For western blotting**			
Anti-ARG2 Antibody	1:300	Bs-11397R-Bio	Bioss
Anti-ODC1 Antibody	1:1000	Ab97395	Abcam
Anti-OAT Antibody	1:1000	Ab137679	Abcam
Anti-ARID1A Antibody	1:1000	12354S	CST[a]
Anti-rabbit IgG, HRP-linked Antibody	1:3000	7074	CST
Anti-mouse IgG, HRP-linked Antibody	1:3000	7076	CST
GAPDH	1:3000	10494-1-AP	Proteintech
**For immunohistochemistry**			
Anti-ARG2 Antibody	1:200	Bs-11397R-Bio	Bioss
Anti-ODC1 Antibody	1:200	Ab97395	Abcam
Anti-OAT Antibody	1:200	Ab137679	Abcam
Anti-ARID1A Antibody	1:500	12354S	CST
**For immunofluorescence**			

Anti-mouse CD11b	1:100	17800	CST
Anti-mouse Ly6G	1:200	88876	CST
Anti-human CD11b	1:2000	ab133357	Abcam
Anti-human CD66b	1:200	ab197678	Abcam
Anti-human CD14	1:800	ab133335	Abcam
Anti-human HLA-DR	1:200	ab92511	Abcam
Anti-human LOX-1	1:200	SC-373995	Santa Cruz
Goat anti-rabbit IgG H&L (HRP)	1:2000	ab205718	Abcam
**For PMN-MDSC deletion**			
Anti-mouse Ly6G	10mg/kg	BE0075-1	BioXCell
Mouse IgG	10mg/kg	I8765	Sigma

[a]CST, Cell Signaling Technology
