# Supplementary material for: Targeting arginine metabolism reverses bone immunosuppressive microenvironment and metastasis in ARID1A-deficient triple negative breast cancer
Source: Nat Commun. 2026 May 26;17:6863. doi: 10.1038/s41467-026-73574-3 (PMC13389217; doi:10.1038/s41467-026-73574-3)
Supplement: Supplementary file 2 — Reporting Summary [file 41467_2026_73574_MOESM2_ESM.pdf]

nature portfolio

Corresponding author(s): Xichun Hu
Zhonghua Tao
Mengdi Yang

Last updated by author(s): Mar 29, 2026

Reporting Summary

Nature Portfolio wishes to improve the reproducibility of the work that we publish. This form provides structure for consistency and transparency in reporting. For further information on Nature Portfolio policies, see our Editorial Policies and the Editorial Policy Checklist.

Statistics

For all statistical analyses, confirm that the following items are present in the figure legend, table legend, main text, or Methods section.

n/a	Confirmed	
☐	☒	The exact sample size (*n*) for each experimental group/condition, given as a discrete number and unit of measurement
☐	☒	A statement on whether measurements were taken from distinct samples or whether the same sample was measured repeatedly
☐	☒	The statistical test(s) used AND whether they are one- or two-sided<br>*Only common tests should be described solely by name; describe more complex techniques in the Methods section.*
☒	☐	A description of all covariates tested
☐	☒	A description of any assumptions or corrections, such as tests of normality and adjustment for multiple comparisons
☐	☒	A full description of the statistical parameters including central tendency (e.g. means) or other basic estimates (e.g. regression coefficient) AND variation (e.g. standard deviation) or associated estimates of uncertainty (e.g. confidence intervals)
☐	☒	For null hypothesis testing, the test statistic (e.g. *F*, *t*, *r*) with confidence intervals, effect sizes, degrees of freedom and *P* value noted<br>*Give P values as exact values whenever suitable.*
☒	☐	For Bayesian analysis, information on the choice of priors and Markov chain Monte Carlo settings
☒	☐	For hierarchical and complex designs, identification of the appropriate level for tests and full reporting of outcomes
☐	☒	Estimates of effect sizes (e.g. Cohen's *d*, Pearson's *r*), indicating how they were calculated

Our web collection on statistics for biologists contains articles on many of the points above.

Software and code

Policy information about availability of computer code

Data collection	The CHIP-Seq and ATAC-Seq data were downloaded from the GEO under accession code GSE234179.
Data analysis	The code used to analyse the dataset is openly available at GitHub (https://github.com/shuangyuePan/Bone-marrow-ScRNA-Seq) and the https://doi.org/10.5281/zenodo.19248693.<br>Samples were compared statistically using the GraphPad Prism software (Version 6.0) and R for Windows (R 4.1.1).<br>Survival curves were calculated according to the Kaplan-Meier method.<br>Flow Cytometry data was analyzed using CytExpert software (Beckman Coulter) and FlowJo version 10.<br>The statistical significance of comparisons between two groups was analyzed with a two-tailed Student's t-test. One-way analysis of variance (ANOVA) was used to compare differences among multiple groups. Two-way ANOVA was used to assess the interaction between two independent factors. Categorical variables were analyzed using the Chi-square test when all expected cell frequencies were ≥ 5; otherwise, Fisher's exact test was employed for small sample sizes or sparse data.

For manuscripts utilizing custom algorithms or software that are central to the research but not yet described in published literature, software must be made available to editors and reviewers. We strongly encourage code deposition in a community repository (e.g. GitHub). See the Nature Portfolio guidelines for submitting code & software for further information.

Data

Policy information about availability of data

All manuscripts must include a data availability statement. This statement should provide the following information, where applicable:

- Accession codes, unique identifiers, or web links for publicly available datasets
- A description of any restrictions on data availability
- For clinical datasets or third party data, please ensure that the statement adheres to our policy

The raw single-cell sequencing data generated in this study have been deposited in the Genome Sequence Archive at National Genomics Data Center, China (https://ngdc.cncb.ac.cn/gsa/browse/CRA028289 ; BioProject: PRJCA043637).
The raw RNA-Sequencing data generated in this study have been deposited in National Center for Biotechnology Information (https://www.ncbi.nlm.nih.gov/bioproject/PRJNA1295162; BioProject: PRJNA1295162).
The mass spectrometry-based untargeted metabolomics data have been deposited to Metabolights under the accession number MTBLS12768 (https://www.ebi.ac.uk/metabolights/editor/MTBLS12768/overview).
The CHIP-Seq and ATAC-Seq data were downloaded from the GEO under accession code GSE234179 (https://www.ncbi.nlm.nih.gov/geo/query/acc.cgi?acc=GSE234179).
The code used to analyse the dataset is openly available at GitHub (https://github.com/shuangyuePan/Bone-marrow-ScRNA-Seq) and the https://doi.org/10.5281/zenodo.19248693. The remaining data are available within the Article, Supplementary Information or Source Data file. Source data are provided with this paper.

Research involving human participants, their data, or biological material

Policy information about studies with human participants or human data. See also policy information about sex, gender (identity/presentation), and sexual orientation and race, ethnicity and racism.

Reporting on sex and gender	Sex and gender were not considered in this study and this information was not collected as part of our protocol.
Reporting on race, ethnicity, or other socially relevant groupings	Race, ethnicity, or other socially relevant groupings were not involved.
Population characteristics	In our cohort of 663 patients, the majority (n=556, 83.9%) were over 35 years of age, while 107 patients (16.1%) fell within the 18-34 age range. Regarding menopausal status, 302 patients (45.5%) were menopause and 359 (54.1%) were pre-menopausal, with menopausal status unknown for 2 patients (0.3%). Family history of breast cancer was present in 131 patients (19.8%), while 532 patients (80.2%) had no documented family history. Molecular subtyping classified 239 cases (36.0%) as triple-negative, 114 (17.2%) as HER2-positive, 141 (21.3%) as Luminal A, and 157 (23.7%) as Luminal B, with 12 cases (1.8%) unclassified subtype.
Recruitment	A cohort of 663 patients with breast cancer were recruited at the Fudan University Shanghai Cancer Center. Informed consent was obtained for each patient involved. And no any self-selection of the data was involved.
Ethics oversight	This study was approved and monitored by the Ethics Committee of FUSCC (1705172-9).

Note that full information on the approval of the study protocol must also be provided in the manuscript.

Field-specific reporting

Please select the one below that is the best fit for your research. If you are not sure, read the appropriate sections before making your selection.

☒ Life sciences ☐ Behavioural & social sciences ☐ Ecological, evolutionary & environmental sciences

For a reference copy of the document with all sections, see nature.com/documents/nr-reporting-summary-flat.pdf

Life sciences study design

All studies must disclose on these points even when the disclosure is negative.

Sample size	No statistical method was used to predetermine sample size. Sample size was chosen based on previous reference in the field. All experiments maintained minimum sample sizes of n ≥ 3 for in vitro replicates and n ≥ 5 for in vivo cohorts.
Data exclusions	No data were excluded from analysis.
Replication	For each representative data, experiments were performed at least three times with similar results unless otherwise noted in the manuscript.
Randomization	Randomization was used for mouse samples and experiment in vitro. No randomization was used for patient samples since no treatment was given.
Blinding	No blinding was applied.

Reporting for specific materials, systems and methods

We require information from authors about some types of materials, experimental systems and methods used in many studies. Here, indicate whether each material, system or method listed is relevant to your study. If you are not sure if a list item applies to your research, read the appropriate section before selecting a response.

Materials & experimental systems

n/a	Involved in the study	
	x	Antibodies
	x	Eukaryotic cell lines
x		Palaeontology and archaeology
	x	Animals and other organisms
x		Clinical data
x		Dual use research of concern
x		Plants

Methods

n/a	Involved in the study	
	x	ChIP-seq
	x	Flow cytometry
x		MRI-based neuroimaging

Antibodies

Antibodies used

For flow cytometry staining:
Anti-human CD45, abs1840506, Absin, 1:100;
Anti-human CD11b,550993, BD Pharmingen, 1:200;
Anti-human CD14,abs1840145, Absin, 1:100;
Anti-human CD66b,305116, BD Pharmingen, 1:200;
Anti-human HLA-DR,562804, BD Pharmingen, 1:200;
Anti-mouse CD45,557659, BD Pharmingen, 1:200;
Anti-mouse CD11b,557396, BD Pharmingen, 1:200;
Anti-mouse Ly6C,560592, BD Pharmingen, 1:200;
Anti-mouse Ly6G,560599, BD Pharmingen, 1:200.
For western blot:
Anti-ARG2 Antibody, Bs-11397R-Bio, Bioss, 1:300;
Anti-ODC1 Antibody, Ab97395, Abcam, 1:1000;
Anti-OAT Antibody, Ab137679, Abcam, 1:1000;
Anti-ARID1A Antibody, 12354S, CST, 1:1000;
Anti-rabbit IgG, HRP-linked Antibody,7074 CST, 1:3000;
Anti-mouse IgG, HRP-linked Antibody,7076 CST, 1:3000;
GAPDH,10494-1-AP, Proteintech, 1:3000.
For immunohistochemistry:
Anti-ARG2 Antibody, Bs-11397R-Bio, Bioss, 1:200;
Anti-ODC1 Antibody, Ab97395, Abcam, 1:200;
Anti-OAT Antibody, Ab137679, Abcam, 1:200;
Anti-ARID1A Antibody, 12354S, CST, 1:500.
For immunofluorescence:
Anti-mouse CD11b,17800, CST, 1:100;
Anti-mouse Ly6G, 88876, CST, 1:200;
Anti-human CD11b, ab133357, Abcam, 1:2000;
Anti-human CD66b, ab197678, Abcam, 1:200;
Anti-human CD14, ab133335, Abcam, 1:800;
Anti-human HLA-DR, ab92511, Abcam, 1:200;
Anti-human LOX-1, SC-373995, Santa Cruz, 1:200.
For PMN-MDSC deletion:
Anti-mouse Ly6G, BE0075-1 , BioXCell;
Mouse IgG, I8765, Sigma.

Validation

The validation of all of the antibodies depends on product data sheet and published literature.

Eukaryotic cell lines

Policy information about cell lines and Sex and Gender in Research

Cell line source(s)

MDA-MB-231(HTB-26), BT-549(HTB-122) and 4T-1(CRL-2539) cells were obtained from the American Type Culture Collection (ATCC).

Authentication

STR profiling

Mycoplasma contamination

All lines were tested and confirmed to be free of mycoplasma at the time of experiments.

Commonly misidentified lines (See ICLAC register)

N/A

Animals and other research organisms

Policy information about studies involving animals; ARRIVE guidelines recommended for reporting animal research, and Sex and Gender in Research

Laboratory animals	Six- to eight-week-old female Balb/c and NSG mice were used. Arid1a flox/flox;Mmtv-Cre mice were purchased from Shanghai Model Organisms Center, Inc (Shanghai, China). All animals were maintained in a specific pathogen-free (SPF) facility with a temperature between 21 ± 2°C, humidity of 45 to 65%, and a regulated 12-hour light/dark cycle. The animals were provided with normal diet food and water ad libitum (Jiangsu Xietong Pharmaceutical Bio-engineering Co., Ltd., catalog number 1010088).
Wild animals	The study did not involve wild animals.
Reporting on sex	These results apply only to females.
Field-collected samples	The study did not involve samples collected from the field.
Ethics oversight	The animal experiments were approved by the Fudan University Shanghai Cancer Center Institutional Review Board and strictly carried out in accordance with the People's Republic of China Legislation Regarding the Use and Care of Laboratory Animals (FUSCC-IACUC-2023673).

Note that full information on the approval of the study protocol must also be provided in the manuscript.

Plants

Seed stocks	N/A
Novel plant genotypes	N/A
Authentication	N/A

ChIP-seq

Data deposition

☒ Confirm that both raw and final processed data have been deposited in a public database such as GEO.

☐ Confirm that you have deposited or provided access to graph files (e.g. BED files) for the called peaks.

Data access links<br>*May remain private before publication.*	https://www.ncbi.nlm.nih.gov/geo/query/acc.cgi?acc=GSE234179.
Files in database submission	ChIP-Seq dataset in this study was downloaded from GSE234179.
Genome browser session<br>(e.g. UCSC)	Not applicable.

Methodology

Replicates	Reported in the published literature (Chen et al. Cancer Commun (Lond), 2023, PMID: 37434394).
Sequencing depth	Reported in the published literature (Chen et al. Cancer Commun (Lond), 2023, PMID: 37434394).
Antibodies	Reported in the published literature (Chen et al. Cancer Commun (Lond), 2023, PMID: 37434394).
Peak calling parameters	Reported in the published literature (Chen et al. Cancer Commun (Lond), 2023, PMID: 37434394).
Data quality	Reported in the published literature (Chen et al. Cancer Commun (Lond), 2023, PMID: 37434394).
Software	Model-based Analysis of ChIP-Seq (MACS, v1.4.3) was used.

Flow Cytometry

Plots

Confirm that:

- [x] The axis labels state the marker and fluorochrome used (e.g. CD4-FITC).
- [x] The axis scales are clearly visible. Include numbers along axes only for bottom left plot of group (a 'group' is an analysis of identical markers).
- [x] All plots are contour plots with outliers or pseudocolor plots.
- [x] A numerical value for number of cells or percentage (with statistics) is provided.

Methodology

Sample preparation	Bone marrow was flushed from mouse tibia and femurs using a needle. Bone marrow cells were filtered through a 70 µm filter and centrifuged at 600g at 4°C for 7 min. Cells were incubated for 5 min at room temperature (RT) in 2 ml of red blood cell lysis buffer. The lysis reaction was terminated and centrifuged again, after which the living cells were counted using the automated cell counter. Cells were harvested and labeled for flow cytometry with antibodies.<br>Tumors were surgically excised from the tibia and washed in PBS. Necrotic portions of the tumor were carefully excised. The viable tumor tissues were then minced into small fragments and dissociated in DMEM with collagenase type IV, deoxyribonuclease, hyaluronidase and bovine serum albumin (BSA) at 37°C for 2 hours to facilitate enzymatic digestion. The resulting cell suspension was passed through a 70 µm cell strainer to remove undigested tissue fragments. The filtrate was then centrifuged at 500g for 7 minutes to pellet the cells. The supernatant was discarded, and the cell pellet was resuspended in fresh culture medium for further analysis or experimental use.<br>Bone-marrow cells were obtained from 6-week-old Balb/c mice and treated with GM-CSF and IL-6 after removing erythrocytes. After co-culturing with breast cancer cells and metabolites for 72 h, cells were harvested and labeled for flow cytometry with antibodies.<br>Human peripheral blood was incubated with 3% dextran for 18 min, and supernatants were collected and followed by differential density gradient separation. Samples were centrifuged at 500 relative centrifugal force for 30 min at 20°C. PBMCs including granulocytes were collected and incubated with GM-CSF and IL-6 for 72h, cells were harvested and labeled for flow cytometry with antibodies.
Instrument	CytoFLEX LX (Beckman Coulter)
Software	CytExpert software (Beckman Coulter); FlowJo v10.8.1.
Cell population abundance	Post-sort purities were assessed by flow cytometry and confirmed to be > 95%.
Gating strategy	Reported in Supplementary information.

- [x] Tick this box to confirm that a figure exemplifying the gating strategy is provided in the Supplementary Information.
