# Supplementary material for: Targeting arginine metabolism reverses bone immunosuppressive microenvironment and metastasis in ARID1A-deficient triple negative breast cancer
Source: Nat Commun. 2026 May 26;17:6863. doi: 10.1038/s41467-026-73574-3 (PMC13389217; doi:10.1038/s41467-026-73574-3)
Supplement: Supplementary file 3 — Transparent Peer Review file [file 41467_2026_73574_MOESM3_ESM.pdf]

nature portfolio

Peer Review File

Targeting arginine metabolism reverses bone immunosuppressive microenvironment and metastasis in ARID1A-deficient triple negative breast cancer

Corresponding Author: Professor Xichun Hu

Parts of this Peer Review File have been redacted as indicated to remove third-party material.

This file contains all reviewer reports in order by version, followed by all author rebuttals in order by version.

Version 0:

Reviewer comments:

Reviewer #1

(Remarks to the Author)
In this study, Pan et al discover a novel link between ARID1A loss in breast cancer cells and the upregulation of arginine metabolism, which promotes bone metastasis via expansion of PMN-MDSCs in the tumor microenvironment. ARID1A is a core subunit of the SWI/SNF chromatin remodeling complex which is found mutated in almost 10% of all cancers. Previous studies have shown that this chromatin regulator controls the expression of a diverse set of cellular metabolism genes. Here the authors use transcriptomic and metabolomic approaches to link ARID1A loss to changes in arginine metabolism gene expression and subsequent production of ornithine and spermine in triple negative breast cancer cells. Using a comprehensive set of in vivo mouse models as well as in vitro human cell lines, the authors show that loss of ARID1A in tumor cells promotes bone metastasis via the expansion of PMN-MDSCs. Furthermore, they show that exogenous treatment with ornithine or spermine is sufficient to expand PMN-MDSCs, suggesting that the production of these metabolites by Arid1a KO cells in the tumor microenvironment is a potent driver of metastasis. Finally, from an interesting therapeutic perspective, they show that treatment with small molecule inhibitors of ornithine and spermine production prevents ARID1A-deficient triple negative breast cancer bone metastasis.

A major strength of this study is the extensive cellular and animal data presented. The authors make use of multiple cell lines (mouse and human), mouse models and transplantation approaches (intracardiac and intratibial) to dissect the link between ARID1A loss and bone metastasis. However, the molecular analysis lacks many details and is hard to follow, in particular the transcriptomics and metabolomics. I have raised several concerns for the authors and hope that my comments will help improve the clarity of the manuscript.

1. Both the transcriptomics and metabolomics data need to be presented in more detail beyond the KEGG enrichment analysis in Figure 3. The authors should provide PCA plots, volcan plots etc. to give a better sense of how many genes/metabolites are differentially regulated in the two conditions. Also, the ChIP-seq data is poorly described, H3K27ac should be mentioned in the text, as well as more details on the experimental design. Is this analysis of published data from the same experimental conditions as used for the RNA-seq?

2. To what extent is ARID1A loss impacting SWI/SNF activity in these tumor cells? In addition to the RNAseq data, ATAC-seq would allow for a more direct assessment of SWI/SNF dependent changes in chromatin accessibility. In addition to the ARID1A ko experiments, using SWI/SNF inhibitors would also determine if is this is a general function of SWI/SNF or specifically linked to ARID1A.

3. ARID1A functions within the multi-subunit SWI/SNF complex yet overexpression of a single subunit reduces metastasis whereas knockout increases metastasis compared to control cells. Could the authors comment on why the phenotype is so sensitive to ARID1A dosage within the SWI/SNF complex, is ARID1B expressed in these cancer models?

4. Is it known what makes PMN-MDSCs so sensitive to ornithine and spermine compared to other cells in the niche? Do they express specific transporters at their cell surface? This could be addressed using the scRNAseq datasets.

5. Have the authors tried to combine ornithine and spermine treatments? Same question for the inhibitors, what is the consequence of dual inhibition?

Minor:

1. In the ctDNA analysis, are mutations in any other SWI/SNF subunits detected? As mentioned by the authors, almost 10% of all cancers have mutations in ARID1A, so detecting 53/663 in this cohort does not appear as a strong enrichment. Is the mechanism described in this study specific to the ARID1A subunit or a more general SWI/SNF function?

2. For the engineering of the Arid1a KO and OE cell lines, more details are required. While the cellular phenotypes are striking, could the authors comment on the incomplete nature of the knock-out as well as how they overexpressed such a large protein using viruses (dCas9 approach?).

3. The ChIP-seq dataset GSE98765 does not link to the study by Chen et al.

Reviewer #2

(Remarks to the Author)
This manuscript investigates an important topic that is gaining considerable interest of metabolic crosstalk between cancer cells and immune cells. This is a complex and nuanced topic, and the current study attempts to understand the important question of what tumour mutations change tumour cell metabolism to promote bone marrow metastasis tropism. Particularly important to note is that there is currently no effective/ targeted treatment option for patients with breast cancer bone marrow metastasis. This underscores the importance of the work. Whilst this is an interesting and important topic, the manuscript in its current form lacks major controls and overstates the data making it very hard to interpret.
Unfortunately, there are no good models of breast cancer -> bone metastasis. This project relies on cell line injection models and the limitations of this should be discussed.
In the introduction, the authors define PMN-MDSCs as CD11b+Ly6G+Ly6C-low. This is precisely the same definition as a neutrophil and highlights the problem with MDSC nomenclature. A fundamental problem with using MDSC nomenclature is that it treats PMN-MDSCs as distinct from neutrophils and M-MDSCs as distinct from monocytes, when in fact recent work has demonstrated they represent functional states of neutrophils and monocytes. Labeling them as a separate category obscures their true lineage and hampers our ability to understand their biology in the broader context of myeloid cell plasticity.
Injection of luciferase labelled cells: allows to follow tumour cells but Luciferase is not naturally expressed in mammalian tissues; therefore, introduction of luciferase-expressing cells provides a neoantigen that is recognised as foreign by the host immune system. This can elicit inflammatory responses, contributing to the overall immunogenicity of the injected cell line, and skewing readouts of inflammatory cells.
The methods are not very detailed and a lot of experimental details that are required to understand the data are missing. For example, tumour cell growth after manipulation, size of tumours at euthanasia (is it the same between experimental groups?), Figure 5, is is not clear which model the experiments are performed in.
Can we assume the MDA-MB-231 would behave the same way as 4T1 in terms of bone marrow metastasis?
Manipulations of Arid1 – do tumours and cell lines grow at the same rate upon Arid1-ko? Throughout the manuscript, we cannot understand if the results are a function of faster tumour growth / bigger tumours.
Figure 4E – in the feature plots, do these cells come from mice injected with Arid1NC or Arid1ko? What is the difference between neutrophil and a PMN-MDSC. As mentioned above, recent publications have indicated that these are not separate entities, but a functional continuum impacted by the systemic environment driven by the tumour.
Figure 4F – using pseudotime trajectories in this manner is increasingly common but it is a fundamentally flawed methodology. In the case of neutrophils and PMN-MDSCs, using pseudotime is problematic because these populations are highly plastic and can interconvert depending on environmental cues. Without a clear, unidirectional differentiation pathway, the algorithm may infer a trajectory that assumes PMN-MDSCs arise from neutrophils (or vice-versa), even though the real biology may reflect activation or functional reprogramming rather than a linear differentiation process. In addition, the authors use “Pre-MDSC” but do not define the population.
Further, Monocle relies heavily on dimensionality reduction and the construction of a minimum-spanning tree, which means the inferred trajectory depends strongly on how the algorithm embeds the data and on the choice of root cell. If the starting point is mis-specified, the directionality of the pseudotime is effectively arbitrary and the “differentiation path” can be a computational artifact rather than a true biological progression. Given that the starting point is not defined by the authors nor is what “early myeloid”, “pre-MDSC”, this data is impossible to interpret.
The gating and identification of human PMN-MDSCs in figure 5A / S7B is flawed. The gating strategy appears to take a lot of CD11b- cells. Furthermore, CD66 gate looks predominantly like boarder effect/ spread. FMOs are required. As it stands, this is not convincing that there is an increase in PMN-MDSCs.
Mtmv-Cre mice experiments: was there any difference in primary tumour growth between Arid1-ko and Arid1-wt tumours? Were mice euthanized at the same time point (but potentially with different tumour sizes) or at the same tumour size?
Figure 5C-F - the authors indicate that they “replicate the intracardial injection model” but it is not clear with which cells into which mice therefore the data are uninterpretable. Similarly, for the intratibial injection.
Do the tumour cells grow the same in the presence of CB-1158 and DFMO? Do they curtail PMN-MDSCs because the

tumour cells die? Perhaps a reductionist / in vitro co-culture experiment is required to fully understand how these interact and to be sure that the effect of CB-1158 and DFMO are not indirect.
Minor comments:
- Figure 1C – no associations with liver, lung, brain or LN mets. Looks like this is because it is underpowered and there could be an association with liver
- Figure 4D – the text says: "we found the could granulocytic myeloid cells in the Arid1ko group was significantly elevated compared to that of Arid1NC group". But there is no quantification. T-SNE intensity call also reflect shifts in populations

Reviewer #3

(Remarks to the Author)
Bone metastasis represents a lethal complication of advanced breast cancer. Pan et al. elucidated a novel mechanism for bone metastasis. They found that the AT-rich interactive domain-containing protein 1A (ARID1A) remodeled the immunosuppressive metastatic microenvironment through upregulation of arginine metabolism. In their cohort study involving 663 patients with metastatic breast cancer, circulating tumor DNA (ctDNA) analysis revealed a significant positive correlation between ARID1A mutations and metastatic incidence. Notably, most of these mutations resulted in ARID1A loss-of-function, while ARID1A knockdown in tumor cells markedly activated arginine metabolic pathways—particularly those governing ornithine and spermine synthesis. The researchers further demonstrated that tumor-derived ornithine and spermine drove the expansion of polymorphonuclear myeloid-derived suppressor cells (PMN-MDSCs) in the bone marrow microenvironment, thereby promoting osteotropic metastasis. Importantly, pharmacological inhibition of key arginine metabolic enzymes—arginase 2 (ARG2) and ornithine decarboxylase 1 (ODC1)—significantly attenuated bone metastasis in preclinical models. The findings provide novel insights into metastatic microenvironment remodeling and open new avenues for preventing bone metastasis in breast cancer patients. However, several concerns should be further addressed:
1. While genomic alterations in ARID1A were linked to metastasis, the study did not explicitly address whether corresponding changes in ARID1A protein expression similarly correlate with metastatic progression. Dose ARID1A protein downregulation in primary tumors or metastatic sites serve as a functional biomarker for, or contributor to, breast cancer bone metastasis?
2. Pan et al. reported an inverse correlation between ARID1A expression and the levels of arginine metabolic enzymes ARG2/ODC1 in metastatic breast cancer. The molecular mechanisms underlying ARID1A-mediated regulation of arginine metabolism were still unclear. Does ARID1A directly repress ARG2/ODC1 transcription through chromatin remodeling?
3. The authors showed the expression level of ARID1A is negatively correlated with the production of ornithine/spermine and the expansion of PMN-MDSCs. To further substantiate the clinical significance of these findings, they should validate the relationships between ornithine/spermine levels and metastasis incidence, as well as between ARID1A expression levels and the properties of PMN-MDSCs in breast cancer patients.
4. Knockdown of ARID1A or supplementation of ornithine and spermine in the culture medium promotes the expansion of PMN-MDSCs. To further elucidate the underlying mechanism, it is crucial to verify whether ARID1A regulates PMN-MDSCs expansion through the modulation of ornithine and spermine levels, specifically via the enzymes arginase 2 (ARG2) and ornithine decarboxylase 1 (ODC1).
5. The authors have proposed that ARID1A promotes the expansion of PMN-MDSCs by upregulating the arginine metabolic pathway. To substantiate this hypothesis, it is essential to conduct experiments that specifically inhibit the arginine metabolic pathway within PMN-MDSCs.

Reviewer #4

(Remarks to the Author)
I think the science shown is pretty robust and the mechanistic work to support the science is sound. I think the conclusions ignore the fact that most bone metastases don't seem to have this mechanism (as evidenced by 45% of patients with bone mets without ARID1A mutations and a discussion on potential other mechanisms or more acknowledgement for this I would say is important. I think also the clinical significance of this could be discussed in more detail including a discussion of ARG inhibitors and current clinical trials that are underway and have been conducted for these targets.

Version 1:

Reviewer comments:

Reviewer #1

(Remarks to the Author)
I thank the authors for addressing all the points raised and for their efforts generating several new in vivo datasets which have strengthened the manuscript.

In particular, the experiments using BRM014 inhibitors add interesting molecular insights into the activity of the residual SWI/SNF chromatin remodelling complexes which lack Arid1a subunits. These results also support the authors model which suggests that Arid1a is the main driver of this mechanism rather than the SWI/SNF complex as a whole. Also, the explanation for the unique sensitivity of PMN-MDSCs to ornithine and spermidine treatments is interesting as are the new experiments revealing an additive effect of the inhibitors on metastasis.

Finally, while the new details on the analysis of the RNA-seq, ChIP-seq and ATAC-seq is an important addition, it would be

interesting to also include a genome-browser view of just the Arg2 locus to integrate all these data in one figure panel. Having a single locus overview of the H3K27ac levels, chromatin accessibility and gene expression would help support the slightly counter intuitive model whereby Arid1a loss leads to de-repression at the Arg2 locus.

(Remarks on code availability)

Reviewer #2

(Remarks to the Author)
I want to commend the authors on addressing thoroughly all the previous comments. This work is very interesting and provides novel insight into the mechanisms by which tumours induce an immunosuppressive microenvironment.
The authors have addressed my comments sufficiently.

(Remarks on code availability)

Reviewer #3

(Remarks to the Author)
The authors have addressed all my concerns.

(Remarks on code availability)

Open Access This Peer Review File is licensed under a Creative Commons Attribution 4.0 International License, which permits use, sharing, adaptation, distribution and reproduction in any medium or format, as long as you give appropriate credit to the original author(s) and the source, provide a link to the Creative Commons license, and indicate if changes were made.
In cases where reviewers are anonymous, credit should be given to 'Anonymous Referee' and the source.
The images or other third party material in this Peer Review File are included in the article's Creative Commons license, unless indicated otherwise in a credit line to the material. If material is not included in the article's Creative Commons license and your intended use is not permitted by statutory regulation or exceeds the permitted use, you will need to obtain permission directly from the copyright holder.
To view a copy of this license, visit https://creativecommons.org/licenses/by/4.0/

We thank the reviewers for their thought-provoking comments and have extensively revised the manuscript accordingly. We believe the revised manuscript is substantially improved in its scientific rigor, clarity, and strength of argument.

REVIEWER COMMENTS

Reviewer #1 (Remarks to the Author); expert in epigenetics:

In this study, Pan et al discover a novel link between ARID1A loss in breast cancer cells and the upregulation of arginine metabolism, which promotes bone metastasis via expansion of PMN-MDSCs in the tumor microenvironment. ARID1A is a core subunit of the SWI/SNF chromatin remodeling complex which is found mutated in almost 10% of all cancers. Previous studies have shown that this chromatin regulator controls the expression of a diverse set of cellular metabolism genes. Here the authors use transcriptomic and metabolomic approaches to link ARID1A loss to changes in arginine metabolism gene expression and subsequent production of ornithine and spermine in triple negative breast cancer cells. Using a comprehensive set of in vivo mouse models as well as in vitro human cell lines, the authors show that loss of ARID1A in tumor cells promotes bone metastasis via the expansion of PMN-MDSCs. Furthermore, they show that exogenous treatment with ornithine or spermine is sufficient to expand PMN-MDSCs, suggesting that the production of these metabolites by Arid1a KO cells in the tumor microenvironment is a potent driver of metastasis. Finally, from an interesting therapeutic perspective, they show that treatment with small molecule inhibitors of ornithine and spermine production prevents ARID1A-deficient

triple negative breast cancer bone metastasis.

A major strength of this study is the extensive cellular and animal data presented. The authors make use of multiple cell lines (mouse and human), mouse models and transplantation approaches (intracardiac and intratibial) to dissect the link between ARID1A loss and bone metastasis. However, the molecular analysis lacks many details and is hard to follow, in particular the transcriptomics and metabolomics. I have raised several concerns for the authors and hope that my comments will help improve the clarity of the manuscript.

1. Both the transcriptomics and metabolomics data need to be presented in more detail beyond the KEGG enrichment analysis in Figure 3. The authors should provide PCA plots, volcan plots etc. to give a better sense of how many genes/metabolites are differentially regulated in the two conditions. Also, the ChIP-seq data is poorly described, H3K27ac should be mentioned in the text, as well as more details on the experimental design. Is this analysis of published data from the same experimental conditions as used for the RNA-seq?

We would like to express our sincere gratitude to the reviewer for the insightful and valuable comments, which are crucial for us to optimize the presentation of transcriptomic, metabolomic, and ChIP-seq data and enhance the overall rigor of our manuscript. We have carefully addressed all the concerns raised, with detailed revisions as follows:

1. To make the transcriptomic and metabolomic data more informative beyond KEGG enrichment analysis, we have now added PCA plots (for sample clustering

evaluation), OPLS-DA plots (for group separation validation) and volcano plots (for direct visualization of differentially regulated genes/metabolites) as supplementary Figs. 4A-D. Detailed descriptions of these plots, including the quantification of differentially expressed genes/metabolites between the two conditions, have been supplemented in the Results section (Page 8, line 201-205).

2. We have supplemented the explicit mention of H3K27ac in the corresponding text (Page 9, line 229) as suggested. Moreover, we have expanded the description of the ChIP-seq experimental design and analytical procedures in the revised manuscript (Page 33, line 902-913) to improve the clarity and reproducibility of this part of the study.
3. For the consistency of experimental conditions between datasets: the published ChIP-seq data and our RNA-seq data were derived from parallel experiments using MDA-MB-231 cells, with the same grouping strategy (ARID1A negative control vs. ARID1A knockout), ensuring the comparability of the two datasets for subsequent joint analysis.

(Page 8, line 201-205)

"Transcriptomic and metabolomic profiling of ARID1A^{KO} and negative control cells revealed distinct global profiles for each group (Supplementary Figs. 4A and B). Volcano plots identified 1445 upregulated and 2835 downregulated genes, along with 693 upregulated and 710 downregulated metabolites (Supplementary Figs. 4C and D)."

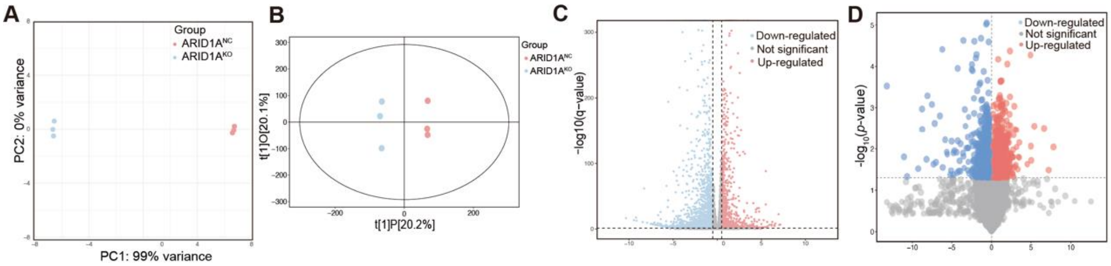

Fig S4. ARID1A-deficient TNBC upregulates the arginine metabolic pathway. (A) *PCA plot of RNA-Seq from ARID1A*^{NC} *and ARID1A*^{KO} *cells. (**B**) OPLS-DA plot of metabolomics from ARID1A*^{NC} *and ARID1A*^{KO} *cells. (**C**) Volcano plot of differentially expressed genes from RNA-Seq in ARID1A*^{NC} *and ARID1A*^{KO} *cells. (**D**) Volcano plot of differential metabolites from metabolomics in ARID1A*^{NC} *and ARID1A*^{KO} *cells.*

(Page 9, line 229)

"Notably, the H3K27ac enrichment at the specific regulatory region of ARG2 was increased by 10-fold in ARID1A^{KO} *MDA-MB-231 cells compared with control cells, indicating enhanced transcriptional potential of ARG2 in the absence of ARID1A (Fig. 3E)."*

(Page 33, line 902-913)

"ATAC-seq and ChIP-seq library construction and analysis

The ATAC-seq and ChIP-seq data for MDA-MB-231 cells were obtained from the Gene Expression Omnibus (GSE234179), deposited by our research team (Chen et al., Cancer Commun, 2023). ChIP-seq libraries were sequenced on an Illumina NovaSeq 6000 platform at Cloud-seq (Shanghai, China). ATAC-seq libraries were sequenced on an Illumina X-Ten system. Both generated 150-bp paired-end reads. ATAC-seq and

H3K27ac ChIP-seq signals were normalized to RPKM, and comparative log2(KO/NC) tracks were generated. ATAC-seq peaks were called from aligned reads using MACS3, and a union peak set was intersected with transcriptional start sites (TSSs, UCSC refGene, hg38) to define promoter-proximal regions. Visualization matrices were generated using deepTools computeMatrix in reference-point mode with a window of ±2 kb and a bin size of 100 bp."

2. To what extent is ARID1A loss impacting SWI/SNF activity in these tumor cells? In addition to the RNAseq data, ATAC-seq would allow for a more direct assessment of SWI/SNF dependent changes in chromatin accessibility. In addition to the ARID1A ko experiments, using SWI/SNF inhibitors would also determine if is this is a general function of SWI/SNF or specifically linked to ARID1A.

We sincerely thank the reviewer for the constructive and insightful suggestions. To directly evaluate SWI/SNF-dependent chromatin accessibility changes as suggested, we performed ATAC-seq analysis on ARID1A^{KO} and negative control MDA-MB-231 cells (GSE234179). Results showed that ARID1A deficiency reduced global chromatin accessibility, indicating impaired SWI/SNF-mediated chromatin remodeling—consistent with ARID1A being a core SWI/SNF subunit. This finding is presented in Supplementary Fig. 4E, with descriptions incorporated into the revised manuscript (Page 9, line 221-228).

To distinguish whether the observed phenotypes reflect general SWI/SNF function or ARID1A-specific effects, we utilized a SWI/SNF inhibitor (BRM014, a catalytic

inhibitor targeting SWI/SNF ATPase) and an intracardiac injection bone metastasis model in Balb/c mice, with evaluations in both *Arid1a*^{NC} and *Arid1a*^{KO} groups. BRM014 had no significant effect on bone metastasis in *Arid1a*^{NC} mice but suppressed bone metastasis in *Arid1a*^{KO} mice. Similarly, BRM014 failed to alter the concentration of PMN-MDSCs in the bone marrow of *Arid1a*^{NC} mice but significantly reduced their elevated levels in *Arid1a*^{KO} mice. These data confirm that ARID1A deficiency-induced bone metastasis depends on the residual SWI/SNF complex; notably, standalone SWI/SNF inhibition cannot mimic the phenotypic alterations caused by ARID1A deficiency, which implicates additional ARID1A-specific functional roles in mediating tumor progression. These results are shown in Supplementary Figs. 12A–C, with detailed analyses added to the revised manuscript (Page 17, line 460-476).

(Page 9, line 221-228)

"To explore the potential regulatory mechanism underlying low ARID1A expression-induced ARG2 transcription activation, we performed Assay for transposase accessible chromatin (ATAC)-seq and Chromatin Immunoprecipitation-Sequencing (ChIP-seq) analysis in ARID1A^{KO} *and negative control MDA-MB-231 cells. Our analyses revealed that ARID1A deficiency led to a global reduction in chromatin accessibility, indicating impaired SWI/SNF-dependent chromatin remodeling—consistent with ARID1A's established role as a core subunit of the SWI/SNF complex (Supplementary Fig. 4E)."*

E

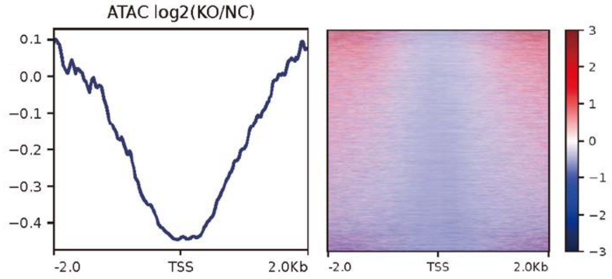

Supplementary Fig.4 ARID1A-deficient TNBC upregulates the arginine metabolic pathway. *(E) ATAC signal at global chromatin accessibility in ARID1A^{KO} cells compared to ARID1A^{NC} cells.*

(Page 17, line 460-476)

"To evaluate whether ARID1A deficiency promotes bone metastasis through residual SWI/SNF activity, we employed a bone metastasis model using the BRM014, a catalytic inhibitor of SWI/SNF ATPase activity. We found that BRM014 treatment did not significantly alter bone metastasis in Arid1a^{NC} control mice. In contrast, BRM014 suppressed bone metastasis in Arid1a^{KO} mice (Supplementary Figs. 12A and B). Similarly, BRM014 failed to alter the concentration of PMN-MDSCs in the bone marrow of Arid1a^{NC} mice but significantly reduced their elevated levels in Arid1a^{KO} mice (Supplementary Fig. 12C). Collectively, these data indicate that bone metastasis driven by ARID1A deficiency is dependent on the residual SWI/SNF complex; notably, standalone SWI/SNF inhibition cannot mimic the phenotypic alterations caused by ARID1A deficiency, which implicates additional ARID1A-specific functional roles in mediating tumor progression. However, targeting the SWI/SNF complex poses a significant challenge due to concerns of on-target toxicity, stemming from its ubiquitous

expression and indispensable functions in normal cells, we pivoted to a more promising therapeutic strategy centered on targeting the arginine signaling pathway."

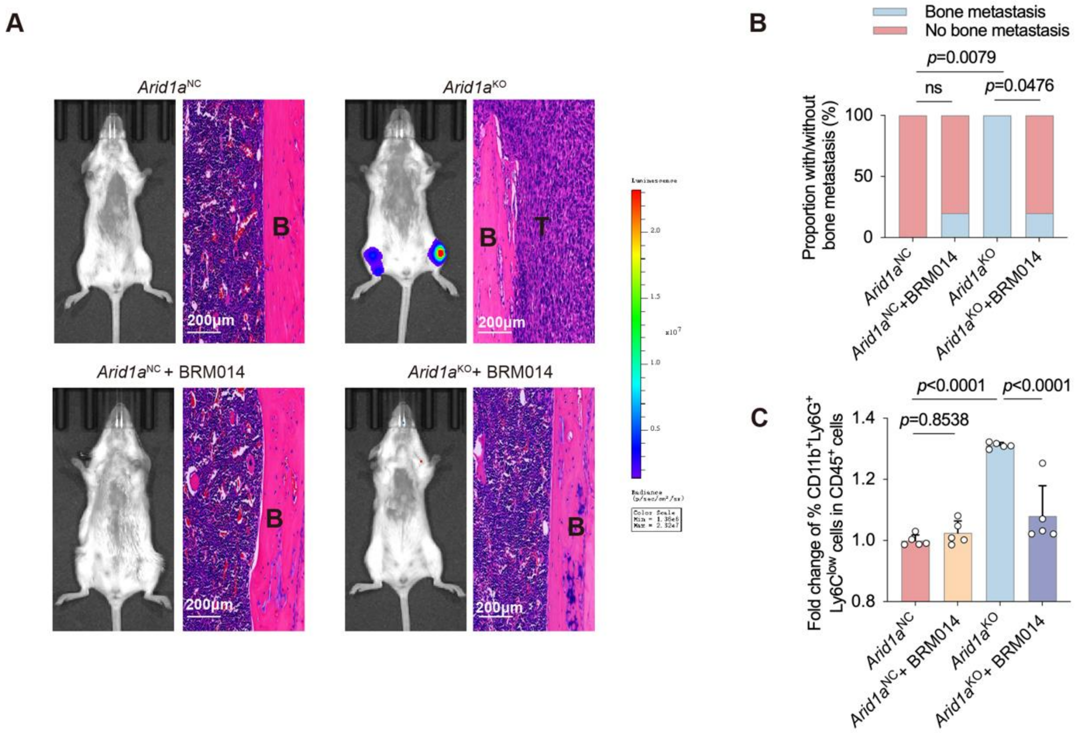

Supplementary Fig. 12. ARID1A deficiency relies on residual SWI/SNF complex to promote bone metastasis. *(A) BLI and H&E staining images of mice in Arid1a^{NC}, Arid1a^{NC} plus BRM014, Arid1a^{KO}, Arid1a^{KO} plus BRM014 group with intracardiac injection. Scale bar, 200μm. B bone, T tumor.* ***(B)*** *Statistical charts presenting the bone metastasis incidence in each group with intracardiac injection. n=5 per group, fisher's exact test was used.* ***(C)*** *Bone marrow of mice was harvested from each group and analyzed by flow cytometry to quantify PMN-MDSC proportions. n=5 per group, one-way ANOVA for multiple comparisons. p values are indicated in the figures. Source data are provided as a Source Data file.*

3. ARID1A functions within the multi-subunit SWI/SNF complex yet overexpression

of a single subunit reduces metastasis whereas knockout increases metastasis compared to control cells. Could the authors comment on why the phenotype is so sensitive to ARID1A dosage within the SWI/SNF complex, is ARID1B expressed in these cancer models?

We thank the reviewer for raising this critical point regarding the dosage sensitivity of ARID1A in our bone metastasis models and the potential role of ARID1B.

The bone metastasis phenotype is acutely sensitive to ARID1A dosage can be explained through its indispensable structural and functional roles within the SWI/SNF complex:

1. **Structural keystone of SWI/SNF complex:** ARID1A belongs to the SWI/SNF complex and is a core member of the ATP-dependent polymorphic BRG/BRM-associated factor (BAF) chromatin remodeling unit. Its presence or absence dictates the complex's protein composition, nuclear localization, and partner interactions (Jennifer N. Wu et al., Cancer Discov. 2013 Jan)[1]. A change in its dosage thus alters the fundamental identity and capability of the SWI/SNF complex in the cell.
2. **Functional roles in SWI/SNF complex:** The ARID1A protein comprises the DNA-binding subunit of SWI/SNF chromatin remodeling complexes and ARID1A regulates chromatin accessibility. Chromatin remodeling dynamically modifies chromatin architecture to allow access of condensed genomic DNA to the transcription regulatory machinery, thereby controlling gene expression (Sankari Nagarajan et al., Nat Genet. 2020 Feb)[2]. In our study, the loss of ARID1A altered chromatin accessibility which in turn activated ARG2 expression and promoted the

upregulation of the arginine metabolic pathway. This ultimately led to an increase in the concentration of PMN-MDSCs within the bone marrow tumor microenvironment, fostering an immunosuppressive milieu that facilitates enhanced tumor cell retention. This explains why the phenotype is exquisitely sensitive to ARID1A levels, as it controls gene expression by constituting an indispensable and uniquely encoded layer of regulatory control within the epigenetic complex.

Furthermore, we performed IHC to analyze *Arid1b* expression in tumors from *Arid1a*^{NC} and *Arid1a*^{KO} mice as suggested. The results showed that *Arid1b* was present in both *Arid1a*^{NC} and *Arid1a*^{KO} tumor tissues, but with no significant difference in its expression levels between the two groups (As shown in panels A and B of the figure below).

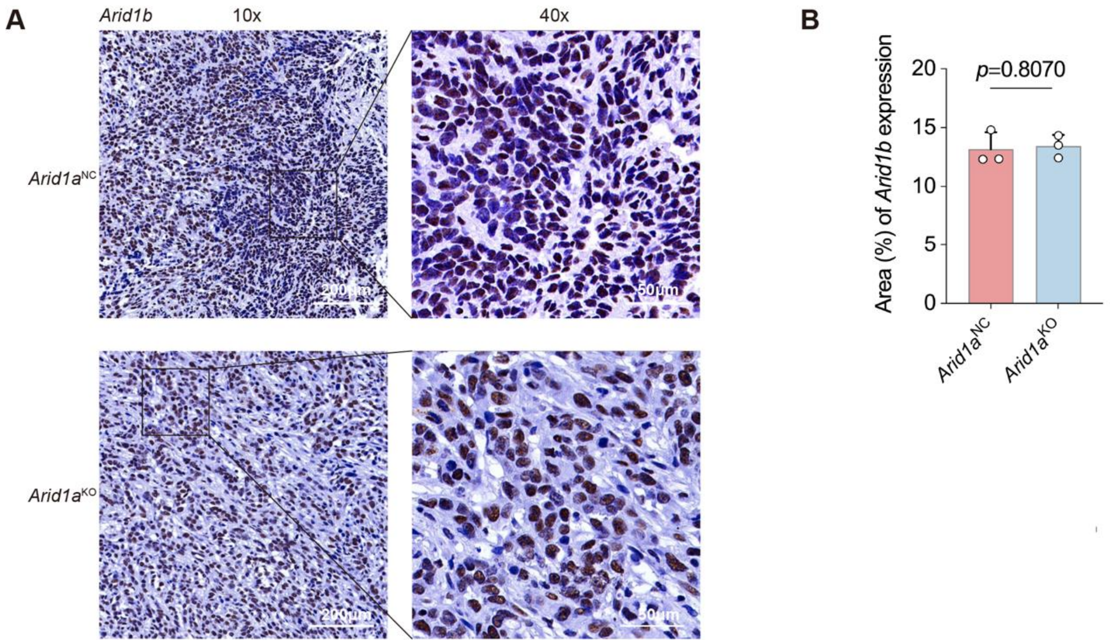

(A) *IHC images of Arid1b expression in tumors from Arid1a*^{NC} *mice compared to Arid1a*^{KO} *mice. Scale bar, 10x, 200μm.* ***(B)*** *Quantification of the protein expression*

levels of Arid1b in two groups. n=3 per group, unpaired two-tailed Student's t test was used.

4. Is it known what makes PMN-MDSCs so sensitive to ornithine and spermine compared to other cells in the niche? Do they express specific transporters at their cell surface? This could be addressed using the scRNAseq datasets.

We sincerely thank the reviewer for this insightful question. We propose that the specific sensitivity of PMN-MDSCs to ornithine and spermine stems from their inherent metabolic specialization in the arginine-polyamine axis. This is supported by three lines of evidence:

1. PMN-MDSCs are characterized by their ability to suppress immune responses primarily through arginine metabolism through the expression of immunosuppressive enzymes arginase 1 (ARG1) and, in some contexts, arginase 2 (ARG2) (Hamad Alshetaiwi et al., Sci Immunol. 2020 Feb; Filippo Veglia et al., Nat Rev Immunol. 2021 Aug)[3, 4]. This established role highlights that PMN-MDSCs are inherently specialized cells whose identity and function are closely tied to the core pathway involving arginine metabolism.

2. Our in-depth analysis of the ScRNA-seq datasets revealed that polyamine metabolic processes are specifically enriched in PMN-MDSCs compared to other cells (Supplementary Fig. 10A). Polyamines (putrescine, spermine, spermidine) are direct downstream metabolites synthesized from ornithine, which itself is derived from arginine. This directly links PMN-MDSCs to the downstream branch of

arginine metabolism where ornithine and spermine reside. This result has been added in the revised manuscript (Page 15, line 400-404).

3. In the ARID1A^{KO} group, where this sensitivity is pronounced, PMN-MDSCs exhibit upregulated expression of key enzymes (ARG2 and SAT1) which are pivotal for arginine and polyamine metabolism, rather than changes in metabolite transporters.

Therefore, we propose that the heightened sensitivity of PMN-MDSCs to ornithine and spermine stems from the innate, enzyme-driven capacity to rapidly process these specific metabolites, granting them a competitive advantage in metabolizing niche resources.

(Page 15, line 400-404)

"We found that PMN-MDSCs exhibited a significant enrichment in amine and polyamine metabolism pathways compared to other cell types, indicating a metabolic reprogramming that renders them particularly dependent on this pathway to fuel their rapid expansion and functional activity (Supplementary Fig. 10A)."

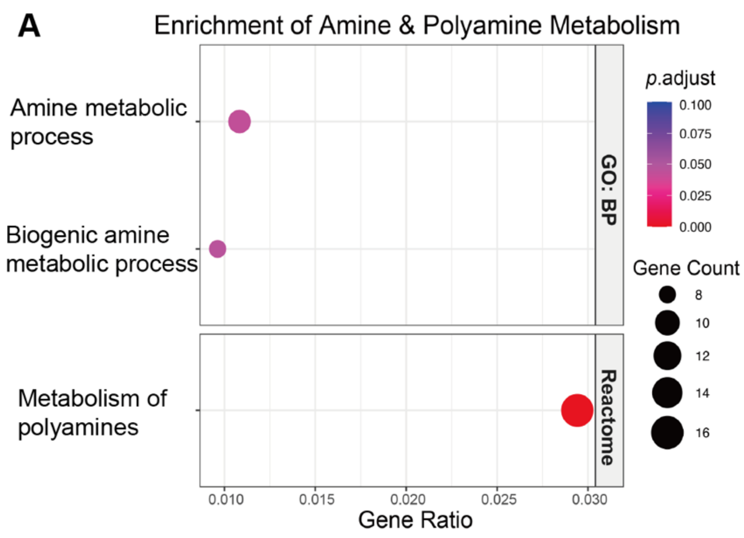

Supplementary Fig. 10 ARG2 and SAT1 are highly expressed in PMN-MDSCs from ARID1A^{KO} group. *(A) Enrichment of amine and polyamine metabolism in PMN-MDSCs.*

5. Have the authors tried to combine ornithine and spermine treatments? Same question for the inhibitors, what is the consequence of dual inhibition?

We thank the reviewer for raising the insightful point regarding the investigation of combined treatments. Accordingly, we examined both metabolite supplementation and inhibitor blockade in combination. To assess potential interactions, we employed two-way ANOVA analysis. For the metabolites, a significant interaction was observed between ornithine and spermine (p<0.0001). However, their combined administration induced only a modest increase in bone marrow PMN-MDSCs compared to spermine alone, while remaining comparable to ornithine alone. This result suggests that the pathway for PMN-MDSC induction could be saturated by metabolic supply. Furthermore, the combination of CB-1158 and DFMO demonstrated greater efficacy in reducing bone metastasis incidence. Specifically regarding PMN-MDSC reduction,

while no significant interaction was found (p=0.3035), the combination was nonetheless more effective than either agent alone, suggesting an additive rather than a synergistic effect. Taken together, these findings suggest that the pathway driving PMN-MDSC induction can be saturated by metabolic supply; and dual inhibitors achieves additive efficacy in lowering both bone metastasis incidence and PMN-MDSC proportion. These results have been added to the revised manuscript (Page 16, line 435-440 and Page 19, line 508-513) .

(Page 16, line 435-440).

"To further explore the combined effect of ornithine and spermine on promoting PMN-MDSCs, we utilized an intracardiac injection model (Fig. 6J). Although a significant interaction was observed between ornithine and spermine ($p<0.0001$). However, we found that their combined administration induced only a modest increase in bone marrow PMN-MDSCs compared to spermine alone, while remaining comparable to ornithine alone. (Fig. 6K)."

REDACTED

Fig 6. Ornithine and spermine secreted by cancer cells expand PMN-MDSCs. ***(J)*** *Mouse models were established by intracardiac injection of wild-type 4T-1 cells, and then subjected to exogenous supplementation with ornithine, spermine, or their*

combination. ***(K)*** *PMN-MDSC proportions in bone marrow of each group. n=5 per group, two-way ANOVA for multiple comparisons. The interaction between the two factors was significant ($p<0.0001$), other p values are indicated in the figures. Source data are provided as a Source Data file.*

(Page 19, line 508-513).

"We next assessed the potential interaction between CB-1158 and DFMO (Fig. 7J). Their combination demonstrated greater efficacy in reducing bone metastasis incidence (Figs. 7K and L). For the reduction of PMN-MDSCs, although no significant interaction was detected ($p=0.3035$), the combination was more effective than either agent alone, suggesting an additive rather than a synergistic effect (Fig. 7M)."

REDACTED

Fig 7. ARG2 and ODC1 inhibitors suppress ARID1A-deficient TNBC bone metastasis. ***(J)*** *Schematic diagram illustrating the experimental design, including drug*

*administration schedules. bid: twice daily; qd: once daily. (**K**) BLI and H&E staining images of mice in each group with intracardiac injection. Scale bar, 200μm. B bone, T tumor. (**L**) Statistical charts presenting the bone metastasis incidence in each group with intracardiac injection. n=5 per group, Fisher's exact test was used. (**M**) Bone marrow of mice was harvested from each group with intracardiac injection, and analyzed by flow cytometry to quantify PMN-MDSC proportions. n=5 per group, statistical analysis by two-way ANOVA showed no significant interaction between treatments (p=0.3035), other p values are indicated in the figures.*

Minor:

1. In the ctDNA analysis, are mutations in any other SWI/SNF subunits detected? As mentioned by the authors, almost 10% of all cancers have mutations in ARID1A, so detecting 53/663 in this cohort does not appear as a strong enrichment.

Is the mechanism described in this study specific to the ARID1A subunit or a more general SWI/SNF function?

Thank you for this insightful question regarding mutations in other SWI/SNF subunits within our ctDNA cohort. In our ctDNA cohort, mutations in other SWI/SNF subunits were detected at the following frequencies: SMARCA4 in 20 patients (3.02%), SMARCB1 in 3 (0.45%), ARID1B in 14 (2.11%), ARID2 in 14 (2.11%), and BRD7 in 2 patients (0.30%). Additionally, none of these alterations in other SWI/SNF subunits showed a statistically significant correlation with bone metastasis. These results have

been presented as supplementary Table 2, with corresponding descriptions added to the Results section (Page 6, line 133-138).

As demonstrated by our results with the SWI/SNF inhibitor, BRM014 treatment did not significantly alter bone metastasis incidence and PMN-MDSC proportion in *Arid1a*^{NC} mice but suppressed them in *Arid1a*^{KO} mice. This indicates that the pro-metastatic state induced by ARID1A deficiency remains dependent on residual SWI/SNF complex activity, the inhibition of SWI/SNF alone fails to replicate the ARID1A deficiency, implicating additional ARID1A-specific roles in tumor progression. This specificity is further supported by clinical ctDNA data, which reveal that bone metastasis is uniquely associated with ARID1A loss rather than mutations in other SWI/SNF subunits.

In addition, thank you for raising the important question regarding the proportion of ARID1A mutations. The figure stating that ARID1A mutations are "present in approximately 10% of tumors" actually represents a pan-cancer average, the frequency in breast cancer is specifically about 7% (Guan-Tian Lang et al., Nat Commun. 2020 Nov)[5]. We have added it to the revised manuscript (page 4, line 67).

(Page 6, line 133-138)

"We also detected mutations in additional SWI/SNF subunits in our ctDNA cohort, with the following frequencies: SMARCA4 in 20 patients (3.02%), SMARCB1 in 3 (0.45%), ARID1B in 14 (2.11%), ARID2 in 14 (2.11%), and BRD7 in 2 patients (0.30%). Notably,

none of these mutations showed a statistically significant correlation with bone metastasis (Supplementary Table 2).”

Supplementary Table 2. Mutated SWI/SNF subunits in the ctDNA cohort.

Mutated SWI/SNF subunits (Total=663)	***Bone metastasis***	***No bone metastasis***	***p value[a]***
ARID1A (n=53)	*34*	*19*	*0.0081*
ARID1B (n=14)	*6*	*8*	*0.7675*
ARID2 (n=14)	*7*	*7*	*0.8058*
SMARCA4 (n=20)	*11*	*9*	*0.4531*
SMARCB1 (n=3)	*3*	*0*	*0.0640*
BRD7 (n=2)	*2*	*0*	*0.1307*

[a] p values were determined using the Pearson's chi-square test.

(Page 4, line 67)

“ARID1A mutations, present in approximately 10% of tumors (about 7% in breast cancer)[7], predominantly consist of inactivating mutations that drive loss of ARID1A protein, with its deficiency conferring poor clinical outcomes across multiple cancer types[8,9].”

2. For the engineering of the Arid1a KO and OE cell lines, more details are required.

While the cellular phenotypes are striking, could the authors comment on the incomplete nature of the knock-out as well as how they overexpressed such a large protein using viruses (dCas9 approach?).

We thank the reviewer for raising the point regarding the the engineering of the *Arid1a*^{KO} and *Arid1a*^{OE} cell lines. As suggested, more detailed descriptions of the *Arid1a*^{KO} and *Arid1a*^{OE} cell line generation have been added to the revised manuscript (Page 26, line 714-737). Regarding the incomplete *Arid1a* knockout observed by western blot, we appreciate the reviewer's attention to this technical detail. This is a common outcome in CRISPR/Cas9 editing of large genes such as ARID1A (240 kDa). Following DNA cleavage, repair via non-homologous end joining (NHEJ) can generate in-frame indels, potentially allowing the expression of a truncated protein that may still be detected by antibodies targeting upstream epitopes. Additionally, even after selection, the polyclonal population may contain a minor fraction of incompletely edited cells, which could contribute to a faint residual signal in western blot. Furthermore, the observed phenotypic differences between *Arid1a*^{NC} and *Arid1a*^{KO} cells support that the *Arid1a*^{KO} cell line is a valid loss-of-function model for this study. To overexpress ARID1A, we utilized a CRISPR activation (CRISPRa) approach via the synergistic activation mediator (SAM) system. This system is based on a deactivated Cas9 (dCas9) platform.

(Page 26, line 714-737)

"Stable transfection using lentiviral infection

To generate ARID1A^{KO} cells, a lentiviral CRISPR/Cas9 system was employed. The Lenti-CAS9-Puro vector and the single-guide RNA (sgRNA) expression vector GV371-EGFP (Shanghai Genechem, Shanghai, China), carrying Cas9 and an ARID1A-targeting guide RNA respectively, were co-transfected with packaging plasmids into HEK293T cells using Lipofectamine 3000 (L3000-150, Invitrogen) to generate lentiviral particles. For the construction of ARID1A^{OE} cells, aCRISPR/Cas9 activation (CRISPRa) approach was employed using thesynergistic activation mediator (SAM) system (Genechem). This system is based on a deactivated Cas9 (dCas9) platform. The SAM vector was programmed with a specific sgRNA targeting the promoter region of the ARID1A and the empty vector was used as negative control. The vector was similarly co-transfected with packaging plasmids into HEK293T cells for lentivirus production. For both KO and OE constructions, the virus-containing supernatant was collected 72h after incubation, filtered and used to transduce cells in the presence of polybrene (5 μg/mL). To establish stable polyclonal populations, successfully transduced cells were selected with puromycin, with the antibiotic concentration optimized for the specific cell line. The primers used for plasmid construction were listed in Supplementary Table 7. The ARID1A knockout and overexpression efficiency was confirmed via western blotting."

3. The ChIP-seq dataset GSE98765 does not link to the study by Chen et al.

Thank you for pointing this out. We have carefully checked and corrected it to the accurate dataset citation (GSE234179).

Reviewer #2 (Remarks to the Author); expert in breast cancer, bone metastasis and tumour microenvironment:

This manuscript investigates an important topic that is gaining considerable interest of metabolic crosstalk between cancer cells and immune cells. This is a complex and nuanced topic, and the current study attempts to understand the important question of what tumour mutations change tumour cell metabolism to promote bone marrow metastasis tropism. Particularly important to note is that there is currently no effective/ targeted treatment option for patients with breast cancer bone marrow metastasis. This underscores the importance of the work. Whilst this is an interesting and important topic, the manuscript in its current form lacks major controls and overstates the data making it very hard to interpret.

Unfortunately, there are no good models of breast cancer -> bone metastasis. This project relies on cell line injection models and the limitations of this should be discussed.

Thank you for raising this important point regarding the limitations of our experimental models. We have incorporated the discussion on the limitations of the models into the Discussion section (Page 21, line 566-573).

(Page 21, line 566-573)

“We established models of bone metastasis in mice by performing intracardiac and intratibial injections with luciferase-expressing TNBC cells, and monitored tumor burden in vivo using BLI. It is important to acknowledge that these experimental models bypass the critical early stages of the metastatic cascade, including primary tumor development and spontaneous dissemination, thus limiting their ability to fully recapitulate the natural progression of breast cancer to bone. Future studies employing more physiologically relevant, spontaneous metastasis models will be necessary to better mimic the clinical course of the disease.”

In the introduction, the authors define PMN-MDSCs as CD11b+Ly6G+Ly6C-low. This is precisely the same definition as a neutrophil and highlights the problem with MDSC nomenclature. A fundamental problem with using MDSC nomenclature is that it treats PMN-MDSCs as distinct from neutrophils and M-MDSCs as distinct from monocytes, when in fact recent work has demonstrated they represent functional states of neutrophils and monocytes. Labeling them as a separate category obscures their true lineage and hampers our ability to understand their biology in the broader context of myeloid cell plasticity.

Thank you for raising this very important and insightful point regarding the definition of PMN-MDSCs. We agree that the nomenclature and the relationship between PMN-MDSCs and classical neutrophils represent a key and actively debated issue in the field of tumor immunology. We agree with the growing consensus, as noted by the reviewer, that PMN-MDSCs are better understood as neutrophils that have acquired a potent

immunosuppressive phenotype under the sustained pressure of the tumor microenvironment, sometimes termed "pathologically activated" or "granulocytic" myeloid-derived suppressor cells.

In our study, we employed the operational definition of murine PMN-MDSCs as $CD11b^{+}Ly6G^{+}Ly6C^{low}$ cells isolated from tumor-bearing hosts. This definition remains the established standard in the field to identify and interrogate the granulocytic population responsible for immunosuppression in cancer, allowing for comparison with a vast body of prior work (Ni Li et al., Nat Commun. 2022 Nov; Filippo Veglia et al., Nat Rev Immunol. 2021 Aug; Rui Zhang et al., Signal Transduct Target Ther. 2023 Mar)[4, 6, 7]. Our study was primarily designed to investigate the functional contribution of this immunosuppressive granulocytic subset to bone metastasis in ARID1A-deficient breast cancer. Using the term "PMN-MDSC" most precisely conveys to readers that we are focusing on this functionally defined immunosuppressive state, rather than making a claim about its ontological distinction from neutrophils.

We appreciate the importance of not overlooking this conceptual nuance. Therefore, in the revised Discussion section, we have added a dedicated paragraph to clarify this scope (Page 22, line 598-605). We acknowledge the evolving understanding that these cells likely represent a functional state within the spectrum of neutrophil plasticity and cite key literature supporting this view, placing our findings within this broader conceptual framework.

(Page 22, line 598-605)

"PMN-MDSCs, also known as pathologically activated neutrophils, are a critical component of the TME and play crucial roles in tumour progression and therapy resistance[54-56]*. As defined by the phenotypic markers CD11b^{+}Ly6G^{+}Ly6C^{low}, PMN-MDSCs are better understood as a functional immunosuppressive state acquired by neutrophils, under specific tumor microenvironmental pressures*[53]*. We revealed a critical immunosuppressive functional state of neutrophils within the ARID1A deficient bone metastatic niche which underscores the dynamic nature of myeloid cells in cancer."*

Injection of luciferase labelled cells: allows to follow tumour cells but Luciferase is not naturally expressed in mammalian tissues; therefore, introduction of luciferase-expressing cells provides a neoantigen that is recognised as foreign by the host immune system. This can elicit inflammatory responses, contributing to the overall immunogenicity of the injected cell line, and skewing readouts of inflammatory cells. The methods are not very detailed and a lot of experimental details that are required to understand the data are missing. For example, tumour cell growth after manipulation, size of tumours at euthanasia (is it the same between experimental groups?), Figure 5, is is not clear which model the experiments are performed in.

We appreciate the reviewer for these insightful comments, which have helped strengthen the manuscript. In response, we have made the following key revisions:

1. We thank the reviewer for raising this important methodological point regarding the potential immunogenicity of luciferase. We fully acknowledge that the

expression of this non-mammalian protein could elicit local immune responses. Nonetheless, luciferase-based bioluminescence imaging remains an established method for the longitudinal and quantitative tracking of metastatic burden in mouse models. To address this consideration, we have incorporated a statement in the Discussion section (Page 21, line 573-578) acknowledging this methodological constraint while underscoring the utility of the approach for in vivo tracking.

2. We thank the reviewer for highlighting the need for greater methodological detail. In response, we have thoroughly revised the Methods section to provide a comprehensive description of the experimental procedures and details.
3. The legend for Figure 5 has been revised to explicitly specify the experimental model (intracardiac vs. intratibial injection) used for each panel, eliminating any ambiguity.

(Page 21, line 573-578)

"Additionally, while the luciferase reporter is essential for in vivo imaging, its expression may introduce immunogenic effects that could influence tumor behavior and the host microenvironment[48]*. Despite this potential constraint, luciferase-based imaging remains an effective method for longitudinally tracking metastatic progression in preclinical bone metastasis research*[29,31,49]*."*

*"**Fig 5. ARID1A deficiency drives the expansion of PMN-MDSCs.** (**A**) Proportions of human PMN-MDSCs after in vitro co-culture with ARID1A^{NC} and ARID1A^{KO} MDA-MB-231 cells. n=5 per group, unpaired two-tailed Student's t test. (**B**) Proportions of PMN-MDSCs in the bone marrow of Arid1a$^{flox/flox}$;Mmtv-Cre mice compared to Mmtv-Cre control mice. n=6 per group, unpaired two-tailed Student's t test. (**C**) Bone marrow was harvested from **Balb/c mice following intracardiac injection of Arid1a^{NC} and Arid1a^{KO} 4T-1 cells**, and analyzed by flow cytometry to quantify PMN-MDSC proportions. n=7 per group, unpaired two-tailed Student's t test. (**D**) PMN-MDSC proportions in bone metastatic tumors from **Balb/c mice following intratibial injection of Arid1a^{NC} and Arid1a^{KO} 4T-1 cells.** n=5 per group, unpaired two-tailed Student's t test. (**E-F**) The percentage of CD11b^{+}Ly6G^{+} cells in bone metastatic tumors from **Balb/c mice following intratibial injection of Arid1a^{NC} and Arid1a^{KO} 4T-1 cells** was quantified by IF staining, with quantitative analysis between experimental groups. n=3 per group, unpaired two-tailed Student's t test. Scale bar, 20μm. (**G-H**) The proportion of PMN-MDSCs in tumor tissues from TNBC patients with high versus low ARID1A expression was assessed by TSA staining on tissue microarrays, followed by quantitative comparison. n=40 per group, unpaired two-tailed Student's t test. Scale bar, 20μm. All p values are indicated in the figures. Source data are provided as a Source Data file."*

Can we assume the MDA-MB-231 would behave the same way as 4T1 in terms of bone marrow metastasis?

We thank the reviewer for this comment. In our experiments, intracardiac injection of luciferase-labeled ARID1A^{NC} and ARID1A^{KO} MDA-MB-231 cells into female NSG mice that has been humanized with human CD34+ hematopoietic stem cells revealed that these cells exhibited a comparable pattern of bone metastasis to 4T-1 cells. These results are now included as Figure 2D and G, with the corresponding descriptions in the revised manuscript (Page 7, line 169-172).

(Page 7, line 169-172)

"Similarly, in NOD-scid IL2Rγnull (NSG) mice humanized with human CD34+ hematopoietic stem cells and injected with MDA-MB-231 cells, the incidence of bone metastasis was significantly greater in the KO group compared with the NC controls (Fig. 2G)."

REDACTED

Fig 2. ARID1A deficiency promotes bone metastasis of breast cancer. (D) *Representative BLI, H&E staining images of two groups of mice following intracardiac injection with equal numbers of ARID1A*^{NC} *and ARID1A*^{KO} *MDA-MB-231 cells. Scale bar, 200μm. n=6 per group.* ***(G)*** *Following intracardiac injection of MDA-MB-231 cells, bone metastasis was detected in 5 mice (5/6) in the ARID1A*^{KO} *group, whereas no metastasis (0/6) was observed in the ARID1A*^{NC} *group. n=6 per group, Fisher's exact test was used.*

Manipulations of Arid1 – do tumours and cell lines grow at the same rate upon Arid1-

ko? Throughout the manuscript, we cannot understand if the results are a function of faster tumour growth / bigger tumours.

We thank the reviewer for raising this critical question. To clarify, the observed pro-metastatic phenotype is not attributable to a general increase in tumor growth rate or size upon ARID1A deficiency. Our integrated experimental data support a model in which ARID1A deficiency reshapes the immune microenvironment to drive metastasis. The key evidence is as follows:

1. **No intrinsic proliferation difference in vitro:** To address the possibility of cell-autonomous growth differences, we performed comprehensive in vitro assays. Colony formation and CCK-8 viability assays using multiple cell lines (4T-1, MDA-MB-231, BT-549) demonstrated that ARID1A knockout does not confer a significant intrinsic proliferative advantage under standard culture conditions. These results are now included as supplementary Figs. 11A and B, with the corresponding descriptions in the revised manuscript (Page 17, line 449-452).
2. **The crucial role of PMN-MDSCs:** To directly test the core role of PMN-MDSCs in this process, we performed in vivo depletion using an anti-Ly6G antibody. The depletion of PMN-MDSCs completely abolished the bone metastatic advantage of the $Arid1a^{KO}$ group compared to the $Arid1a^{NC}$ group. These findings demonstrate PMN-MDSC as a key mediator of bone metastasis in ARID1A-deficient TNBC (Supplementary Figs. 11B-D), with the corresponding descriptions in the revised manuscript (Page 17, line 453-458).

In summary, our data collectively demonstrate that ARID1A deficiency drive bone metastasis by reshaping the tumor immune microenvironment, particularly by enhancing the recruitment and utilization of PMN-MDSCs, thereby conferring a growth and metastatic advantage supported by these immune cells.

(Page 17, line 449-452 and Page 17, line 453-458)

"To further validate the critical role of PMN-MDSCs in ARID1A deficiency-mediated bone metastasis, we examined whether the proliferative capacity differed between ARID1A^{NC} and ARID1A^{KO} cells. We found that ARID1A^{NC} and ARID1A^{KO} cells exhibited comparable proliferative capacities in vitro (Supplementary Figs. 11A and B). Given that the intrinsic growth of tumor cells was unaffected, we administered anti-Ly6G antibody to delete PMN-MDSCs in mice[41]. In the intracardiac injection model established with Arid1a^{NC} or Arid1a^{KO} cells, anti-Ly6G antibody treatment significantly reduced bone metastatic incidence in Arid1a^{KO} group (Supplementary Figs. 11C-E). These findings demonstrate PMN-MDSC as a key mediator of bone metastasis in ARID1A-deficient TNBC."

REDACTED

Supplementary Fig. 11 PMN-MDSCs play a critical role in ARID1A deficiency-mediated bone metastasis. *(**A**) CCK-8 assays of TNBC cells (4T-1, MDA-MB-231, BT-549) between* $ARID1A^{NC}$ *and* $ARID1A^{KO}$ *group. n=7, two-way ANOVA was used. (**B**) Clone formation assays of TNBC cells (4T-1, MDA-MB-231, BT-549) between* $ARID1A^{NC}$ *and* $ARID1A^{KO}$ *group. n=3, unpaired two-tailed Student's t test. (**C**) Treatment schedule schematic. Following intracardiac injection of* $Arid1a^{NC}$ *and* $Arid1a^{KO}$ *4T-1 cells, mice were allocated to receive either anti-Ly6G antibody or isotype control IgG. (**D**) BLI and H&E staining images of mice in* $Arid1a^{NC}$ *plus IgG,* $Arid1a^{NC}$ *plus αLy6G,* $Arid1a^{KO}$ *plus IgG，* $Arid1a^{NC}$ *plus αLy6G group. Scale bar,*

*200μm. B bone, T tumor. n=5 per group. (**E**) Statistical charts presenting the bone metastasis incidence in each group. n=5, data was analyzed by Fisher's exact test. p values are indicated in the figures.*

Figure 4E – in the feature plots, do these cells come from mice injected with Arid1NC or Arid1ko? What is the difference between neutrophil and a PMN-MDSC. As mentioned above, recent publications have indicated that these are not separate entities, but a functional continuum impacted by the systemic environment driven by the tumour.

Thank you for this critical and insightful question. All cells presented in Figure 4E were isolated from the bone marrow of mice injected with either *Arid1a*^{KO} or *Arid1a*^{NC} tumor cells. The plots were generated from the aggregated single-cell data of this mixed population, aiming to visualize different cell types expressing characteristic markers. We fully agree with the reviewer that current evidence supports viewing PMN-MDSCs not as a distinct lineage, but as a functional immunosuppressive state acquired by neutrophils under tumor-derived pressures. In Figure 4E, cells expressing high levels of the canonical granulocyte marker Ly6G were annotated as Neutrophils. Within that broader population, the subset that expressed high levels of IL-1β was annotated as PMN-MDSCs. This annotation, consistent with studies like Alshetaiwi et al., (Alshetaiwi et al., Sci. Immunol. 2020)[3] was used to identify and focus on that specific neutrophil subpopulation exhibiting a pronounced immunosuppressive phenotype within the bone marrow microenvironment shaped by ARID1A-deficient tumors. Our

emphasis was on revealing this acquired functional state, its role in promoting bone metastasis, and its regulatory mechanisms, rather than on lineage distinctions. Therefore, in the revised Discussion section, we have added a dedicated paragraph to clarify this scope (Page 22, line 598-605). We thank the reviewer again for this constructive comment, which has significantly strengthened the rigor of our study.

(Page 22, line 598-605)

"PMN-MDSCs, also known as pathologically activated neutrophils, are a critical component of the TME and play crucial roles in tumour progression and therapy resistance[54-56]. As defined by the phenotypic markers CD11b^{+}Ly6G^{+}Ly6C^{low}, PMN-MDSCs are better understood as a functional immunosuppressive state acquired by neutrophils, under specific tumor microenvironmental pressures[53]. We revealed a critical immunosuppressive functional state of neutrophils within the ARID1A deficient bone metastatic niche which underscores the dynamic nature of myeloid cells in cancer."

Figure 4F – using pseudotime trajectories in this manner is increasingly common but it is a fundamentally flawed methodology. In the case of neutrophils and PMN-MDSCs, using pseudotime is problematic because these populations are highly plastic and can interconvert depending on environmental cues. Without a clear, unidirectional differentiation pathway, the algorithm may infer a trajectory that assumes PMN-MDSCs arise from neutrophils (or vice-versa), even though the real biology may reflect activation or functional reprogramming rather than a linear differentiation process. In

addition, the authors use “Pre-MDSC” but do not define the population. Further, Monocle relies heavily on dimensionality reduction and the construction of a minimum-spanning tree, which means the inferred trajectory depends strongly on how the algorithm embeds the data and on the choice of root cell. If the starting point is mis-specified, the directionality of the pseudotime is effectively arbitrary and the “differentiation path” can be a computational artifact rather than a true biological progression. Given that the starting point is not defined by the authors nor is what “early myeloid”, “pre-MDSC”, this data is impossible to interpret.

We sincerely thank the reviewer for these insightful and critical comments regarding our use of pseudotemporal trajectory analysis. We fully acknowledge and appreciate the fundamental methodological limitations raised, particularly concerning cell plasticity and the interpretative challenges of such computational approaches. Our goal was not to assert a rigid, unidirectional differentiation pathway but to use this as an exploratory tool to interrogate whether ARID1A deficiency is associated with a transcriptional shift within the granulocyte continuum toward states enriched in immunosuppressive signatures following reference in the field (Alshetaiwi et al., Sci. Immunol. 2020)[3]. In response to the reviewer’s valid concerns, we have revised the manuscript to present the pseudotime analysis strictly as a descriptive visualization of the developmental progression within granulocytic myeloid states.

The reviewer correctly emphasizes that the interpretation of a trajectory depends critically on the definition of its start and key states. The root of our trajectory was defined as an early myeloid state characterized by high expression of canonical early

neutrophil progenitor markers (*Elane*, *Mpo*, and *Prtn3*), a strategy consistent with prior work in the field (Alshetaiwi et al., Sci. Immunol. 2020). We defined a transitional cell state, which aligns with the Pre-MDSC (G-MDSC transition) state identified by Alshetaiwi et al. This state was specifically identified by elevated expression of *Retnlg*, *Pirb* and *Asprv1*. These definitions and marker expression patterns are now detailed in the revised manuscript (Page13, line 334-341) and presented in Supplementary Figures 6A-C. Furthermore, we performed an independent, complementary analysis using Single-Cell Velocity (scVelo). This method infers directional dynamics directly from spliced/unspliced mRNA ratios. Critically, scVelo reconstructed a consistent progression from the early state toward the immunosuppressive granulocyte state (Supplementary Fig. 6D).

We sincerely appreciate the reviewer's expertise, which have significantly improved the rigor and clarity of our manuscript.

(Page13, line 334-341)

"The trajectory was rooted in early myeloid cells, defined as neutrophil progenitors expressing high levels of progenitor markers such as Elane, Mpo, and Prtn3 (Supplementary Fig. 6A). The Pre-MDSC represents a transitional cell type within the granulocytic transcriptional continuum, marked by high expression of Asprv1, Pirb and Retnlg (Supplementary Fig. 6B). PMN-MDSC were characterized as a functional immunosuppressive state acquired by neutrophils under specific tumor

microenvironmental pressures, defined by high expression of Cd84, Arg2, Il1b, and Spi1 (Supplementary Fig. 6C). "

*"To further validate this finding, we performed an independent analysis using Single-Cell Velocity (scVelo), a method that infers directionality in cellular state transitions. ScVelo consistently reconstructed the same developmental trajectory (*Supplementary Fig. 6D*)."*

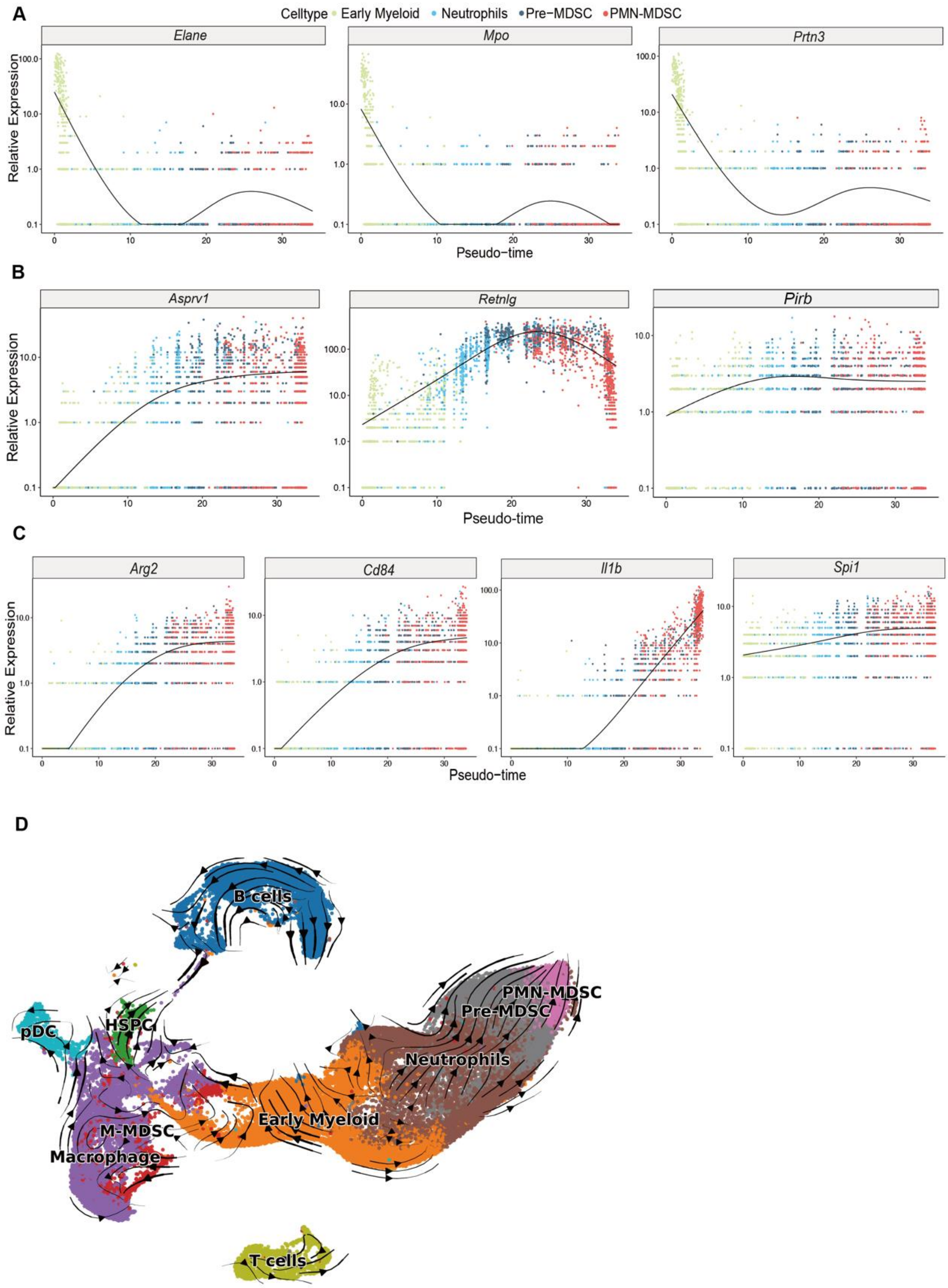

Supplementary Fig. 6 The developmental trajectory of PMN-MDSCs in TNBC. *(A) Early myeloid cells were marked by high expression of Elane, Mpo, and Prtn3. (**B**) Pre-MDSCs were marked by high expression of Asprv1, Pirb and Retnlg. (**C**) PMN-MDSCs were marked by high expression of Cd84, Arg2, Il1b, and Spi1. (**D**) ScVelo analysis of*

the developmental trajectory within granulocytic myeloid clusters.

The gating and identification of human PMN-MDSCs in figure 5A / S7B is flawed. The gating strategy appears to take a lot of CD11b-cells. Furthermore, CD66 gate looks predominantly like boarder effect/ spread. FMOs are required. As it stands, this is not convincing that there is an increase in PMN-MDSCs.

We thank the reviewer for this detailed and important technical critique regarding the gating strategy for human PMN-MDSCs. In response to the reviewer's point, we performed the recommended FMO controls. The CD11b FMO confirmed our gating strategy was appropriate. Regarding CD66b, the FMO control confirmed the presence of a positive population, rather than non-specific border spread. Based on this reference, we conservatively adjusted the positivity gate downward to more accurately capture this population. We then repeated the key in vitro functional assays for human PMN-MDSCs using this updated gating strategy. Reassuringly, the results from these repeated experiments were fully consistent with our original observations, corroborating the initial biological conclusion. All relevant figures (Fig. 5A; Fig. 6D; Fig. 7F) have been updated in the revised manuscript. The complete gating strategy, including FMO controls, is provided in Supplementary Figure 8.

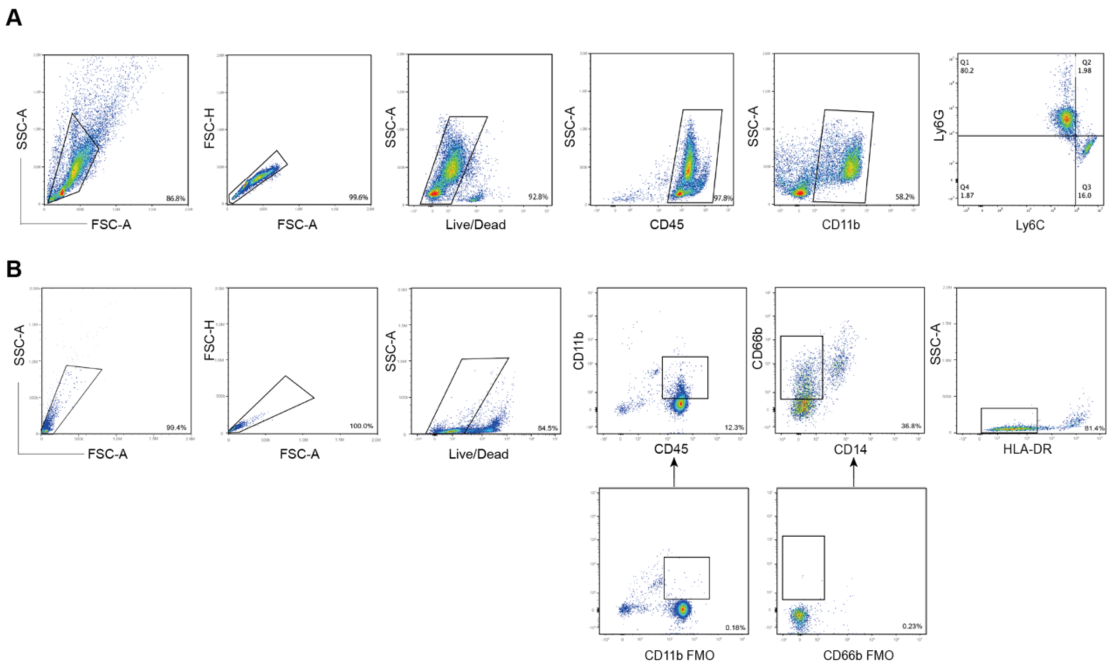

Supplementary Fig. 8 Gating strategies for isolating PMN-MDSCs. *(A) Gating logic for isolating PMN-MDSCs of mice.* ***(B)*** *Gating logic for isolating PMN-MDSCs of humans.*

Mtmv-Cre mice experiments: was there any difference in primary tumour growth between Arid1-ko and Arid1-wt tumours? Were mice euthanized at the same time point (but potentially with different tumour sizes) or at the same tumour size?

We thank the reviewer for this critical question regarding primary tumor growth. In the MMTV-Cre model used in our study, tumors are chemically induced and develop asynchronously. Consequently, to ensure an equivalent endpoint tumor burden for comparative analysis, mice were euthanized individually once their primary mammary tumor reached a predetermined ethical size threshold. The detailed endpoint criteria are provided in the Methods section of the revised manuscript (Page 28, line 783-785).

(Page 28, line 783-785)

"Mice were then euthanized when the primary tumor reached a predefined humane endpoint size to ensure comparable tumor burden at analysis and bone marrow was collected for flow cytometric analysis."

Figure 5C-F - the authors indicate that they "replicate the intracardial injection model" but it is not clear with which cells into which mice therefore the data are uninterpretable. Similarly, for the intratibial injection.

Thank you for this comment. As detailed above**,** the legend for Figure 5 has been revised to explicitly specify the experimental model (intracardiac vs. intratibial injection) used for each panel, eliminating any ambiguity.

Do the tumour cells grow the same in the presence of CB-1158 and DFMO? Do they curtail PMN-MDSCs because the tumour cells die? Perhaps a reductionist / in vitro co-culture experiment is required to fully understand how these interact and to be sure that the effect of CB-1158 and DFMO are not indirect.

Thank you for raising this important point regarding the mechanism of action of these inhibitors. To assess whether CB-1158 and DFMO exert cytotoxic effects on tumor cells, we performed CCK-8 viability assays on TNBC cells (4T-1 and MDA-MB-231) treated with CB-1158 or DFMO. We found that neither CB-1158 nor DFMO showed significant direct cytotoxic effects on these cell lines (Figs. 7A-D). Our data support that the anti-tumor efficacy of these agents is not mediated by direct tumor cell killing

but through the inhibition of key metabolic enzymes in the tumor immune microenvironment. As established in the literature, CB-1158 functions as an arginase inhibitor, reducing the production of ornithine (Tharp et al., Nat Cancer. 2024 Jul)[8], while DFMO inhibits ornithine decarboxylase (ODC1), leading to depletion of polyamines such as spermine (Xu et al., Immunity. 2024 Aug)[9]. By depleting these metabolites in the tumor microenvironment, they cripple the immunosuppressive capacity of PMN-MDSCs, thereby unleashing anti-tumor immunity indirectly. Thus, the reduction in PMN-MDSCs observed in vivo is not a consequence of tumor cell death, but rather the direct pharmacological disruption of their metabolic support network. These results have been added in the revised manuscript (Page 18, line 490-493).

(Page 18, line 490-493)

"First, we assessed whether CB-1158 and DFMO exert cytotoxic effects on tumor cells and found that neither CB-1158 nor DFMO exhibited significant direct cytotoxicity against TNBC cells (Figs. 7A-D)."

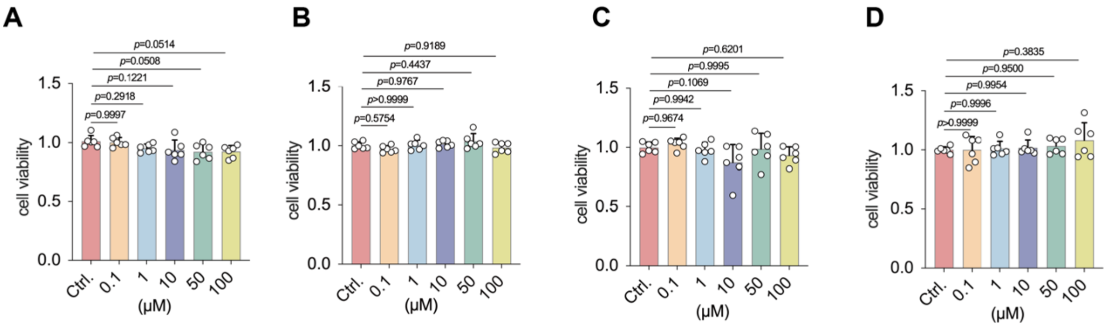

Fig 7. ARG2 and ODC1 inhibitors suppresses ARID1A-deficient TNBC bone metastasis. *(A-B) CCK-8 assays of different concentrations of CB-1158 (A) and DFMO*

(B) on 4T-1 cells for 48h. n=6, per group, one-way ANOVA for multiple comparisons. ***(C-D)*** *CCK-8 assays of different concentrations of CB-1158 (C) and DFMO (D) on MDA-MB-231 cells for 48h. n=6, per group, one-way ANOVA for multiple comparisons.*

Minor comments:

- Figure 1C – no associations with liver, lung, brain or LN mets. Looks like this is because it is underpowered and there could be an association with liver.

We thank the reviewer for this critical comment. To avoid overinterpretation, we have revised the text pertaining to Figure 1C to clarify that ARID1A deficiency did not show a statistically significant correlation with liver, lung, brain, or lymph node metastasis in our study cohort (Page 6, line 129-133). The reviewer's point about potential undetected associations is well-taken and will be noted as a consideration for future studies with larger cohorts.

(Page 6, line 129-133)

"Further, ***no statistically significant*** *associations were observed in the liver (Supplementary Fig. 1A; p=0.0803), lung (Supplementary Fig. 1B, p=0.9222), brain (Supplementary Fig. 1C, p=0.6910), or lymph node metastases (Supplementary Fig. 1D, p=0.5685) between ARID1A wildtype and ARID1A-mutated groups (Supplementary Table 1)."*

- Figure 4D – the text says: "we found the could granulocytic myeloid cells in the

Arid1ko group was significantly elevated compared to that of Arid1NC group". But there is no quantification. T-SNE intensity call also reflect shifts in populations

We thank the reviewer for this comment. We have added the quantitative analysis in the revised manuscript (Page 13, line 327-329).

(Page 13, line 327-329)

"We found that the count of granulocytic myeloid cells in the Arid1a^{KO} group was significantly elevated compared to that in the Arid1a^{NC} group (37.11% vs 40.03%, p<0.001, Fisher's exact test. Fig. 4D), while the other types of cells showed no difference."

Reviewer #3 (Remarks to the Author); expert in cancer metabolism:

Bone metastasis represents a lethal complication of advanced breast cancer. Pan et al. elucidated a novel mechanism for bone metastasis. They found that the AT-rich interactive domain-containing protein 1A (ARID1A) remodeled the immunosuppressive metastatic microenvironment through upregulation of arginine metabolism. In their cohort study involving 663 patients with metastatic breast cancer, circulating tumor DNA (ctDNA) analysis revealed a significant positive correlation between ARID1A mutations and metastatic incidence. Notably, most of these mutations resulted in ARID1A loss-of-function, while ARID1A knockdown in tumor cells markedly activated arginine metabolic pathways—particularly those governing

ornithine and spermine synthesis. The researchers further demonstrated that tumor-derived ornithine and spermine drove the expansion of polymorphonuclear myeloid-derived suppressor cells (PMN-MDSCs) in the bone marrow microenvironment, thereby promoting osteotropic metastasis. Importantly, pharmacological inhibition of key arginine metabolic enzymes—arginase 2 (ARG2) and ornithine decarboxylase 1 (ODC1)—significantly attenuated bone metastasis in preclinical models. The findings provide novel insights into metastatic microenvironment remodeling and open new avenues for preventing bone metastasis in breast cancer patients. However, several concerns should be further addressed:

1. While genomic alterations in ARID1A were linked to metastasis, the study did not explicitly address whether corresponding changes in ARID1A protein expression similarly correlate with metastatic progression. Dose ARID1A protein downregulation in primary tumors or metastatic sites serve as a functional biomarker for, or contributor to, breast cancer bone metastasis?

Thank you for raising this important question regarding the relationship between ARID1A protein expression and bone metastasis. We analyzed ARID1A protein expression using a set of tissue microarray containing samples from 108 TNBC patients. During follow-up, we observed that bone metastasis occurred in 23.5% (12/51) of patients with high ARID1A expression, compared to 49.1% (28/57) of patients with low ARID1A expression. This difference was statistically significant (p=0.0060, chi-square test), supporting the conclusion that downregulation of ARID1A protein is significantly associated with and may promote bone metastasis in TNBC. This result

has been present in Figure 1I, with detailed descriptions on Page 7, line 147-151 in the revised manuscript.

(Page 7, line 147-151)

"To test this hypothesis at the protein level, we analyzed ARID1A expression in a set of tissue microarray (TMA) comprising 108 TNBC patients. Supporting our genetic findings, patients with low ARID1A expression exhibited a significantly higher incidence of bone metastasis (28/57, 49.1%) than those with high expression (12/51, 23.5%, p=0.0060; Fig. 1I)"

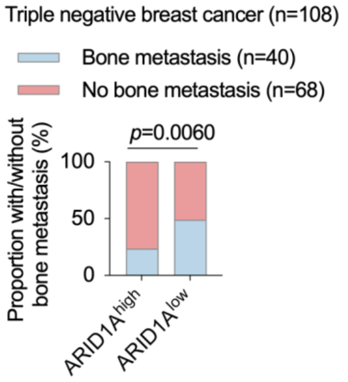

Fig 1. ARID1A mutations promote bone metastasis in breast cancer patients. (I) *The association between ARID1A protein expression levels and bone metastasis in TNBC patients. Pearson's chi-square test was used.*

2. Pan et al. reported an inverse correlation between ARID1A expression and the levels of arginine metabolic enzymes ARG2/ODC1 in metastatic breast cancer. The molecular mechanisms underlying ARID1A-mediated regulation of arginine metabolism were

still unclear. Does ARID1A directly repress ARG2/ODC1 transcription through chromatin remodeling?

Thank you for raising this critical question regarding the molecular mechanism. To investigate the chromatin-based mechanism by which ARID1A regulates arginine metabolism, we performed ATAC-seq and CHIP-seq analysis in ARID1A^{KO} and control MDA-MB-231 cells (GSE234179). Consistent with its role as a core subunit of the canonical BAF (cBAF) complex, ARID1A deficiency led to a global reduction in chromatin accessibility at SWI/SNF-dependent regions. However, at the specific regulatory region of ARG2, H3K27ac CHIP signals were significantly elevated in ARID1A^{KO} MDA-MB-231 cells compared to control cells. Integrated analysis of ChIP-seq and ATAC-seq revealed that ARID1A deficiency facilitates the recruitment of transcription factor ARID3A to the enhancer region within the regulatory domain of ARG2, thereby promoting its expression. The resultant accumulation of ornithine fuels its enzyme ODC1, thereby driving the arginine metabolic pathway. These results are now included as as Figs. 3E-G and supplementary Figs. 4E-I, with the corresponding descriptions in the revised manuscript (Page 9, Line 221-249).

(Page 9, Line 221-249)

"To explore the potential regulatory mechanism underlying low ARID1A expression-induced ARG2 transcription activation, we performed Assay for transposase accessible chromatin (ATAC)-seq and Chromatin Immunoprecipitation-Sequencing (ChIP-seq) analysis in ARID1A^{KO} and negative control MDA-MB-231 cells. Our analyses revealed

that ARID1A deficiency led to a global reduction in chromatin accessibility, indicating impaired SWI/SNF-dependent chromatin remodeling—consistent with ARID1A's established role as a core subunit of the SWI/SNF complex (Supplementary Fig. 4E). Notably, the H3K27ac enrichment at the specific regulatory region of ARG2 was increased by 10-fold in ARID1A^{KO} MDA-MB-231 cells compared with control cells, indicating enhanced transcriptional potential of ARG2 in the absence of ARID1A (Fig. 3E). Subsequent motif enrichment analysis of this differentially accessible region identified ARID3A as a highly enriched candidate transcription factor. To determine its functional role, we conducted siRNA-mediated knockdown of ARID3A, which significantly reduced the expression of ARG2 (Supplementary Figs. 4F and G). This suggests that ARID3A may serve as a key transcriptional regulator mediating the upregulation of ARG2 following ARID1A deficiency. We next performed ChIP-quantitative polymerase chain reaction (CHIP-qPCR) to investigate the binding of ARID3A to the regulatory region of ARG2 and further localized this binding to a specific enhancer site (Fig. 3F). To determine whether this binding site is functionally required for enhancer activity, we cloned the enhancer fragment containing the wild-type (WT) binding site, alongside a corresponding mutant version (MUT) into the pGL3-promoter luciferase reporter plasmid. Dual-luciferase reporter assays revealed that the luciferase activity driven by the enhancer with the WT site was significantly higher than that with the MUT site, demonstrating that this binding site is essential for ARID3A to activate the enhancer of ARG2 (Fig. 3G and Supplementary Fig. 4H). Consistently, we observed that the H3K27ac signal at this specific binding site was

significantly elevated in ARID1A^{KO} cells compared to ARID1A^{NC} cells (Supplementary Fig. 4I).”

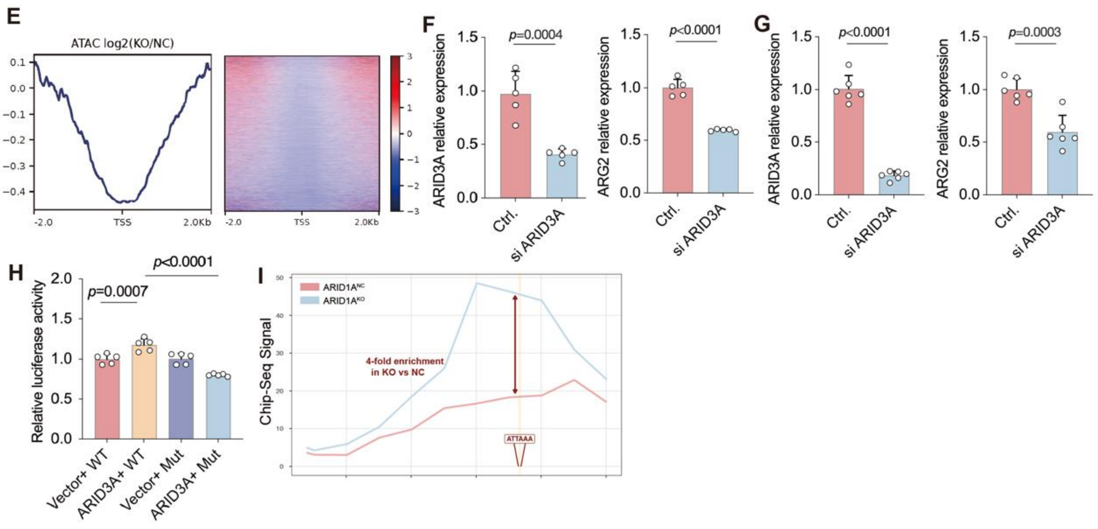

Supplementary Fig. 4 ARID1A-deficient TNBC upregulates the arginine metabolic pathway. *(**E**) ATAC signal at global chromatin accessibility in ARID1A^{KO} cells compared to ARID1A^{NC} cells. (**F**) ARG2 mRNA expression in MDA-MB-231 cells upon siARID3A knockdown. n=5, unpaired two-tailed Student’s t test. (**G**) ARG2 mRNA expression in BT-549 cells upon siARID3A knockdown. n=6, unpaired two-tailed Student’s t test was used. (**H**) Luciferase activity of the ARG2 enhancer upon ARID3A overexpression in BT-549 cells co-transfected with WT or MUT reporter constructs. n=5 per group, one-way ANOVA for multiple comparisons. (**I**) H3K27ac ChIP−seq signal enrichment at the ARID3A-binding site within the ARG2 enhancer in ARID1A^{KO} and ARID1A^{NC} MDA-MB-231 cells.*

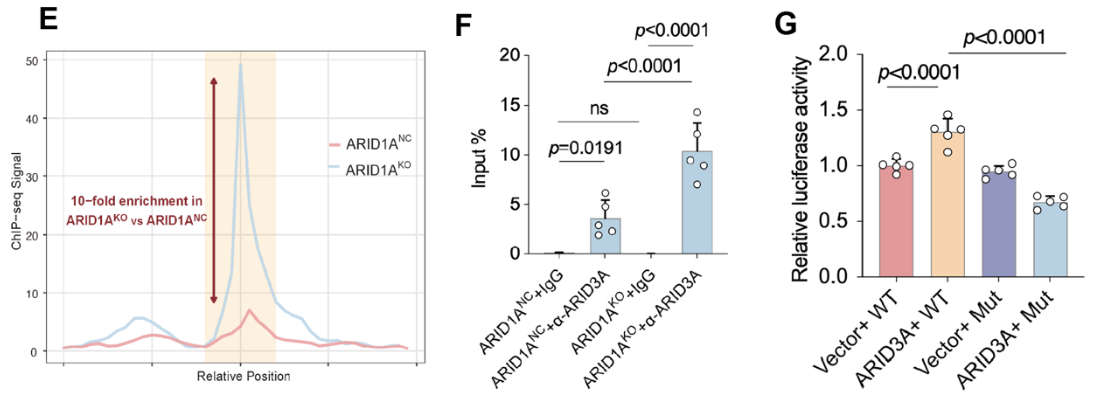

Fig 3. ARID1A deficiency promotes arginine pathway activation and ornithine/spermine production. (E) *H3K27ac ChIP−seq signal enrichment coverage at ARG2 regulatory regionpromoter in ARID1A*^{KO} *and ARID1A*^{NC} *MDA-MB-231 cells.* ***(F)*** *ChIP-qPCR assays of ARID3A binding in the ARG2 enhancer. n=5, two-way ANOVA for multiple comparisons.* ***(G)*** *Luciferase activity of the ARG2 enhancer upon ARID3A overexpression in MDA-MB-231 cells co-transfected with WT or MUT reporter constructs. n=5 per group, two-way ANOVA for multiple comparisons.*

3. The authors showed the expression level of ARID1A is negatively correlated with the production of ornithine/spermine and the expansion of PMN-MDSCs. To further substantiate the clinical significance of these findings, they should validate the relationships between ornithine/spermine levels and metastasis incidence, as well as between ARID1A expression levels and the properties of PMN-MDSCs in breast cancer patients.

Thank you for this valuable suggestion to further substantiate the clinical relevance of our findings. We measured the peripheral blood serum levels of ornithine and spermine in 40 TNBC patients. Patients were stratified into high and low-level groups based on

the median concentration of these metabolites. We found that the incidence of bone metastasis was 15.0% (3/20) in the group with low ornithine levels, compared to 60.0% (12/20) in the group with high levels (p=0.0033, chi-square test). Similarly, it was 10.0% (2/20) in the low spermine group, versus 65.0% (13/20) in the high spermine group (p=0.0003, chi-square test). This suggests that elevated levels of ornithine and spermine are associated with a higher incidence of bone metastasis in TNBC patients. These results have been present in supplementary Figs. 5I and J, with descriptions on Page 12, line 305-311 in the revised manuscript.

Furthermore, we performed multiplex immunofluorescence staining for human PMN-MDSC markers on a set of tissue microarray containing samples from 108 TNBC patients. We observed that tumors with low ARID1A expression contained a significantly higher density of infiltrating PMN-MDSCs compared to those with high ARID1A expression. These results have been present in Figs. 5G and H, with descriptions on Page 15, line 385-390 in the revised manuscript.

(Page 12, line 305-311)

"To dissect the roles of ornithine and spermine in bone metastasis, we measured the peripheral blood serum levels of ornithine and spermine in 40 TNBC patients. Patients were stratified into high and low-level groups based on the median concentration of these metabolites. We found that the incidence of bone metastasis was 15.0% (3/20) in the group with low ornithine levels, compared to 60.0% (12/20) in the group with high

levels (p=0.0033); similarly, it was 10.0% (2/20) in the low spermine group, versus 65.0% (13/20) in the high spermine group (p=0.0003; Supplementary Figs. 5I and J).”

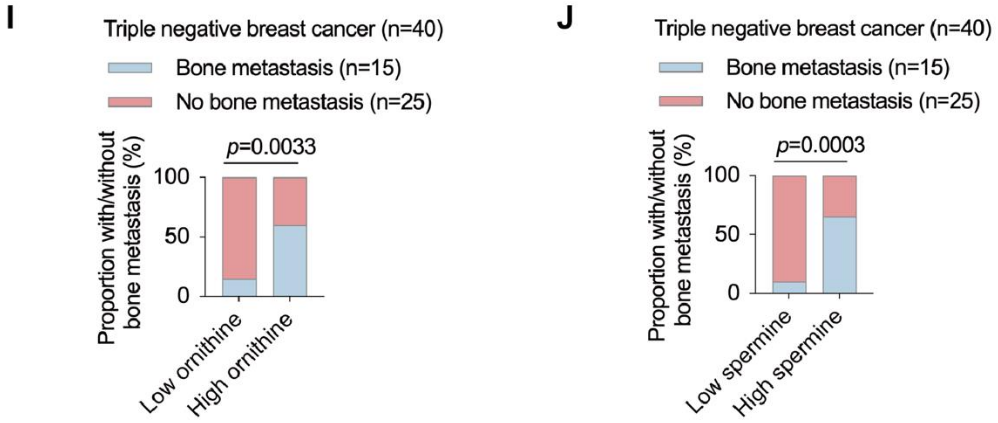

Supplementary Fig. 5 ARID1A deficiency promotes ornithine and spermine production which are associated with bone metastasis in TNBC. (I) *Association between peripheral blood serum ornithine levels and bone metastasis in TNBC patients. n=40, Pearson's chi-square test was used.* ***(J)*** *Association between serum spermine levels and bone metastasis in TNBC patients. n=40, Pearson's chi-square test was used.*

(Page 15, line 385-390)

“Furthermore, to assess the clinical relevance of ARID1A and PMN-MDSCs in breast cancer, we analyzed a set of tissue microarray comprising samples from 108 TNBC patients. Multiplex immunofluorescence staining for human PMN-MDSC markers revealed that tumors with low ARID1A expression exhibited a significantly higher density of PMN-MDSCs compared to those with high ARID1A expressionn (Figs. 5G and H).”

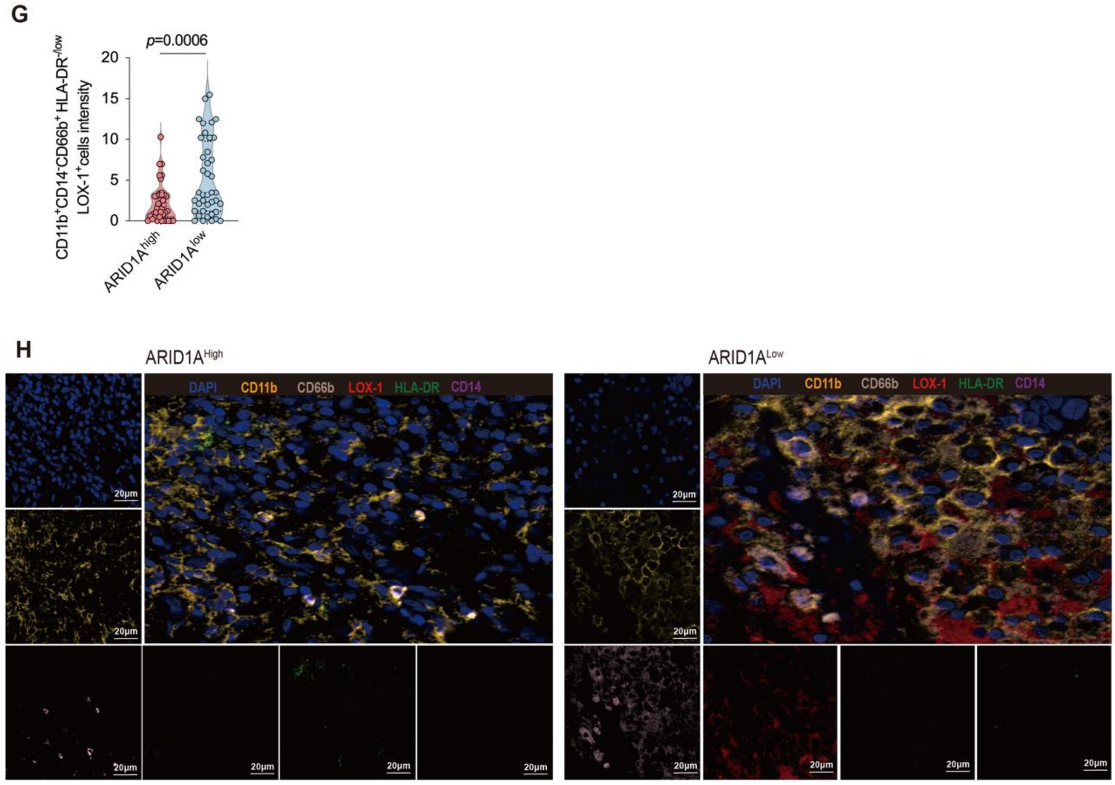

Fig 5. ARID1A deficiency drives the expansion of PMN-MDSCs. (G-H) *The proportion of PMN-MDSCs in tumor tissues from TNBC patients with high versus low ARID1A expression was assessed by TSA staining on tissue microarrays, followed by quantitative comparison. Total n=108, unpaired two-tailed Student's t test. Scale bar, 20μm.*

4. Knockdown of ARID1A or supplementation of ornithine and spermine in the culture medium promotes the expansion of PMN-MDSCs. To further elucidate the underlying mechanism, it is crucial to verify whether ARID1A regulates PMN-MDSCs expansion through the modulation of ornithine and spermine levels, specifically via the enzymes arginase 2 (ARG2) and ornithine decarboxylase 1 (ODC1).

We appreciate the reviewer for this important mechanistic question. Our data collectively demonstrate that ARID1A deficiency drives PMN-MDSC expansion and

bone metastasis specifically by upregulating the ornithine-spermine metabolic axis via ARG2 and ODC1. First of all, integrated ATAC-seq and ChIP-seq analyses (GSE234179) revealed that ARID1A deficiency facilitates the recruitment of transcription factor ARID3A to the enhancer region within the regulatory domain of ARG2, thereby promoting its expression. Second, we confirmed that ARID1A deficiency results in a marked increase in the concentrations of ornithine and spermine. Third, to functionally test the necessity of this pathway, we employed specific enzyme inhibitors. As established in the literature, CB-1158 functions as an arginase inhibitor, reducing the production of ornithine (Tharp et al., Nat Cancer. 2024 Jul)[8], while DFMO inhibits ODC1, leading to depletion of polyamines such as spermine (Xu et al., Immunity. 2024 Aug)[9]. In our ARID1A-deficient models, treatment with either CB-1158 or DFMO alone significantly reduced bone metastasis burden and diminished PMN-MDSC accumulation. Notably, the combination of both agents produced a additive effect, these results have been present in Figs. 7E-M and supplementary Figs. 13A-C (Page 18, line 493-522). These results confirm that the effects of ARID1A deficiency are mediated through these key metabolic enzymes.

(Page 18, line 493-522)

"Then we focused on their potential immunomodulatory roles in the bone metastatic niche and found that CB-1158 and DFMO exhibited a remarkable capacity to curtail the proportions of PMN-MDSCs in both human and murine in vitro systems (Figs. 7E and F). Further intracardiac inoculation of Arid1a^{KO} 4T-1 cells significantly increased

PMN-MDSC accumulation in murine bone marrow compared to controls, whereas treatment with CB-1158 or DFMO effectively reduced PMN-MDSC proportions (Supplementary Fig. 13A). Furthermore, the increased incidence of bone metastasis triggered by Arid1a knockout was significantly mitigated by administering inhibitors targeting ARG2 and ODC1 (Supplementary Figs. 13B and C). In the intratibial bone metastasis model, CB-1158 and DFMO treatment significantly attenuated intratibial tumor progression [tumor volume: NC, 706.10 ± 29.72 mm3; KO, 901.68 ± 152.39 mm3; CB-1158, 699.93 ± 26.34 mm3; DFMO, 651.29 ± 47.78 mm3; tumor weight: NC, 0.77 ± 0.09 g; KO, 0.97 ± 0.17 g; CB-1158, 0.73 ± 0.03 g; DFMO, 0.69 ± 0.05 g; Figs. 7 G-I]. We next assessed the potential interaction between CB-1158 and DFMO. Their combination demonstrated greater efficacy in reducing bone metastasis incidence (Figs. 7K and L). For the reduction of PMN-MDSCs, although no significant interaction was detected (p=0.3035), the combination was more effective than either agent alone, suggesting an additive rather than a synergistic effect (Fig. 7M). Collectively, these data indicate that the inhibition of ARG2 or ODC1, especially that of their combination, resulted in a significant reduction in the proportion of PMN-MDSCs. This, in turn, substantially impaired the progression of bone metastasis, highlighting the pivotal role of these molecular interventions in disrupting the metastatic cascade within the bone microenvironment.

REDACTED

Fig 7. ARG2 and ODC1 inhibitors suppresses ARID1A-deficient TNBC bone metastasis. *(**A-B**) CCK-8 assays of different concentrations of CB-1158 (A) and DFMO (B) on 4T-1 cells for 48h. n=6, per group, one-way ANOVA for multiple comparisons. (**C-D**) CCK-8 assays of different concentrations of CB-1158 (C) and DFMO (D) on*

MDA-MB-231 cells for 48h. n=6, per group, one-way ANOVA for multiple comparisons. ***(E)*** *Proportions of mouse PMN-MDSCs induced by co-culture with Arid1a^{NC}, Arid1a^{KO} 4T-1 cells, Arid1a^{KO} 4T-1 cells plus CB-1158, and Arid1a^{KO} 4T-1 cells plus DFMO. n=5 per group, one-way ANOVA for multiple comparisons.* ***(F)*** *Proportion of human PMN-MDSCs induced by in vitro co-culture with ARID1A^{NC}, ARID1A^{KO} MDA-MB-231 cells, ARID1A^{KO} MDA-MB-231 cells plus CB-1158, and ARID1A^{KO} MDA-MB-231 cells plus DFMO. n=5 per group, one-way ANOVA for multiple comparisons.* ***(G-I)*** *Image of intratibial tumors in mice injected with Arid1a^{NC} 4T-1 cells, Arid1a^{KO} 4T-1 cells, as well as those treated with CB-1158 and DFMO (G), tumor volume (H), and tumor weight (I). n=5 per group, data are presented as mean ± SD, one-way ANOVA for multiple comparisons.* ***(J)*** *Schematic diagram illustrating the experimental design, including drug administration schedules. bid: twice daily; qd: once daily.* ***(K)*** *BLI and H&E staining images of mice in each group with intracardiac injection. Scale bar, 200μm. B bone, T tumor.* ***(L)*** *Statistical charts presenting the bone metastasis incidence in each group with intracardiac injection. n=5 per group, Fisher's exact test was used.* ***(M)*** *Bone marrow of mice was harvested from each group with intracardiac injection, and analyzed by flow cytometry to quantify PMN-MDSC proportions. n=5 per group, statistical analysis by two-way ANOVA showed no significant interaction between treatments (p=0.3035), other p values are indicated in the figures.* ***(N-Q)*** *Kaplan-Meier survival curves for 108 TNBC patients with low and high level of ARID1A, ARG2, ODC1 and OAT. The log-rank test was employed to*

calculate the difference between the two groups. All p values are indicated in the figures. Source data are provided as a Source Data file.

5. The authors have proposed that ARID1A promotes the expansion of PMN-MDSCs by upregulating the arginine metabolic pathway. To substantiate this hypothesis, it is essential to conduct experiments that specifically inhibit the arginine metabolic pathway within PMN-MDSCs.

We sincerely thank the reviewer for their thoughtful and insightful comment. It is worth noting that the central role of arginine metabolism in PMN-MDSCs has already been proved in prior published work. In the study by Steggerda et al, the authors directly isolated PMN-MDSCs from cancer patients and demonstrated that these cells potently deplete extracellular L-arginine, leading to strong suppression of T-cell proliferation. This depletion is mainly mediated by the high expression, activity and secretion of arginase enzymes in these cells. Crucially, they showed that pharmacological inhibition of arginase with CB-1158 blocked arginine consumption by PMN-MDSCs and fully restored T-cell proliferation in co-culture assays. Further assays confirmed CB-1158's potent inhibition directly targeting the primary enzyme responsible for arginine catabolism in these cells (J Immunother Cancer. 2017)[10]. In the other study by Gao et al. (J Immunother Cancer, 2019), the authors showed that the natural compound Prim-O-glucosylcimifugin (POG) effectively inhibits the immunosuppressive function of PMN-MDSCs by specifically targeting their arginine metabolic pathway[11]. These studies demonstrate that the arginine metabolic pathway

plays an essential role within PMN-MDSCs to execute their immunosuppressive roles in tumor microenviroment.

Reviewer #4 (Remarks to the Author); expert in ctDNA:

I think the science shown is pretty robust and the mechanistic work to support the science is sound. I think the conclusions ignore the fact that most bone metastases don't seem to have this mechanism (as evidenced by 45% of patients with bone mets without ARID1A mutations and a discussion on potential other mechanisms or more acknowledgement for this I would say is important. I think also the clinical significance of this could be discussed in more detail including a discussion of ARG inhibitors and current clinical trials that are underway and have been conducted for these targets.

We sincerely thank the reviewer for their feedback on our work and for these pivotal suggestions, which significantly strengthen the context and impact of our work. We fully agree that our discovery of the ARID1A-ARG2/ODC1-PMN-MDSC axis likely represents one important mechanism driving bone metastasis, but not the exclusive one. Accordingly, we have revised the Discussion to explicitly acknowledge that our model may explain a subset of bone metastasis cases. We have added a paragraph discussing the heterogeneity of bone metastases and outlining potential alternative mechanisms, thereby positioning our findings as characterizing a key molecularly defined subtype (Page 20, line 556-563). Regarding the clinical significance of our findings, the translational discussion in the revised manuscript has been expanded to detail current

and completed clinical trials targeting the arginine metabolic pathway (Page 23, line 631-638). We once again deeply appreciate the reviewer's insightful comments, which have significantly strengthened the clinical relevance of our work.

(Page 20, line 556-563)

"Our findings delineate a coherent molecular axis linking ARID1A deficiency to arginine metabolism-driven immunosuppression and bone metastasis. However, it is important to note that our clinical data indicate among patients without ARID1A mutations, 45.2% of them still developed bone metastasis, suggesting that the mechanism we describe represents a significant yet specific subset. The broader process is driven by diverse and complex synergistic mechanisms, including enhanced angiogenesis, adaptive immune resistance, among others."

(Page 23, line 631-638)

"The additive effect in suppressing PMN-MDSCs by dual inhibition confirms the vulnerability of ARID1A deficient bone metastases to arginine pathway blockade, highlighting a tangible clinical opportunity with several relevant agents already in development. Promising candidates include the arginase inhibitor CB-1158 and the ODC1 inhibitor DFMO. CB-1158 has shown acceptable safety and early signs of immune modulation in Phase I/II trials for solid tumors (NCT02903914; NCT03314935), while DFMO is being actively repurposed in oncology (NCT06059118; NCT06892678; NCT03536728)."

We sincerely thank the reviewers for their thorough evaluation, insightful critiques, and constructive suggestions. Addressing these points has allowed us to strengthen the mechanistic depth, methodological clarity, and overall rigor of our study. We believe the revised manuscript now presents a more robust and compelling account of our findings, and we are grateful for the opportunity to improve it.

Reference:

1. Wu JN, Roberts CW. (2013). ARID1A mutations in cancer: another epigenetic tumor suppressor? Cancer Discov. 3, 35-43. 10.1158/2159-8290.CD-12-0361.

2. Nagarajan S, Rao SV, Sutton J, Cheeseman D, Dunn S, Papachristou EK, Prada JG, Couturier DL, Kumar S, Kishore K, et al. (2020). ARID1A influences HDAC1/BRD4 activity, intrinsic proliferative capacity and breast cancer treatment response. Nat Genet. 52, 187-197. 10.1038/s41588-019-0541-5.

3. Alshetaiwi H, Pervolarakis N, McIntyre LL, Ma D, Nguyen Q, Rath JA, Nee K, Hernandez G, Evans K, Torosian L, et al. (2020). Defining the emergence of myeloid-derived suppressor cells in breast cancer using single-cell transcriptomics. Sci Immunol. 5. 10.1126/sciimmunol.aay6017.

4. Veglia F, Sanseviero E, Gabrilovich DI. (2021). Myeloid-derived suppressor cells in the era of increasing myeloid cell diversity. Nat Rev Immunol. 21, 485-498. 10.1038/s41577-020-00490-y.

5. Lang GT, Jiang YZ, Shi JX, Yang F, Li XG, Pei YC, Zhang CH, Ma D, Xiao Y, Hu PC, et al. (2020). Characterization of the genomic landscape and actionable mutations

in Chinese breast cancers by clinical sequencing. Nat Commun. 11, 5679. 10.1038/s41467-020-19342-3.

6. Li N, Liu Q, Han Y, Pei S, Cheng B, Xu J, Miao X, Pan Q, Wang H, Guo J, et al. (2022). ARID1A loss induces polymorphonuclear myeloid-derived suppressor cell chemotaxis and promotes prostate cancer progression. Nat Commun. 13, 7281. 10.1038/s41467-022-34871-9.

7. Zhang R, Dong M, Tu J, Li F, Deng Q, Xu J, He X, Ding J, Xia J, Sheng D, et al. (2023). PMN-MDSCs modulated by CCL20 from cancer cells promoted breast cancer cell stemness through CXCL2-CXCR2 pathway. Signal Transduct Target Ther. 8, 97. 10.1038/s41392-023-01337-3.

8. Tharp KM, Kersten K, Maller O, Timblin GA, Stashko C, Canale FP, Menjivar RE, Hayward MK, Berestjuk I, Ten Hoeve J, et al. (2024). Tumor-associated macrophages restrict CD8(+) T cell function through collagen deposition and metabolic reprogramming of the breast cancer microenvironment. Nat Cancer. 5, 1045-1062. 10.1038/s43018-024-00775-4.

9. Xu H, Zhang X, Wang X, Li B, Yu H, Quan Y, Jiang Y, You Y, Wang Y, Wen M, et al. (2024). Cellular spermine targets JAK signaling to restrain cytokine-mediated autoimmunity. Immunity. 57, 1796-1811 e1798. 10.1016/j.immuni.2024.05.025.

10. Steggerda SM, Bennett MK, Chen J, Emberley E, Huang T, Janes JR, Li W, MacKinnon AL, Makkouk A, Marguier G, et al. (2017). Inhibition of arginase by CB-1158 blocks myeloid cell-mediated immune suppression in the tumor microenvironment. J Immunother Cancer. 5, 101. 10.1186/s40425-017-0308-4.

11. Gao W, Zhang X, Yang W, Dou D, Zhang H, Tang Y, Zhong W, Meng J, Bai Y, Liu Y, et al. (2019). Prim-O-glucosylcimifugin enhances the antitumour effect of PD-1 inhibition by targeting myeloid-derived suppressor cells. J Immunother Cancer. 7, 231. 10.1186/s40425-019-0676-z.

We thank the reviewers for their thought-provoking comments and have extensively revised the manuscript accordingly. We believe the revised manuscript is substantially improved in its scientific rigor, clarity, and strength of argument.

REVIEWER COMMENTS

Reviewer #1 (Remarks to the Author); expert in epigenetics:

In this study, Pan et al discover a novel link between ARID1A loss in breast cancer cells and the upregulation of arginine metabolism, which promotes bone metastasis via expansion of PMN-MDSCs in the tumor microenvironment. ARID1A is a core subunit of the SWI/SNF chromatin remodeling complex which is found mutated in almost 10% of all cancers. Previous studies have shown that this chromatin regulator controls the expression of a diverse set of cellular metabolism genes. Here the authors use transcriptomic and metabolomic approaches to link ARID1A loss to changes in arginine metabolism gene expression and subsequent production of ornithine and spermine in triple negative breast cancer cells. Using a comprehensive set of in vivo mouse models as well as in vitro human cell lines, the authors show that loss of ARID1A in tumor cells promotes bone metastasis via the expansion of PMN-MDSCs. Furthermore, they show that exogenous treatment with ornithine or spermine is sufficient to expand PMN-MDSCs, suggesting that the production of these metabolites by Arid1a KO cells in the tumor microenvironment is a potent driver of metastasis. Finally, from an interesting therapeutic perspective, they show that treatment with small molecule inhibitors of ornithine and spermine production prevents ARID1A-deficient

triple negative breast cancer bone metastasis.

A major strength of this study is the extensive cellular and animal data presented. The authors make use of multiple cell lines (mouse and human), mouse models and transplantation approaches (intracardiac and intratibial) to dissect the link between ARID1A loss and bone metastasis. However, the molecular analysis lacks many details and is hard to follow, in particular the transcriptomics and metabolomics. I have raised several concerns for the authors and hope that my comments will help improve the clarity of the manuscript.

1. Both the transcriptomics and metabolomics data need to be presented in more detail beyond the KEGG enrichment analysis in Figure 3. The authors should provide PCA plots, volcan plots etc. to give a better sense of how many genes/metabolites are differentially regulated in the two conditions. Also, the ChIP-seq data is poorly described, H3K27ac should be mentioned in the text, as well as more details on the experimental design. Is this analysis of published data from the same experimental conditions as used for the RNA-seq?

We would like to express our sincere gratitude to the reviewer for the insightful and valuable comments, which are crucial for us to optimize the presentation of transcriptomic, metabolomic, and ChIP-seq data and enhance the overall rigor of our manuscript. We have carefully addressed all the concerns raised, with detailed revisions as follows:

1. To make the transcriptomic and metabolomic data more informative beyond KEGG enrichment analysis, we have now added PCA plots (for sample clustering

evaluation), OPLS-DA plots (for group separation validation) and volcano plots (for direct visualization of differentially regulated genes/metabolites) as supplementary Figs. 4A-D. Detailed descriptions of these plots, including the quantification of differentially expressed genes/metabolites between the two conditions, have been supplemented in the Results section (Page 8, line 201-205).

2. We have supplemented the explicit mention of H3K27ac in the corresponding text (Page 9, line 229) as suggested. Moreover, we have expanded the description of the ChIP-seq experimental design and analytical procedures in the revised manuscript (Page 33, line 902-913) to improve the clarity and reproducibility of this part of the study.
3. For the consistency of experimental conditions between datasets: the published ChIP-seq data and our RNA-seq data were derived from parallel experiments using MDA-MB-231 cells, with the same grouping strategy (ARID1A negative control vs. ARID1A knockout), ensuring the comparability of the two datasets for subsequent joint analysis.

(Page 8, line 201-205)

"Transcriptomic and metabolomic profiling of ARID1A^{KO} and negative control cells revealed distinct global profiles for each group (Supplementary Figs. 4A and B). Volcano plots identified 1445 upregulated and 2835 downregulated genes, along with 693 upregulated and 710 downregulated metabolites (Supplementary Figs. 4C and D)."

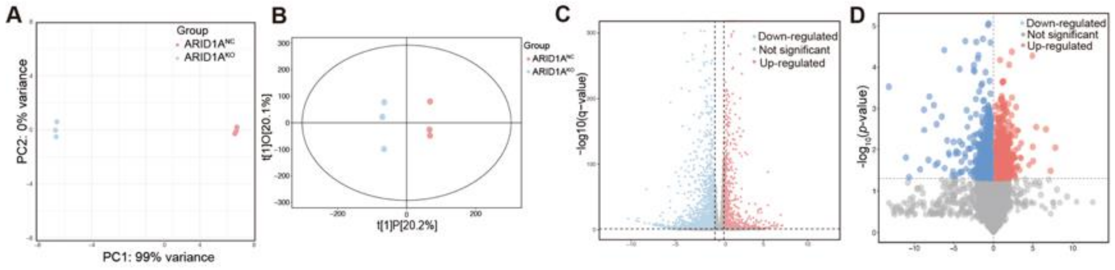

Fig S4. ARID1A-deficient TNBC upregulates the arginine metabolic pathway. *(**A**) PCA plot of RNA-Seq from ARID1A^{NC} and ARID1A^{KO} cells. (**B**) OPLS-DA plot of metabolomics from ARID1A^{NC} and ARID1A^{KO} cells. (**C**) Volcano plot of differentially expressed genes from RNA-Seq in ARID1A^{NC} and ARID1A^{KO} cells. (**D**) Volcano plot of differential metabolites from metabolomics in ARID1A^{NC} and ARID1A^{KO} cells.*

(Page 9, line 229)

"Notably, the H3K27ac enrichment at the specific regulatory region of ARG2 was increased by 10-fold in ARID1A^{KO} MDA-MB-231 cells compared with control cells, indicating enhanced transcriptional potential of ARG2 in the absence of ARID1A (Fig. 3E)."

(Page 33, line 902-913)

"ATAC-seq and ChIP-seq library construction and analysis

The ATAC-seq and ChIP-seq data for MDA-MB-231 cells were obtained from the Gene Expression Omnibus (GSE234179), deposited by our research team (Chen et al., Cancer Commun, 2023). ChIP-seq libraries were sequenced on an Illumina NovaSeq 6000 platform at Cloud-seq (Shanghai, China). ATAC-seq libraries were sequenced on an Illumina X-Ten system. Both generated 150-bp paired-end reads. ATAC-seq and

H3K27ac ChIP-seq signals were normalized to RPKM, and comparative log2(KO/NC) tracks were generated. ATAC-seq peaks were called from aligned reads using MACS3, and a union peak set was intersected with transcriptional start sites (TSSs, UCSC refGene, hg38) to define promoter-proximal regions. Visualization matrices were generated using deepTools computeMatrix in reference-point mode with a window of ±2 kb and a bin size of 100 bp."

2. To what extent is ARID1A loss impacting SWI/SNF activity in these tumor cells? In addition to the RNAseq data, ATAC-seq would allow for a more direct assessment of SWI/SNF dependent changes in chromatin accessibility. In addition to the ARID1A ko experiments, using SWI/SNF inhibitors would also determine if is this is a general function of SWI/SNF or specifically linked to ARID1A.

We sincerely thank the reviewer for the constructive and insightful suggestions. To directly evaluate SWI/SNF-dependent chromatin accessibility changes as suggested, we performed ATAC-seq analysis on ARID1A^{KO} and negative control MDA-MB-231 cells (GSE234179). Results showed that ARID1A deficiency reduced global chromatin accessibility, indicating impaired SWI/SNF-mediated chromatin remodeling—consistent with ARID1A being a core SWI/SNF subunit. This finding is presented in Supplementary Fig. 4E, with descriptions incorporated into the revised manuscript (Page 9, line 221-228).

To distinguish whether the observed phenotypes reflect general SWI/SNF function or ARID1A-specific effects, we utilized a SWI/SNF inhibitor (BRM014, a catalytic

inhibitor targeting SWI/SNF ATPase) and an intracardiac injection bone metastasis model in Balb/c mice, with evaluations in both *Arid1a*^{NC} and *Arid1a*^{KO} groups. BRM014 had no significant effect on bone metastasis in *Arid1a*^{NC} mice but suppressed bone metastasis in *Arid1a*^{KO} mice. Similarly, BRM014 failed to alter the concentration of PMN-MDSCs in the bone marrow of *Arid1a*^{NC} mice but significantly reduced their elevated levels in *Arid1a*^{KO} mice. These data confirm that ARID1A deficiency-induced bone metastasis depends on the residual SWI/SNF complex; notably, standalone SWI/SNF inhibition cannot mimic the phenotypic alterations caused by ARID1A deficiency, which implicates additional ARID1A-specific functional roles in mediating tumor progression. These results are shown in Supplementary Figs. 12A–C, with detailed analyses added to the revised manuscript (Page 17, line 460-476).

(Page 9, line 221-228)

"To explore the potential regulatory mechanism underlying low ARID1A expression-induced ARG2 transcription activation, we performed Assay for transposase accessible chromatin (ATAC)-seq and Chromatin Immunoprecipitation-Sequencing (ChIP-seq) analysis in ARID1A^{KO} *and negative control MDA-MB-231 cells. Our analyses revealed that ARID1A deficiency led to a global reduction in chromatin accessibility, indicating impaired SWI/SNF-dependent chromatin remodeling—consistent with ARID1A's established role as a core subunit of the SWI/SNF complex (Supplementary Fig. 4E)."*

E

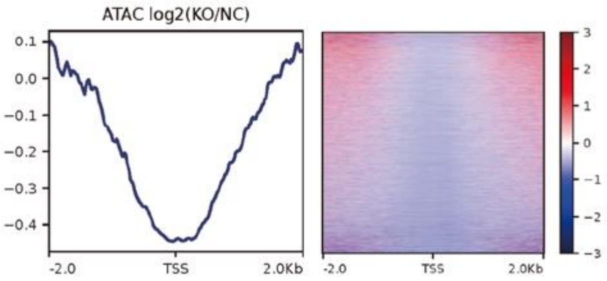

Supplementary Fig.4 ARID1A-deficient TNBC upregulates the arginine metabolic pathway. *(E) ATAC signal at global chromatin accessibility in ARID1A^{KO} cells compared to ARID1A^{NC} cells.*

(Page 17, line 460-476)

"To evaluate whether ARID1A deficiency promotes bone metastasis through residual SWI/SNF activity, we employed a bone metastasis model using the BRM014, a catalytic inhibitor of SWI/SNF ATPase activity. We found that BRM014 treatment did not significantly alter bone metastasis in Arid1a^{NC} control mice. In contrast, BRM014 suppressed bone metastasis in Arid1a^{KO} mice (Supplementary Figs. 12A and B). Similarly, BRM014 failed to alter the concentration of PMN-MDSCs in the bone marrow of Arid1a^{NC} mice but significantly reduced their elevated levels in Arid1a^{KO} mice (Supplementary Fig. 12C). Collectively, these data indicate that bone metastasis driven by ARID1A deficiency is dependent on the residual SWI/SNF complex; notably, standalone SWI/SNF inhibition cannot mimic the phenotypic alterations caused by ARID1A deficiency, which implicates additional ARID1A-specific functional roles in mediating tumor progression. However, targeting the SWI/SNF complex poses a significant challenge due to concerns of on-target toxicity, stemming from its ubiquitous

expression and indispensable functions in normal cells, we pivoted to a more promising therapeutic strategy centered on targeting the arginine signaling pathway."

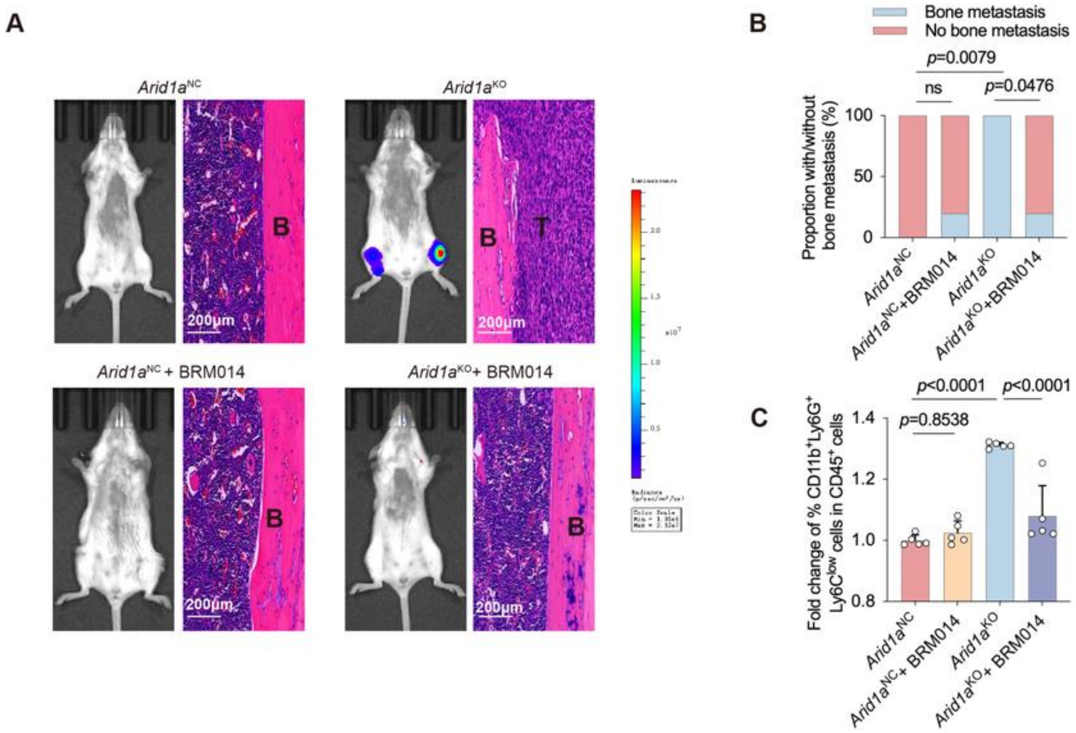

Supplementary Fig. 12. ARID1A deficiency relies on residual SWI/SNF complex to promote bone metastasis. *(**A**) BLI and H&E staining images of mice in Arid1a^{NC}, Arid1a^{NC} plus BRM014, Arid1a^{KO}, Arid1a^{KO} plus BRM014 group with intracardiac injection. Scale bar, 200μm. B bone, T tumor. (**B**) Statistical charts presenting the bone metastasis incidence in each group with intracardiac injection. n=5 per group, fisher's exact test was used. (**C**) Bone marrow of mice was harvested from each group and analyzed by flow cytometry to quantify PMN-MDSC proportions. n=5 per group, one-way ANOVA for multiple comparisons. p values are indicated in the figures. Source data are provided as a Source Data file.*

3. ARID1A functions within the multi-subunit SWI/SNF complex yet overexpression

of a single subunit reduces metastasis whereas knockout increases metastasis compared to control cells. Could the authors comment on why the phenotype is so sensitive to ARID1A dosage within the SWI/SNF complex, is ARID1B expressed in these cancer models?

We thank the reviewer for raising this critical point regarding the dosage sensitivity of ARID1A in our bone metastasis models and the potential role of ARID1B.

The bone metastasis phenotype is acutely sensitive to ARID1A dosage can be explained through its indispensable structural and functional roles within the SWI/SNF complex:

1. **Structural keystone of SWI/SNF complex:** ARID1A belongs to the SWI/SNF complex and is a core member of the ATP-dependent polymorphic BRG/BRM-associated factor (BAF) chromatin remodeling unit. Its presence or absence dictates the complex's protein composition, nuclear localization, and partner interactions (Jennifer N. Wu et al., Cancer Discov. 2013 Jan)[1]. A change in its dosage thus alters the fundamental identity and capability of the SWI/SNF complex in the cell.
2. **Functional roles in SWI/SNF complex:** The ARID1A protein comprises the DNA-binding subunit of SWI/SNF chromatin remodeling complexes and ARID1A regulates chromatin accessibility. Chromatin remodeling dynamically modifies chromatin architecture to allow access of condensed genomic DNA to the transcription regulatory machinery, thereby controlling gene expression (Sankari Nagarajan et al., Nat Genet. 2020 Feb)[2]. In our study, the loss of ARID1A altered chromatin accessibility which in turn activated ARG2 expression and promoted the

upregulation of the arginine metabolic pathway. This ultimately led to an increase in the concentration of PMN-MDSCs within the bone marrow tumor microenvironment, fostering an immunosuppressive milieu that facilitates enhanced tumor cell retention. This explains why the phenotype is exquisitely sensitive to ARID1A levels, as it controls gene expression by constituting an indispensable and uniquely encoded layer of regulatory control within the epigenetic complex.

Furthermore, we performed IHC to analyze *Arid1b* expression in tumors from *Arid1a*^{NC} and *Arid1a*^{KO} mice as suggested. The results showed that *Arid1b* was present in both *Arid1a*^{NC} and *Arid1a*^{KO} tumor tissues, but with no significant difference in its expression levels between the two groups (As shown in panels A and B of the figure below).

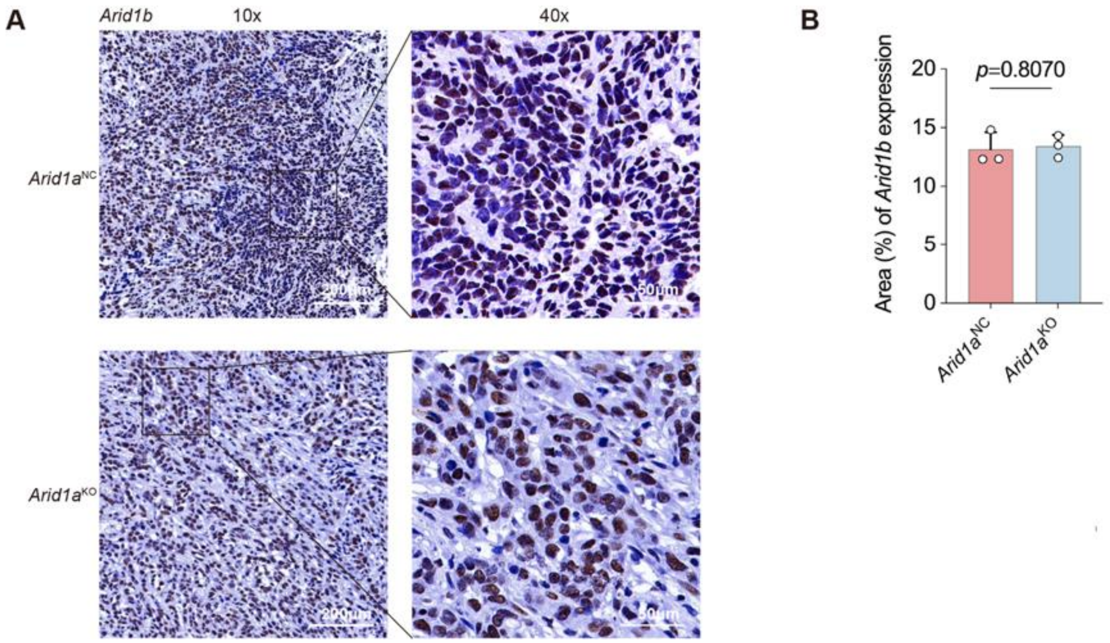

(A) *IHC images of Arid1b expression in tumors from Arid1a*^{NC} *mice compared to Arid1a*^{KO} *mice. Scale bar, 10x, 200μm.* ***(B)*** *Quantification of the protein expression*

levels of Arid1b in two groups. n=3 per group, unpaired two-tailed Student's t test was used.

4. Is it known what makes PMN-MDSCs so sensitive to ornithine and spermine compared to other cells in the niche? Do they express specific transporters at their cell surface? This could be addressed using the scRNAseq datasets.

We sincerely thank the reviewer for this insightful question. We propose that the specific sensitivity of PMN-MDSCs to ornithine and spermine stems from their inherent metabolic specialization in the arginine-polyamine axis. This is supported by three lines of evidence:

1. PMN-MDSCs are characterized by their ability to suppress immune responses primarily through arginine metabolism through the expression of immunosuppressive enzymes arginase 1 (ARG1) and, in some contexts, arginase 2 (ARG2) (Hamad Alshetaiwi et al., Sci Immunol. 2020 Feb; Filippo Veglia et al., Nat Rev Immunol. 2021 Aug)[3, 4]. This established role highlights that PMN-MDSCs are inherently specialized cells whose identity and function are closely tied to the core pathway involving arginine metabolism.
2. Our in-depth analysis of the ScRNA-seq datasets revealed that polyamine metabolic processes are specifically enriched in PMN-MDSCs compared to other cells (Supplementary Fig. 10A). Polyamines (putrescine, spermine, spermidine) are direct downstream metabolites synthesized from ornithine, which itself is derived from arginine. This directly links PMN-MDSCs to the downstream branch of

arginine metabolism where ornithine and spermine reside. This result has been added in the revised manuscript (Page 15, line 400-404).

3. In the ARID1A^{KO} group, where this sensitivity is pronounced, PMN-MDSCs exhibit upregulated expression of key enzymes (ARG2 and SAT1) which are pivotal for arginine and polyamine metabolism, rather than changes in metabolite transporters.

Therefore, we propose that the heightened sensitivity of PMN-MDSCs to ornithine and spermine stems from the innate, enzyme-driven capacity to rapidly process these specific metabolites, granting them a competitive advantage in metabolizing niche resources.

(Page 15, line 400-404)

"We found that PMN-MDSCs exhibited a significant enrichment in amine and polyamine metabolism pathways compared to other cell types, indicating a metabolic reprogramming that renders them particularly dependent on this pathway to fuel their rapid expansion and functional activity (Supplementary Fig. 10A)."

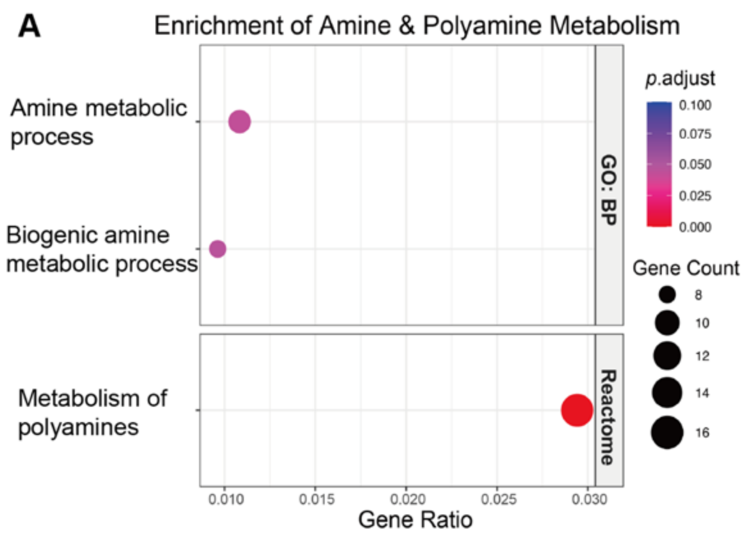

Supplementary Fig. 10 ARG2 and SAT1 are highly expressed in PMN-MDSCs from ARID1A^{KO} group. *(A) Enrichment of amine and polyamine metabolism in PMN-MDSCs.*

5. Have the authors tried to combine ornithine and spermine treatments? Same question for the inhibitors, what is the consequence of dual inhibition?

We thank the reviewer for raising the insightful point regarding the investigation of combined treatments. Accordingly, we examined both metabolite supplementation and inhibitor blockade in combination. To assess potential interactions, we employed two-way ANOVA analysis. For the metabolites, a significant interaction was observed between ornithine and spermine (p<0.0001). However, their combined administration induced only a modest increase in bone marrow PMN-MDSCs compared to spermine alone, while remaining comparable to ornithine alone. This result suggests that the pathway for PMN-MDSC induction could be saturated by metabolic supply. Furthermore, the combination of CB-1158 and DFMO demonstrated greater efficacy in reducing bone metastasis incidence. Specifically regarding PMN-MDSC reduction,

while no significant interaction was found (*p*=0.3035), the combination was nonetheless more effective than either agent alone, suggesting an additive rather than a synergistic effect. Taken together, these findings suggest that the pathway driving PMN-MDSC induction can be saturated by metabolic supply; and dual inhibitors achieves additive efficacy in lowering both bone metastasis incidence and PMN-MDSC proportion. These results have been added to the revised manuscript (Page 16, line 435-440 and Page 19, line 508-513) .

(Page 16, line 435-440).

"To further explore the combined effect of ornithine and spermine on promoting PMN-MDSCs, we utilized an intracardiac injection model (Fig. 6J). Although a significant interaction was observed between ornithine and spermine ($p<0.0001$). However, we found that their combined administration induced only a modest increase in bone marrow PMN-MDSCs compared to spermine alone, while remaining comparable to ornithine alone. (Fig. 6K)."

REDACTED

Fig 6. Ornithine and spermine secreted by cancer cells expand PMN-MDSCs. *(J) Mouse models were established by intracardiac injection of wild-type 4T-1 cells, and then subjected to exogenous supplementation with ornithine, spermine, or their*

*combination. (**K**) PMN-MDSC proportions in bone marrow of each group. n=5 per group, two-way ANOVA for multiple comparisons. The interaction between the two factors was significant ($p<0.0001$), other p values are indicated in the figures. Source data are provided as a Source Data file.*

(Page 19, line 508-513).

"We next assessed the potential interaction between CB-1158 and DFMO (Fig. 7J). Their combination demonstrated greater efficacy in reducing bone metastasis incidence (Figs. 7K and L). For the reduction of PMN-MDSCs, although no significant interaction was detected ($p=0.3035$), the combination was more effective than either agent alone, suggesting an additive rather than a synergistic effect (Fig. 7M)."

REDACTED

Fig 7. ARG2 and ODC1 inhibitors suppress ARID1A-deficient TNBC bone metastasis. *(**J**) Schematic diagram illustrating the experimental design, including drug*

*administration schedules. bid: twice daily; qd: once daily. (**K**) BLI and H&E staining images of mice in each group with intracardiac injection. Scale bar, 200μm. B bone, T tumor. (**L**) Statistical charts presenting the bone metastasis incidence in each group with intracardiac injection. n=5 per group, Fisher's exact test was used. (**M**) Bone marrow of mice was harvested from each group with intracardiac injection, and analyzed by flow cytometry to quantify PMN-MDSC proportions. n=5 per group, statistical analysis by two-way ANOVA showed no significant interaction between treatments (p=0.3035), other p values are indicated in the figures.*

Minor:

1. In the ctDNA analysis, are mutations in any other SWI/SNF subunits detected? As mentioned by the authors, almost 10% of all cancers have mutations in ARID1A, so detecting 53/663 in this cohort does not appear as a strong enrichment.

Is the mechanism described in this study specific to the ARID1A subunit or a more general SWI/SNF function?

Thank you for this insightful question regarding mutations in other SWI/SNF subunits within our ctDNA cohort. In our ctDNA cohort, mutations in other SWI/SNF subunits were detected at the following frequencies: SMARCA4 in 20 patients (3.02%), SMARCB1 in 3 (0.45%), ARID1B in 14 (2.11%), ARID2 in 14 (2.11%), and BRD7 in 2 patients (0.30%). Additionally, none of these alterations in other SWI/SNF subunits showed a statistically significant correlation with bone metastasis. These results have

been presented as supplementary Table 2, with corresponding descriptions added to the Results section (Page 6, line 133-138).

As demonstrated by our results with the SWI/SNF inhibitor, BRM014 treatment did not significantly alter bone metastasis incidence and PMN-MDSC proportion in *Arid1a*^{NC} mice but suppressed them in *Arid1a*^{KO} mice. This indicates that the pro-metastatic state induced by ARID1A deficiency remains dependent on residual SWI/SNF complex activity, the inhibition of SWI/SNF alone fails to replicate the ARID1A deficiency, implicating additional ARID1A-specific roles in tumor progression. This specificity is further supported by clinical ctDNA data, which reveal that bone metastasis is uniquely associated with ARID1A loss rather than mutations in other SWI/SNF subunits.

In addition, thank you for raising the important question regarding the proportion of ARID1A mutations. The figure stating that ARID1A mutations are "present in approximately 10% of tumors" actually represents a pan-cancer average, the frequency in breast cancer is specifically about 7% (Guan-Tian Lang et al., Nat Commun. 2020 Nov)[5]. We have added it to the revised manuscript (page 4, line 67).

(Page 6, line 133-138)

"We also detected mutations in additional SWI/SNF subunits in our ctDNA cohort, with the following frequencies: SMARCA4 in 20 patients (3.02%), SMARCB1 in 3 (0.45%), ARID1B in 14 (2.11%), ARID2 in 14 (2.11%), and BRD7 in 2 patients (0.30%). Notably,

none of these mutations showed a statistically significant correlation with bone metastasis (Supplementary Table 2).”

Supplementary Table 2. Mutated SWI/SNF subunits in the ctDNA cohort.

Mutated SWI/SNF subunits (Total=663)	***Bone metastasis***	***No bone metastasis***	***p value[a]***
ARID1A (n=53)	*34*	*19*	*0.0081*
ARID1B (n=14)	*6*	*8*	*0.7675*
ARID2 (n=14)	*7*	*7*	*0.8058*
SMARCA4 (n=20)	*11*	*9*	*0.4531*
SMARCB1 (n=3)	*3*	*0*	*0.0640*
BRD7 (n=2)	*2*	*0*	*0.1307*

[a] p values were determined using the Pearson's chi-square test.

(Page 4, line 67)

“ARID1A mutations, present in approximately 10% of tumors (about 7% in breast cancer)[7], predominantly consist of inactivating mutations that drive loss of ARID1A protein, with its deficiency conferring poor clinical outcomes across multiple cancer types[8,9].”

2. For the engineering of the Arid1a KO and OE cell lines, more details are required.

While the cellular phenotypes are striking, could the authors comment on the incomplete nature of the knock-out as well as how they overexpressed such a large protein using viruses (dCas9 approach?).

We thank the reviewer for raising the point regarding the the engineering of the *Arid1a*^{KO} and *Arid1a*^{OE} cell lines. As suggested, more detailed descriptions of the *Arid1a*^{KO} and *Arid1a*^{OE} cell line generation have been added to the revised manuscript (Page 26, line 714-737). Regarding the incomplete *Arid1a* knockout observed by western blot, we appreciate the reviewer's attention to this technical detail. This is a common outcome in CRISPR/Cas9 editing of large genes such as ARID1A (240 kDa). Following DNA cleavage, repair via non-homologous end joining (NHEJ) can generate in-frame indels, potentially allowing the expression of a truncated protein that may still be detected by antibodies targeting upstream epitopes. Additionally, even after selection, the polyclonal population may contain a minor fraction of incompletely edited cells, which could contribute to a faint residual signal in western blot. Furthermore, the observed phenotypic differences between *Arid1a*^{NC} and *Arid1a*^{KO} cells support that the *Arid1a*^{KO} cell line is a valid loss-of-function model for this study. To overexpress ARID1A, we utilized a CRISPR activation (CRISPRa) approach via the synergistic activation mediator (SAM) system. This system is based on a deactivated Cas9 (dCas9) platform.

(Page 26, line 714-737)

"Stable transfection using lentiviral infection

To generate ARID1A^{KO} cells, a lentiviral CRISPR/Cas9 system was employed. The Lenti-CAS9-Puro vector and the single-guide RNA (sgRNA) expression vector GV371-EGFP (Shanghai Genechem, Shanghai, China), carrying Cas9 and an ARID1A-targeting guide RNA respectively, were co-transfected with packaging plasmids into HEK293T cells using Lipofectamine 3000 (L3000-150, Invitrogen) to generate lentiviral particles. For the construction of ARID1A^{OE} cells, aCRISPR/Cas9 activation (CRISPRa) approach was employed using thesynergistic activation mediator (SAM) system (Genechem). This system is based on a deactivated Cas9 (dCas9) platform. The SAM vector was programmed with a specific sgRNA targeting the promoter region of the ARID1A and the empty vector was used as negative control. The vector was similarly co-transfected with packaging plasmids into HEK293T cells for lentivirus production. For both KO and OE constructions, the virus-containing supernatant was collected 72h after incubation, filtered and used to transduce cells in the presence of polybrene (5 μg/mL). To establish stable polyclonal populations, successfully transduced cells were selected with puromycin, with the antibiotic concentration optimized for the specific cell line. The primers used for plasmid construction were listed in Supplementary Table 7. The ARID1A knockout and overexpression efficiency was confirmed via western blotting."

3. The ChIP-seq dataset GSE98765 does not link to the study by Chen et al.

Thank you for pointing this out. We have carefully checked and corrected it to the accurate dataset citation (GSE234179).

Reviewer #2 (Remarks to the Author); expert in breast cancer, bone metastasis and tumour microenvironment:

This manuscript investigates an important topic that is gaining considerable interest of metabolic crosstalk between cancer cells and immune cells. This is a complex and nuanced topic, and the current study attempts to understand the important question of what tumour mutations change tumour cell metabolism to promote bone marrow metastasis tropism. Particularly important to note is that there is currently no effective/ targeted treatment option for patients with breast cancer bone marrow metastasis. This underscores the importance of the work. Whilst this is an interesting and important topic, the manuscript in its current form lacks major controls and overstates the data making it very hard to interpret.

Unfortunately, there are no good models of breast cancer -> bone metastasis. This project relies on cell line injection models and the limitations of this should be discussed.

Thank you for raising this important point regarding the limitations of our experimental models. We have incorporated the discussion on the limitations of the models into the Discussion section (Page 21, line 566-573).

(Page 21, line 566-573)

"We established models of bone metastasis in mice by performing intracardiac and intratibial injections with luciferase-expressing TNBC cells, and monitored tumor burden in vivo using BLI. It is important to acknowledge that these experimental models bypass the critical early stages of the metastatic cascade, including primary tumor development and spontaneous dissemination, thus limiting their ability to fully recapitulate the natural progression of breast cancer to bone. Future studies employing more physiologically relevant, spontaneous metastasis models will be necessary to better mimic the clinical course of the disease."

In the introduction, the authors define PMN-MDSCs as CD11b+Ly6G+Ly6C-low. This is precisely the same definition as a neutrophil and highlights the problem with MDSC nomenclature. A fundamental problem with using MDSC nomenclature is that it treats PMN-MDSCs as distinct from neutrophils and M-MDSCs as distinct from monocytes, when in fact recent work has demonstrated they represent functional states of neutrophils and monocytes. Labeling them as a separate category obscures their true lineage and hampers our ability to understand their biology in the broader context of myeloid cell plasticity.

Thank you for raising this very important and insightful point regarding the definition of PMN-MDSCs. We agree that the nomenclature and the relationship between PMN-MDSCs and classical neutrophils represent a key and actively debated issue in the field of tumor immunology. We agree with the growing consensus, as noted by the reviewer, that PMN-MDSCs are better understood as neutrophils that have acquired a potent

immunosuppressive phenotype under the sustained pressure of the tumor microenvironment, sometimes termed "pathologically activated" or "granulocytic" myeloid-derived suppressor cells.

In our study, we employed the operational definition of murine PMN-MDSCs as $CD11b^{+}Ly6G^{+}Ly6C^{low}$ cells isolated from tumor-bearing hosts. This definition remains the established standard in the field to identify and interrogate the granulocytic population responsible for immunosuppression in cancer, allowing for comparison with a vast body of prior work (Ni Li et al., Nat Commun. 2022 Nov; Filippo Veglia et al., Nat Rev Immunol. 2021 Aug; Rui Zhang et al., Signal Transduct Target Ther. 2023 Mar)[4, 6, 7]. Our study was primarily designed to investigate the functional contribution of this immunosuppressive granulocytic subset to bone metastasis in ARID1A-deficient breast cancer. Using the term "PMN-MDSC" most precisely conveys to readers that we are focusing on this functionally defined immunosuppressive state, rather than making a claim about its ontological distinction from neutrophils.

We appreciate the importance of not overlooking this conceptual nuance. Therefore, in the revised Discussion section, we have added a dedicated paragraph to clarify this scope (Page 22, line 598-605). We acknowledge the evolving understanding that these cells likely represent a functional state within the spectrum of neutrophil plasticity and cite key literature supporting this view, placing our findings within this broader conceptual framework.

(Page 22, line 598-605)

"PMN-MDSCs, also known as pathologically activated neutrophils, are a critical component of the TME and play crucial roles in tumour progression and therapy resistance[54-56]. As defined by the phenotypic markers $CD11b^+Ly6G^+Ly6C^{low}$, PMN-MDSCs are better understood as a functional immunosuppressive state acquired by neutrophils, under specific tumor microenvironmental pressures[53]. We revealed a critical immunosuppressive functional state of neutrophils within the ARID1A deficient bone metastatic niche which underscores the dynamic nature of myeloid cells in cancer."

Injection of luciferase labelled cells: allows to follow tumour cells but Luciferase is not naturally expressed in mammalian tissues; therefore, introduction of luciferase-expressing cells provides a neoantigen that is recognised as foreign by the host immune system. This can elicit inflammatory responses, contributing to the overall immunogenicity of the injected cell line, and skewing readouts of inflammatory cells. The methods are not very detailed and a lot of experimental details that are required to understand the data are missing. For example, tumour cell growth after manipulation, size of tumours at euthanasia (is it the same between experimental groups?), Figure 5, is is not clear which model the experiments are performed in.

We appreciate the reviewer for these insightful comments, which have helped strengthen the manuscript. In response, we have made the following key revisions:

1. We thank the reviewer for raising this important methodological point regarding the potential immunogenicity of luciferase. We fully acknowledge that the

expression of this non-mammalian protein could elicit local immune responses. Nonetheless, luciferase-based bioluminescence imaging remains an established method for the longitudinal and quantitative tracking of metastatic burden in mouse models. To address this consideration, we have incorporated a statement in the Discussion section (Page 21, line 573-578) acknowledging this methodological constraint while underscoring the utility of the approach for in vivo tracking.

2. We thank the reviewer for highlighting the need for greater methodological detail. In response, we have thoroughly revised the Methods section to provide a comprehensive description of the experimental procedures and details.
3. The legend for Figure 5 has been revised to explicitly specify the experimental model (intracardiac vs. intratibial injection) used for each panel, eliminating any ambiguity.

(Page 21, line 573-578)

"Additionally, while the luciferase reporter is essential for in vivo imaging, its expression may introduce immunogenic effects that could influence tumor behavior and the host microenvironment[48]*. Despite this potential constraint, luciferase-based imaging remains an effective method for longitudinally tracking metastatic progression in preclinical bone metastasis research*[29,31,49]*."*

*“**Fig 5. ARID1A deficiency drives the expansion of PMN-MDSCs.** (**A**) Proportions of human PMN-MDSCs after in vitro co-culture with ARID1A^{NC} and ARID1A^{KO} MDA-MB-231 cells. n=5 per group, unpaired two-tailed Student’s t test. (**B**) Proportions of PMN-MDSCs in the bone marrow of Arid1a$^{flox/flox}$;Mmtv-Cre mice compared to Mmtv-Cre control mice. n=6 per group, unpaired two-tailed Student’s t test. (**C**) Bone marrow was harvested from **Balb/c mice following intracardiac injection of Arid1a^{NC} and Arid1a^{KO} 4T-1 cells**, and analyzed by flow cytometry to quantify PMN-MDSC proportions. n=7 per group, unpaired two-tailed Student’s t test. (**D**) PMN-MDSC proportions in bone metastatic tumors from **Balb/c mice following intratibial injection of Arid1a^{NC} and Arid1a^{KO} 4T-1 cells.** n=5 per group, unpaired two-tailed Student’s t test. (**E-F**) The percentage of CD11b^{+}Ly6G^{+} cells in bone metastatic tumors from **Balb/c mice following intratibial injection of Arid1a^{NC} and Arid1a^{KO} 4T-1 cells** was quantified by IF staining, with quantitative analysis between experimental groups. n=3 per group, unpaired two-tailed Student’s t test. Scale bar, 20μm. (**G-H**) The proportion of PMN-MDSCs in tumor tissues from TNBC patients with high versus low ARID1A expression was assessed by TSA staining on tissue microarrays, followed by quantitative comparison. n=40 per group, unpaired two-tailed Student’s t test. Scale bar, 20μm. All p values are indicated in the figures. Source data are provided as a Source Data file.”*

Can we assume the MDA-MB-231 would behave the same way as 4T1 in terms of bone marrow metastasis?

We thank the reviewer for this comment. In our experiments, intracardiac injection of luciferase-labeled ARID1A^{NC} and ARID1A^{KO} MDA-MB-231 cells into female NSG mice that has been humanized with human CD34+ hematopoietic stem cells revealed that these cells exhibited a comparable pattern of bone metastasis to 4T-1 cells. These results are now included as Figure 2D and G, with the corresponding descriptions in the revised manuscript (Page 7, line 169-172).

(Page 7, line 169-172)

"Similarly, in NOD-scid IL2Rγnull (NSG) mice humanized with human CD34+ hematopoietic stem cells and injected with MDA-MB-231 cells, the incidence of bone metastasis was significantly greater in the KO group compared with the NC controls (Fig. 2G)."

REDACTED

Fig 2. ARID1A deficiency promotes bone metastasis of breast cancer. (D) *Representative BLI, H&E staining images of two groups of mice following intracardiac injection with equal numbers of ARID1A*^{NC} *and ARID1A*^{KO} *MDA-MB-231 cells. Scale bar, 200μm. n=6 per group.* ***(G)*** *Following intracardiac injection of MDA-MB-231 cells, bone metastasis was detected in 5 mice (5/6) in the ARID1A*^{KO} *group, whereas no metastasis (0/6) was observed in the ARID1A*^{NC} *group. n=6 per group, Fisher's exact test was used.*

Manipulations of Arid1 – do tumours and cell lines grow at the same rate upon Arid1-

ko? Throughout the manuscript, we cannot understand if the results are a function of faster tumour growth / bigger tumours.

We thank the reviewer for raising this critical question. To clarify, the observed pro-metastatic phenotype is not attributable to a general increase in tumor growth rate or size upon ARID1A deficiency. Our integrated experimental data support a model in which ARID1A deficiency reshapes the immune microenvironment to drive metastasis. The key evidence is as follows:

1. **No intrinsic proliferation difference in vitro:** To address the possibility of cell-autonomous growth differences, we performed comprehensive in vitro assays. Colony formation and CCK-8 viability assays using multiple cell lines (4T-1, MDA-MB-231, BT-549) demonstrated that ARID1A knockout does not confer a significant intrinsic proliferative advantage under standard culture conditions. These results are now included as supplementary Figs. 11A and B, with the corresponding descriptions in the revised manuscript (Page 17, line 449-452).
2. **The crucial role of PMN-MDSCs:** To directly test the core role of PMN-MDSCs in this process, we performed in vivo depletion using an anti-Ly6G antibody. The depletion of PMN-MDSCs completely abolished the bone metastatic advantage of the *Arid1a*^{KO} group compared to the *Arid1a*^{NC} group. These findings demonstrate PMN-MDSC as a key mediator of bone metastasis in ARID1A-deficient TNBC (Supplementary Figs. 11B-D), with the corresponding descriptions in the revised manuscript (Page 17, line 453-458).

In summary, our data collectively demonstrate that ARID1A deficiency drive bone metastasis by reshaping the tumor immune microenvironment, particularly by enhancing the recruitment and utilization of PMN-MDSCs, thereby conferring a growth and metastatic advantage supported by these immune cells.

(Page 17, line 449-452 and Page 17, line 453-458)

"To further validate the critical role of PMN-MDSCs in ARID1A deficiency-mediated bone metastasis, we examined whether the proliferative capacity differed between ARID1A^{NC} and ARID1A^{KO} cells. We found that ARID1A^{NC} and ARID1A^{KO} cells exhibited comparable proliferative capacities in vitro (Supplementary Figs. 11A and B). Given that the intrinsic growth of tumor cells was unaffected, we administered anti-Ly6G antibody to delete PMN-MDSCs in mice[41]. In the intracardiac injection model established with Arid1a^{NC} or Arid1a^{KO} cells, anti-Ly6G antibody treatment significantly reduced bone metastatic incidence in Arid1a^{KO} group (Supplementary Figs. 11C-E). These findings demonstrate PMN-MDSC as a key mediator of bone metastasis in ARID1A-deficient TNBC."

REDACTED

Supplementary Fig. 11 PMN-MDSCs play a critical role in ARID1A deficiency-mediated bone metastasis. *(**A**) CCK-8 assays of TNBC cells (4T-1, MDA-MB-231, BT-549) between ARID1A^{NC} and ARID1A^{KO} group. n=7, two-way ANOVA was used. (**B**) Clone formation assays of TNBC cells (4T-1, MDA-MB-231, BT-549) between ARID1A^{NC} and ARID1A^{KO} group. n=3, unpaired two-tailed Student's t test. (**C**) Treatment schedule schematic. Following intracardiac injection of Arid1a^{NC} and Arid1a^{KO} 4T-1 cells, mice were allocated to receive either anti-Ly6G antibody or isotype control IgG. (**D**) BLI and H&E staining images of mice in Arid1a^{NC} plus IgG, Arid1a^{NC} plus αLy6G, Arid1a^{KO} plus IgG，Arid1a^{NC} plus αLy6G group. Scale bar,*

*200μm. B bone, T tumor. n=5 per group. (**E**) Statistical charts presenting the bone metastasis incidence in each group. n=5, data was analyzed by Fisher's exact test. p values are indicated in the figures.*

Figure 4E – in the feature plots, do these cells come from mice injected with Arid1NC or Arid1ko? What is the difference between neutrophil and a PMN-MDSC. As mentioned above, recent publications have indicated that these are not separate entities, but a functional continuum impacted by the systemic environment driven by the tumour.

Thank you for this critical and insightful question. All cells presented in Figure 4E were isolated from the bone marrow of mice injected with either *Arid1a*^{KO} or *Arid1a*^{NC} tumor cells. The plots were generated from the aggregated single-cell data of this mixed population, aiming to visualize different cell types expressing characteristic markers. We fully agree with the reviewer that current evidence supports viewing PMN-MDSCs not as a distinct lineage, but as a functional immunosuppressive state acquired by neutrophils under tumor-derived pressures. In Figure 4E, cells expressing high levels of the canonical granulocyte marker Ly6G were annotated as Neutrophils. Within that broader population, the subset that expressed high levels of IL-1β was annotated as PMN-MDSCs. This annotation, consistent with studies like Alshetaiwi et al., (Alshetaiwi et al., Sci. Immunol. 2020)[3] was used to identify and focus on that specific neutrophil subpopulation exhibiting a pronounced immunosuppressive phenotype within the bone marrow microenvironment shaped by ARID1A-deficient tumors. Our

emphasis was on revealing this acquired functional state, its role in promoting bone metastasis, and its regulatory mechanisms, rather than on lineage distinctions. Therefore, in the revised Discussion section, we have added a dedicated paragraph to clarify this scope (Page 22, line 598-605). We thank the reviewer again for this constructive comment, which has significantly strengthened the rigor of our study.

(Page 22, line 598-605)

"PMN-MDSCs, also known as pathologically activated neutrophils, are a critical component of the TME and play crucial roles in tumour progression and therapy resistance[54-56]*. As defined by the phenotypic markers* $CD11b^{+}Ly6G^{+}Ly6C^{low}$*, PMN-MDSCs are better understood as a functional immunosuppressive state acquired by neutrophils, under specific tumor microenvironmental pressures*[53]*. We revealed a critical immunosuppressive functional state of neutrophils within the ARID1A deficient bone metastatic niche which underscores the dynamic nature of myeloid cells in cancer."*

Figure 4F – using pseudotime trajectories in this manner is increasingly common but it is a fundamentally flawed methodology. In the case of neutrophils and PMN-MDSCs, using pseudotime is problematic because these populations are highly plastic and can interconvert depending on environmental cues. Without a clear, unidirectional differentiation pathway, the algorithm may infer a trajectory that assumes PMN-MDSCs arise from neutrophils (or vice-versa), even though the real biology may reflect activation or functional reprogramming rather than a linear differentiation process. In

addition, the authors use “Pre-MDSC” but do not define the population. Further, Monocle relies heavily on dimensionality reduction and the construction of a minimum-spanning tree, which means the inferred trajectory depends strongly on how the algorithm embeds the data and on the choice of root cell. If the starting point is mis-specified, the directionality of the pseudotime is effectively arbitrary and the “differentiation path” can be a computational artifact rather than a true biological progression. Given that the starting point is not defined by the authors nor is what “early myeloid”, “pre-MDSC”, this data is impossible to interpret.

We sincerely thank the reviewer for these insightful and critical comments regarding our use of pseudotemporal trajectory analysis. We fully acknowledge and appreciate the fundamental methodological limitations raised, particularly concerning cell plasticity and the interpretative challenges of such computational approaches. Our goal was not to assert a rigid, unidirectional differentiation pathway but to use this as an exploratory tool to interrogate whether ARID1A deficiency is associated with a transcriptional shift within the granulocyte continuum toward states enriched in immunosuppressive signatures following reference in the field (Alshetaiwi et al., Sci. Immunol. 2020)[3]. In response to the reviewer’s valid concerns, we have revised the manuscript to present the pseudotime analysis strictly as a descriptive visualization of the developmental progression within granulocytic myeloid states.

The reviewer correctly emphasizes that the interpretation of a trajectory depends critically on the definition of its start and key states. The root of our trajectory was defined as an early myeloid state characterized by high expression of canonical early

neutrophil progenitor markers (*Elane*, *Mpo*, and *Prtn3*), a strategy consistent with prior work in the field (Alshetaiwi et al., Sci. Immunol. 2020). We defined a transitional cell state, which aligns with the Pre-MDSC (G-MDSC transition) state identified by Alshetaiwi et al. This state was specifically identified by elevated expression of *Retnlg*, *Pirb* and *Asprv1*. These definitions and marker expression patterns are now detailed in the revised manuscript (Page13, line 334-341) and presented in Supplementary Figures 6A-C. Furthermore, we performed an independent, complementary analysis using Single-Cell Velocity (scVelo). This method infers directional dynamics directly from spliced/unspliced mRNA ratios. Critically, scVelo reconstructed a consistent progression from the early state toward the immunosuppressive granulocyte state (Supplementary Fig. 6D).

We sincerely appreciate the reviewer's expertise, which have significantly improved the rigor and clarity of our manuscript.

(Page13, line 334-341)

"The trajectory was rooted in early myeloid cells, defined as neutrophil progenitors expressing high levels of progenitor markers such as Elane, Mpo, and Prtn3 (Supplementary Fig. 6A). The Pre-MDSC represents a transitional cell type within the granulocytic transcriptional continuum, marked by high expression of Asprv1, Pirb and Retnlg (Supplementary Fig. 6B). PMN-MDSC were characterized as a functional immunosuppressive state acquired by neutrophils under specific tumor

microenvironmental pressures, defined by high expression of Cd84, Arg2, Il1b, and Spi1 (Supplementary Fig. 6C). "

*"To further validate this finding, we performed an independent analysis using Single-Cell Velocity (scVelo), a method that infers directionality in cellular state transitions. ScVelo consistently reconstructed the same developmental trajectory (*Supplementary Fig. 6D*)."*

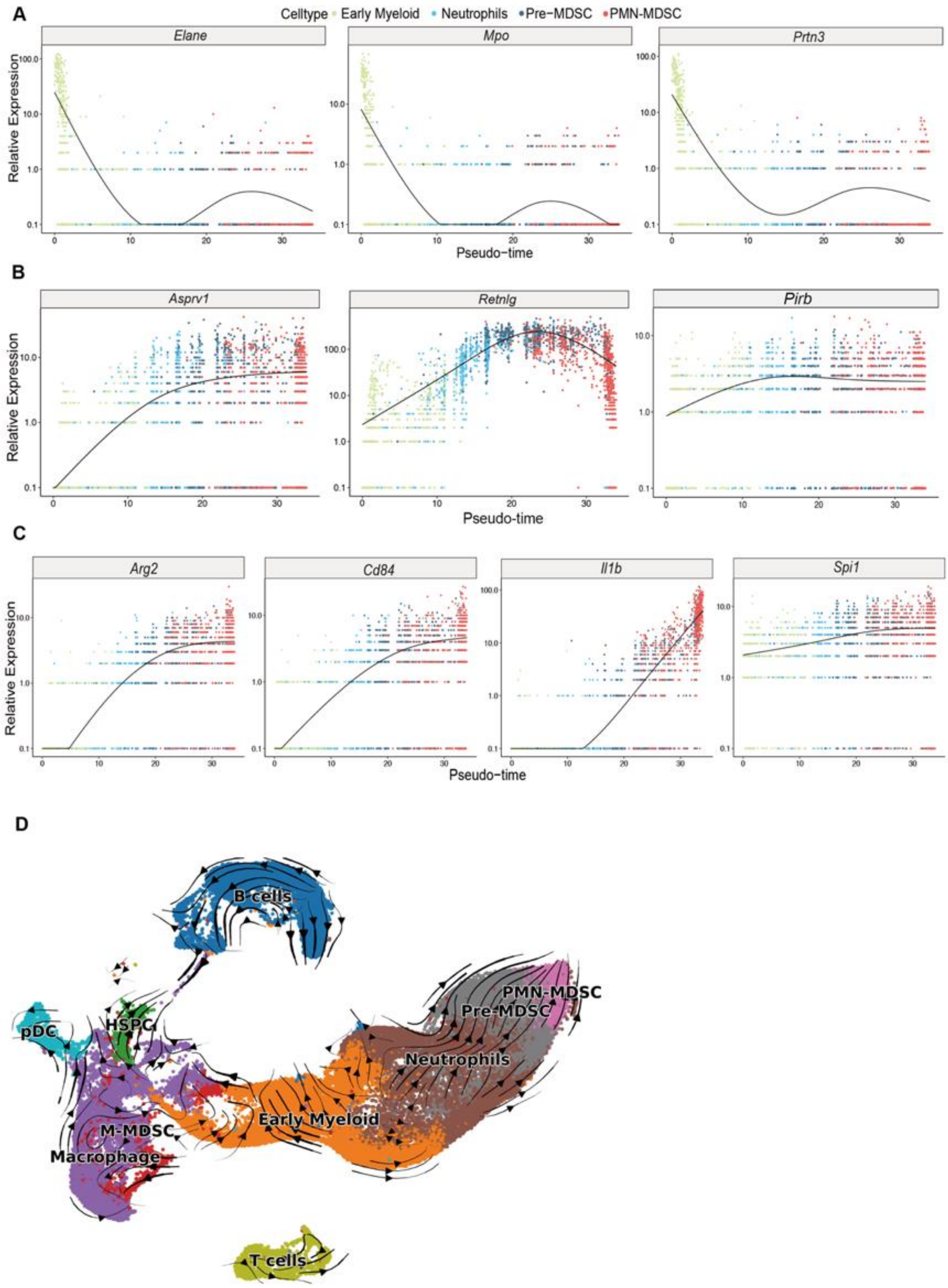

***Supplementary Fig. 6 The developmental trajectory of PMN-MDSCs in TNBC.** (A) Early myeloid cells were marked by high expression of Elane, Mpo, and Prtn3. (**B**) Pre-MDSCs were marked by high expression of Asprv1, Pirb and Retnlg. (**C**) PMN-MDSCs were marked by high expression of Cd84, Arg2, Il1b, and Spi1. (**D**) ScVelo analysis of*

the developmental trajectory within granulocytic myeloid clusters.

The gating and identification of human PMN-MDSCs in figure 5A / S7B is flawed. The gating strategy appears to take a lot of CD11b-cells. Furthermore, CD66 gate looks predominantly like boarder effect/ spread. FMOs are required. As it stands, this is not convincing that there is an increase in PMN-MDSCs.

We thank the reviewer for this detailed and important technical critique regarding the gating strategy for human PMN-MDSCs. In response to the reviewer's point, we performed the recommended FMO controls. The CD11b FMO confirmed our gating strategy was appropriate. Regarding CD66b, the FMO control confirmed the presence of a positive population, rather than non-specific border spread. Based on this reference, we conservatively adjusted the positivity gate downward to more accurately capture this population. We then repeated the key in vitro functional assays for human PMN-MDSCs using this updated gating strategy. Reassuringly, the results from these repeated experiments were fully consistent with our original observations, corroborating the initial biological conclusion. All relevant figures (Fig. 5A; Fig. 6D; Fig. 7F) have been updated in the revised manuscript. The complete gating strategy, including FMO controls, is provided in Supplementary Figure 8.

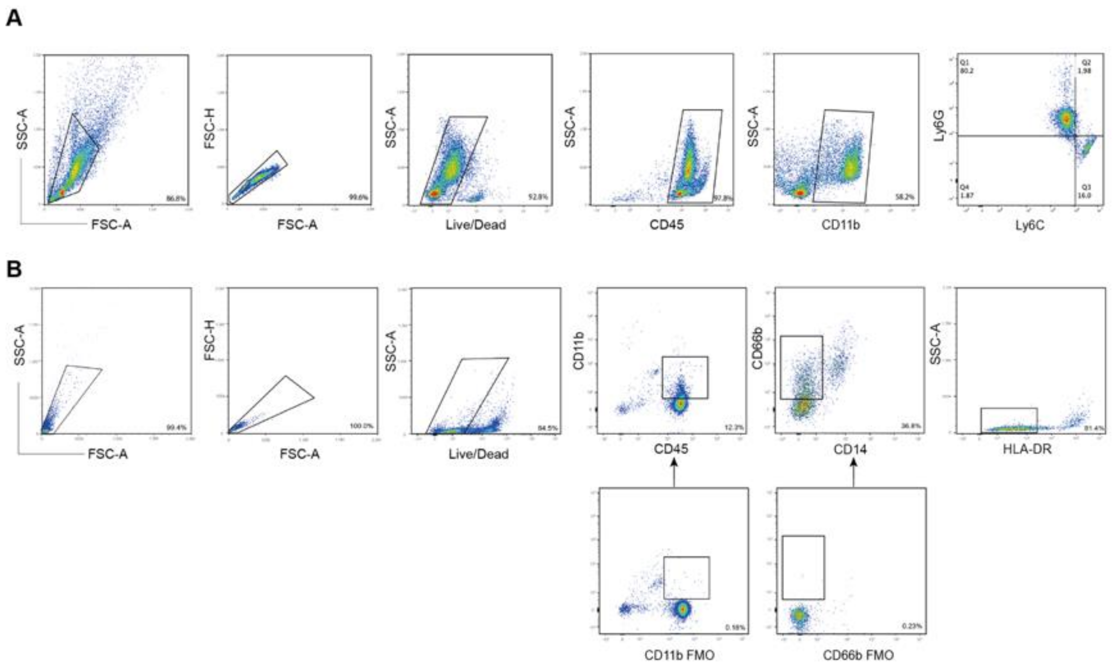

Supplementary Fig. 8 Gating strategies for isolating PMN-MDSCs. *(A) Gating logic for isolating PMN-MDSCs of mice.* ***(B)*** *Gating logic for isolating PMN-MDSCs of humans.*

Mtmv-Cre mice experiments: was there any difference in primary tumour growth between Arid1-ko and Arid1-wt tumours? Were mice euthanized at the same time point (but potentially with different tumour sizes) or at the same tumour size?

We thank the reviewer for this critical question regarding primary tumor growth. In the MMTV-Cre model used in our study, tumors are chemically induced and develop asynchronously. Consequently, to ensure an equivalent endpoint tumor burden for comparative analysis, mice were euthanized individually once their primary mammary tumor reached a predetermined ethical size threshold. The detailed endpoint criteria are provided in the Methods section of the revised manuscript (Page 28, line 783-785).

(Page 28, line 783-785)

"Mice were then euthanized when the primary tumor reached a predefined humane endpoint size to ensure comparable tumor burden at analysis and bone marrow was collected for flow cytometric analysis."

Figure 5C-F - the authors indicate that they "replicate the intracardial injection model" but it is not clear with which cells into which mice therefore the data are uninterpretable. Similarly, for the intratibial injection.

Thank you for this comment. As detailed above**,** the legend for Figure 5 has been revised to explicitly specify the experimental model (intracardiac vs. intratibial injection) used for each panel, eliminating any ambiguity.

Do the tumour cells grow the same in the presence of CB-1158 and DFMO? Do they curtail PMN-MDSCs because the tumour cells die? Perhaps a reductionist / in vitro co-culture experiment is required to fully understand how these interact and to be sure that the effect of CB-1158 and DFMO are not indirect.

Thank you for raising this important point regarding the mechanism of action of these inhibitors. To assess whether CB-1158 and DFMO exert cytotoxic effects on tumor cells, we performed CCK-8 viability assays on TNBC cells (4T-1 and MDA-MB-231) treated with CB-1158 or DFMO. We found that neither CB-1158 nor DFMO showed significant direct cytotoxic effects on these cell lines (Figs. 7A-D). Our data support that the anti-tumor efficacy of these agents is not mediated by direct tumor cell killing

but through the inhibition of key metabolic enzymes in the tumor immune microenvironment. As established in the literature, CB-1158 functions as an arginase inhibitor, reducing the production of ornithine (Tharp et al., Nat Cancer. 2024 Jul)[8], while DFMO inhibits ornithine decarboxylase (ODC1), leading to depletion of polyamines such as spermine (Xu et al., Immunity. 2024 Aug)[9]. By depleting these metabolites in the tumor microenvironment, they cripple the immunosuppressive capacity of PMN-MDSCs, thereby unleashing anti-tumor immunity indirectly. Thus, the reduction in PMN-MDSCs observed in vivo is not a consequence of tumor cell death, but rather the direct pharmacological disruption of their metabolic support network. These results have been added in the revised manuscript (Page 18, line 490-493).

(Page 18, line 490-493)

"First, we assessed whether CB-1158 and DFMO exert cytotoxic effects on tumor cells and found that neither CB-1158 nor DFMO exhibited significant direct cytotoxicity against TNBC cells (Figs. 7A-D)."

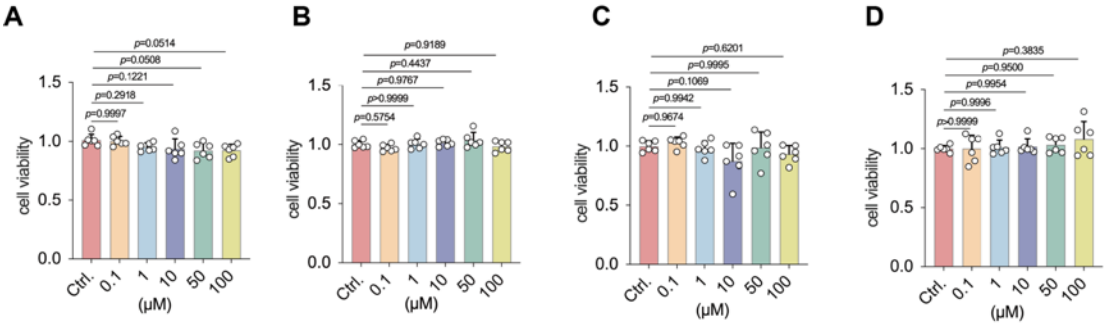

Fig 7. ARG2 and ODC1 inhibitors suppresses ARID1A-deficient TNBC bone metastasis. *(A-B) CCK-8 assays of different concentrations of CB-1158 (A) and DFMO*

(B) on 4T-1 cells for 48h. n=6, per group, one-way ANOVA for multiple comparisons.

(C-D) *CCK-8 assays of different concentrations of CB-1158 (C) and DFMO (D) on MDA-MB-231 cells for 48h. n=6, per group, one-way ANOVA for multiple comparisons.*

Minor comments:

- Figure 1C – no associations with liver, lung, brain or LN mets. Looks like this is because it is underpowered and there could be an association with liver.

We thank the reviewer for this critical comment. To avoid overinterpretation, we have revised the text pertaining to Figure 1C to clarify that ARID1A deficiency did not show a statistically significant correlation with liver, lung, brain, or lymph node metastasis in our study cohort (Page 6, line 129-133). The reviewer's point about potential undetected associations is well-taken and will be noted as a consideration for future studies with larger cohorts.

(Page 6, line 129-133)

"Further, ***no statistically significant*** *associations were observed in the liver (Supplementary Fig. 1A; p=0.0803), lung (Supplementary Fig. 1B, p=0.9222), brain (Supplementary Fig. 1C, p=0.6910), or lymph node metastases (Supplementary Fig. 1D, p=0.5685) between ARID1A wildtype and ARID1A-mutated groups (Supplementary Table 1)."*

- Figure 4D – the text says: "we found the could granulocytic myeloid cells in the

Arid1ko group was significantly elevated compared to that of Arid1NC group”. But there is no quantification. T-SNE intensity call also reflect shifts in populations

We thank the reviewer for this comment. We have added the quantitative analysis in the revised manuscript (Page 13, line 327-329).

(Page 13, line 327-329)

“We found that the count of granulocytic myeloid cells in the Arid1a^{KO} group was significantly elevated compared to that in the Arid1a^{NC} group (37.11% vs 40.03%, $p<0.001$, Fisher’s exact test. Fig. 4D), while the other types of cells showed no difference.”

Reviewer #3 (Remarks to the Author); expert in cancer metabolism:

Bone metastasis represents a lethal complication of advanced breast cancer. Pan et al. elucidated a novel mechanism for bone metastasis. They found that the AT-rich interactive domain-containing protein 1A (ARID1A) remodeled the immunosuppressive metastatic microenvironment through upregulation of arginine metabolism. In their cohort study involving 663 patients with metastatic breast cancer, circulating tumor DNA (ctDNA) analysis revealed a significant positive correlation between ARID1A mutations and metastatic incidence. Notably, most of these mutations resulted in ARID1A loss-of-function, while ARID1A knockdown in tumor cells markedly activated arginine metabolic pathways—particularly those governing

ornithine and spermine synthesis. The researchers further demonstrated that tumor-derived ornithine and spermine drove the expansion of polymorphonuclear myeloid-derived suppressor cells (PMN-MDSCs) in the bone marrow microenvironment, thereby promoting osteotropic metastasis. Importantly, pharmacological inhibition of key arginine metabolic enzymes—arginase 2 (ARG2) and ornithine decarboxylase 1 (ODC1)—significantly attenuated bone metastasis in preclinical models. The findings provide novel insights into metastatic microenvironment remodeling and open new avenues for preventing bone metastasis in breast cancer patients. However, several concerns should be further addressed:

1. While genomic alterations in ARID1A were linked to metastasis, the study did not explicitly address whether corresponding changes in ARID1A protein expression similarly correlate with metastatic progression. Dose ARID1A protein downregulation in primary tumors or metastatic sites serve as a functional biomarker for, or contributor to, breast cancer bone metastasis?

Thank you for raising this important question regarding the relationship between ARID1A protein expression and bone metastasis. We analyzed ARID1A protein expression using a set of tissue microarray containing samples from 108 TNBC patients. During follow-up, we observed that bone metastasis occurred in 23.5% (12/51) of patients with high ARID1A expression, compared to 49.1% (28/57) of patients with low ARID1A expression. This difference was statistically significant (p=0.0060, chi-square test), supporting the conclusion that downregulation of ARID1A protein is significantly associated with and may promote bone metastasis in TNBC. This result

has been present in Figure 1I, with detailed descriptions on Page 7, line 147-151 in the revised manuscript.

(Page 7, line 147-151)

"To test this hypothesis at the protein level, we analyzed ARID1A expression in a set of tissue microarray (TMA) comprising 108 TNBC patients. Supporting our genetic findings, patients with low ARID1A expression exhibited a significantly higher incidence of bone metastasis (28/57, 49.1%) than those with high expression (12/51, 23.5%, p=0.0060; Fig. 1I)"

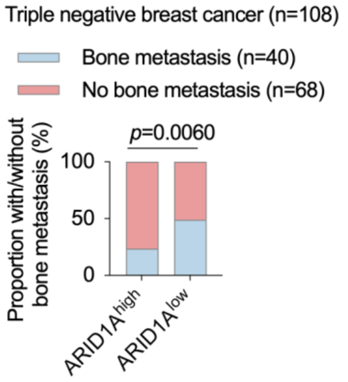

Fig 1. ARID1A mutations promote bone metastasis in breast cancer patients. (I) *The association between ARID1A protein expression levels and bone metastasis in TNBC patients. Pearson's chi-square test was used.*

2. Pan et al. reported an inverse correlation between ARID1A expression and the levels of arginine metabolic enzymes ARG2/ODC1 in metastatic breast cancer. The molecular mechanisms underlying ARID1A-mediated regulation of arginine metabolism were

still unclear. Does ARID1A directly repress ARG2/ODC1 transcription through chromatin remodeling?

Thank you for raising this critical question regarding the molecular mechanism. To investigate the chromatin-based mechanism by which ARID1A regulates arginine metabolism, we performed ATAC-seq and CHIP-seq analysis in ARID1A^{KO} and control MDA-MB-231 cells (GSE234179). Consistent with its role as a core subunit of the canonical BAF (cBAF) complex, ARID1A deficiency led to a global reduction in chromatin accessibility at SWI/SNF-dependent regions. However, at the specific regulatory region of ARG2, H3K27ac CHIP signals were significantly elevated in ARID1A^{KO} MDA-MB-231 cells compared to control cells. Integrated analysis of ChIP-seq and ATAC-seq revealed that ARID1A deficiency facilitates the recruitment of transcription factor ARID3A to the enhancer region within the regulatory domain of ARG2, thereby promoting its expression. The resultant accumulation of ornithine fuels its enzyme ODC1, thereby driving the arginine metabolic pathway. These results are now included as as Figs. 3E-G and supplementary Figs. 4E-I, with the corresponding descriptions in the revised manuscript (Page 9, Line 221-249).

(Page 9, Line 221-249)

"To explore the potential regulatory mechanism underlying low ARID1A expression-induced ARG2 transcription activation, we performed Assay for transposase accessible chromatin (ATAC)-seq and Chromatin Immunoprecipitation-Sequencing (ChIP-seq) analysis in ARID1A^{KO} and negative control MDA-MB-231 cells. Our analyses revealed

that ARID1A deficiency led to a global reduction in chromatin accessibility, indicating impaired SWI/SNF-dependent chromatin remodeling—consistent with ARID1A's established role as a core subunit of the SWI/SNF complex (Supplementary Fig. 4E). Notably, the H3K27ac enrichment at the specific regulatory region of ARG2 was increased by 10-fold in ARID1A^{KO} MDA-MB-231 cells compared with control cells, indicating enhanced transcriptional potential of ARG2 in the absence of ARID1A (Fig. 3E). Subsequent motif enrichment analysis of this differentially accessible region identified ARID3A as a highly enriched candidate transcription factor. To determine its functional role, we conducted siRNA-mediated knockdown of ARID3A, which significantly reduced the expression of ARG2 (Supplementary Figs. 4F and G). This suggests that ARID3A may serve as a key transcriptional regulator mediating the upregulation of ARG2 following ARID1A deficiency. We next performed ChIP-quantitative polymerase chain reaction (CHIP-qPCR) to investigate the binding of ARID3A to the regulatory region of ARG2 and further localized this binding to a specific enhancer site (Fig. 3F). To determine whether this binding site is functionally required for enhancer activity, we cloned the enhancer fragment containing the wild-type (WT) binding site, alongside a corresponding mutant version (MUT) into the pGL3-promoter luciferase reporter plasmid. Dual-luciferase reporter assays revealed that the luciferase activity driven by the enhancer with the WT site was significantly higher than that with the MUT site, demonstrating that this binding site is essential for ARID3A to activate the enhancer of ARG2 (Fig. 3G and Supplementary Fig. 4H). Consistently, we observed that the H3K27ac signal at this specific binding site was

significantly elevated in ARID1A^{KO} cells compared to ARID1A^{NC} cells (Supplementary Fig. 4I).”

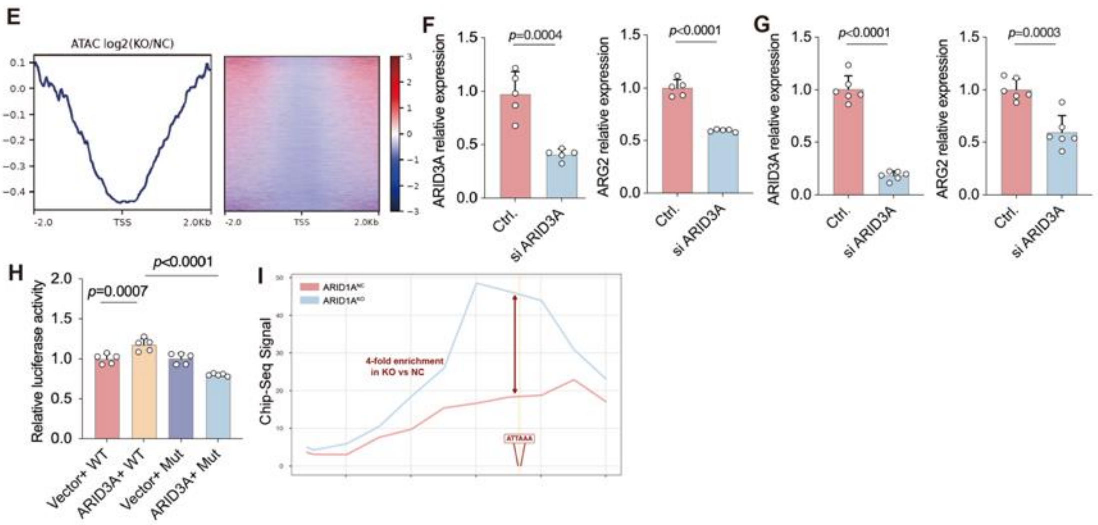

***Supplementary Fig. 4 ARID1A-deficient TNBC upregulates the arginine metabolic pathway.** (**E**) ATAC signal at global chromatin accessibility in ARID1A^{KO} cells compared to ARID1A^{NC} cells. (**F**) ARG2 mRNA expression in MDA-MB-231 cells upon siARID3A knockdown. n=5, unpaired two-tailed Student’s t test. (**G**) ARG2 mRNA expression in BT-549 cells upon siARID3A knockdown. n=6, unpaired two-tailed Student’s t test was used. (**H**) Luciferase activity of the ARG2 enhancer upon ARID3A overexpression in BT-549 cells co-transfected with WT or MUT reporter constructs. n=5 per group, one-way ANOVA for multiple comparisons. (**I**) H3K27ac ChIP−seq signal enrichment at the ARID3A-binding site within the ARG2 enhancer in ARID1A^{KO} and ARID1A^{NC} MDA-MB-231 cells.*

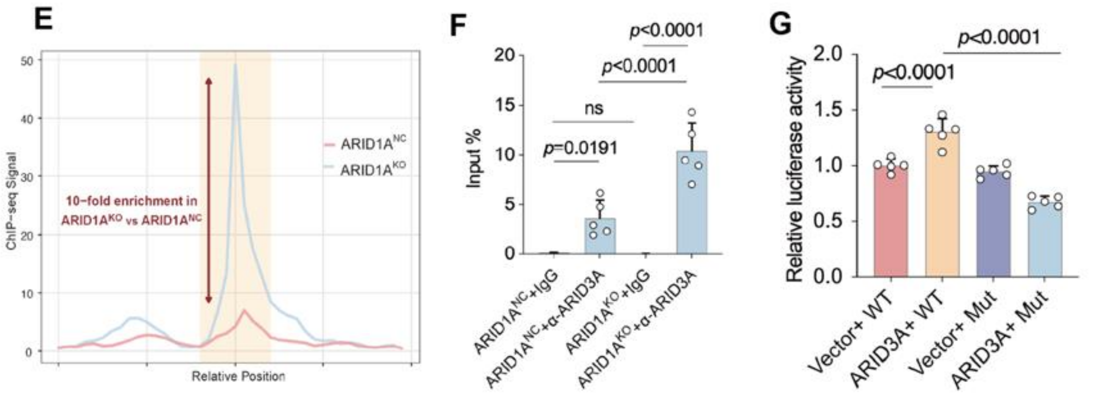

Fig 3. ARID1A deficiency promotes arginine pathway activation and ornithine/spermine production. *(**E**) H3K27ac ChIP−seq signal enrichment coverage at ARG2 regulatory regionpromoter in ARID1A^{KO} and ARID1A^{NC} MDA-MB-231 cells. (**F**) ChIP-qPCR assays of ARID3A binding in the ARG2 enhancer. n=5, two-way ANOVA for multiple comparisons. (**G**) Luciferase activity of the ARG2 enhancer upon ARID3A overexpression in MDA-MB-231 cells co-transfected with WT or MUT reporter constructs. n=5 per group, two-way ANOVA for multiple comparisons.*

3. The authors showed the expression level of ARID1A is negatively correlated with the production of ornithine/spermine and the expansion of PMN-MDSCs. To further substantiate the clinical significance of these findings, they should validate the relationships between ornithine/spermine levels and metastasis incidence, as well as between ARID1A expression levels and the properties of PMN-MDSCs in breast cancer patients.

Thank you for this valuable suggestion to further substantiate the clinical relevance of our findings. We measured the peripheral blood serum levels of ornithine and spermine in 40 TNBC patients. Patients were stratified into high and low-level groups based on

the median concentration of these metabolites. We found that the incidence of bone metastasis was 15.0% (3/20) in the group with low ornithine levels, compared to 60.0% (12/20) in the group with high levels (*p*=0.0033, chi-square test). Similarly, it was 10.0% (2/20) in the low spermine group, versus 65.0% (13/20) in the high spermine group (*p*=0.0003, chi-square test). This suggests that elevated levels of ornithine and spermine are associated with a higher incidence of bone metastasis in TNBC patients. These results have been present in supplementary Figs. 5I and J, with descriptions on Page 12, line 305-311 in the revised manuscript.

Furthermore, we performed multiplex immunofluorescence staining for human PMN-MDSC markers on a set of tissue microarray containing samples from 108 TNBC patients. We observed that tumors with low ARID1A expression contained a significantly higher density of infiltrating PMN-MDSCs compared to those with high ARID1A expression. These results have been present in Figs. 5G and H, with descriptions on Page 15, line 385-390 in the revised manuscript.

(Page 12, line 305-311)

"To dissect the roles of ornithine and spermine in bone metastasis, we measured the peripheral blood serum levels of ornithine and spermine in 40 TNBC patients. Patients were stratified into high and low-level groups based on the median concentration of these metabolites. We found that the incidence of bone metastasis was 15.0% (3/20) in the group with low ornithine levels, compared to 60.0% (12/20) in the group with high

levels (p=0.0033); similarly, it was 10.0% (2/20) in the low spermine group, versus 65.0% (13/20) in the high spermine group (p=0.0003; Supplementary Figs. 5I and J).”

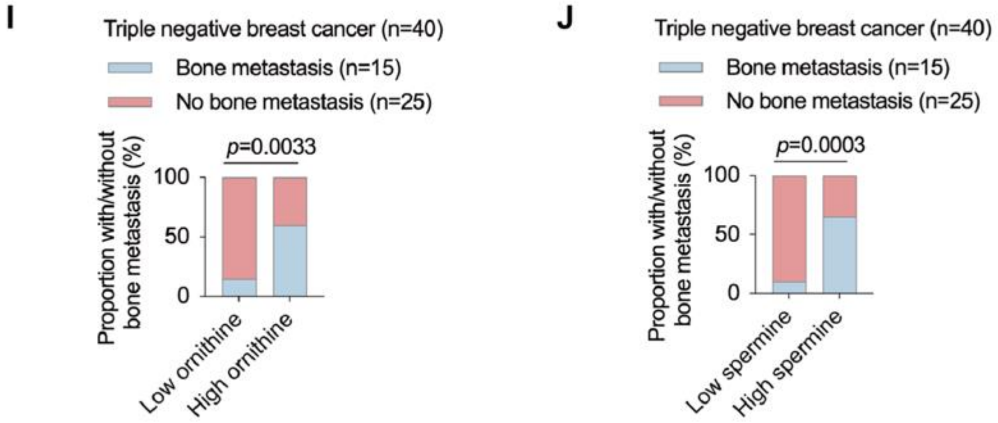

Supplementary Fig. 5 ARID1A deficiency promotes ornithine and spermine production which are associated with bone metastasis in TNBC. (I) *Association between peripheral blood serum ornithine levels and bone metastasis in TNBC patients. n=40, Pearson's chi-square test was used.* ***(J)*** *Association between serum spermine levels and bone metastasis in TNBC patients. n=40, Pearson's chi-square test was used.*

(Page 15, line 385-390)

“Furthermore, to assess the clinical relevance of ARID1A and PMN-MDSCs in breast cancer, we analyzed a set of tissue microarray comprising samples from 108 TNBC patients. Multiplex immunofluorescence staining for human PMN-MDSC markers revealed that tumors with low ARID1A expression exhibited a significantly higher density of PMN-MDSCs compared to those with high ARID1A expressionn (Figs. 5G and H).”

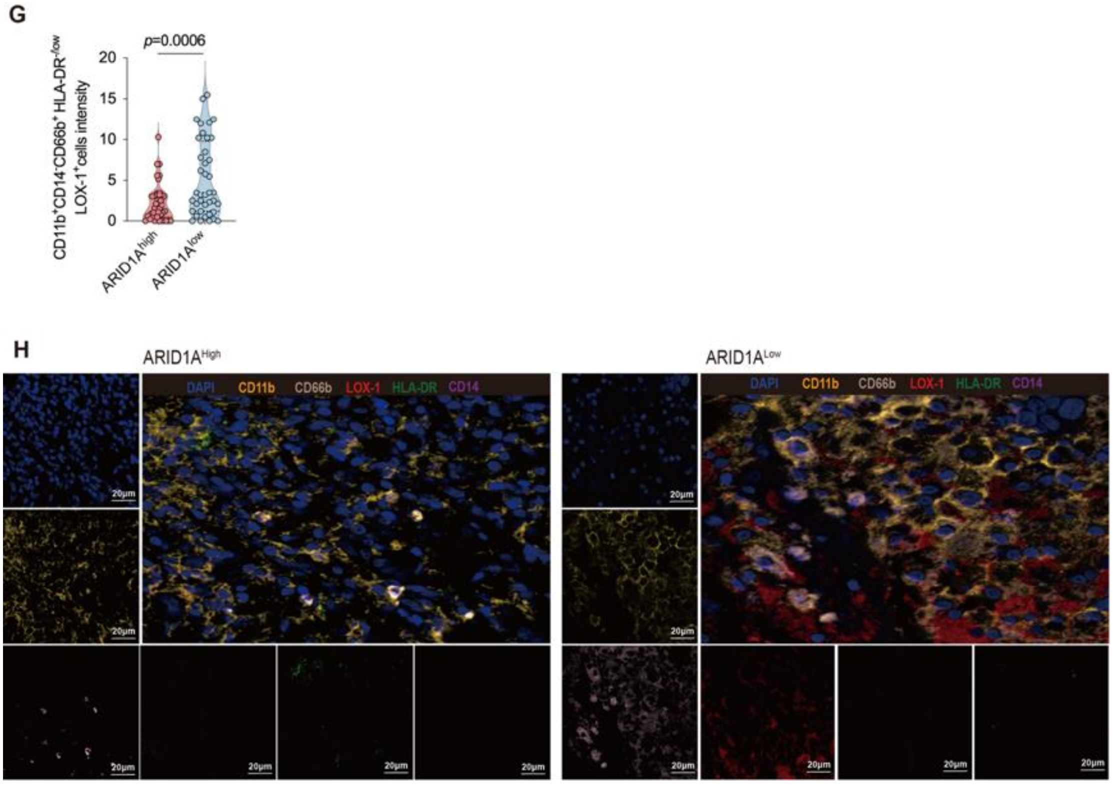

Fig 5. ARID1A deficiency drives the expansion of PMN-MDSCs. (G-H) The proportion of PMN-MDSCs in tumor tissues from TNBC patients with high versus low ARID1A expression was assessed by TSA staining on tissue microarrays, followed by quantitative comparison. Total n=108, unpaired two-tailed Student's t test. Scale bar, 20μm.

4. Knockdown of ARID1A or supplementation of ornithine and spermine in the culture medium promotes the expansion of PMN-MDSCs. To further elucidate the underlying mechanism, it is crucial to verify whether ARID1A regulates PMN-MDSCs expansion through the modulation of ornithine and spermine levels, specifically via the enzymes arginase 2 (ARG2) and ornithine decarboxylase 1 (ODC1).

We appreciate the reviewer for this important mechanistic question. Our data collectively demonstrate that ARID1A deficiency drives PMN-MDSC expansion and

bone metastasis specifically by upregulating the ornithine-spermine metabolic axis via ARG2 and ODC1. First of all, integrated ATAC-seq and ChIP-seq analyses (GSE234179) revealed that ARID1A deficiency facilitates the recruitment of transcription factor ARID3A to the enhancer region within the regulatory domain of ARG2, thereby promoting its expression. Second, we confirmed that ARID1A deficiency results in a marked increase in the concentrations of ornithine and spermine. Third, to functionally test the necessity of this pathway, we employed specific enzyme inhibitors. As established in the literature, CB-1158 functions as an arginase inhibitor, reducing the production of ornithine (Tharp et al., Nat Cancer. 2024 Jul)[8], while DFMO inhibits ODC1, leading to depletion of polyamines such as spermine (Xu et al., Immunity. 2024 Aug)[9]. In our ARID1A-deficient models, treatment with either CB-1158 or DFMO alone significantly reduced bone metastasis burden and diminished PMN-MDSC accumulation. Notably, the combination of both agents produced a additive effect, these results have been present in Figs. 7E-M and supplementary Figs. 13A-C (Page 18, line 493-522). These results confirm that the effects of ARID1A deficiency are mediated through these key metabolic enzymes.

(Page 18, line 493-522)

"Then we focused on their potential immunomodulatory roles in the bone metastatic niche and found that CB-1158 and DFMO exhibited a remarkable capacity to curtail the proportions of PMN-MDSCs in both human and murine in vitro systems (Figs. 7E and F). Further intracardiac inoculation of Arid1a^{KO} 4T-1 cells significantly increased

PMN-MDSC accumulation in murine bone marrow compared to controls, whereas treatment with CB-1158 or DFMO effectively reduced PMN-MDSC proportions (Supplementary Fig. 13A). Furthermore, the increased incidence of bone metastasis triggered by Arid1a knockout was significantly mitigated by administering inhibitors targeting ARG2 and ODC1 (Supplementary Figs. 13B and C). In the intratibial bone metastasis model, CB-1158 and DFMO treatment significantly attenuated intratibial tumor progression [tumor volume: NC, 706.10 ± 29.72 mm3; KO, 901.68 ± 152.39 mm3; CB-1158, 699.93 ± 26.34 mm3; DFMO, 651.29 ± 47.78 mm3; tumor weight: NC, 0.77 ± 0.09 g; KO, 0.97 ± 0.17 g; CB-1158, 0.73 ± 0.03 g; DFMO, 0.69 ± 0.05 g; Figs. 7 G-I]. We next assessed the potential interaction between CB-1158 and DFMO. Their combination demonstrated greater efficacy in reducing bone metastasis incidence (Figs. 7K and L). For the reduction of PMN-MDSCs, although no significant interaction was detected (p=0.3035), the combination was more effective than either agent alone, suggesting an additive rather than a synergistic effect (Fig. 7M). Collectively, these data indicate that the inhibition of ARG2 or ODC1, especially that of their combination, resulted in a significant reduction in the proportion of PMN-MDSCs. This, in turn, substantially impaired the progression of bone metastasis, highlighting the pivotal role of these molecular interventions in disrupting the metastatic cascade within the bone microenvironment.

REDACTED

Fig 7. ARG2 and ODC1 inhibitors suppresses ARID1A-deficient TNBC bone metastasis. *(**A-B**) CCK-8 assays of different concentrations of CB-1158 (A) and DFMO (B) on 4T-1 cells for 48h. n=6, per group, one-way ANOVA for multiple comparisons. (**C-D**) CCK-8 assays of different concentrations of CB-1158 (C) and DFMO (D) on*

MDA-MB-231 cells for 48h. n=6, per group, one-way ANOVA for multiple comparisons. ***(E)*** *Proportions of mouse PMN-MDSCs induced by co-culture with Arid1a^{NC}, Arid1a^{KO} 4T-1 cells, Arid1a^{KO} 4T-1 cells plus CB-1158, and Arid1a^{KO} 4T-1 cells plus DFMO. n=5 per group, one-way ANOVA for multiple comparisons.* ***(F)*** *Proportion of human PMN-MDSCs induced by in vitro co-culture with ARID1A^{NC}, ARID1A^{KO} MDA-MB-231 cells, ARID1A^{KO} MDA-MB-231 cells plus CB-1158, and ARID1A^{KO} MDA-MB-231 cells plus DFMO. n=5 per group, one-way ANOVA for multiple comparisons.* ***(G-I)*** *Image of intratibial tumors in mice injected with Arid1a^{NC} 4T-1 cells, Arid1a^{KO} 4T-1 cells, as well as those treated with CB-1158 and DFMO (G), tumor volume (H), and tumor weight (I). n=5 per group, data are presented as mean ± SD, one-way ANOVA for multiple comparisons.* ***(J)*** *Schematic diagram illustrating the experimental design, including drug administration schedules. bid: twice daily; qd: once daily.* ***(K)*** *BLI and H&E staining images of mice in each group with intracardiac injection. Scale bar, 200μm. B bone, T tumor.* ***(L)*** *Statistical charts presenting the bone metastasis incidence in each group with intracardiac injection. n=5 per group, Fisher's exact test was used.* ***(M)*** *Bone marrow of mice was harvested from each group with intracardiac injection, and analyzed by flow cytometry to quantify PMN-MDSC proportions. n=5 per group, statistical analysis by two-way ANOVA showed no significant interaction between treatments (p=0.3035), other p values are indicated in the figures.* ***(N-Q)*** *Kaplan-Meier survival curves for 108 TNBC patients with low and high level of ARID1A, ARG2, ODC1 and OAT. The log-rank test was employed to*

calculate the difference between the two groups. All p values are indicated in the figures. Source data are provided as a Source Data file.

5. The authors have proposed that ARID1A promotes the expansion of PMN-MDSCs by upregulating the arginine metabolic pathway. To substantiate this hypothesis, it is essential to conduct experiments that specifically inhibit the arginine metabolic pathway within PMN-MDSCs.

We sincerely thank the reviewer for their thoughtful and insightful comment. It is worth noting that the central role of arginine metabolism in PMN-MDSCs has already been proved in prior published work. In the study by Steggerda et al, the authors directly isolated PMN-MDSCs from cancer patients and demonstrated that these cells potently deplete extracellular L-arginine, leading to strong suppression of T-cell proliferation. This depletion is mainly mediated by the high expression, activity and secretion of arginase enzymes in these cells. Crucially, they showed that pharmacological inhibition of arginase with CB-1158 blocked arginine consumption by PMN-MDSCs and fully restored T-cell proliferation in co-culture assays. Further assays confirmed CB-1158's potent inhibition directly targeting the primary enzyme responsible for arginine catabolism in these cells (J Immunother Cancer. 2017)[10]. In the other study by Gao et al. (J Immunother Cancer, 2019), the authors showed that the natural compound Prim-O-glucosylcimifugin (POG) effectively inhibits the immunosuppressive function of PMN-MDSCs by specifically targeting their arginine metabolic pathway[11]. These studies demonstrate that the arginine metabolic pathway

plays an essential role within PMN-MDSCs to execute their immunosuppressive roles in tumor microenviroment.

Reviewer #4 (Remarks to the Author); expert in ctDNA:

I think the science shown is pretty robust and the mechanistic work to support the science is sound. I think the conclusions ignore the fact that most bone metastases don't seem to have this mechanism (as evidenced by 45% of patients with bone mets without ARID1A mutations and a discussion on potential other mechanisms or more acknowledgement for this I would say is important. I think also the clinical significance of this could be discussed in more detail including a discussion of ARG inhibitors and current clinical trials that are underway and have been conducted for these targets.

We sincerely thank the reviewer for their feedback on our work and for these pivotal suggestions, which significantly strengthen the context and impact of our work. We fully agree that our discovery of the ARID1A-ARG2/ODC1-PMN-MDSC axis likely represents one important mechanism driving bone metastasis, but not the exclusive one. Accordingly, we have revised the Discussion to explicitly acknowledge that our model may explain a subset of bone metastasis cases. We have added a paragraph discussing the heterogeneity of bone metastases and outlining potential alternative mechanisms, thereby positioning our findings as characterizing a key molecularly defined subtype (Page 20, line 556-563). Regarding the clinical significance of our findings, the translational discussion in the revised manuscript has been expanded to detail current

and completed clinical trials targeting the arginine metabolic pathway (Page 23, line 631-638). We once again deeply appreciate the reviewer's insightful comments, which have significantly strengthened the clinical relevance of our work.

(Page 20, line 556-563)

"Our findings delineate a coherent molecular axis linking ARID1A deficiency to arginine metabolism-driven immunosuppression and bone metastasis. However, it is important to note that our clinical data indicate among patients without ARID1A mutations, 45.2% of them still developed bone metastasis, suggesting that the mechanism we describe represents a significant yet specific subset. The broader process is driven by diverse and complex synergistic mechanisms, including enhanced angiogenesis, adaptive immune resistance, among others."

(Page 23, line 631-638)

"The additive effect in suppressing PMN-MDSCs by dual inhibition confirms the vulnerability of ARID1A deficient bone metastases to arginine pathway blockade, highlighting a tangible clinical opportunity with several relevant agents already in development. Promising candidates include the arginase inhibitor CB-1158 and the ODC1 inhibitor DFMO. CB-1158 has shown acceptable safety and early signs of immune modulation in Phase I/II trials for solid tumors (NCT02903914; NCT03314935), while DFMO is being actively repurposed in oncology (NCT06059118; NCT06892678; NCT03536728)."

We sincerely thank the reviewers for their thorough evaluation, insightful critiques, and constructive suggestions. Addressing these points has allowed us to strengthen the mechanistic depth, methodological clarity, and overall rigor of our study. We believe the revised manuscript now presents a more robust and compelling account of our findings, and we are grateful for the opportunity to improve it.

Reference:

1. Wu JN, Roberts CW. (2013). ARID1A mutations in cancer: another epigenetic tumor suppressor? Cancer Discov. 3, 35-43. 10.1158/2159-8290.CD-12-0361.

2. Nagarajan S, Rao SV, Sutton J, Cheeseman D, Dunn S, Papachristou EK, Prada JG, Couturier DL, Kumar S, Kishore K, et al. (2020). ARID1A influences HDAC1/BRD4 activity, intrinsic proliferative capacity and breast cancer treatment response. Nat Genet. 52, 187-197. 10.1038/s41588-019-0541-5.

3. Alshetaiwi H, Pervolarakis N, McIntyre LL, Ma D, Nguyen Q, Rath JA, Nee K, Hernandez G, Evans K, Torosian L, et al. (2020). Defining the emergence of myeloid-derived suppressor cells in breast cancer using single-cell transcriptomics. Sci Immunol. 5. 10.1126/sciimmunol.aay6017.

4. Veglia F, Sanseviero E, Gabrilovich DI. (2021). Myeloid-derived suppressor cells in the era of increasing myeloid cell diversity. Nat Rev Immunol. 21, 485-498. 10.1038/s41577-020-00490-y.

5. Lang GT, Jiang YZ, Shi JX, Yang F, Li XG, Pei YC, Zhang CH, Ma D, Xiao Y, Hu PC, et al. (2020). Characterization of the genomic landscape and actionable mutations

in Chinese breast cancers by clinical sequencing. Nat Commun. 11, 5679. 10.1038/s41467-020-19342-3.

6. Li N, Liu Q, Han Y, Pei S, Cheng B, Xu J, Miao X, Pan Q, Wang H, Guo J, et al. (2022). ARID1A loss induces polymorphonuclear myeloid-derived suppressor cell chemotaxis and promotes prostate cancer progression. Nat Commun. 13, 7281. 10.1038/s41467-022-34871-9.

7. Zhang R, Dong M, Tu J, Li F, Deng Q, Xu J, He X, Ding J, Xia J, Sheng D, et al. (2023). PMN-MDSCs modulated by CCL20 from cancer cells promoted breast cancer cell stemness through CXCL2-CXCR2 pathway. Signal Transduct Target Ther. 8, 97. 10.1038/s41392-023-01337-3.

8. Tharp KM, Kersten K, Maller O, Timblin GA, Stashko C, Canale FP, Menjivar RE, Hayward MK, Berestjuk I, Ten Hoeve J, et al. (2024). Tumor-associated macrophages restrict CD8(+) T cell function through collagen deposition and metabolic reprogramming of the breast cancer microenvironment. Nat Cancer. 5, 1045-1062. 10.1038/s43018-024-00775-4.

9. Xu H, Zhang X, Wang X, Li B, Yu H, Quan Y, Jiang Y, You Y, Wang Y, Wen M, et al. (2024). Cellular spermine targets JAK signaling to restrain cytokine-mediated autoimmunity. Immunity. 57, 1796-1811 e1798. 10.1016/j.immuni.2024.05.025.

10. Steggerda SM, Bennett MK, Chen J, Emberley E, Huang T, Janes JR, Li W, MacKinnon AL, Makkouk A, Marguier G, et al. (2017). Inhibition of arginase by CB-1158 blocks myeloid cell-mediated immune suppression in the tumor microenvironment. J Immunother Cancer. 5, 101. 10.1186/s40425-017-0308-4.

11. Gao W, Zhang X, Yang W, Dou D, Zhang H, Tang Y, Zhong W, Meng J, Bai Y, Liu Y, et al. (2019). Prim-O-glucosylcimifugin enhances the antitumour effect of PD-1 inhibition by targeting myeloid-derived suppressor cells. J Immunother Cancer. 7, 231. 10.1186/s40425-019-0676-z.

ADDITIONAL REVIEWER COMMENTS

Reviewer #1 (Remarks to the Author):

I thank the authors for addressing all the points raised and for their efforts generating several new in vivo datasets which have strengthened the manuscript.

In particular, the experiments using BRM014 inhibitors add interesting molecular insights into the activity of the residual SWI/SNF chromatin remodelling complexes which lack Arid1a subunits. These results also support the authors model which suggests that Arid1a is the main driver of this mechanism rather than the SWI/SNF complex as a whole. Also, the explanation for the unique sensitivity of PMN-MDSCs to ornithine and spermidine treatments is interesting as are the new experiments revealing an additive effect of the inhibitors on metastasis.

Finally, while the new details on the analysis of the RNA-seq, ChIP-seq and ATAC-seq is an important addition, it would be interesting to also include a genome-browser view of just the Arg2 locus to integrate all these data in one figure panel. Having a

single locus overview of the H3K27ac levels, chromatin accessibility and gene expression would help support the slightly counter intuitive model whereby Arid1a loss leads to de-repression at the Arg2 locus.

We sincerely thank the reviewer for the very positive and constructive feedback. We fully agree with this constructive suggestion. In the revised manuscript, we have integrated the H3K27ac ChIP-seq signal enrichment at the *ARG2* regulatory region, chromatin accessibility, and gene expression into a single panel (Fig. 3). This integrated view clearly illustrates that *ARID1A* deficiency leads to de-repression at the *ARG2* locus.

Once again, we greatly appreciate the reviewer's time, expertise, and insightful comments throughout the review process.

Reviewer #2 (Remarks to the Author):

I want to commend the authors on addressing thoroughly all the previous comments. This work is very interesting and provides novel insight into the mechanisms by which tumours induce an immunosuppressive microenvironment.

The authors have addressed my comments sufficiently.

We greatly appreciate the reviewer's very positive comments and acknowledgment of our revisions. Thank you for your time and valuable input.

Reviewer #3 (Remarks to the Author):

The authors have addressed all my concerns.

We greatly appreciate the reviewer's thoughtful and constructive comments throughout the review process, which have helped us improve the quality of our work.
